# Fake paper identification in the pool of withdrawn and rejected manuscripts submitted to *Naunyn–Schmiedeberg’s Archives of Pharmacology*

**DOI:** 10.1007/s00210-023-02741-w

**Published:** 2023-10-05

**Authors:** Jonathan Wittau, Serkan Celik, Tim Kacprowski, Thomas M. Deserno, Roland Seifert

**Affiliations:** 1 Institute of Pharmacology, Hannover Medical School, Carl-Neuberg-Straße 1, 30625 Hannover, Germany; 2 Braunschweig Integrated Centre of Systems Biology, TU Braunschweig, Braunschweig, Germany; 3 Peter L. Reichertz Institute for Medical Informatics of TU Braunschweig and Hannover Medical School, 38106 Braunschweig, Germany

**Keywords:** Fake paper, Paper mill, *Naunyn–Schmiedeberg’s Archives of Pharmacology*, Withdrawn, Rejected, Scientific misconduct

## Abstract

Honesty of publications is fundamental in science. Unfortunately, science has an increasing fake paper problem with multiple cases having surfaced in recent years, even in renowned journals. There are companies, the so-called paper mills, which professionally fake research data and papers. However, there is no easy way to systematically identify these papers. Here, we show that scanning for exchanged authors in resubmissions is a simple approach to detect potential fake papers. We investigated 2056 withdrawn or rejected submissions to *Naunyn–Schmiedeberg’s Archives of Pharmacology (NSAP)*, 952 of which were subsequently published in other journals. In six cases, the stated authors of the final publications differed by more than two thirds from those named in the submission to *NSAP*. In four cases, they differed completely. Our results reveal that paper mills take advantage of the fact that journals are unaware of submissions to other journals. Consequently, papers can be submitted multiple times (even simultaneously), and authors can be replaced if they withdraw from their purchased authorship. We suggest that publishers collaborate with each other by sharing titles, authors, and abstracts of their submissions. Doing so would allow the detection of suspicious changes in the authorship of submitted and already published papers. Independently of such collaboration across publishers, every scientific journal can make an important contribution to the integrity of the scientific record by analyzing its own pool of withdrawn and rejected papers versus published papers according to the simple algorithm proposed in the present paper.

**Supplementary Information:**

The online version contains supplementary material available at 10.1007/s00210-023-02741-w.

## Introduction

Research builds on knowledge gained in previous studies. Scientific publications are the primary sources of this knowledge. Unfortunately, an increasing number of fake papers is contaminating the scientific literature (Else and Van Noorden 2021). Fake papers contain fictitious and manipulated data. Companies called paper mills professionally produce fake papers and publish them in the name of paying customers. Paper mills thus offer the opportunity to become an author of scientific publications without conducting research (Else and Van Noorden 2021; Byrne and Christopher 2020). Reasons for turning to paper mills include pressure to publish, lack of time for research, and financial and career benefits (Tian et al. 2016; Lin 2013; Quan et al. 2017).

In recent years, several fake paper cases have been discovered (Else and Van Noorden 2021). *Naunyn–Schmiedeberg’s Archives of Pharmacology* (*NSAP*) was affected by paper mill submissions too. In 2020 and 2021, the journal retracted 11 publications due to a paper mill involvement (Seifert 2021). It is hard to say how many scientific publications are fake. In a report from 2022, the proportion of fake papers is estimated at about 2% (COPE & STM 2022). Other researchers even estimate the share of potential fakes to be up to 28% (Sabel et al. 2023).

It is important to retract fake papers and prevent their further publication. Unfortunately, there is a lack of ways to identify them easily. Current approaches include the identification of manipulated images, the detection of fake reviewers, or a mix of signs that indicate possible fakes (Byrne and Christopher 2020; Seifert 2021; Christopher 2021; Day 2022). Often fake papers are just discovered by chance (Seifert 2021). Based on its own experience in dealing with fake papers, *NSAP* has published a list of 20 features observed among these papers. Strikingly, one paper withdrawn from *NSAP* had also been submitted to another journal but with completely different authors (Seifert 2021).

In this study, we systematically searched for similar cases, i.e., publications that were also submitted to *NSAP*, but with extensively differing lists of authors. We show that this is an easy method to systematically scan for papers with possible paper mill involvement.

## Methods

### Identification of resubmissions with extensively differing authorship

We searched for publications that had been rejected by, or withdrawn from, *NSAP* but were subsequently published in a different journal with a different authorship. The following paragraphs briefly describe how we automated the search for such cases (Celik 2022).

**Fig. 1:**
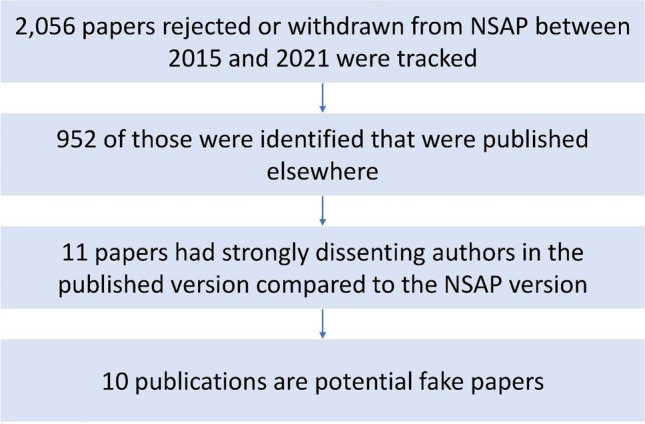
Workflow overview

We analyzed all unpublished papers submitted to *NSAP* between 2015 and 2021 (Fig. 1). Titles, abstracts, and authors were extracted from the *NSAP* manuscripts. For each abstract, two summaries were calculated using extractive (TextRank) and abstractive (Pegasus) methods (Mihalcea and Tarau 2004; Zhang et al. 2020). One summary contained an extraction of the most relevant keywords; the other was a semantically similar text that was generated. To find publications similar to the *NSAP* submissions, the databases of PubMed (https://pubmed.ncbi.nlm.nih.gov), Semantic Scholar (https://www.semanticscholar.org), and Google Scholar (https://scholar.google.com) were queried using the titles, abstracts, and previously calculated summarizations.

We also used Solr (https://solr.apache.org/) as an additional database to index all publications from PubMed by using their abstracts, titles, and authors. Furthermore, we indexed the biomedical entities of these publications by using PubTator Central (Wei et al. 2019). The integration of PubTator Central allowed the consideration of different names for the same biomedical entity. PubTator Central was also used to identify the biomedical entities of the *NSAP* titles and abstracts. Solr was then queried, using the titles, abstracts, and biomedical entities of the *NSAP* manuscripts to find further similar publications.

Semantic similarity was also taken into account through the use of abstractive summarization methods and the integration of PubTator Central. To find resubmissions among the publications we retrieved, we compared titles and abstracts of these publications to those of the *NSAP* papers using a combination of different approaches. We calculated the Jaccard coefficient to identify resubmissions based on textual similarity. The Universal Sentence Encoder was used to integrate semantic similarity (Cer et al. 2018). Moreover, we trained an LSTM using a pre-trained FastText model for the embedding, in which similar words lie close together in the embedding space (Bojanowski et al. 2017). We then automatically preselected all resubmissions that had a different list of authors than their corresponding *NSAP* submissions.

### Evaluation of the identified cases

To verify that papers discovered automatically were indeed versions of the corresponding manuscripts that were submitted to *NSAP*, we performed a manual follow-up evaluation. For this purpose, we marked identical text, identical figures, differences in content, and paraphrasing with different colors. We then verified that we had detected publications of the same papers submitted to *NSAP*.

The similarity of authors was calculated using the Jaccard coefficient (number of consistent authors appearing in both versions of a paper divided by the number of authors in both versions of a paper). A similarity of 1 means that the list of authors is identical in both versions. A similarity of 0 means that the lists of authors are disjunct. According to this measure, we selected papers with discrepancies in authorship of more than two thirds.

### Communication with authors and journals

We contacted the authors and journals of the suspected cases. We wrote to a corresponding author of each publication with some simple questions related to the content of the paper. These emails were sent on March 30, 2023. Our aim was to verify the validity of the email addresses provided and to assess the authors’ familiarity with the publication. We pretended to be doctoral students conducting research in a similar field. We also informed the journals that had published these papers about our findings, bringing the authorship manipulation to their attention and asking them to investigate these cases. The emails to the journals were sent on March 15, 2023, by the editor-in-chief of *NSAP* to ensure an official and reputable appearance. The editors-in-chief of the respective journals received a detailed report on our findings as well as the highlighted versions of the *NSAP* paper and the published paper so that they could quickly make up their own opinion.

## Results

### Paper overview

**Fig. 2:**
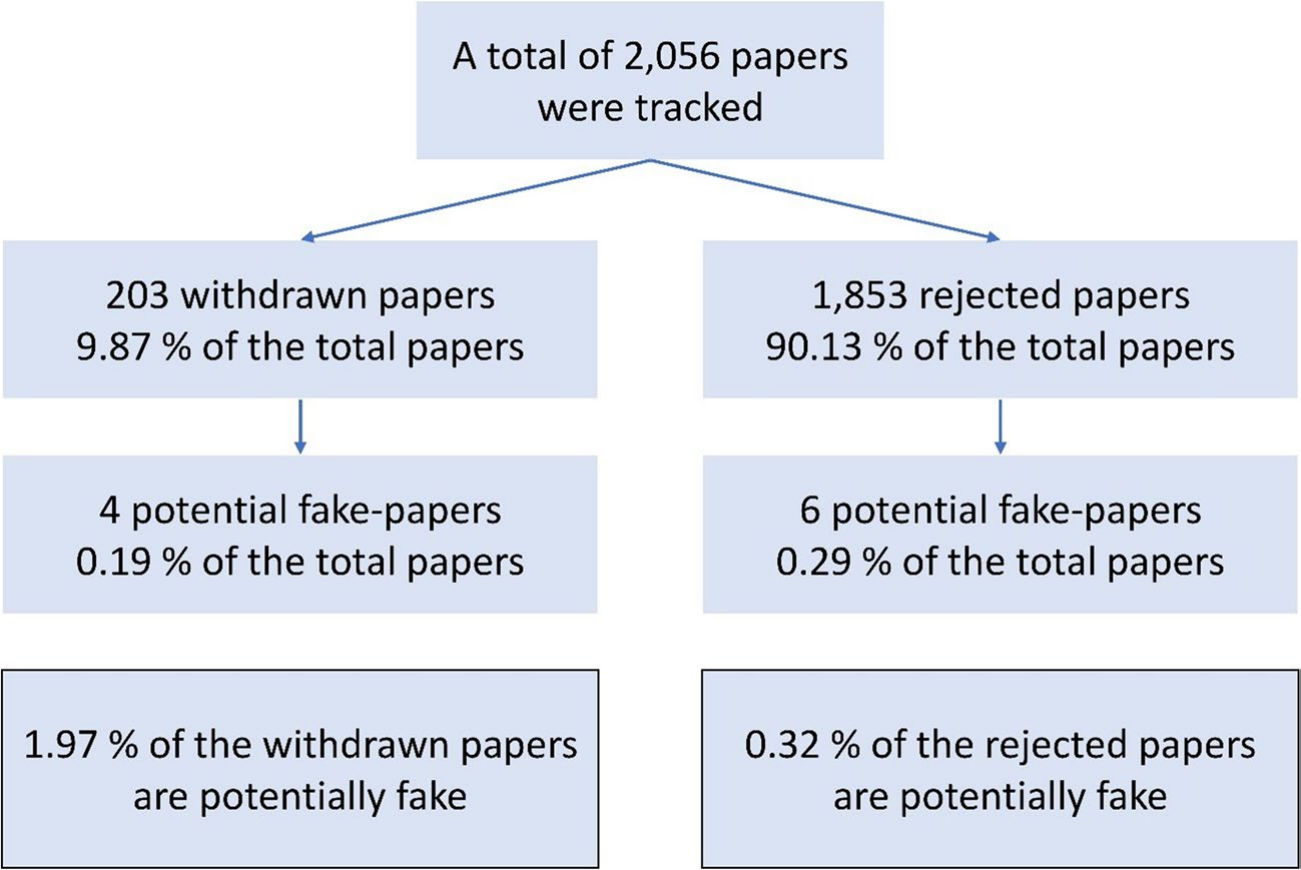
Proportion of potential fake papers

In total, 2056 unpublished manuscripts submitted to *NSAP* were investigated, of which 203 were withdrawn by the authors and 1853 were rejected by *NSAP* (Fig. 2). We identified 952 resubmissions using majority voting of all classifiers described above. In 11 cases, the list of authors differed with a Jaccard coefficient of more than two thirds. We manually identified 10 papers having similar content (Table 1, Supplementary Figures S1-S10). We ordered the papers as follows: First come the papers where all of the authors were replaced (**1,2,3,4**) and then the papers where some of the authors were replaced (**5,6,7,8,9,10**). In seven cases (**1,2,4,5,7,8,9**), the text of both paper versions was almost identical (Table 2). In three cases (**3,6,10**), the text differed more. In the latter, there were completely different sections and some text was paraphrased. In all cases, there were identical figures in both versions, even if not all figures were always identical. Furthermore, we found a second published version of Paper **4** that had already been withdrawn (Yang et al. 2022https://onlinelibrary.wiley.com/doi/10.1002/jbt.23057) (Table 1). We had no possibility to access that paper, but the title and authors were identical to the *NSAP* version.

**Table 1:**
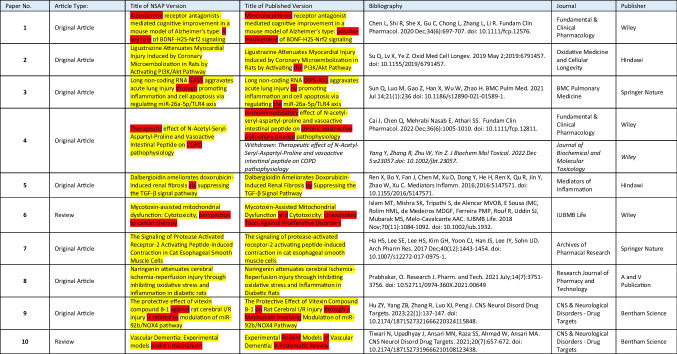
Publications overview

Identical parts of the titles in both paper versions are highlighted in yellow. Differences are highlighted in red

**Table 2:** Similarity of authors, text, and figures

Paper No	Authors (Jaccard coefficients)	Text	Figures
1	0.00	✓	✓
2	0.00	✓	✓
3	0.00	(✓)	✓
4	0.00	✓	✓
5	0.33	✓	✓
6	0.08	(✓)	✓
7	0.19	✓	✓
8	0.25	✓	✓
9	0.27	✓	✓
10	0.25	(✓)	(✓)

Text: ✓, almost equal; (✓), partially equal or rephrased. Figures: ✓, identical; (✓), changes in figure design, but not in content

The incriminated papers were either original articles (**1,2,3,4,5,7,8,9**) or reviews (**6,10**) and were published by various publishers (Table 1).

*NSAP* did not publish the papers for various reasons (Table 3). Two papers were withdrawn by the authors without explanation (**2,3**), one was considered withdrawn because the authors did not report back to *NSAP* (**1**), and in one case we did not find out the reason for the withdrawal (**5**). Rejections occurred, when original data requested by the reviewers or editors were not provided (**4,9**) or when signs of plagiarism were detected (**6,8**). Reviewers also criticized a paper as deficient (**10**) or even raised concerns about data credibility (**9**).

**Table 3:** Reasons for rejection/withdrawal at *NSAP*

Paper No	Reason
1	The paper was considered withdrawn as the authors did not respond to the revision instructions and ignored several attempts to contact them
2	The paper was withdrawn by the authors without giving a reason
3	The paper was withdrawn by the authors without giving a reason
4	The paper was rejected since the authors did not provide original data
5	The paper has been withdrawn by the authors. Unfortunately, we did not find out if the authors gave a reason for the withdrawal
6	The paper was rejected because plagiarism was detected. In addition, the reviewer did not find it useful or novel
7	The paper was rejected. Unfortunately, we did not find the reason in the rejection report
8	The paper was rejected because it contained a number of phrases taken from other published papers
9	The paper was rejected since the authors did not provide data requested by the reviewers. Furthermore, reviewers reported concerns about the credibility of some data
10	The paper was rejected because reviewers criticized language, form, and content as deficient

**Fig. 3:**
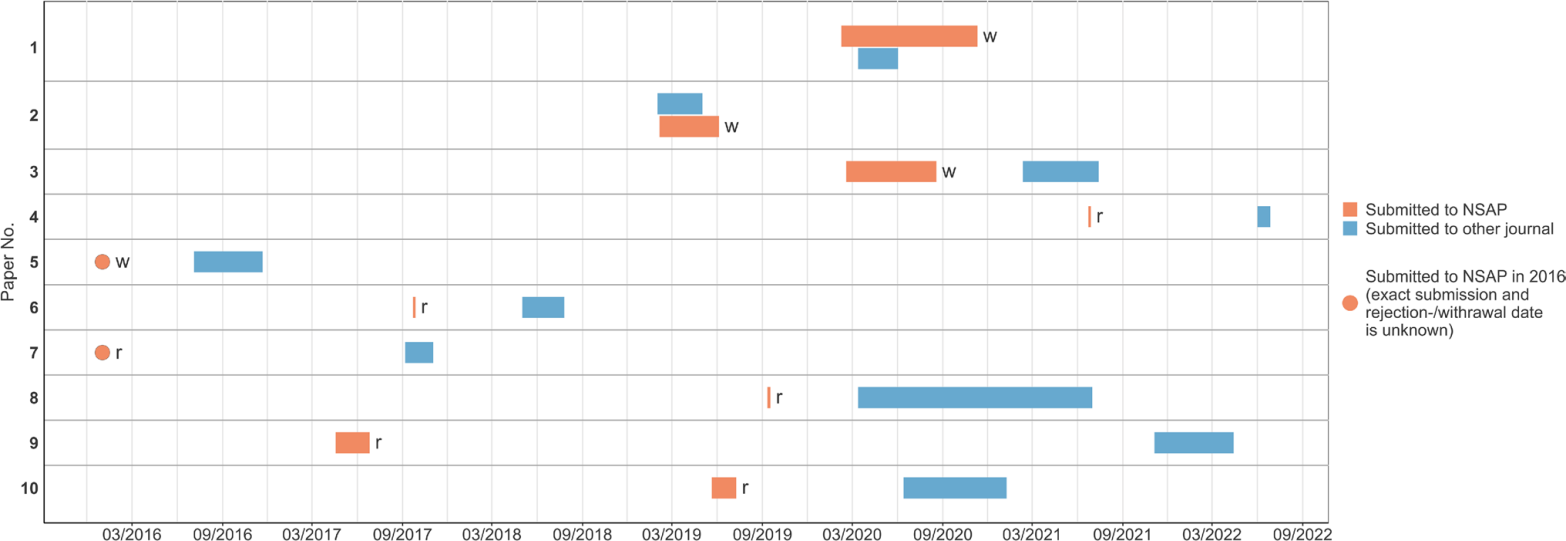
Timeline of the publication process. Each timeline starts with submission to a journal and ends with either rejection (r) or withdrawal (w) at NSAP or publication in another journal

All papers were published between 2016 and 2022 (Fig. 3). In five cases (**3,4,6,8,10**), the papers were submitted to the finally publishing journals no more than a year after rejection/withdrawal by *NSAP*. In one case (**9**), however, more than 4 years had passed. It is also noticeable that two manuscripts (**1,2**) were submitted to two journals at the same time, which violates *NSAP*’s submission guidelines (https://www.springer.com/journal/210/submission-guidelines).

### Exchanged authors

**Fig. 4:**
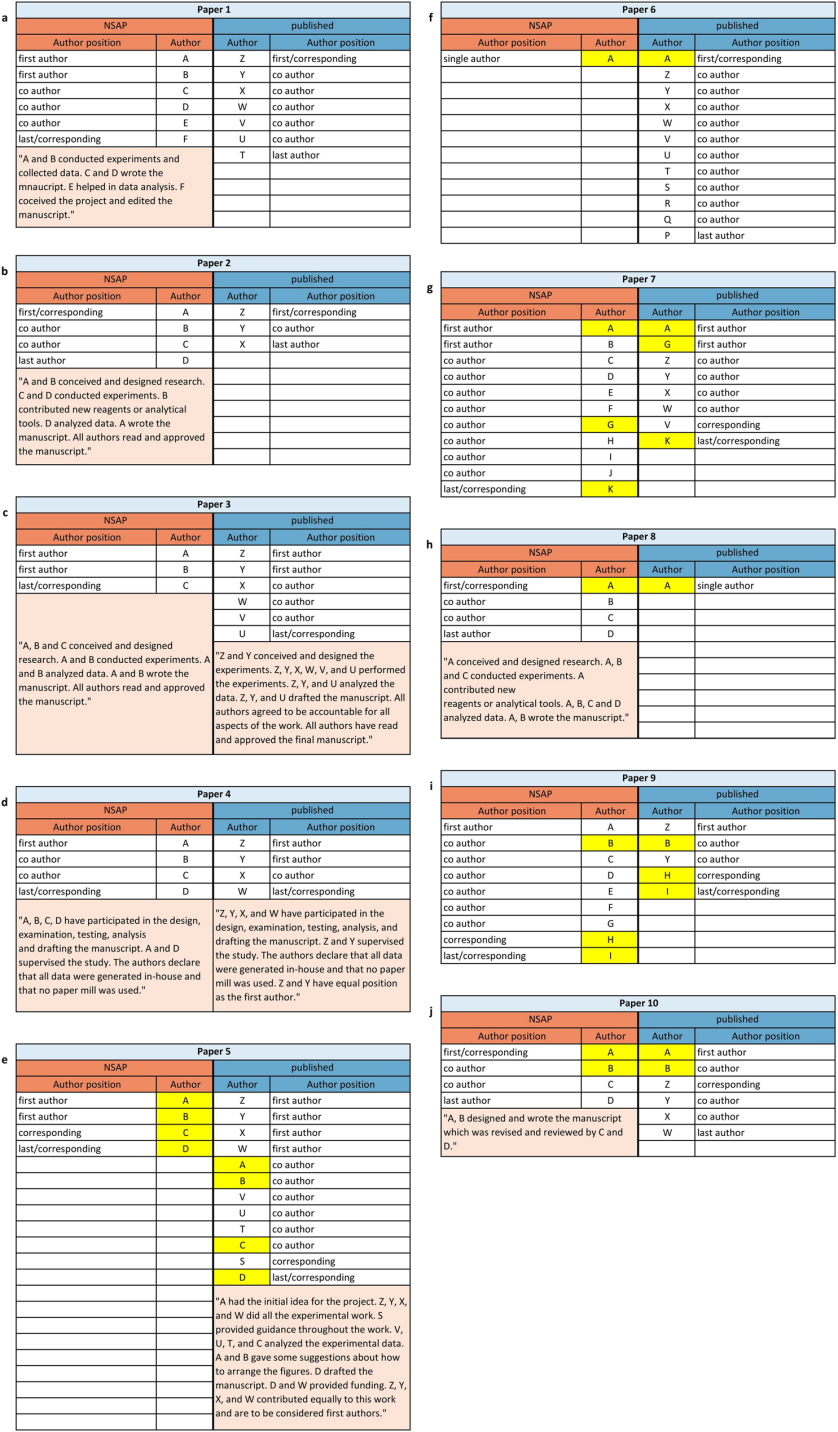
Author comparison. The authors were pseudonymized by letters. If an author appeared in both versions of a paper, the same letter was assigned and marked in yellow. The pseudonyms refer only to both versions of a paper. Author A from publication 1 has nothing to do with author A from publication 2. If available, the Author Contribution Statements were highlighted

**Fig. 5:**
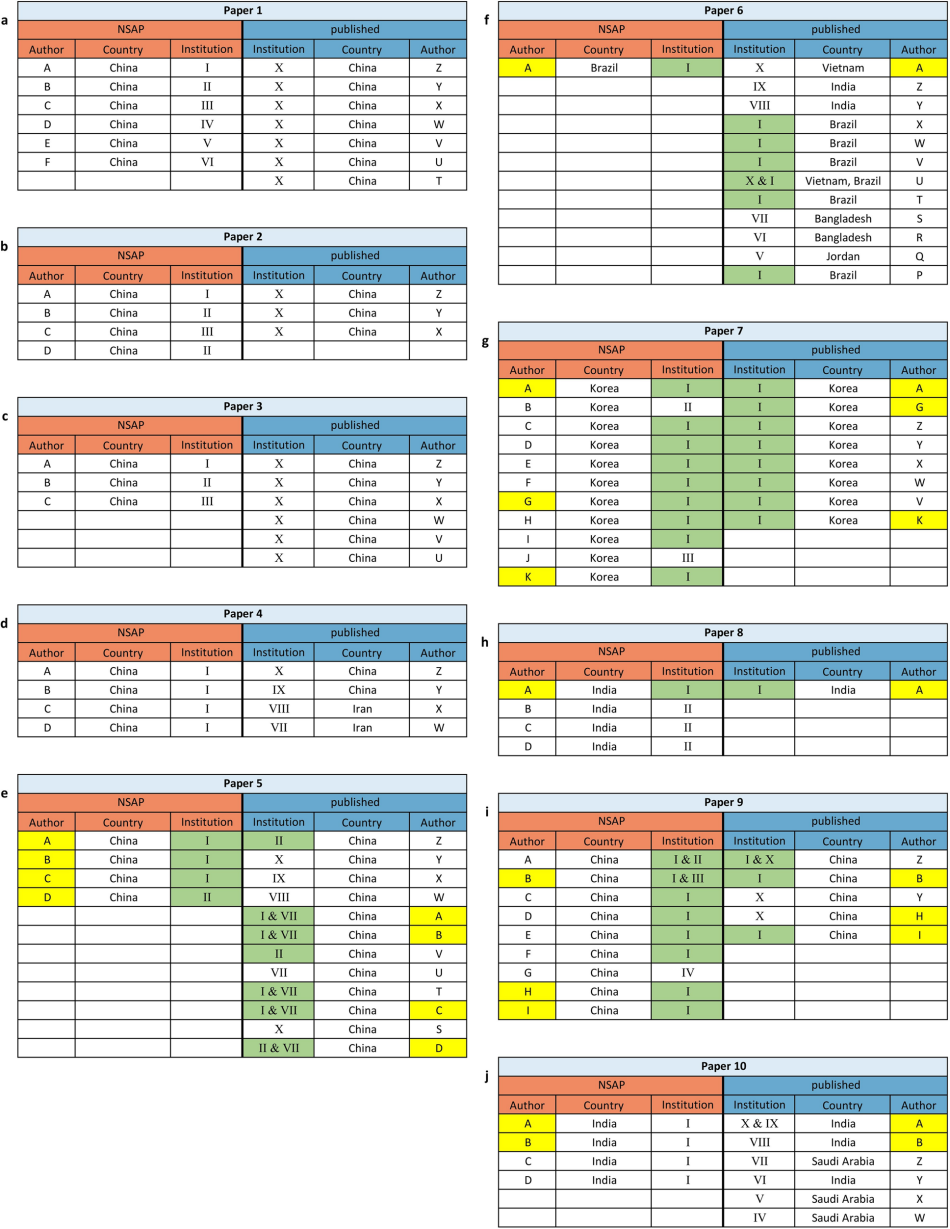
Comparison of the authors’ institutions. The institutions were pseudonymized by Roman numerals. Institution II from publication 1 has nothing to do with institution II from publication 2. If an institution appeared in both versions of a paper, the same Roman number was assigned and marked in green. Hospitals affiliated to a university were considered as a separate institution but just a different institute of the same university or hospital were considered the same institution. The authors are pseudonymized by letters (see Fig. 4)

In all 10 cases, the authors of the papers differed significantly between the version submitted to *NSAP* and the published version (Fig. 4 and Table 2). In four cases (**1,2,3,4**), all authors had been exchanged. In six cases (**5,6,7,8,9,10**), only some of the authors had been exchanged, but considering the small difference between the two paper versions, the lists of authors were far too different to be legitimate. In all cases, the changes in authorship went beyond what is usual in pharmacology (Table 4). In seven cases (**1,2,3,4,5,8,10**), at least one of the two versions of a paper contained an Author Contribution Statement, explaining in detail how each author was involved in the research. These statements cannot be true. Paper **4** even included a statement guaranteeing all data had been generated by the stated authors and not by a paper mill. Since the lists of authors in both versions are completely different, this is obviously not true. In six cases (**1,2,3,4,8,10**), all exchanged authors were replaced by authors from other institutions (Fig. 5). In the other four cases (**5,6,7,9**), some but not all authors were exchanged for authors from the same institution. In three cases (**6,9,10**), even the institutions of some remaining authors changed. The authors came from several countries (*NSAP* version/published version). Most of them were from China (30/35), South Korea (11/8), India (8/6), and Brazil (1/6), but in the published versions there were also authors from Saudi Arabia (0/3), Vietnam, Iran, and Bangladesh (0/2), and Jordan (0/1) (Fig. 5 and Table 5).

**Table 4:** Comparison with usual authorship changes

	Usual changes of authorship in pharmacology:		
	First author	Co author	Last author
	Usually remains the same	Usually remains the same, additions are possible if new data are presented	Usually remains the same
	Do the changes in the authorship of the identified cases correspond to usual changes?		
Paper No	First author	Co-author	Last author
1	No	No	No
2	No	No	No
3	No	No	No
4	No	No	No
5	No	No, new authors have been added, but no new data have been presented	Yes
6	Yes	Yes, but the number of co-authors added does not fit the amount of new data	No
7	No	No	Yes
8	Yes	No	No
9	No	No	Yes
10	Yes	No	No
No. of deviations	7	8	7

Usual changes in authorship of pharmacological papers are indicated by the editor-in-chief of *NSAP*, based on many years of experience

**Table 5:** Origins of both paper versions

	*NSAP*			Published		
Paper No	Country	No. of authors	No. of institutions	Country	No. of authors	No. of institutions
1	China	6	6	China	7	1
2	China	4	3	China	3	1
3	China	3	3	China	6	1
4	China	4	1	China, Iran	4	4
5	China	4	2	China	12	6
6	Brazil	1	1	Vietnam, India, Brazil, Bangladesh, Jordan	12	7
7	Korea	11	3	Korea	8	1
8	India	4	2	India	1	1
9	China	9	4	China	5	2
10	India	4	1	India, Saudi Arabia	6	7

Hospitals affiliated to a university were considered as a separate institution but just a different institute of the same university or hospital were considered the same institution

### Communication with authors and journals

Contacting the authors and journals that had published the papers was not very successful (Table 6). More than 4 months after addressing the corresponding authors of each paper, we still had not received a single answer. In one case (**6**), we got a message that the email address did not exist. Furthermore, only two journals answered (**5,7**). In one case (**5**), the editor-in-chief as well as the publisher’s research integrity team responded after 1 and 3 days, respectively, promising to investigate the matter, but then nothing happened anymore. The other journal (**7**) responded after 79 days. We have been informed that they had investigated the case and found misconduct in the authorship of the *NSAP* version, but not in the published version. A paper mill was not involved in their opinion.

**Table 6:** Communication with authors and journals

Paper No	Author response	Journal response	Response time
1	No	No	
2	No	No	
3	No	No	
4	No	No	
5	No	Yes, we received feedback from the editor-in-chief and from the research integrity team of the publisher. It was promised to investigate the case	Editorial: 1 day Research integrity team: 3 days
6	No, but we received a message that the email address is invalid	No	
7	No	Yes, the editor-in-chief reported us that the case had been investigated. The journal concluded that there had been misconduct in the authorship of the *NSAP* version, but not in the published version. A paper mill was not involved in their opinion	Editorial: 79 days
8	No	No	
9	No	No	
10	No	No	

All journals and authors were contacted on March 15 and March 30, 2023, respectively. The table is of August 01, 2023

### Consequences of our attempts

By August 01, 2023, not a single paper has been retracted or flagged with notes of concern.

## Discussion

Our results reveal that there is a practice of submitting papers to multiple journals, but with different authors. We identified 10 publications of papers also submitted to *NSAP*, but with extensively differing lists of authors. In most cases, text, figures, and tables were nearly identical to the *NSAP* version (Supplementary Figures S1-S10). We did not receive an answer from any of the corresponding authors we contacted. Of the journals we informed about our findings, only two responded to us.

Else (2023) reported on online advertisements how to purchase authorship in scientific papers. One of the authors of this paper (RS) recently received an email, probably from a paper mill, offering to buy his papers to publish them under different authors’ names for $2000 per paper. Alternatively, he could remain the author and publish the work himself, but with credit to other authors provided by the sender of the email, for $1000. The full text of this very revealing email is attached to this article (Supplementary Figure S11). Given this and considering how extensively the authors were exchanged in the studied papers, we suspect a paper mill was involved in the publications we discovered. In the cases where all authors were exchanged, it is virtually impossible to imagine any other explanation than the involvement of a paper mill. In the other cases, authors were still exchanged far too extensively to be explainable given the minor “scientific” changes between the submissions. This impression is reinforced by the fact that the institutions involved in a paper were also often changed arbitrarily between submissions, and in some cases, institutions from completely different countries were added. Of course, that could also be a case of misconduct without a paper mill being involved. Possibly, customers pay for a specific journal. If the publication in the desired journal (*NSAP* in our case) is unsuccessful, some authors may decide not to participate further. Another reason for changing authors may be that it makes it more difficult for publishers to notice simultaneous submissions of a paper to multiple journals. Springer Nature, for example, relies on author names for their paper tracking software.

It is not allowed to submit a paper to more than one journal at the same time. However, in two cases (papers **1** and **2**), we proved that a paper had been submitted simultaneously to *NSAP* and another journal. Submitting a paper to different journals simultaneously increases a paper mill’s chances of a quick publication. This takes advantage of the fact that it is very easy to withdraw a paper from consideration for publication in a journal. An author can withdraw a paper anytime in the peer review stage without giving an explanation. Thus, once a dually submitted paper has been accepted in one journal, it can easily be withdrawn from the second journal without raising suspicion of scientific misconduct. Even simply not responding to emails from the journal is sufficient to ultimately achieve a withdrawal. This is certainly a weak point in current peer review procedures of journals. In the case of the withdrawn publication **4**, the withdrawal may have come too late, so that the paper was public twice for a short time with different lists of authors. We found a higher proportion of potential fakes in the withdrawn papers (1.97%) than in the rejected papers (0.32%) (Fig. 2), supporting the view that withdrawal from a journal in the peer review stage is an important tool of paper mills. In this way, paper mills waste the time of editors and reviewers alike.

There may be legitimate reasons why the authors did not respond to us, but it could also be that the email addresses were not assigned to real persons or that the authors were unable to answer our content-related questions. However, reputable scientists take responsibility for their publications and are reachable for requests relating to their work.

The lack of reaction from most of the journals we contacted may be due to three reasons. First, journals may not be sufficiently aware of the fake paper problem and the sale of authorships. Second, journals may shy away from the tedious and time-consuming work associated with the professional handling of fake paper cases. Third, journals may fear loss of reputation should fake paper cases become public. In any case, paper mills probably use these three possible explanations at the advantage of their business model.

It is important that the affected journals mentioned in this study (Table 1) investigate these cases and, if applicable, retract them or at least post notes of concern. The publications we identified were downloaded up to more than 1000 times and cited up to more than 20 times (Table 7), so they already polluted the scientific record and will continue to do so without retraction notes.

**Table 7:** Scientific impact of the publications

Paper No	No. of citations (Semantic Scholar)	No. of downloads (Journals’ websites)
1	9	–-
2	23	989
3	7	1094
4	0	228
5	16	996
6	18	–-
7	10	478
8	1	–-
9	2	25
10	1	64
Mean:	8.7	553.43

The table is of August 01, 2023

We identified about 0.5% of the investigated papers as potential fakes. This is much less than other estimates of the fake paper share, ranging from 2% (COPE & STM 2022) to 28% (Sabel et al. 2023). However, even at our relatively low rate, 14,500 papers could have been fake in 2020 alone as 2.9 million scientific articles were published that year (White 2021).

Our method can detect purchased authorships if the list of authors of a paper changes substantially between submitted versions. Since there may be legitimate reasons for adding or removing an author between two submitted versions of a paper (Table 4), we looked only for publications with lists of authors differing by a Jaccard coefficient of more than 0.66. We only know of two submitted versions of each paper (the one submitted to *NSAP* and the published one), but there may be further versions, submitted to other journals. This hypothesis is supported by the fact that in case of paper **9**, 5 years passed between the *NSAP* submission and the final publication. Probably, (unsuccessful) attempts were made to publish paper **9** in other journals during this time. We were limited to searching for titles and abstracts in public databases that were similar in content to the titles and abstracts of the unpublished *NSAP* papers. If titles and abstracts had been changed too much between the submissions, we might not have discovered these publications even if the remainder of the paper was identical.

### Recommendations for publishers and scientific journals

There is a large market for fake authorships in scientific papers (Else 2023) and experienced through emails from paper mills (Supplementary Figure S11). It is possible to detect fake papers if the list of authors changes extensively between submissions to different journals. Currently, paper mills take advantage of the fact that journals do not know about submissions to other journals and that withdrawn and rejected papers are not publicly available. The case of the withdrawn paper (**4**), which was probably published by mistake but is now no longer available, shows that paper mills are interested in concealing their previous submissions of a paper because this is an essential part of the business model. Therefore, publishers urgently need to collaborate and build a common database of all submissions they receive, including rejected and withdrawn papers. Resubmissions could be identified more accurately the more parts of a paper were shared in this database with other publishers. At least titles, abstracts, and authors should be shared among different publishers. As a side effect, papers that were illegally submitted to several journals at the same time and thus unnecessarily waste editorial resources could be identified. The International Association of Scientific, Technical and Medical Publishers (STM) is currently testing a tool that is meant to automatically detect whether the same paper has been submitted to multiple journals simultaneously. This tool works by sharing data on submissions among publishers (Else 2022). Perhaps this tool could also be used to search for exchanged lists of authors.

Independently of such collaboration across different publishers, every scientific journal can make immediately its own contribution to the integrity of the scientific record. Specifically, scanning for extensive changes in the lists of authors of withdrawn and rejected papers in the files of any given journal versus finally published paper versions in other journals is a simple approach to detect potential fake papers. The strategy delineated in this paper is suitable to identify at least a part of the fake papers published until now.

### Supplementary Information

Below is the link to the electronic supplementary material.

Supplementary file1 (PDF 401 KB)

Supplementary file2 (PDF 1280 KB)

Supplementary file3 (PDF 966 KB)

Supplementary file4 (PDF 694 KB)

Supplementary file5 (PDF 601 KB)

Supplementary file6 (PDF 969 KB)

Supplementary file7 (PDF 5820 KB)

Supplementary file8 (PDF 607 KB)

Supplementary file9 (PDF 1152 KB)

Supplementary file10 (PDF 947 KB)

Supplementary file11 (PDF 103 KB)

## Data Availability

All source data of this study are available upon reasonable request.

## Abbreviations

*NSAP*: Naunyn-Schmiedeberg’s Archives of Pharmacology
LSTM: Long Short-Term Memory
