# Supplementary material for: Fake paper identification in the pool of withdrawn and rejected manuscripts submitted to Naunyn–Schmiedeberg’s Archives of Pharmacology
Source: Naunyn Schmiedebergs Arch Pharmacol. 2023 Oct 5;397(4):2171–81. doi: 10.1007/s00210-023-02741-w (PMC10933159; doi:10.1007/s00210-023-02741-w)
Supplement: Supplementary file 1 — Supplementary file1 (PDF 401 KB) [file 210_2023_2741_MOESM1_ESM.pdf]

Figure S1

Color coding:

Yellow highlighted text	The text is identical in the NSAP version and the published version of this paper.
Red highlighted text	There are differences in the text between the NSAP version and the published version of this paper (different content or different wording).
Yellow bordered figure	This figure is identical in both versions of this paper.

Naunyn-Schmiedeberg's Archives of Pharmacology

Aldosterone receptor antagonists-mediated cognitive improvement in a mouse model of Alzheimer's type: A key role of BDNF-H2S-Nrf2 signaling

--Manuscript Draft--

Manuscript Number:	NSAP-D-20-00138
Full Title:	Aldosterone receptor antagonists-mediated cognitive improvement in a mouse model of Alzheimer's type: A key role of BDNF-H2S-Nrf2 signaling
Article Type:	Original Article
Corresponding Author:	Jun Wang<br>The first people hospital of Taizhou<br>CHINA
Corresponding Author Secondary Information:	
Corresponding Author's Institution:	The first people hospital of Taizhou
Corresponding Author's Secondary Institution:	
First Author:	Ning Li
First Author Secondary Information:	
Order of Authors:	Ning Li
	Yan Wang
	Wensheng Li
	Haiyan Li
	Liu Yang
	Jun Wang
Order of Authors Secondary Information:	
Funding Information:	
Abstract:	The present study explored the role and mechanisms of aldosterone receptor blockers in β-amyloid (Aβ)-induced cognitive impairment. A single intracerebroventricular injection of Aβ 1-42 was given to mice and after 14 days of injection, memory was evaluated using Morris Water maze test. Spironolactone (25 and 50 mg/kg) and eplerenone (50 and 100 mg/kg) were adminsiretred for 2 days before and for 14 days after Aβ injection. Aldosterone receptor blockers attenuated Aβ-induced cognitive impairment assessed in terms of decrease in day 4 escape latency time (ELT) in comparison to day 1 ELT (suggesting an increase in learning) along with an increase in time spent in target quadrant on day 5 (suggesting the retrieval of learned things). These drugs also increased the expression of BDNF, H_2S, Nrf2, reduced glutathione and decreased β-amyloid and TNF-α in frontal cortex and hippocampus. Co-administration of ANA-12, BDNF receptor antagonist (0.25 and 0.5 mg/kg) abolished cognitive improving functions of aldosterone blockers; attenuated H_2S, Nrf2, reduced glutathione; decreased β-amyloid and TNF-α. It is concluded that spironolactone and eplerenone attenuate cognitive decline of Alzheimer's type, possibly through upregulation of BDNF levels in the frontal cortex and hippocampus, which may increase H_2S, decrease Aβ, activate Nrf2-dependent antioxidant system and decrease neuroinflammation.
Suggested Reviewers:	Xiaohong Lv<br>xiaohong.lv@yahoo.com
	Zonggui Wang<br>zg.wang@yahoo.com
	Kailiang Cheng

Powered by Editorial Manager® and ProduXion Manager® from Aries Systems Corporation

	chen_kailiang18@yahoo.com

Powered by Editorial Manager® and ProduXion Manager® from Aries Systems Corporation

Manuscript
Click here to access/download;Manuscript;manuscript.docx
Click here to view linked References

Aldosterone receptor antagonists-mediated cognitive improvement in a mouse model of Alzheimer's type: A key role of BDNF-H_2S-Nrf2 signaling

Ning Li[1#], Yan Wang[2#], Wensheng Li[3], Haiyan Li[4], Liu Yang[5], Jun Wang[6*]

Running Title: Aldosterone antagonists and cognitive improvement

[1]Department of Neurology, Jinan Third People's Hospital, Shandong, Jinan, 250132, China.

[2]Department of Neurology, People's Hospital of Danyang, Jiangsu Danyang, 212300, China.

[3]Department of Neurology, No. 1 Hospital, Handan City, Hebei Province, Hebei, Handan, 056002, China.

[4]Department of Neurology, Beijing Shijitan Hospital, Capital Medical University, Beijing, 100038, China

[5]Department of Neurology, Emergency Medical Center of Chongqing, Chongqing, 400014, China.

[6]Department of Neurology, The First People's Hospital of Taizhou , Zhejiang,Taizhou, 318020, China.

# These Authors contributed equally.

Ning Li: ln1021ln@sina.com

Yan Wang: 13952919671w@sina.com

Wensheng Li: 17891319212m0@sina.com

Haiyan Li: lihaiyandb@sina.com

Liu Yang: emilybruce@sina.cn

Jun Wang: zhanghanc2006@sina.com

[*]**Corresponding Author:** Jun Wang, Department of Neurology, The First People's Hospital of Taizhou , Zhejiang,Taizhou, 318020, China. Email: zhanghanc2006@sina.com

Abstract

The present study explored the role and mechanisms of aldosterone receptor blockers in β-amyloid (Aβ)-induced cognitive impairment. A single intracerebroventricular injection of $A\beta_{1-42}$ was given to mice and after 14 days of injection, memory was evaluated using Morris Water maze test. Spironolactone (25 and 50 mg/kg) and eplerenone (50 and 100 mg/kg) were adminsiretred for 2 days before and for 14 days after Aβ injection. Aldosterone receptor blockers attenuated Aβ-induced cognitive impairment assessed in terms of decrease in day 4 escape latency time (ELT) in comparison to day 1 ELT (suggesting an increase in learning) along with an increase in time spent in target quadrant on day 5 (suggesting the retrieval of learned things). These drugs also increased the expression of BDNF, H_2S, Nrf2, reduced glutathione and decreased β-amyloid and TNF-α in frontal cortex and hippocampus. Co-administration of ANA-12, BDNF receptor antagonist (0.25 and 0.5 mg/kg) abolished cognitive improving functions of aldosterone blockers; attenuated H_2S, Nrf2, reduced glutathione; decreased β-amyloid and TNF-α. It is concluded that spironolactone and eplerenone attenuate cognitive decline of Alzheimer's type, possibly through upregulation of BDNF levels in the frontal cortex and hippocampus, which may increase H_2S, decrease Aβ, activate Nrf2-dependent antioxidant system and decrease neuroinflammation.

Key Words: beta-amyloid, Morris Water maze, Hippocampus, frontal cortex, Alzheimer's disease

Introduction

Alzheimer's disease is the most common neurodegenerative cause of dementia in all over the world, which is responsible for not only increasing the morbidity and mortality but also increasing the economic burden on the health care system (Lane et al., 2018). However, the present drug therapy is not suitable enough to give long-lasting benefits to patients. Accordingly, there is a need to identify new drugs for its effective management. Spironolactone and eplerenone are aldosterone receptor blockers and have been employed in the clinics as potassium-sparing diuretics. In clinics, these are predominantly used for the management of congestive heart failure and these have been reported to reduce cardiac remodeling and mortality rate in these patients (Yang et al., 2019). Studies have shown their potential in modifying the pathogenesis of diseases including diabetic cardiomyopathy (Liu et al., 2018), diabetic nephropathy (Zuo and Xu, 2019), treatment-resistant hypertension (Imprialos et al., 2018), chorioretinopathy (Chatziralli et al., 2018), inflammasome formation ((Wada et al., 2017), vascular dysfunction (Silva et al., 2015) and muscular dystrophy (Lowe et al., 2018). Furthermore, studies have shown the usefulness of aldosterone receptor blockers in improving memory functions (Zhang et al., 2019; Rotenstein et al., 2015). However, their role and mechanisms in dementia of Alzheimer's type are not explored.

Brain-derived neurotrophic factor (BDNF) belongs to the family of the neurotrophin family and its role in improving learning and memory is well documented. Moreover, there have been studies showing the key role in the pathogenesis of Alzheimer's disease (Tanila, 2017; Song et al., 2015). H_2S is a gaseous neurotransmitter and its beneficial effects in memory formation including in Alzheimer's disease is also very well documented (Wei et al., 2014). Nuclear factor erythroid 2-related factor (Nrf2) is a transitional factor and its importance lies in

increasing the cellular levels of antioxidants. A decrease in the levels of Nrf2 along with antioxidants has been shown to be involved in the pathogenesis of Alzheimer's disease (Youssef et al., 2018).

Based on these, the present study was designed to investigate the learning and memory improving functions of spironolactone and eplerenone in the Aβ-induced model of dementia of Alzheimer's type in mice. Furthermore, the study also explored the possible role of BDNF, H_2S and Nrf2 signaling in aldosterone-mediated cognition improving functions in a mouse model of Alzheimer's type.

Material and Methods

Animals and Drugs

Swiss albino mice of weight ranging from 20 to 24 g were employed for the study. Any animal outside this range was excluded. The experiments were approved by the Ethic committee of Jinan Third People's Hospital with the ethic number: 2019A2568B25. The animals were habituated for 1 week in the laboratory conditions before the actual start of experiments. They were kept in the laboratory with 12 hours light/dark, 40 % humidity and 24° to 25° C temperature. ANA-12 was employed as a specific BDNF receptor antagonist (Tocris, USA). The doses of ANA-12 (Ren et al., 2013), spironolactone (Silva et al., 2015) and eplerenone (Raz-Pasteur et al., 2012) were selected used as per literature reports.

Induction of Cognitive Deficits of Alzheimer's Type

Amyloid-β (Aβ) is considered as one of the major pathological mediators in the pathogenesis of Alzheimer's disease (AD) and abnormal accumulation of Aβ impair synaptic function and induces cognitive deficits. The development of cognitive deficits of Alzheimer's type in animals is very successfully done by an intracerebroventricular injection (ICV) of $A\beta_{1-42}$,

which delivers the peptide in the ventricles of the brain (Koh et al., 2017; Ahmad et al., 2017). Accordingly, a single ICV injection of $A\beta_{1-42}$ (100 μM/10μl) was employed to induce symptoms of Alzheimer's disease in mice as per previously reported studies (Kim et al., 2016).

Morris Water Maze Test to Evaluate Learning and Memory

The Morris Water Maze test is very frequently employed to assess hippocampal-dependent spatial learning. In this five day test, animals were trained to locate a hidden platform in opaque water located in one of the quadrants (total four quadrants) for the first four days. In a 120 s trial, the time taken by an animal to locate the platform was denoted as escape latency time (ELT). In normal animals, there is a decrease in ELT with each trial and it indicates the normal learning ability. On the fifth day, the retrieval of memory was assessed by noting the time spent in the target quadrant (in which hidden platform was located for the first four days). The maximum time spent in the target quadrant indicates the normal memory (retrieval of learned things) of animals. Accordingly in the present study, ELT (an index of learning) and time spent in the target quadrant (an index of memory) were evaluated in the Morris Water Maze test as per previously described methods (Bromley-Brits et al., 2011; Vorhees and Williams, 2006).

Biochemical Estimations

After the completion of cognitive testing on the Morris Water maze test i.e. on the last day of testing, mice were sacrificed. The frontal cortex and hippocampus regions were isolated and homogenized in phosphate buffer solution. The supernatants of the frontal cortex and hippocampus were employed for the quantification of different biochemical parameters. The quantification of BDNF, β-amyloid, TNF-α, Nrf2 was done using commercially available ELISA kits. The procedure for their quantification was followed as per instructions mentioned in ELISA kits. The quantification of reduced glutathione was done by a colorimetric method (Cappiello et

al., 2013). The levels of H_2S were measured using reversed-phase high-performance liquid chromatography (Shen et al., 2015). The expression of all these biochemical parameters was done with respect to protein content, which was quantified using the Folin Lowey method (Okutucu et al., 2007).

Experimental protocol

The nine groups were employed and each group comprised eight animals.

i. Sham control: ICV injection of the vehicle of $A\beta_{1\text{-}42}$ (phosphate buffer solution; 10μl) was made in mice. These kept for 14 days and thereafter, cognitive testing was performed for five days using the Morris water maze test. After the last day of testing, animals were sacrificed using cervical dislocation and the brain was isolated (frontal cortex and hippocampus) for various biochemical parameters.

ii. AD control: ICV injection of $A\beta_{1\text{-}42}$ (dissolved in phosphate buffer solution; 10μl) was made in mice and kept for 14 days. The rest of the protocol was the same as per the sham control group.

iii. Spironolactone (25 mg/kg) in AD control: Spironolactone (25 mg/kg *p.o.*) was administered three days before ICV injection of $A\beta_{1\text{-}42}$ and continued for 14 days after injection. After 14 days, cognitive testing was performed as described in the sham control group. The biochemical tests were also performed as described in the sham control group.

iv. Spironolactone (50 mg/kg) in AD control: Spironolactone (50 mg/kg *p.o.*) was administered three days before ICV injection of $A\beta_{1\text{-}42}$ and continued for 14 days after injection. The rest of the protocol was the same as described in the sham group.

v. Eplerenone (50 mg/kg) in AD control: Eplerenone (50 mg/kg *p.o*) was administered three days before the ICV injection of $A\beta_{1-42}$ and continued for 14 days after injection. The rest of the protocol was the same as described in the sham group.

vi. Eplerenone (100 mg/kg) in AD control: Eplerenone (100 mg/kg *p.o.*) was administered three days before the ICV injection of $A\beta_{1-42}$ and continued for 14 days after injection. The rest of the protocol was the same as described in the sham group.

vii. Trk antagonist, ANA-12 (0.25 mg/kg) and Spironolactone (50 mg/kg) in AD control: ANA-12 (0.25 mg/kg *p.o.*) was co-administered with spironolactone (50 mg/kg *p.o.*) for three days before ICV injection and for 14 days after ICV injection. The rest of the protocol was the same as described in the sham control group.

viii. Trk antagonist, ANA-12 (0.50 mg/kg) and Spironolactone (50 mg/kg) in AD control: ANA-12 (0.50 mg/kg *p.o.*) was co-administered with spironolactone (50 mg/kg *p.o.*) for three days before ICV injection and for 14 days after ICV injection. The rest of the protocol was the same as described in the sham control group.

ix. Trk antagonist, ANA-12 (0.50 mg/kg) and Eplerenone (100 mg/kg) in AD control: ANA-12 (0.50 mg/kg *p.o.*) was co-administered with eplerenone (100 mg/kg *p.o.*) for three days before ICV injection and for 14 days after ICV injection. The rest of the protocol was the same as described in the sham control group.

Statistical Analysis

The results of this study were represented as mean ± SEM. The data obtained were compared using Two way ANOVA followed by Tukey's multiple comparison test. The $p < 0.05$ was considered to be statistically significant.

Results

Spironolactone and Eplerenone attenuate Aβ_{1-42}-induced cognitive decline

A single ICV injection of Aβ_{1-42} significantly impaired cognitive functions in mice as assessed on the Morris Water Maze test after 14 days of Aβ injection. In sham control animals, there was a significant decrease in day 4 ELT in comparison to day 1 ELT suggesting the normal ability of leaning. In contrast, there was no alteration in ELT on day 4 in comparison to day 1 ELT suggesting that these animals failed to acquire new information **(Table 1)**. Furthermore, there was a significant decrease in TSTQ on day 5 in Aβ-injected mice in comparison to TSTQ on day 5 in sham control mice, suggesting a significant impairment in memory in Aβ-injected mice **(Figure 1)**. Treatment with aldosterone antagonists i.e. spironolactone (25 and 50 mg/kg) and eplerenone (50 and 100 mg/kg) for 3 days before Aβ injection and 14 days after Aβ injection significantly ameliorated the cognitive decline in Aβ-injected mice in a dose-dependent manner. There was a significant decrease in the day 4 ELT in comparison to day 1 ELT **(Table 1)** along with a rise in TSTQ on day 5 in spironolactone and eplerenone-treated mice on Morris Water maze test **(Figure 1)**.

Spironolactone and eplerenone produce cognitive improvement by increasing the levels of BDNF in the brain

In spironolactone and eplerenone-treated mice, there was a significant increase in the expression of BDNF in the frontal cortex and hippocampus regions of Aβ-injected mice. Indeed, injection of Aβ led to a significant decline in the BDNF levels in these brain regions in comparison to sham control mice and these levels were restored by aldosterone antagonists in Aβ-injected mice **(Figure 2)**. It suggests the possible role of a decrease in the BDNF levels in attenuating cognitive function in Aβ-injected mice and an increase in BDNF levels contributing

to improve the cognitive functions in spironolactone and eplerenone-treated mice. To further explore the role of BDNF in spironolactone and eplerenone-mediated improvement in cognitive functions, BDNF antagonist, Trk antagonist (ANA-12) was co-administered with spironolactone and eplerenone. Co-administration of ANA-12 (0.25 and 0.5 mg/kg) significantly led to attenuation of spironolactone and eplerenone-mediated decrease in day 4 ELT and day 5 TSTQ in Morris Water maze test **(Table 1 and Figure 1)**. However, ANA-12 (0.25 and 0.5 mg/kg) did not modulate BDNF levels in spironolactone and eplerenone-treated mice

Spironolactone and eplerenone increase H_2S, decrease neuroinflammation, β-amyloid and oxidative stress in the brain

A single ICV injection of Aβ produced significant alterations in biochemical milieu in the frontal cortex and hippocampus region of brain, which included increase in TNF-α (a marker of neuroinflammation) **(Figure 3);** decrease in H_2S levels **(Figure 4);** increase in β-amyloid **(Figure 5);** decrease in antioxidants including reduced glutathione **(Figure 6)** and Nrf2 **(Figure 7)**. Treatment with spironolactone and eplerenone attenuated Aβ-induced deleterious effects on various biochemical parameters. Indeed, treatment with aldosterone antagonists decreased the levels of TNF-α and β-amyloid; and increased the levels of H_2S, reduced glutathione and Nrf2. Co-administration of ANA-12 attenuated spironolactone and eplerenone-mediated restoration of TNF-α, β-amyloid, H_2S, reduced glutathione and Nrf2 in frontal cortex and hippocampus.

Discussion

In the present study, a single ICV injection of $Aβ_{1-42}$ significantly impaired learning and memory in mice as assessed in the Morris water maze test. In $Aβ_{1-42}$ injected mice, day 4 ELT was not significantly different from day 1 ELT suggesting that these mice failed to learn on four

day's trials. Moreover, there was no increase in day 5 TSTQ (time spent in the target quadrant) suggesting the impairment in memory. $A\beta_{1-42}$ is very frequently used to induce dementia of Alzheimer's type in mice (Xu et al., 2016; Yun et al., 2018). The impairment in cognitive functions observed in this study is in line with the previous studies showing impairment in learning and memory on the ICV injection of $A\beta_{1-42}$ (Min et al., 2017). Morris Water Maze test is the most widely employed test to assess learning and memory in mice (Kilic et al., 2019) and it specifically assesses hippocampal-dependent spatial memory (Kathner-Schaffert et al., 2019).

In the present study, treatment with aldosterone antagonists i.e. spironolactone and eplerenone led to an improvement in Aβ-induced decrease in learning and memory. Treatment with these agents was associated with a significant decrease in day 4 ELT (indicating an increase in learning) and an increase in day 5 TSTQ (retrieval of learned things i.e. memory) in Aβ-injected mice in Morris Water maze test. Aldosterone antagonists are potassium-sparing diuretics and these are clinically employed in the management of congestive heart failure (Yang et al., 2019). However, studies have shown their expanding spectrum in a number of diseases including diabetic nephropathy (Zuo and Xu, 2019), treatment-resistant hypertension (Imprialos et al., 2018), chorioretinopathy (Chatziralli et al., 2018) etc. There have been studies showing that activation of aldosterone receptors may be associated with impairment in memory (Zhang et al., 2019) and blockade of these receptors may be beneficial in memory (Rotenstein et al., 2015). However, it is the first study showing the memory improvement actions of spironolactone and eplerenone in Aβ-induced cognitive impairment of Alzheimer's type.

In the present study, spironolactone and eplerenone-mediated improvement in cognitive functions were associated with the restoration of BDNF levels in the frontal cortex and hippocampus region in Aβ-injected mice. It suggests that restoration of BDNF levels may

possibly contribute to mediating beneficial effects of spironolactone and eplerenone. The possible contribution of BDNF in cognitive improvement was further observed in this study as co-administration of BDNF receptor antagonist (Trk antagonist, ANA-12) significantly led to attenuation of spironolactone and eplerenone-mediated cognition-restorative actions. To best of our knowledge, it is the first study showing that aldosterone antagonists may improve cognition by enhancing the levels of BDNF in the frontal cortex and hippocampus.

In this study, aldosterone antagonists also restored the levels of H_2S in the frontal cortex and hippocampus regions in Aβ-injected mice suggesting the possible role of H_2S in cognition improving actions. H_2S is a gaseous neurotransmitter and its importance in memory function has been emphasized (Mohseni et al., 2019). Furthermore, the administration of ANA-12 (BDNF antagonist) ameliorated the aldosterone-antagonists-mediated increase in the levels of H_2S suggesting the possible interrelationship between BDNF and H_2S in aldosterone-mediated beneficial actions. The previous studies have shown that exogenous administration of H2S may increase the expression of BDNF to improve cognitive functions (Mohseni et al., 2019; Li et al., 2019). However, it is the first report suggesting that the blockade of BDNF receptors attenuates the expression of H_2S and attenuates the improvement in cognitive functions. Accordingly, it may be tentatively proposed that the blockade of aldosterone receptors may increase the levels of BDNF, which may subsequently increase the levels of H_2S to improve the learning and memory functions in an animal model of Alzheimer's type.

Spironolactone and eplerenone also decreased the levels of Aβ in the frontal cortex and hippocampus, and this action was abolished in the presence of BDNF receptor antagonist. It possibly suggests the Aβ clearing actions of aldosterone receptor blockers, which is possibly mediated through the upregulation of BDNF. Furthermore, treatment with aldosterone

antagonists also attenuated Aβ injection-induced increase in TNF-α levels in the frontal cortex and hippocampus suggesting the decrease in neuroinflammation, which is a very important contributor in the cognitive decline of Alzheimer's type. Aldosterone antagonists also restored the expression of Nrf2, which is a key transcriptional factor involved in increasing the levels of antioxidants. Moreover, there was also an increase in the levels of reduced glutathione (an antioxidant) in frontal cortex and hippocampus regions in response to spironolactone and eplerenone treatment. Aldosterone-blockers mediated decrease in neuroinflammation and an increase in antioxidant parameters was abolished in ANA-12 treated mice. Based on these, it may be proposed that spironolactone and eplerenone-mediated increase in BDNF levels may eventually increase the levels of H_2S, increase the clearance of Aβ, decrease neuroinflammation and increase the intrinsic protective antioxidant activity, which may be involved in preventing Aβ-mediated decline in cognitive functions.

Conclusion

Spironolactone and eplerenone have the potential to attenuate the cognitive decline of Alzheimer's type, which may be possibly mediated through the upregulation of BDNF levels in the frontal cortex and hippocampus. An increase in BDNF may increase the levels of H2S, prevent deposition of Aβ, activate the Nrf2-dependent endogenous antioxidant system and decrease neuroinflammation to prevent Aβ-mediated decline in cognitive functions.

Author's Contribution Statement:

NL and YW conducted experiments and collected data. WL and HLwrote the mnaucript. LY helped in data analysis. JW coceived the project and edited the manuscript.

Compliance to Ethical Standards

All experiments were condcuted in an ethical manner after approval from ethical committe of Jinan Third People's Hospital with the ethic number: 2019A2568B25.

Conflict statement

The authors declare no conflicts of interests.

References

Ahmad, A., Ali, T., Park, H.Y., Badshah, H., Rehman, S.U., Kim, M.O., 2017. Neuroprotective Effect of Fisetin Against Amyloid-Beta-Induced Cognitive/Synaptic Dysfunction, Neuroinflammation, and Neurodegeneration in Adult Mice. Mol. Neurobiol. 54, 2269–2285. https://doi.org/10.1007/s12035-016-9795-4

Bromley-Brits, K., Deng, Y., Song, W., 2011. Morris water maze test for learning and memory deficits in Alzheimer's disease model mice. J. Vis. Exp. JoVE. https://doi.org/10.3791/2920

Cappiello, M., Peroni, E., Lepore, A., Moschini, R., Del Corso, A., Balestri, F., Mura, U., 2013. Rapid colorimetric determination of reduced and oxidized glutathione using an end point coupled enzymatic assay. Anal. Bioanal. Chem. 405, 1779–1785. https://doi.org/10.1007/s00216-012-6577-3

Chatziralli, I., Vlachodimitropoulou, A., Daoula, C., Vrettou, C., Galani, E., Theodossiadis, G., Theodossiadis, P., 2018. Eplerenone in the treatment of central serous chorioretinopathy: a review of the literature. Int. J. Retina Vitr. 4, 33. https://doi.org/10.1186/s40942-018-0137-8

Imprialos, K.P., Bouloukou, S., Kerpiniotis, G., Katsimardou, A., Patoulias, D., Bakogiannis, C., Faselis, C., 2018. Mineralocorticoid Receptor Antagonists in Essential and Resistant Hypertension. Curr. Pharm. Des. 24, 5500–5507. https://doi.org/10.2174/1381612825666190306163310

Kathner-Schaffert, C., Karapetow, L., Günther, M., Rudolph, M., Dahab, M., Baum, E., Lehmann, T., Witte, O.W., Redecker, C., Schmeer, C.W., Keiner, S., 2019. Early Stroke Induces Long-Term Impairment of Adult Neurogenesis Accompanied by Hippocampal-Mediated Cognitive Decline. Cells 8. https://doi.org/10.3390/cells8121654

Kilic, F.S., Kaygisiz, B., Aydin, S., Yildirim, E., Oner, S., Erol, K., 2019. The effects and mechanisms of the action of galangin on spatial memory in rats. Bratisl. Lek. Listy 120, 881–886. https://doi.org/10.4149/BLL_2019_148

Kim, H.Y., Lee, D.K., Chung, B.-R., Kim, H.V., Kim, Y., 2016. Intracerebroventricular Injection of Amyloid-β Peptides in Normal Mice to Acutely Induce Alzheimer-like Cognitive Deficits. J. Vis. Exp. JoVE. https://doi.org/10.3791/53308

Koh, E.-J., Kim, K.-J., Song, J.-H., Choi, J., Lee, H.Y., Kang, D.-H., Heo, H.J., Lee, B.-Y., 2017. Spirulina maxima Extract Ameliorates Learning and Memory Impairments via Inhibiting GSK-3β Phosphorylation Induced by Intracerebroventricular Injection of Amyloid-β 1-42 in Mice. Int. J. Mol. Sci. 18. https://doi.org/10.3390/ijms18112401

Lane, C.A., Hardy, J., Schott, J.M., 2018. Alzheimer's disease. Eur. J. Neurol. 25, 59–70. https://doi.org/10.1111/ene.13439

Li, X., Zhuang, Y.-Y., Wu, L., Xie, M., Gu, H.-F., Wang, B., Tang, X.-Q., 2019. Hydrogen Sulfide Ameliorates Cognitive Dysfunction in Formaldehyde-Exposed Rats: Involvement

in the Upregulation of Brain-Derived Neurotrophic Factor. Neuropsychobiology 1–12. https://doi.org/10.1159/000501294

Liu, W., Gong, W., He, M., Liu, Y., Yang, Y., Wang, M., Wu, M., Guo, S., Yu, Y., Wang, X., Sun, F., Li, Y., Zhou, L., Qin, S., Zhang, Z., 2018. Spironolactone Protects against Diabetic Cardiomyopathy in Streptozotocin-Induced Diabetic Rats. J. Diabetes Res. 2018. https://doi.org/10.1155/2018/9232065

Lowe, J., Kadakia, F.K., Zins, J.G., Haupt, M., Peczkowski, K.K., Rastogi, N., Floyd, K.T., Gomez-Sanchez, E.P., Gomez-Sanchez, C.E., Elnakish, M.T., Rafael-Fortney, J.A., Janssen, P.M.L., 2018. Mineralocorticoid Receptor Antagonists in Muscular Dystrophy Mice During Aging and Exercise. J. Neuromuscul. Dis. 5, 295–306. https://doi.org/10.3233/JND-180323

Min, L.-J., Kobayashi, Y., Mogi, M., Tsukuda, K., Yamada, A., Yamauchi, K., Abe, F., Iwanami, J., Xiao, J.-Z., Horiuchi, M., 2017. Administration of bovine casein-derived peptide prevents cognitive decline in Alzheimer disease model mice. PloS One 12, e0171515. https://doi.org/10.1371/journal.pone.0171515

Mohseni, F., Bagheri, F., Rafaiee, R., Norozi, P., Khaksari, M., 2019. Hydrogen sulfide improves spatial memory impairment via increases of BDNF expression and hippocampal neurogenesis following early postnatal alcohol exposure. Physiol. Behav. 112784. https://doi.org/10.1016/j.physbeh.2019.112784

Okutucu, B., Dinçer, A., Habib, O., Zihnioglu, F., 2007. Comparison of five methods for determination of total plasma protein concentration. J. Biochem. Biophys. Methods 70, 709–711. https://doi.org/10.1016/j.jbbm.2007.05.009

Raz-Pasteur, A., Gamliel-Lazarovich, A., Coleman, R., Keidar, S., 2012. Eplerenone reduced lesion size in early but not advanced atherosclerosis in apolipoprotein E-deficient mice. J. Cardiovasc. Pharmacol. 60, 508–512. https://doi.org/10.1097/FJC.0b013e31826f5535

Ren, Q., Zhang, J.-C., Fujita, Y., Ma, M., Wu, J., Hashimoto, K., 2013. Effects of TrkB agonist 7,8-dihydroxyflavone on sensory gating deficits in mice after administration of methamphetamine. Pharmacol. Biochem. Behav. 106, 124–127. https://doi.org/10.1016/j.pbb.2013.03.016

Rotenstein, L.S., Sheridan, M., Garg, R., Adler, G.K., 2015. Effect of mineralocorticoid receptor blockade on hippocampal-dependent memory in adults with obesity. Obes. Silver Spring Md 23, 1136–1142. https://doi.org/10.1002/oby.21104

Shen, X., Kolluru, G.K., Yuan, S., Kevil, C.G., 2015. Measurement of H2S in vivo and in vitro by the monobromobimane method. Methods Enzymol. 554, 31–45. https://doi.org/10.1016/bs.mie.2014.11.039

Silva, M.A.B., Bruder-Nascimento, T., Cau, S.B.A., Lopes, R.A.M., Mestriner, F.L.A.C., Fais, R.S., Touyz, R.M., Tostes, R.C., 2015. Spironolactone treatment attenuates vascular dysfunction in type 2 diabetic mice by decreasing oxidative stress and restoring NO/GC signaling. Front. Physiol. 6, 269. https://doi.org/10.3389/fphys.2015.00269

Song, J.-H., Yu, J.-T., Tan, L., 2015. Brain-Derived Neurotrophic Factor in Alzheimer's Disease: Risk, Mechanisms, and Therapy. Mol. Neurobiol. 52, 1477–1493. https://doi.org/10.1007/s12035-014-8958-4

Tanila, H., 2017. The role of BDNF in Alzheimer's disease. Neurobiol. Dis. 97, 114–118. https://doi.org/10.1016/j.nbd.2016.05.008

Vorhees, C.V., Williams, M.T., 2006. Morris water maze: procedures for assessing spatial and related forms of learning and memory. Nat. Protoc. 1, 848–858. https://doi.org/10.1038/nprot.2006.116

Wada, T., Ishikawa, A., Watanabe, E., Nakamura, Y., Aruga, Y., Hasegawa, H., Onogi, Y., Honda, H., Nagai, Y., Takatsu, K., Ishii, Y., Sasahara, M., Koya, D., Tsuneki, H., Sasaoka, T., 2017. Eplerenone prevented obesity-induced inflammasome activation and glucose intolerance. J. Endocrinol. 235, 179–191. https://doi.org/10.1530/JOE-17-0351

Wei, H.-J., Li, X., Tang, X.-Q., 2014. Therapeutic benefits of H_2S in Alzheimer's disease. J. Clin. Neurosci. Off. J. Neurosurg. Soc. Australas. 21, 1665–1669. https://doi.org/10.1016/j.jocn.2014.01.006

Xu, M., Dong, Y., Wan, S., Yan, T., Cao, J., Wu, L., Bi, K., Jia, Y., 2016. Schisantherin B ameliorates Aβ1-42-induced cognitive decline via restoration of GLT-1 in a mouse model of Alzheimer's disease. Physiol. Behav. 167, 265–273. https://doi.org/10.1016/j.physbeh.2016.09.018

Yang, P., Shen, W., Chen, X., Zhu, D., Xu, X., Wu, T., Xu, G., Wu, Q., 2019. Comparative efficacy and safety of mineralocorticoid receptor antagonists in heart failure: a network meta-analysis of randomized controlled trials. Heart Fail. Rev. 24, 637–646. https://doi.org/10.1007/s10741-019-09790-5

Youssef, P., Chami, B., Lim, J., Middleton, T., Sutherland, G.T., Witting, P.K., 2018. Evidence supporting oxidative stress in a moderately affected area of the brain in Alzheimer's disease. Sci. Rep. 8, 11553. https://doi.org/10.1038/s41598-018-29770-3

Yun, J., Yeo, I.J., Hwang, C.J., Choi, D.-Y., Im, H.-S., Kim, J.Y., Choi, W.R., Jung, M.H., Han, S.-B., Hong, J.T., 2018. Estrogen deficiency exacerbates Aβ-induced memory

impairment through enhancement of neuroinflammation, amyloidogenesis and NF-κB activation in ovariectomized mice. Brain. Behav. Immun. 73, 282–293. https://doi.org/10.1016/j.bbi.2018.05.013

Zhang, B., Zhu, X., Wang, L., Hou, Z., Hao, S., Yang, M., Gao, F., Liu, B., 2019. Inadequate Expression and Activation of Mineralocorticoid Receptor Aggravates Spatial Memory Impairment after Traumatic Brain Injury. Neuroscience 424, 1–11. https://doi.org/10.1016/j.neuroscience.2019.10.026

Zuo, C., Xu, G., 2019. Efficacy and safety of mineralocorticoid receptor antagonists with ACEI/ARB treatment for diabetic nephropathy: A meta-analysis. Int. J. Clin. Pract. e13413. https://doi.org/10.1111/ijcp.13413

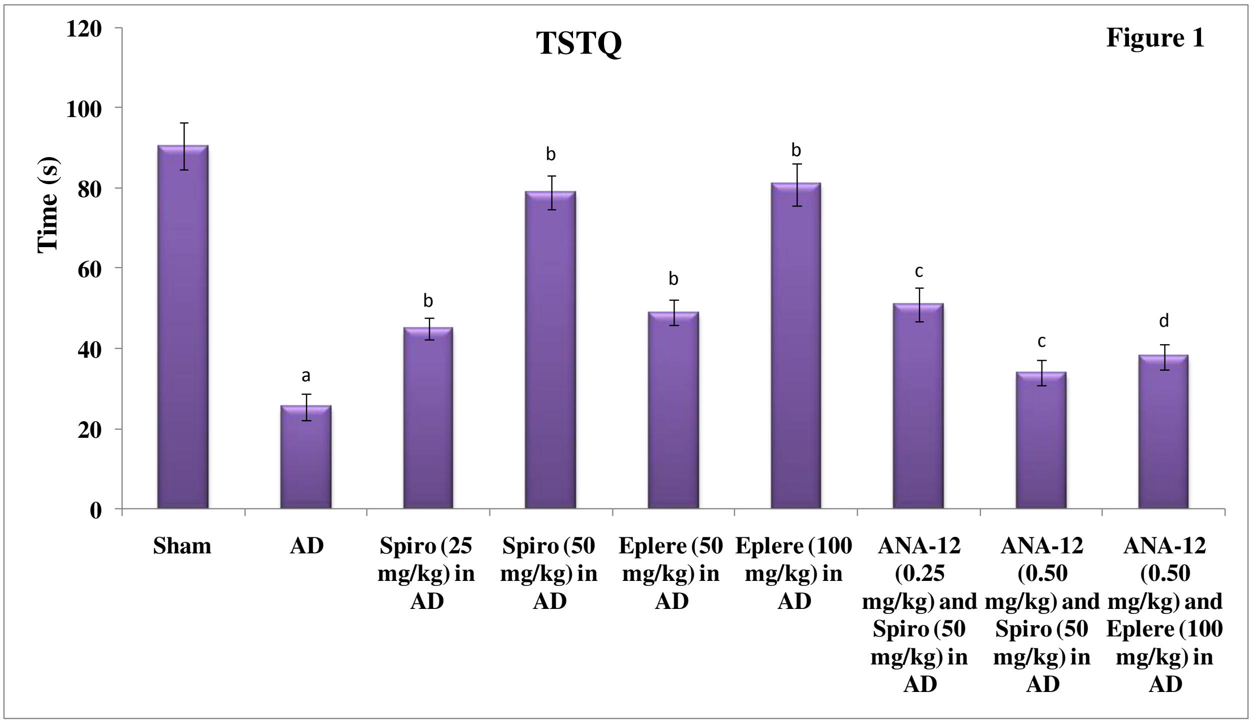

Figure 1: Effect of different interventions on time spent in target quadrant (TSTQ) measured on 5th day on Morris Water Maze test. a= $p < 0.05$ *vs*. sham; b = $p < 0.05$ *vs*. AD; c = $p < 0.05$ *vs*. Spiro (50 mg/kg) in AD; d = $p < 0.05$ *vs*. Eplere (100 mg/kg) in AD. Spiro: Spironolactone; Eplere: Eplerenone.

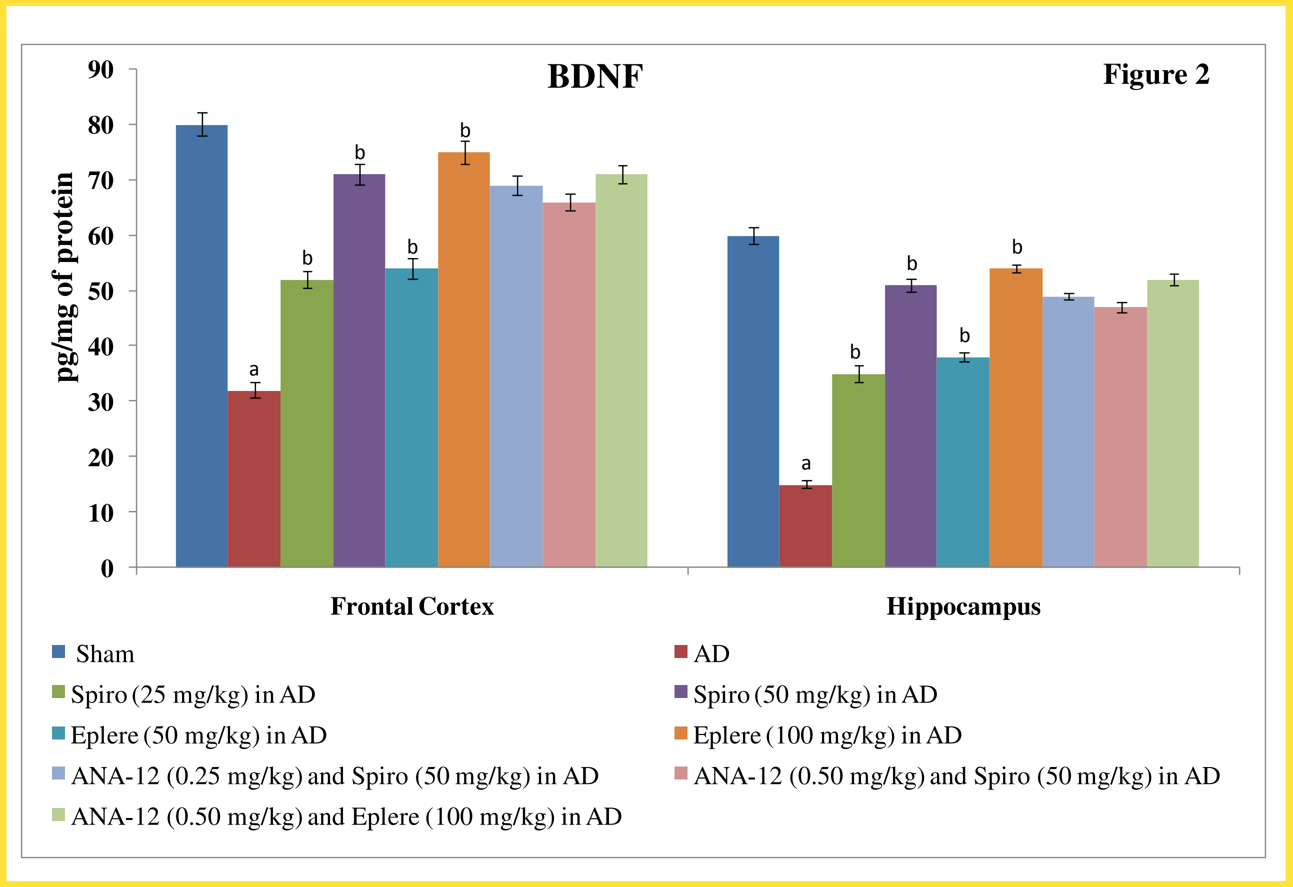

Figure 2: Effect of different interventions on BDNF levels in the brain homogenate. a= $p< 0.05$ *vs*. sham; b = $p< 0.05$ *vs*. AD. Spiro: Spironolactone; Eplere: Eplerenone.

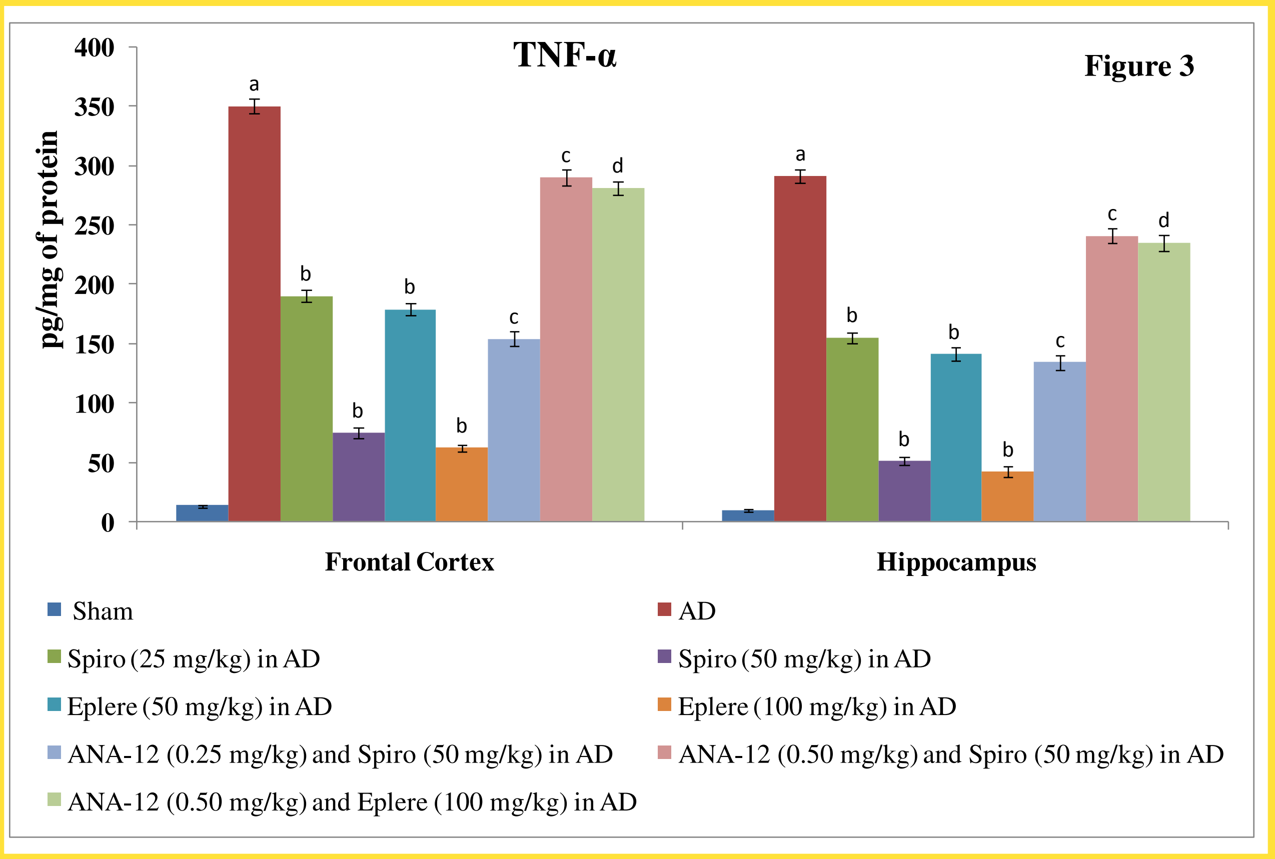

Figure 3: Effect of different interventions on TNF-α levels in the brain homogenate. a= $p< 0.05$ *vs*. sham; b = $p< 0.05$ *vs*. AD; c = $p< 0.05$ *vs*. Spiro (50 mg/kg) in AD; d = $p< 0.05$ *vs*. Eplere (100 mg/kg) in AD. Spiro: Spironolactone; Eplere: Eplerenone.

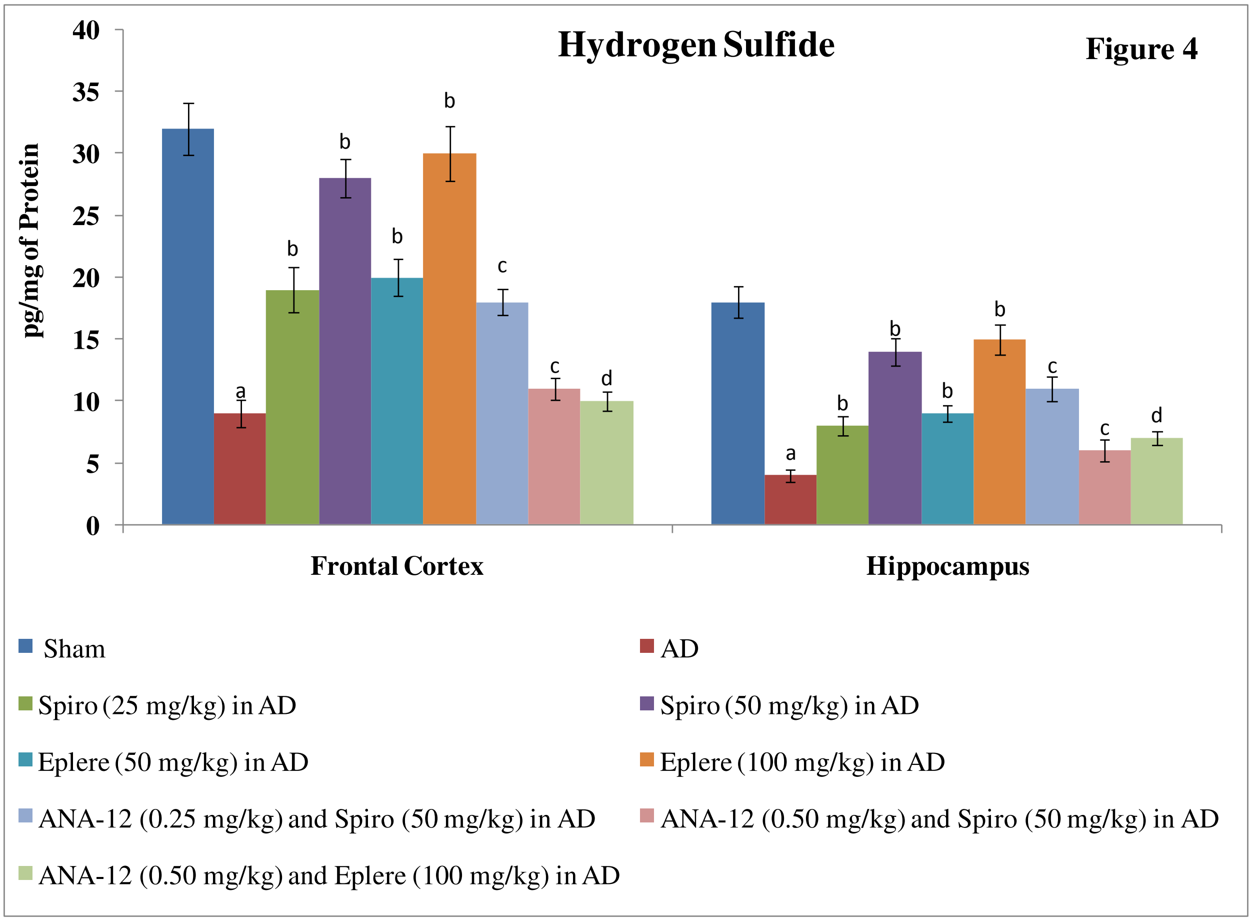

Figure 4: Effect of different interventions on H_2S levels in the brain homogenate. a= $p< 0.05$ *vs*. sham; b = $p< 0.05$ *vs*. AD; c = $p< 0.05$ *vs*. Spiro (50 mg/kg) in AD; d = $p< 0.05$ *vs*. Eplere (100 mg/kg) in AD. Spiro: Spironolactone; Eplere: Eplerenone.

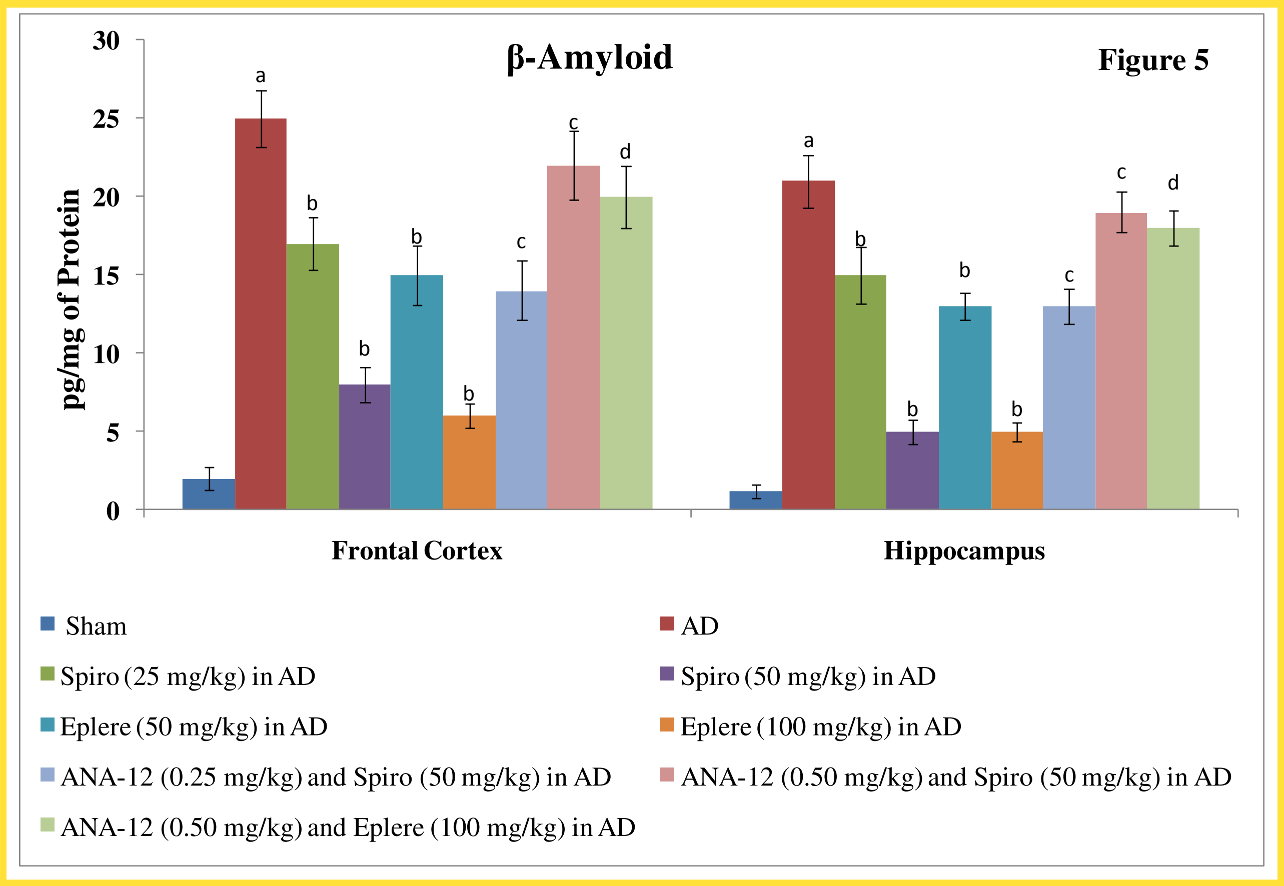

Figure 5: Effect of different interventions on β-amyloid levels in the brain homogenate. a= $p<$ 0.05 *vs*. sham; b = $p<$ 0.05 *vs*. AD; c = $p<$ 0.05 *vs*. Spiro (50 mg/kg) in AD; d = $p<$ 0.05 *vs*. Eplere (100 mg/kg) in AD. Spiro: Spironolactone; Eplere: Eplerenone.

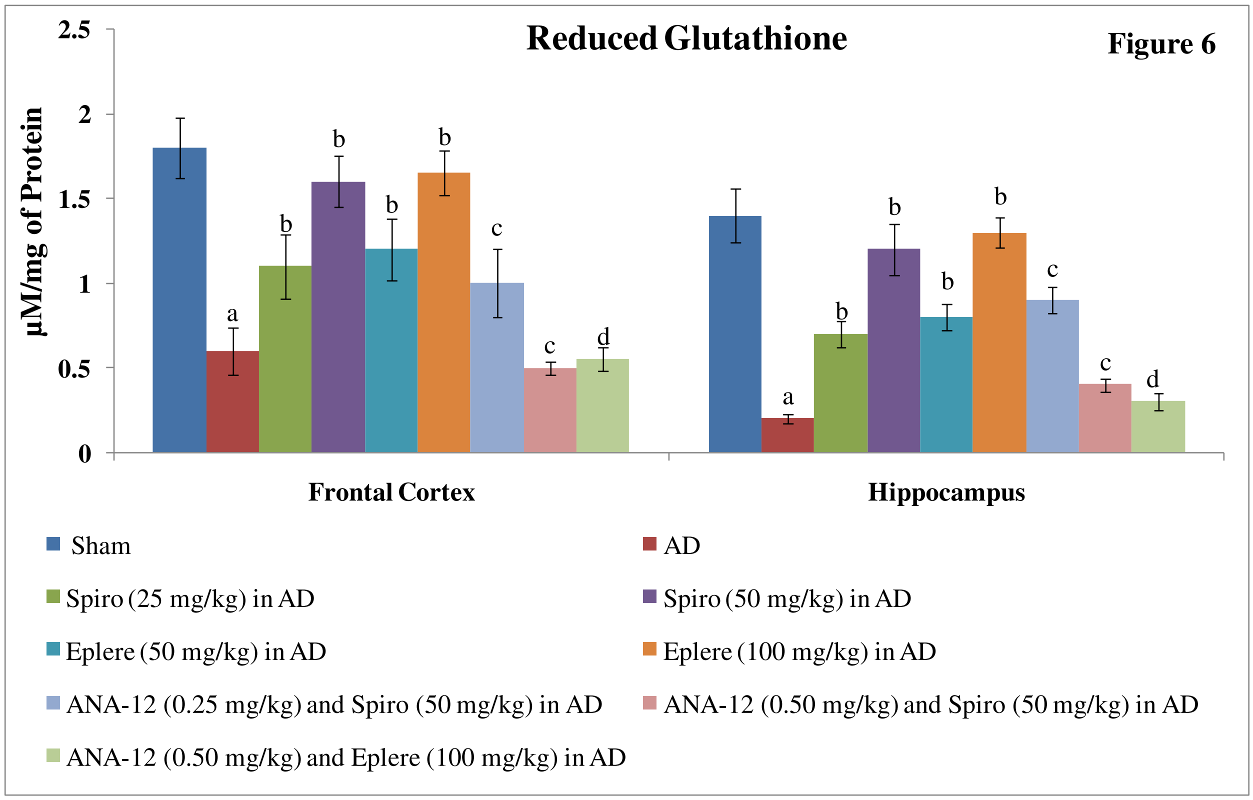

Figure 6: Effect of different interventions on reduced glutathione levels in the brain homogenate. a= $p < 0.05$ *vs*. sham; b = $p < 0.05$ *vs*. AD; c = $p < 0.05$ *vs*. Spiro (50 mg/kg) in AD; d = $p < 0.05$ *vs*. Eplere (100 mg/kg) in AD. Spiro: Spironolactone; Eplere: Eplerenone.

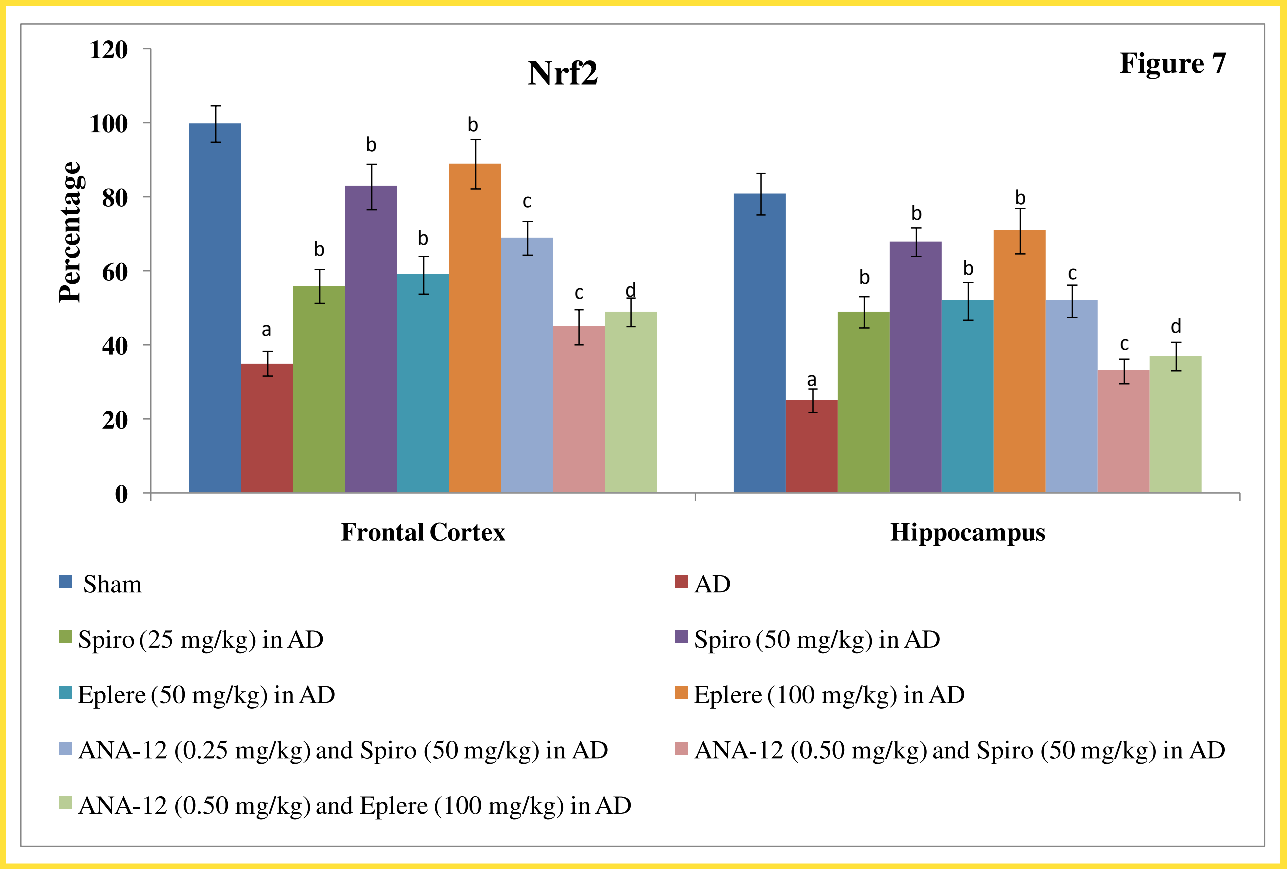

Figure 7: Effect of different interventions on Nrf2 levels in the brain homogenate. a= $p < 0.05$ *vs.* sham; b = $p < 0.05$ *vs.* AD; c = $p < 0.05$ *vs.* Spiro (50 mg/kg) in AD; d = $p < 0.05$ *vs.* Eplere (100 mg/kg) in AD. Spiro: Spironolactone; Eplere: Eplerenone.

Table 1: Effect of different interventions on escape latency time (ETL) in Morris Water Maze test. a= *p*< 0.05 *vs*. day 1 ELT of sham; b = *p*< 0.05 *vs*. day 4 ELT of sham; c = *p*< 0.05 *vs*. day 4 ELT of AD control; d = *p*< 0.05 *vs*. day 4 ELT of spironolactone (50 mg/kg) in AD; e = *p*< 0.05 *vs*. day 4 ELT of eplerenone (100 mg/kg) in AD.

**S. No**	**Groups**	**Day I ELT (s)**	**Day 4 ELT (s)**
1	Sham Control	95.2 ± 5.8	30.6 ± 3.0[a]
2.	AD control	104.4 ± 6.6	86.1 ± 5.7[b]
3.	Spironolactone (25 mg/kg) in AD control	93.1 ± 5.8	67.2 ± 4.3[c]
4.	Spironolactone (50 mg/kg) in AD control	94.5 ± 6.0	43.3 ± 5.0[c]
5.	Eplerenone (50 mg/kg) in AD control	92.6 ± 5.2	64.5 ± 6.2[c]
6.	Eplerenone (100 mg/kg) in AD control	93.3 ± 4.2	41.4 ± 3.1[c]
7.	ANA-12 (0.25 mg/kg) in spironolactone (50 mg/kg) in AD control	92.6 ±4.7	58.2 ± 3.3[d]
8.	ANA-12 (0.50 mg/kg) in spironolactone (50 mg/kg) in AD control	94.6 ±4.9	78.8 ± 5.3[d]
9.	ANA-12 (0.25 mg/kg) in eplerenone (100 mg/kg) in AD control	92.4 ±4.1	75.0 ± 5.0[e]
