# Supplementary material for: Fake paper identification in the pool of withdrawn and rejected manuscripts submitted to Naunyn–Schmiedeberg’s Archives of Pharmacology
Source: Naunyn Schmiedebergs Arch Pharmacol. 2023 Oct 5;397(4):2171–81. doi: 10.1007/s00210-023-02741-w (PMC10933159; doi:10.1007/s00210-023-02741-w)
Supplement: Supplementary file 2 — Supplementary file2 (PDF 1280 KB) [file 210_2023_2741_MOESM2_ESM.pdf]

Figure S2

Color coding:

Yellow highlighted text	The text is identical in the NSAP version and the published version of this paper.
Red highlighted text	There are differences in the text between the NSAP version and the published version of this paper (different content or different wording).
Yellow bordered figure	This figure is identical in both versions of this paper.

Naunyn-Schmiedeberg's Archives of Pharmacology

Ligustrazine Attenuates Myocardial Injury Induced by Coronary Microembolization in Rats by Activating PI3K/Akt Pathway

--Manuscript Draft--

**Manuscript Number:**	NSAP-D-19-00046
**Full Title:**	Ligustrazine Attenuates Myocardial Injury Induced by Coronary Microembolization in Rats by Activating PI3K/Akt Pathway
**Article Type:**	Original Article
**Corresponding Author:**	Qing Mao<br>Southeast University Zhongda Hospital<br>CHINA
**Corresponding Author Secondary Information:**	
**Corresponding Author's Institution:**	Southeast University Zhongda Hospital
**Corresponding Author's Secondary Institution:**	
**First Author:**	Qing Mao
**First Author Secondary Information:**	
**Order of Authors:**	Qing Mao
	Xiulin Liang
	Yufu Wu
	Yongxiang Lu
**Order of Authors Secondary Information:**	
**Funding Information:**	
**Abstract:**	Background/Aims: Myocardial injury, progressive cardiac dysfunction, which are induced by Coronary microembolization (CME), are primarily due to CME-induced local myocardial inflammatory response and myocardial apoptosis. Ligustrazine plays an important protective role in multiple cardiovascular diseases, but its role and the protection mechanism in CME is unclear. This study hypothesised that ligustrazine attenuates CME induced myocardial injury in rats. This study also explored the mechanism underlying this attenuation.<br>Methods: Forty SD rats were randomly divided into CME group, Ligustrazine group, Ligustrazine+LY294002 (Ligustrazine+LY) group and Sham group (ten rats in each). In each group, the cardiac function, apoptotic index, serum c-troponin I (cTnI) level, inflammation [interleukin-6 (IL-6) and tumor necrosis factor-alpha (TNF-α)] and oxidative stress [nitric oxide (NO), superoxide dismutase (SOD), and malondialdehyde (MDA)] were determined. Western blotting was used to detect the proteins which are present in PI3K/Akt pathway.<br>Results: Ligustrazine improved cardiac dysfunction induced by CME, increased serum NO and SOD activities, and decreased the serum level in IL-6, MDA, cTnI and TNF-α. Moreover, ligustrazine inhibited myocardial apoptosis, which is perhaps caused by upregulated Bcl-2, downregulated cleaved caspase-3 and Bax, and the increased protein level in endothelial nitric oxide synthase and phosphorylated Akt. These effects, however, were reduced if ligustrazine was coadministered with LY294002.<br>Conclusions: Ligustrazine attenuates CME-induced myocardial injury. The effects associated with this attenuation are realized by activating myocardium PI3K/Akt signaling pathway.
**Suggested Reviewers:**	Qiang Su<br>suqiang1983@foxmail.com<br>Expert in this field
	Lang Li

Powered by Editorial Manager® and ProduXion Manager® from Aries Systems Corporation

	drlangli@yeah.net<br>Expert in this field
	Jinmin Zhao<br>drzhaojinmin@163.com<br>Expert in this field

Powered by Editorial Manager® and ProduXion Manager® from Aries Systems Corporation

Manuscript

Click here to access/download;Manuscript;manuscript.docx

Click here to view linked References

Ligustrazine Attenuates Myocardial Injury Induced by Coronary Microembolization in Rats by Activating PI3K/Akt Pathway

Qing Mao[1,*], Xiulin Liang[2], Yufu Wu[3], Yongxiang Lu[4]

[1]Department of Cardiology, Nanjing Lishui People's Hospital, Zhongda Hospital Lishui Branch, Southeast University, Nanjing 211200, China.

[2]Department of Neurology, The Second Affiliated Hospital of Guangxi Medical University, Nanning, Guangxi Zhuang Autonomous Region 530007, China.

[3]Department of Cardiology, The Guangxi Zhuang Autonomous Region Brain Hospital, Liuzhou, Guangxi Zhuang Autonomous Region 545005, China.

[4]Department of Cardiology, The Second Affiliated Hospital of Guangxi Medical University, Nanning, Guangxi Zhuang Autonomous Region 530007, China.

*Corresponding author: Qing Mao Email: drmaoqing@126.com

Abstract

Background/Aims: Myocardial injury, progressive cardiac dysfunction, which are induced by Coronary microembolization (CME), are primarily due to CME-induced local myocardial inflammatory response and myocardial apoptosis. Ligustrazine plays an important protective role in multiple cardiovascular diseases, but its role and the protection mechanism in CME is unclear. This study hypothesised that ligustrazine attenuates CME induced myocardial injury in rats. This study also explored the mechanism underlying this attenuation.

Methods: Forty SD rats were randomly divided into CME group, Ligustrazine group, Ligustrazine+LY294002 (Ligustrazine+LY) group and Sham group (ten rats in each). In each group, the cardiac function, apoptotic index, serum c-troponin I (cTnI) level, inflammation [interleukin-6 (IL-6) and tumor necrosis factor-alpha (TNF-α)] and oxidative stress [nitric oxide (NO), superoxide dismutase (SOD), and malondialdehyde (MDA)] were determined. Western blotting was used to detect the proteins which are present in PI3K/Akt pathway.

Results: Ligustrazine improved cardiac dysfunction induced by CME, increased serum NO and SOD activities, and decreased the serum level in IL-6, MDA, cTnI and TNF-α. Moreover, ligustrazine inhibited myocardial apoptosis, which is perhaps caused by upregulated Bcl-2, downregulated cleaved caspase-3 and Bax, and the increased protein level in endothelial nitric oxide synthase and phosphorylated Akt. These effects, however, were reduced if ligustrazine was

coadministered with LY294002.

Conclusions: Ligustrazine attenuates CME-induced myocardial injury. The effects associated with this attenuation are realized by activating myocardium PI3K/Akt signaling pathway.

Key words: ligustrazine; coronary microembolization; PI3K/Akt; oxidative stress; inflammation; apoptosis

Introduction

A serious complication in distal microvascular embolism, coronary microembolization (CME) is normally attributed to the detachment of atherosclerotic plaque debris which occurs in percutaneous coronary intervention (PCI). CME can induce slow flow or no reflow, and therefore is considered to be an independent predictor for major cardiac adverse events and poor long-term prognosis[1-3]. Previous studies have shown that, in progressive cardiac dysfunction, CME induced local myocardial inflammation is the primary factor in causing this disease[4,5]. During the development of CME induced progressive cardiac insufficiency and advanced heart failures, the massive release of inflammatory mediators, for example IL-1βand TNF-α, plays a vital role [6,7]. In addition, several animal studies have shown that, the necrotic/apoptotic cardiomyocytes and the microembolic areas occur during the acute phase of CME [8,9]. In cardiac systolic dysfunction induced by CME, cardiomyocyte apoptosis

plays a key role, and therefore its suppression reduces CME-induced myocardial injuries[10,11]. Su et al [12] reported that cardiomyocyte apoptosis after CME in rats caused myocardial injury. Nicorandil pretreatment significantly reduced CME-induced cardiomyocyte apoptosis and consequently improved myocardial contractile function by activating PI3K/Akt signaling pathway.

Ligustrazine is an alkaloid monomer which is generally extracted from the rhizome of Chinese medicinal plants that belong to umbelliferae family, and is an amide alkaloid. It has wide range of pharmacological activities, with high safety and fewer side effects[13]. Ligustrazine has multiple cardiovascular protective effects according to recent studies on cardiovascular diseases, such as anti-oxidative stress [14], anti-inflammation [15], anti-apoptosis [16], anti-platelet aggregation [17], and amelioration of microcirculation [18]. Although it remains unclear whether ligustrazine protects myocardium, a study by zhang et al shows that ligustrazine improves the cardiac function in CME rats[19]. However, the exact mechanism underlying this improvement is unclear. In this condition, an improved understanding of the relationship between ligustrazine and cardiac function in rats with CME is required to aid the therapy and prevention of CME. Therefore, this study investigated the effect of ligustrazine intervention on cardiomyocyte apoptosis, myocardial inflammation and oxidative stress in CME rats, as well as the PI3K/Akt signaling pathway, which aimed to clarify the mechanism underlying the protection of myocardial injury induced by CME from ligustrazine.

Materials and methods

Animal preparation

The Institutional Animal Care and Use Committees at the Guangxi Medical University approved all the procedures, which were then carried out as per a protocol on Use of Laboratory Animals which is released from the National Institute of Health Guidelines. Forty Sprague-Dawley rats (male, weighted 250-300 g) were provided by Guangxi Medical University. The rats in the experiment were maintained in humidity and temperature controlled houses (50% and 25℃), with standard laboratory chow and water, in a controlled 12 h/12h-light–dark cycle.

Establishment of CME model and experimental grouping

Firstly 30-40 mg/kg pentobarbital was injected intraperitoneally into the rats to keep them under anesthesia. Then tracheotomy was performed, with a ventilator to assist breathing, with the method described by Wang et al [20]. After this step, thoracotomy was then performed on the left sternal border between the 3rd and 4th intercostal spaces. After this step was finished, the ascending aorta was then clamped for a time of 10 s by a vascular clamp after being separated. Approximately 3000 microspheres (suspended in normal saline of 0.1 mL) with 42 μm diameter (Biosphere Medical Inc., Rockland) were rapidly injected by means of a microinjector from the apex of the left ventricle. After

breathing is stabilized, the chest was closed in layers and tracheal intubation was removed. Similarly, saline of 0.1 mL was injected into each rat in the sham group. . The 40 SD rats were divided in a random and equal manner into CME group, Ligustrazine group, Ligustrazine+LY group, as well as the Sham group. Each rat in the Ligustrazine group was administered intragastrically with 27 mg/kg/d ligustrazine (Beijing Yanjing Pharmaceutical Co., Ltd,Beijing, China) for 14 d prior to building the CME model; whilst in the Ligustrazine+LY group, as well as the same administration as the Ligustrazine group, each rat was injected intraperitoneally with LY294002 at 30 min prior to building the CME model, at a dose of 10 mg/kg.

Detection of cardiac function

At 12 hours following CME, observation was performed as it was confirmed by Su et al.'s study that it is at this point of time that the lowest cardiac function occurs [21]. Here a Hewlett Packard Sonos 7500 Ultrasound instrument, with a 12 MHz frequency probe, (Philips Technologies, Amsterdam, NY), was applied in this investigation to measure cardiac output (CO), left ventricle fractional shortening (LVFS), left ventricular ejection fraction (LVEF), and left ventricular end diastolic diameter (LVEDd). In all the measurements, averaged values of triple cardiac cycles were adopted. The echocardiography was conducted by an expert.

Measurement of serum cardiac troponin I (cTnI) level

At 12 hours following CME or sham operation and before sacrificing, 1.0 ml blood was collected at the position of femoral vein, and then serum cTnI level I was determined in line with the Kit instructions. (Roche, Inc., Basel, Switzerland).

Material collection and sample processing

After the detection of cardiac function in the former step, potassium chloride (2 mL, 10%) was injected into each rat at the position of the tail vein, for the purpose that the heart of each rat can be harvested immediately while in the ventricular diastolic phase. Atrial appendage as well as the atria were excluded in the experiment. The ventricle was separated into heart base and the apex at the midpoint of the left ventricle, in a fashion of parallel to the atrioventricular groove. After being processed in liquid nitrogen, the apex was immediately transferred to and preserved at a -80 °C refrigerator for the following western blot detection. The base of the heart was embedded using paraffin and then sliced continuously (4 µm for each slice) after being fixed for 12 h with 4% paraformaldehyde. The slices were used for the following staining with hematoxylin-basic fuchsin-picric acid (HBFP) (to observe myocardial microinfarct areas), TdT-mediated dUTP Nick-End Labeling (TUNEL) and hematoxylin-eosin (HE), which aimed to observe myocardial microinfarct areas.

Detection of cardiomyocyte apoptosis with TUNEL assay

According to the instructions of the kit (Roche, USA), the apoptotic nuclei was yellow (TUNEL positive) under the light microscope. In each slice (×400

magnification), the number of total cardiomyocytes and apoptotic cardiomyocytes in the microinfarct zone, the infarct zone and the infarct edge zone were calculated from 40 randomly chosen solitary areas. The apoptosis index (AI) of cardiomyocytes was determined by dividing the apoptotic cardiomyocytes number by the gross cardiomyocytes number× 100% [22].

Measurement of myocardial microinfarct areas

In diagnosing early myocardial ischemia, HBFP staining can stain nucleus in blue color, normal myocardial cytoplasm in yellow, as well as ischemic myocardium and red blood cells in red. A DMR+Q550 pathological image analyzer (Leica, Wetzlar, Germany) was used to observe (×100 magnification) each of the HBFP-stained slice. For each slice, five random visual fields were selected. The Leica Qwin analysis software plane was applied in this study to determine the infarct area, which was then divided by gross observed area to calculate the infarct percentage [23].

Antioxidant enzymes assay

Commercial kits were used to measure serum SOD, MDA and NO level as per the Kit instructions.

Enzyme-linked immunosorbent assay (ELISA) used in detecting inflammatory cytokines in serum

Level of TNF-α and IL-1β in serum was determined by means of a ELISA kit

(R&D Systems, Minneapolis, MN) as per the Kit instructions.

Western blot analysis

10%-15% SDS-PAGE was used to separate the total protein which was collected in the cardiomyocytes and cardiac tissue before they were electrotransferred to PVDF membrane (Millipore, Atlanta, US), which were blocked with non-fat milk or 5% bovine serum albumin for 1.5h at room temperatures before they were incubated at 4℃ overnight by using media of primary antibodies against p-Akt, Bcl-2, total Akt, Bax, cleaved caspase-3 or GAPDH. All the antibodies were provided by Cell Signaling Technology (Beverly, USA). Secondary antibodies conjugated with horseradish peroxidase were used to incubate the membranes in TBST for 2h at room temperatures after TBS containing 0.1% Tween 20 (TBST) was used to wash the membranes for 5 times. A chemiluminescence detecting equipment (enhanced version, Pierce, Holmdel, US) was used to detect the signals. Image Lab software (Bio-Rad Laboratories, Hercules, CA) was used to assess and quantify the bands for protein amounts.

Statistical analysis

Statistical analysis was carried out by means of the SPSS 20.0 software (IBM, Chicago, IL). Data were presented in a format of mean value ± standard deviation. Differences were compared by means of the method one way ANOVA. P values <0.05 were considered statistical significance. GraphPad Prism software version 5.0 (GraphPad Software, Inc., San Diego, CA) was used to conduct all of the

statistical tests.

Results

Ligustrazine improved cardiac function after CME

Table 1 shows that cardiac dysfunction was induced by CME, which was characterized by increased left ventricular end-diastolic diameter and decreased cardiac output, left ventricular end-systolic diameter, fractional shortening, and left ventricular ejection fraction. The cardiac dysfunction caused by CME was improved significantly by Ligustrazine pretreatment, whilst LY294002 (a specific inhibitor of PI3K/Akt signaling pathway) attenuated these protective effects.

Ligustrazine reduced serum cTnI level after CME

As shown in Fig. 1, in the CME group, serum cTnI levels were significantly enhanced compared to the levels measured in the Sham group. On the other hand, Ligustrazine significantly inhibited its increase after CME. LY294002 treatment eliminated these effects of ligustrazine, and the levels of cTnI were significantly higher in the ligustrazine+LY group than in the ligustrazine group.

Effects of ligustrazine on SOD, MDA and NO

Figure 2 indicates that, in comparison to Sham group, CME group exhibits significantly increased MDA content and significantly decreased SOD and NO level; while these changes were reversed in ligustrazine group (P<0.05), all of

which indicate that, for CME-induced myocardial injury, ligustrazine demonstrates an antioxidative stress effect. However, the effect of ligustrazine on levels of SOD, NO and MDA is significantly abolished by LY294002. This indicates that ligustrazine's attenuating effect on oxidative stress in CME-induced myocardial injuries is closely related with PI3K/Akt signaling pathway.

Effect of ligustrazine on TNF-α and IL-1β

Compared to the Sham group, levels of IL-1β and TNF-α were increased significantly (Fig. 3). These levels were inhibited by the administration of ligustrazine. However, the serum IL-1β and TNF-α concentrations in the ligustrazine+LY group were higher than in the ligustrazine alone group. These results indicated that ligustrazine can activate PI3K/Akt signaling. For this reason, the inflammatory cytokine secretion which is induced by CME in the serum is significantly suppressed.

Pathological observation of CME

HE and HBFP staining results: Albeit no obvious infarcts were noted, subendocardial ischemia occurred occasionally in sham group. In the other three groups, however, multiple microinfarctions were observed. These lesions were mostly wedge-shaped with a focal distribution and were more common in the subendocardial and left ventricle, as shown in Figure 4. HE staining showed that myocardial cell nucleus dissolved or disappeared in the microinfarction, cytoplasmic red staining, degeneration, peripheral myocardial edema, red blood

cell exudation and peripheral inflammatory cell infiltration, and microembolism in arteriole (Figure 5). The infarct size of the CME group, ligustrazine group and ligustrazine+LY group were (9.17±2.79)%, (5.01±1.26)%, and (9.03±3.12)%, respectively. Compared with CME group, in the ligustrazine group the myocardial infarct size was reduced significantly. The apoptotic index of cardiomyocytes was (8.04±1.57)%, (3.38±0.63)%, (0.39±0.094)%, and (7.92±1.65)% in CME group, ligustrazine group, Sham group, and ligustrazine+LY group, respectively (Fig. 6). The apoptotic index of cardiomyocytes in the two groups, i.e. the CME group and ligustrazine+LY group, was significantly increased compared with the Sham group; whilst this index was decreased significantly in the ligustrazine group, compared to the CME group.

Ligustrazine effects on myocardial apoptosis

Cleaved caspase-3, Bcl-2 and Bax protein expression was detected to confirm cardiomyocyte apoptosis after CME. Cleaved caspase-3 protein expression in the CME group demonstrated a significant increase compared to the Sham group (Figure 7), and the Bcl-2/Bax ratio was downregulated significantly. After pretreatment with ligustrazine, cleaved caspase-3 expression showed significant decrease; the Bcl-2/Bax ratio showed significant increase; whilst EX527 attenuated these anti-apoptotic effects. However, if co-treated with LY, the effect of ligustrazine on myocardial apoptosis was significantly attenuated relative to that in ligustrazine group.

Ligustrazine effects the expression of proteins in PI3K/Akt pathway

No difference was detected among the four groups regarding the expression of total Akt and eNOS (see Fig.8). Myocardial levels of p-Akt and p-eNOS were increased significantly after Ligustrazine treatment relative to those in CME group. However, this upregulation of myocardial p-Akt and p-eNOS induced by ligustrazine was attenuated significantly by LY294002.

Discussion

The data presented in this study demonstrated that ligustrazine could protect cardiac function in CME rats, which is associated with suppression of - the damage and inflammatory cytokines which are triggered by oxidative stress. With these alterations, the cleaved caspase-3 as well as the Bax expression was decreased, the Bcl-2 expression involved in PI3K/Akt signaling pathway was up-regulated, and consequently the apoptosis after CME was attenuated.

CME often occurs in patients who suffer from unstable plaque ruptures and acute coronary syndrome during PCI. Unlike epicardial proximal vascular occlusion, the CME-induced decrease in left ventricular function has no close connection with the extent of myocardial perfusion defects [24]. This phenomenon cannot be explained by the lack of local myocardial perfusion or microinfarcts. It is now believed that inflammatory reaction and cardiomyocyte apoptosis in normal myocardial tissues around the microinfarction are

associated with myocardial injury as well as the progressive cardiac dysfunction after CME [25,26]. In the above-mentioned myocardial injury, however, the PI3K/Akt signaling pathway played a critical role[27]. This study suggests that the levels of serum cTnI, oxidative stress, inflammatory factors, and myocardial apoptosis index are significantly elevated in rats after CME, whilst at the same time the cardiac function is deteriorated. Moreover, it is showed here that the PI3K/Akt signaling pathway is down-regulated, which indicates that the modeling is successful, in the sense that it is consistent with pathophysiological change in CME.

Ligustrazine is an effective monomer component in the traditional Chinese medicine ligusticum wallichii. Ligustrazine can resist platelet aggregation, dilate small arteries, improve microcirculation and promote blood circulation and phlegm. Due to its multiple functions, high safety and diverse mechanisms, it has potential application prospects in cardiovascular diseases[28]. Previous studies have found that ligustrazine pretreatment can reduce MDA levels by 19.2%, increase SOD activity by 39.6%, and reduce oxidative stress in a renal ischemia-reperfusion model of C57BL/6 mice with clamped left renal artery [29]. In a cisplatin-induced rat tubular toxicity model, ligustrazine demonstrates dose-related anti-apoptotic and anti-oxidative effects [30]. In a rat model of cerebral ischemia-reperfusion injury, ligustrazine effectively reduces MDA levels and increased SOD content [31]. Ligustrazine also preserves mitochondrial integrity and mitochondrial function, and reduces oxidative brain damage by

reducing the production of oxygen free radical, suggesting that ligustrazine has antioxidative effect [32]. The data presented in this study shows that ligustrazine in a rat CME model can effectively inhibit myocardial inflammation, reduce oxidative stress level, scavenge oxygen free radicals, reduce lipid peroxidation, and protect mitochondrial structure and function, thereby reducing myocardial injury induced by CME.

In recent years, PI3K/Akt signaling pathway has been the focus for reducing CME induced myocardial injury, as a common pathway for many drugs to achieve myocardial protection [12,27]. A variety of intracellular signal transduction mediators and effector proteins are involved in PI3K/Akt signaling pathway, wherein eNOS is one of the downstream targets of signaling pathways [33]. Activated Akt promotes phosphorylation of serine 1177 at eNOS, thereby up-regulating eNOS expression, ultimately reducing the mitochondrial permeability transition pore opening, maintaining mitochondrial outer membrane stability, and improving mitochondrial energy production. This in turn reduces apoptosis and protects the myocardium [34]. In this study, ligustrazine up-regulates the expression of eNOS phosphorylation, while PI3K/Akt signaling pathway inhibitor LY294002 abolishes the phosphorylation of eNOS induced by ligustrazine, indicating that the activation of eNOS by ligustrazine is produced by PI3K/Akt signaling pathway. However, serum cTnI, inflammatory cytokines, oxidative stress, myocardial apoptosis and cardiac function inligustrazine+LY group show no significant differences from those in

the CME group, suggesting that LY294002 can attenuate myocardial protection of ligustrazine. Therefore, this study demonstrates that PI3K/Akt signaling pathway is closely associated with the protective effects of ligustrazine on myocardial injury. Ligustrazine may exert myocardial protection by activating PI3K/Akt signaling pathway and the downstream eNOS, and by sequentially activating the downstream targets.

In summary, ligustrazine has a definite effect on anti-CME-induced myocardial injury. This effect is primarily due to the reduction of myocardial apoptosis, oxidative stress, as well as the myocardial inflammation through PI3K/Akt signaling pathway activation. The data presented in this study can be used as a theoretical basis in applying ligustrazine in the therapy and prevention of myocardial injury induced by CME.

Author's contribution QM and XL conceived and designed research. YW and YL conducted experiments. XL contributed new reagents or analytical tools. YL analyzed data. QM wrote the manuscript. All authors read and approved the manuscript.

Compliance with ethical standards

Research involving human participants and/or animals All applicable international, national, and/or institutional guidelines for the care and use of animals were followed.

Conflicts of interest The authors declare that they have no conflict of interest.

Acknowledgements

This research was done without specific grant from any funding agencies in the public, commercial or not-for-profit sectors.

References

1. Heusch G, Skyschally A, Kleinbongard P. Coronary microembolization and microvascular dysfunction[J]. International journal of cardiology, 2018, 258: 17-23.

2. Bose D, von Birgelen C, Zhou XY, et al. Impact of atherosclerotic plaque composition on coronary microembolization during percutaneous coronary interventions. Basic Res Cardiol 2008;103:587-97.

3. Kurtul A, Yarlioglues M, Murat S N, et al. Usefulness of the platelet-to-lymphocyte ratio in predicting angiographic reflow after primary percutaneous coronary intervention in patients with acute ST-segment elevation myocardial infarction[J]. The American journal of cardiology, 2014, 114(3): 342-347.

4. Dörge H, Schulz R, Belosjorow S, et al. Coronary microembolization: the role of TNF-α in contractile dysfunction[J]. Journal of molecular and cellular cardiology, 2002, 34(1): 51-62.

5. Su Q, Li L, Sun Y, et al. Effects of the TLR4/Myd88/NF-κB Signaling Pathway

on NLRP3 Inflammasome in Coronary Microembolization-Induced Myocardial Injury[J]. Cellular Physiology and Biochemistry, 2018, 47(4): 1497-1508.

6. Lu Y, Li L, Zhao X, et al. Beta blocker metoprolol protects against contractile dysfunction in rats after coronary microembolization by regulating expression of myocardial inflammatory cytokines[J]. Life sciences, 2011, 88(23-24): 1009-1015.

7. Li L, Zhao X, Lu Y, et al. Altered expression of pro-and anti-inflammatory cytokines is associated with reduced cardiac function in rats following coronary microembolization[J]. Molecular and cellular biochemistry, 2010, 342(1-2): 183-190.

8. Chen Z W, Qian J Y, Ma J Y, et al. TNF-α-induced cardiomyocyte apoptosis contributes to cardiac dysfunction after coronary microembolization in mini-pigs[J]. Journal of cellular and molecular medicine, 2014, 18(10): 1953-1963.

9. Su Q, Li L, Zhao J, et al. MiRNA expression profile of the myocardial tissue of pigs with coronary microembolization[J]. Cellular Physiology and Biochemistry, 2017, 43(3): 1012-1024.

10. He W, Su Q, Liang J, et al. The protective effect of nicorandil on cardiomyocyte apoptosis after coronary microembolization by activating Nrf2/HO-1 signaling pathway in rats[J]. Biochemical and biophysical research

communications, 2018, 496(4): 1296-1301.

11. Liu T, Zhou Y, Liu Y C, et al. Coronary Microembolization induces Cardiomyocyte apoptosis through the LOX-1–dependent endoplasmic reticulum stress pathway involving JNK/P38 MAPK[J]. Canadian Journal of Cardiology, 2015, 31(10): 1272-1281.

12. Su Q, Li L, Zhao J, et al. Effects of nicorandil on PI3K/Akt signaling pathway and its anti-apoptotic mechanisms in coronary microembolization in rats[J]. Oncotarget, 2017, 8(59): 99347-99358.

13. Donkor P O, Chen Y, Ding L, et al. Locally and traditionally used Ligusticum species–A review of their phytochemistry, pharmacology and pharmacokinetics[J]. Journal of ethnopharmacology, 2016, 194: 530-548.

14. Guo L, Wang A, Sun Y, et al. Evaluation of antioxidant and immunity function of tetramethylpyrazine phosphate tablets in vivo[J]. Molecules, 2012, 17(5): 5412-5421.

15. Guo L, Yang C, Wang L, et al. Effects of tetramethylpyrazine on cardiac function and mortality rate in septic rats[J]. Chinese journal of integrative medicine, 2012, 18(8): 610-615.

16. Lin K H, Kuo W W, Jiang A Z, et al. Tetramethylpyrazine ameliorated hypoxia-induced myocardial cell apoptosis via HIF-1α/JNK/p38 and IGFBP3/BNIP3 inhibition to upregulate PI3K/Akt survival signaling[J].

Cellular Physiology and Biochemistry, 2015, 36(1): 334-344.

17. Chen H, Li G, Zhan P, et al. Ligustrazine derivatives. Part 5: design, synthesis and biological evaluation of novel ligustrazinyloxy-cinnamic acid derivatives as potent cardiovascular agents[J]. European journal of medicinal chemistry, 2011, 46(11): 5609-5615.

18. Yu K, Chen Z, Pan X, et al. Tetramethylpyrazine-mediated suppression of C6 gliomas involves inhibition of chemokine receptor CXCR4 expression[J]. Oncology reports, 2012, 28(3): 955-960.

19. Zhang Y, Ma X, Guo C, et al. Pretreatment with a combination of ligustrazine and berberine improves cardiac function in rats with coronary microembolization[J]. Acta Pharmacologica Sinica, 2016, 37(4): 463-472.

20. Wang X, Lu Y, Sun Y, et al. TAK-242 protects against apoptosis in coronary microembolization-induced myocardial injury in rats by suppressing TLR4/NF-κB signaling pathway[J]. Cellular Physiology and Biochemistry, 2017, 41(4): 1675-1683.

21. Su Q, Li L, Zhou Y, et al. Effects of pretreatment with metoprolol on cardiomyocyte apoptosis and caspase-8 activation after coronary microembolization in rats[J]. Zhonghua xin xue guan bing za zhi, 2013, 41(8): 693-697.

22. Li L, Su Q, Wang Y, et al. Effect of atorvastatin (Lipitor) on myocardial

apoptosis and caspase-8 activation following coronary microembolization[J]. Cell biochemistry and biophysics, 2011, 61(2): 399-406.

23. Su Q, Lv X, Sun Y, et al. Role of high mobility group A1/nuclear factor-kappa B signaling in coronary microembolization-induced myocardial injury[J]. Biomedicine & Pharmacotherapy, 2018, 105: 1164-1171.

24. Dörge H, Neumann T, Behrends M, et al. Perfusion-contraction mismatch with coronary microvascular obstruction: role of inflammation. American Journal of Physiology-Heart and Circulatory Physiology. 2000;279(6):H2587-92.

25. Su Q, Lv X, Sun Y, et al. Role of TLR4/MyD88/NF-κB signaling pathway in coronary microembolization-induced myocardial injury prevented and treated with nicorandil[J]. Biomedicine & Pharmacotherapy, 2018, 106: 776-784.

26. Liu T, Zhou Y, Wang J Y, et al. Coronary microembolization induces cardiomyocyte apoptosis in swine by activating the LOX-1-dependent mitochondrial pathway and caspase-8-dependent pathway[J]. Journal of cardiovascular pharmacology and therapeutics, 2016, 21(2): 209-218.

27. Wang J, Chen H, Zhou Y, et al. Atorvastatin inhibits myocardial apoptosis in a swine model of coronary microembolization by regulating PTEN/PI3K/Akt signaling pathway[J]. Cellular Physiology and Biochemistry, 2016, 38(1): 207-219.

28. Zhao Y, Liu Y, Chen K. Mechanisms and Clinical Application of Tetramethylpyrazine (an Interesting Natural Compound Isolated from Ligusticum Wallichii): Current Status and Perspective[J]. Oxidative Medicine and Cellular Longevity, 2016, 2016: 2124638.

29. Feng L, Ke N, Cheng F, et al. The protective mechanism of ligustrazine against renal ischemia/reperfusion injury[J]. Journal of Surgical Research, 2011, 166(2): 298-305.

30. Ali B H, Al-Moundhri M, Eldin M T, et al. Amelioration of cisplatin-induced nephrotoxicity in rats by tetramethylpyrazine, a major constituent of the Chinese herb Ligusticum wallichi[J]. Experimental biology and medicine, 2008, 233(7): 891-896.

31. Wan H, Zhu H, Tian M, et al. Protective effect of chuanxiongzine-puerarin in a rat model of transient middle cerebral artery occlusion-induced focal cerebral ischemia[J]. Nuclear medicine communications, 2008, 29(12): 1113-1122.

32. Li S Y, Jia Y H, Sun W G, et al. Stabilization of mitochondrial function by tetramethylpyrazine protects against kainate-induced oxidative lesions in the rat hippocampus[J]. Free Radical Biology and Medicine, 2010, 48(4): 597-608.

33. Fulton D, Gratton J P, McCabe T J, et al. Regulation of endothelium-derived nitric oxide production by the protein kinase Akt[J]. Nature, 1999, 399(6736): 597-601.

34. Balakumar P, Kathuria S, Taneja G, et al. Is targeting eNOS a key mechanistic insight of cardiovascular defensive potentials of statins?[J]. Journal of molecular and cellular cardiology, 2012, 52(1): 83-92.

Table 1 Changes in cardiac function ($x \pm s$)

Group	n	LVEF (%)	LVFS (%)	CO (L/min)	LVEDd (mm)
Sham	10	80.73±5.56	44.03±4.64	0.190±0.032	5.08±0.45
CME	10	58.68±3.77[a]	21.79±2.72[a]	0.109±0.008[a]	7.84±0.59[a]
Ligustrazine	10	69.23±3.99[ab]	37.81±4.95[ab]	0.169±0.018[ab]	6.62±0.52[ab]
Ligustrazine+LY	10	56.52±3.64[a]	20.88±2.76[a]	0.106±0.009[a]	7.89±0.65[a]

CME, coronary microembolization; LVFS, left ventricle fractional shortening; LVEDd, left ventricular end-diastolic diameter; CO, cardiac output; LY, LY294002; LVEF, left ventricle ejection fraction. [a]$P<0.05$ compared with Sham group. [b]$P<0.05$ compared with CME group.

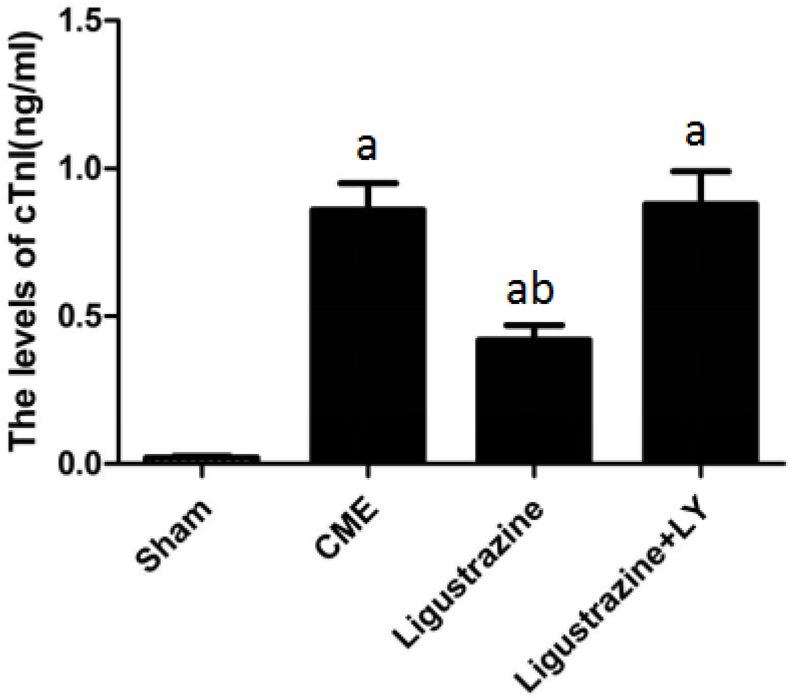

Fig. 1 Parameters of cTnI LY, LY294002; CME, coronary microembolization. [a]*P*<0.05 compared to Sham group; [b]*P*<0.05 compared to CME group. n=10.

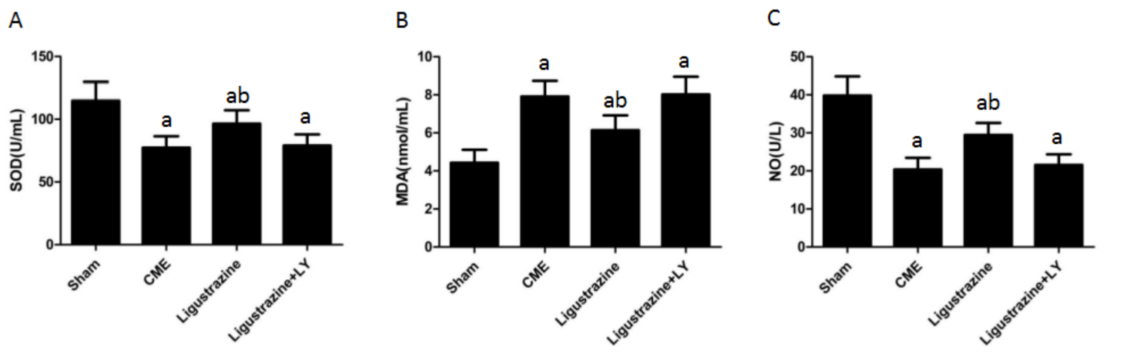

Fig. 2 Effect of ligustrazine on serum levels of cardiac oxidative stress parameters {SOD (A), MDA (B), NO (C)} CME, coronary microembolization; LY, LY294002. $^{a}P<0.05$ compared to Sham group; $^{b}P<0.05$ compared to CME group. n=10.

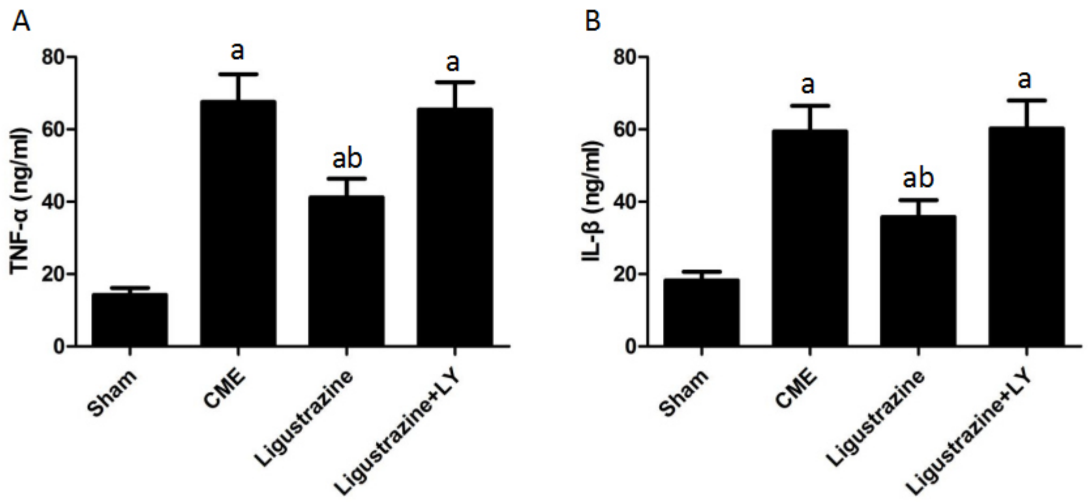

Fig. 3 Effect of ligustrazine on TNF-α (A) and IL-1β (B) in serum LY, LY294002; CME, coronary microembolization. [a]*P*<0.05 compared to Sham group; [b]*P*<0.05 compared to CME group. n=10.

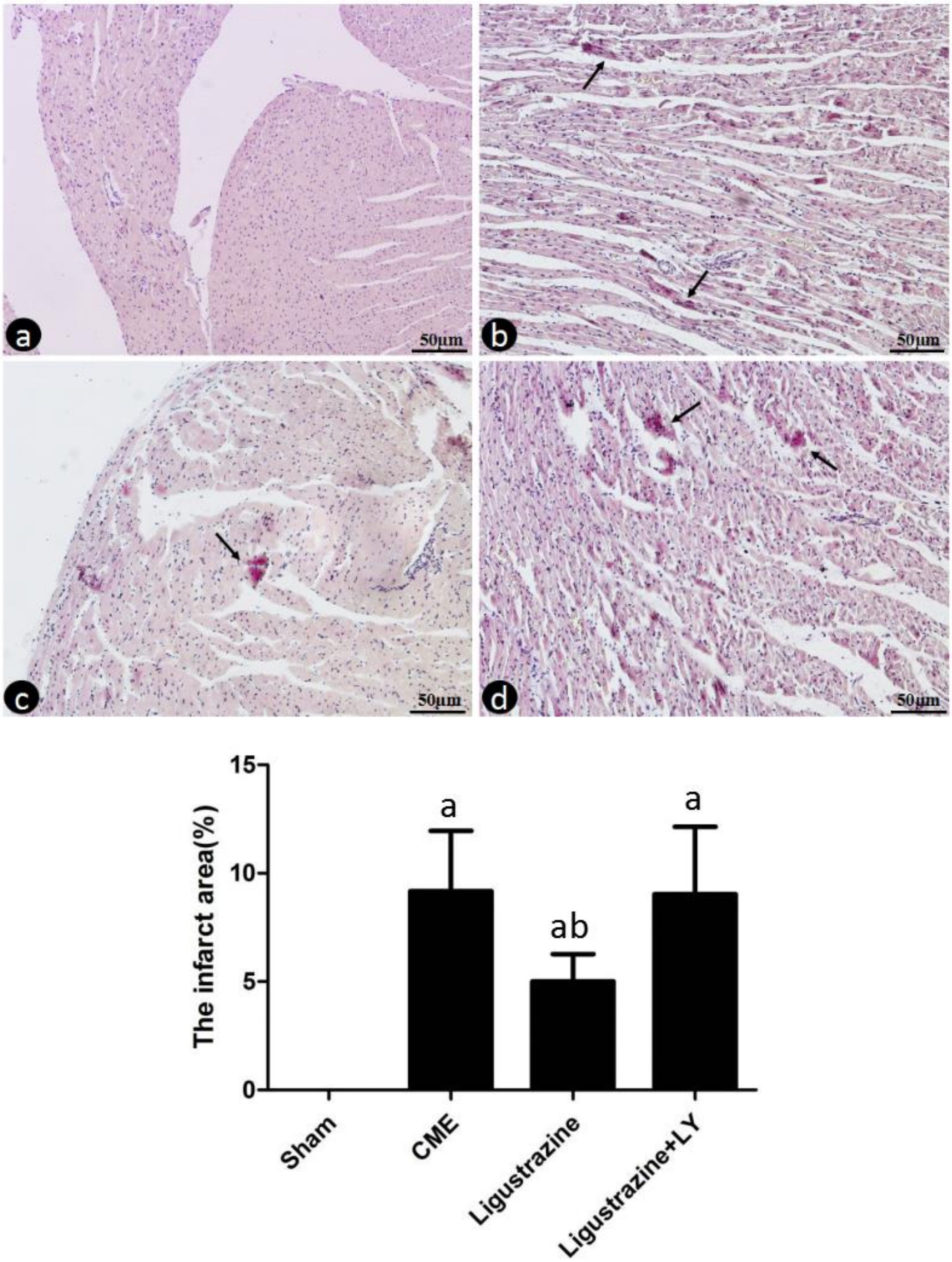

Fig. 4 Pathohistological examination by HBFP staining (magnification, ×200; bar = 50μm) a-d: Sham group, CME group, Ligustrazine group and Ligustrazine+LY group. Ischemic myocardium in red. Arrow indicates microinfarct area. LY, LY294002; CME, coronary microembolization. $^{a}P<0.05$ compared to Sham group; $^{b}P<0.05$ compared to CME group. n=10.

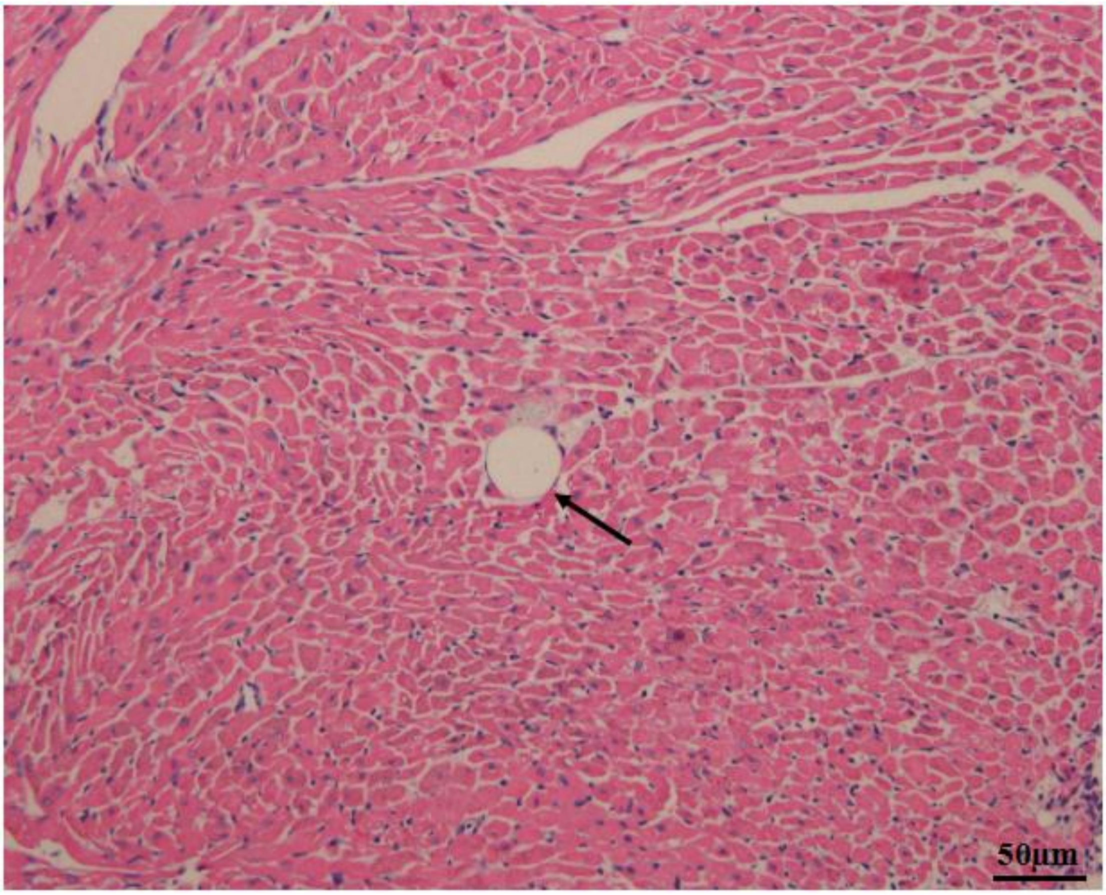

Fig. 5 At 12 hours following CME pathohistological examination by HE staining (magnification, ×400; bar = 50μm) Microspheres with inflammatory cells infiltration are showed in this HE staining. Arrow indicates microspheres. CME, coronary microembolization. n=10.

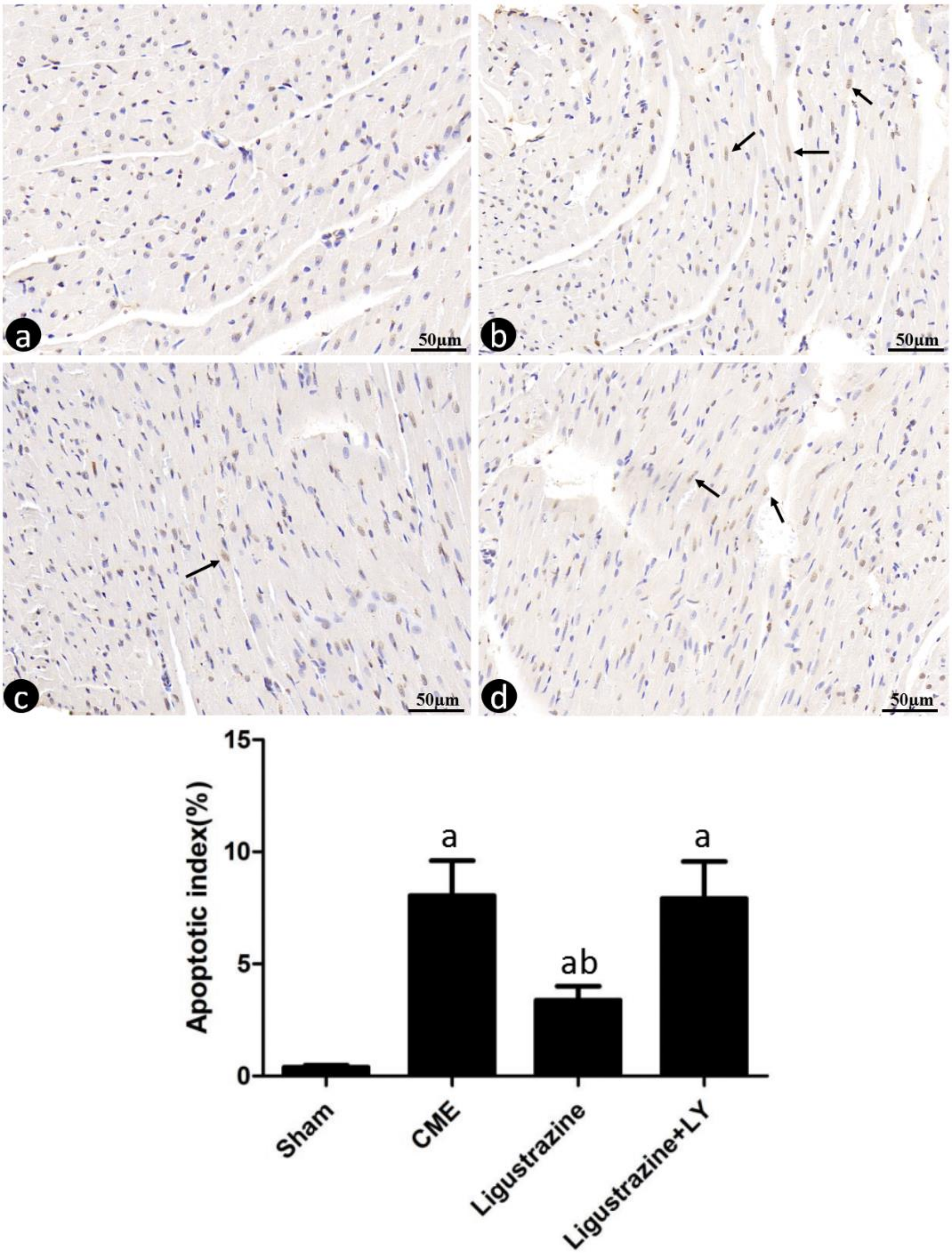

Fig. 6 Cardiomyocyte apoptosis is showed with TUNEL staining (magnification, ×400; bar = 50μm) a-d: Sham group, CME group, Ligustrazine group and Ligustrazine+LY group. Nuclei of apoptotic in yellow whilst the normal cardiomyocytes in light blue Arrow indicates nuclei of apoptotic cardiomyocytes. LY, LY294002; CME, coronary microembolization. $^{a}P<0.05$ compared to Sham group; $^{b}P<0.05$ compared to CME group. n=10.

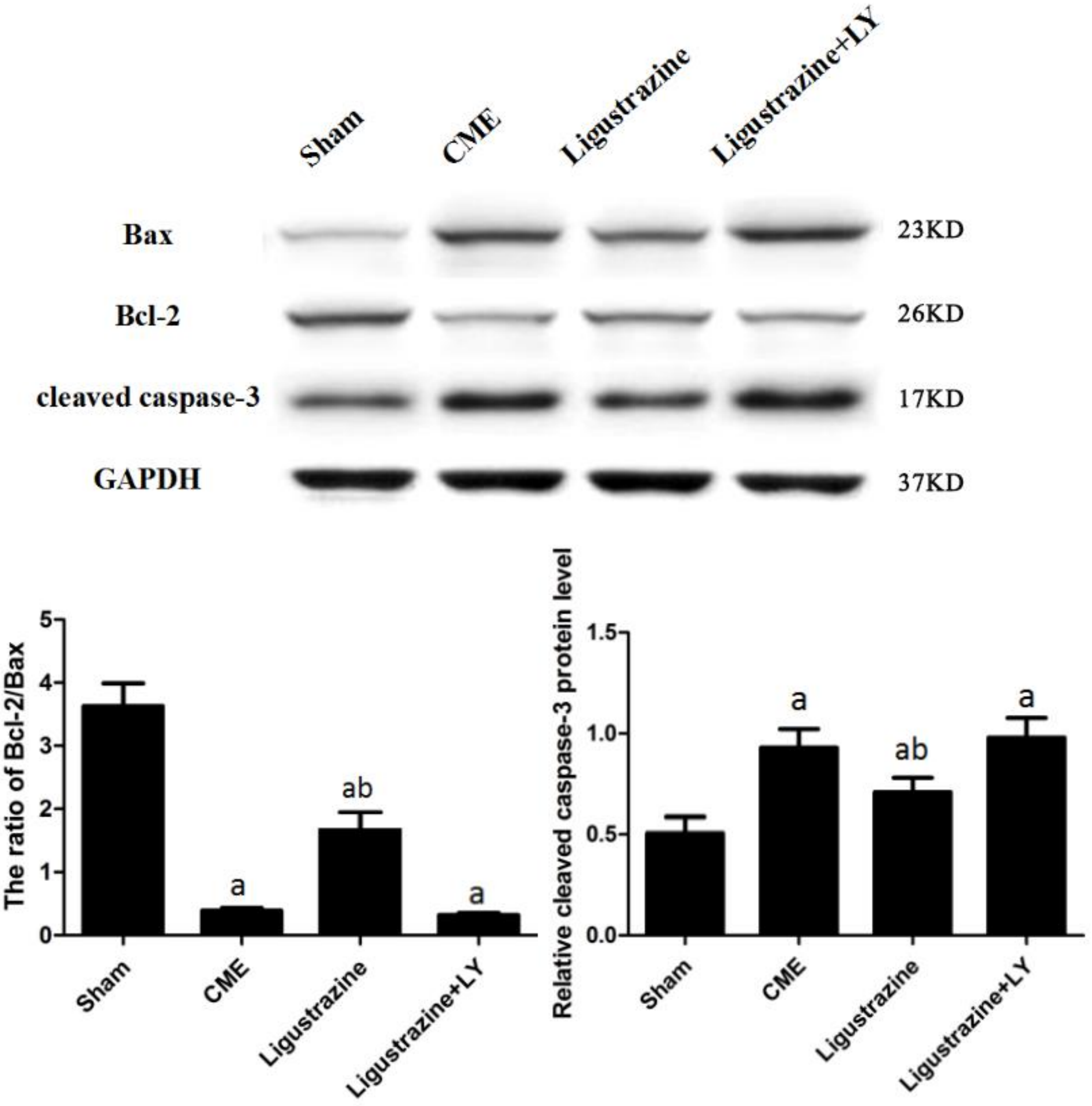

Fig. 7 Effect of ligustrazine on myocardial apoptosis LY, LY294002; CME, coronary microembolization. [a]*P*<0.05 compared to sham group; [b]*P*<0.05 compared to CME group. n=10.

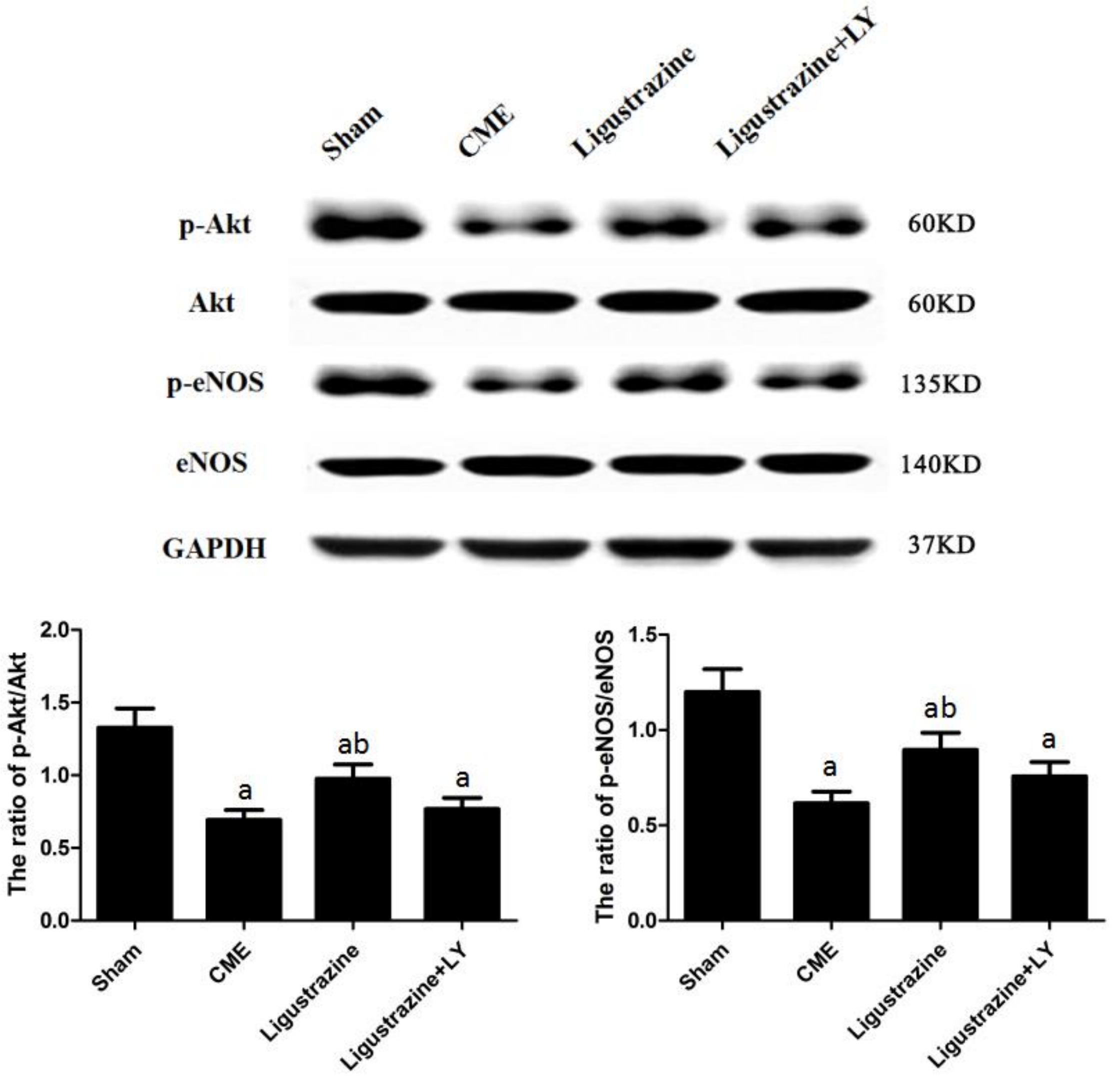

Fig. 8 Effect of ligustrazine on PI3K/Akt signaling pathway. LY, LY294002; CME, coronary microembolization. $^{a}P<0.05$ compared to sham group; $^{b}P<0.05$ compared to CME group. n=10.
