# Supplementary material for: Fake paper identification in the pool of withdrawn and rejected manuscripts submitted to Naunyn–Schmiedeberg’s Archives of Pharmacology
Source: Naunyn Schmiedebergs Arch Pharmacol. 2023 Oct 5;397(4):2171–81. doi: 10.1007/s00210-023-02741-w (PMC10933159; doi:10.1007/s00210-023-02741-w)

Figure S3

Color coding:

Yellow highlighted text	The text is identical in the NSAP version and the published version of this paper.
Red highlighted text	There are differences in the content between the NSAP version and the published version of this paper.
Blue highlighted text	The content is identical in both versions of this paper, but the text has been reworded.
Yellow bordered figure	This Figure is not identical in both versions of this paper.

Naunyn-Schmiedeberg's Archives of Pharmacology

Long non-coding RNA GAS5 aggravates acute lung injury through promoting inflammation and cell apoptosis via regulating miR-26a-5p/TLR4 axis

--Manuscript Draft--

**Manuscript Number:**	NSAP-D-20-00168
**Full Title:**	Long non-coding RNA GAS5 aggravates acute lung injury through promoting inflammation and cell apoptosis via regulating miR-26a-5p/TLR4 axis
**Article Type:**	Original Article
**Corresponding Author:**	Yanhui Du<br>Shandong Provincial Third Hospital<br>CHINA
**Corresponding Author Secondary Information:**	
**Corresponding Author's Institution:**	Shandong Provincial Third Hospital
**Corresponding Author's Secondary Institution:**	
**First Author:**	Xiaoqun Chen
**First Author Secondary Information:**	
**Order of Authors:**	Xiaoqun Chen
	Qin Jiang
	Yanhui Du
**Order of Authors Secondary Information:**	
**Funding Information:**	
**Abstract:**	Acute lung injury (ALI) is a pulmonary disorder that leads to acute respiratory failure and thereby results in a high mortality worldwide. Increasing studies have verified that TLR4 is a promoter in ALI, however, the underlying mechanisms of TLR4 was still rarely investigated. In this study, we aimed at exploring the underlying mechanism ofTLR4 in ALI. RT-qPCR identified that TLR4 expression was upregulated in ALI mice and LPS-induced WI-38 cells. Moreover, miR-26a-5p was confirmed to target TLR4 according to luciferase reporter assay. Besides, overexpression of miR-26a-5p decreased the expression levels of proinflammatory factors (TNF-α and IL-1β) and blocked cell apoptosis, while upregulation of TLR4 reversed the effect of miR-26a-5p overexpression. Therefore, miR-26a-5p alleviated lung injury through regulating TLR4. Afterwards, GAS5 was identified to bind with miR-26a-5p by RNA immunoprecipitation (RIP) and luciferase reporter assay. Functionally, GAS5 upregulation accelerated the inflammation injuries and miR-26a-5p overexpression counteracted the effect of GAS5 upregulation on proinflammatory factors and cell apoptosis. In conclusion, GAS5 accelerated ALI through regulating miR-26a-5p/TLR4 axis in ALI mice and LPS-induced cells, which indicates a promising insight into diagnostics and therapeutics in ALI.
**Suggested Reviewers:**	

Powered by Editorial Manager® and ProduXion Manager® from Aries Systems Corporation

Manuscript
Click here to access/download;Manuscript;manuscript.docx
Click here to view linked References

Long non-coding RNA GAS5 aggravates acute lung injury through promoting inflammation and cell apoptosis via regulating miR-26a-5p/TLR4 axis

Xiaoqun Chen[1, #], Qin Jiang [2, #], Yanhui Du[3, *]

[1]Department of Pediatrics, Northwest Women and Children Hospital, Xi'an City 710061, Shaanxi Province, China.

[2]Department of Pediatric Intensive Care Unit, Qilu Children's Hospital of Shandong University, Jinan City 250022, Shandong Province, China.

[3]Department of Pediatrics, Shandong Provincial Third Hospital, Jinan City 250031, Shandong Province, China.

[#] Xiaoqun Chen and Jiang Qin are co-first authors.

[*]Corresponding Author: Shandong Provincial Third Hospital, No.11 Wuyingshan Middle Road, Tianqiao District, Jinan City, Shandong Province, China. E-mail: duyh3h@163.com.

Abstract

Acute lung injury (ALI) is a pulmonary disorder that leads to acute respiratory failure and thereby results in a high mortality worldwide. Increasing studies have verified that TLR4 is a promoter in ALI, however, the underlying mechanisms of TLR4 was still rarely investigated. In this study, we aimed at exploring the underlying mechanism ofTLR4 in ALI. RT-qPCR identified that TLR4 expression was upregulated in ALI mice and LPS-induced WI-38 cells. Moreover, miR-26a-5p was confirmed to target TLR4 according to luciferase reporter assay. Besides, overexpression of miR-26a-5p decreased the expression levels of proinflammatory factors (TNF-α and IL-1β) and blocked cell apoptosis, while upregulation of TLR4 reversed the effect of miR-26a-5p overexpression. Therefore, miR-26a-5p alleviated lung injury through regulating TLR4. Afterwards, GAS5 was identified to bind with miR-26a-5p by RNA immunoprecipitation (RIP) and luciferase reporter assay. Functionally, GAS5 upregulation accelerated the inflammation injuries and miR-26a-5p overexpression counteracted the effect of GAS5 upregulation on proinflammatory factors and cell apoptosis. In conclusion, GAS5 accelerated ALI through regulating miR-26a-5p/TLR4 axis in ALI mice and LPS-induced cells, which indicates a promising insight into diagnostics and therapeutics in ALI.

Keywords: GAS5, miR-26a-5p, TLR4, acute lung injury

Introduction

Acute lung injury (ALI) is regarded as a severe respiratory dysfunction, characterized by heterogeneous pathologic factors, and finally leads to high morbidity and mortality all over the world (Favarin et al. 2013). The preliminary study has verified that ALI was associated with severe acute inflammatory response (Shin et al. 2017). What's more, various studies have focused on the inflammation of ALI (Ali et al. 2018; Lou et al. 2019; Song et al. 2019a), indicating inflammation is closely related to ALI. Although large numbers of studies have attempted to find a suitable method for ALI therapy, whereas the mortality rate still has not obviously improved. Therefore, to explore the potential mechanism of inflammation is crucial for the clinical therapy of ALI.

Previously, accumulating studies has proved that TLR4 is related to inflammatory response. For example, TLR4 aggravates the inflammation and apoptosis of retinal ganglion cells in high glucose (Hu et al. 2017). In human pancreatic islets, TLR4 can induce inflammatory response (He et al. 2019a). TLR4 silence decreases the inflammation which further prevents the kidney damage and the development of fibrosis in cyclosporine nephrotoxicity (Gonzalez-Guerrero et al. 2017). Although TLR4 was largely investigated in several diseases, the role and mechanisms underlying TLR4 in ALI remain obscure. Thus, in this study, we intended to explore the specific mechanisms of TLR4 in ALI.

MicroRNAs (miRNAs) are a group of short non-coding RNAs with about 22 nucleotides and they can regulate gene expression at post-transcriptional level (Bartel 2004; Machackova et al. 2016). Growing numbers of studies have confirmed that miRNAs could regulate diseases development via targeting specific genes (Bushati and Cohen 2007). For example, miR-126 blocks the development of coronary atherosclerosis in the mice via targeting S1PR2 (Fan et al. 2020). In human cardiac fibroblasts, miR-216a promotes proliferation and fibrogenesis by regulating PTEN and SMAD7 expression (Tao et al. 2019). MiR-38 protects endothelial cell against inflammatory damage in coronary heart disease via targeting CXCR4 (Li et al. 2020). In present study, miR-26a-5p was predicted to target TLR4. Previously, miR-26a-5p was reported to be upregulated in rheumatoid arthritis patients' synovial tissues and elevates the invasive ability of synovial fibroblasts via targeting Smad 1 (Zhang et al. 2018a). MiR-26a-5p negatively regulates the development of neuropathic pain in CCI rat models via targeting MAPK6 (Zhang et al. 2018b). Nevertheless, there was no report about the role of miR-26a-5p in ALI till now. Whether miR-26a-5p targeting TLR4 regulated the development of ALI remains to be elucidated.

In this study, we established animal and cell models of ALI by LPS treatment and we confirmed GAS5 aggravated ALI development through promoting inflammation and cell apoptosis via regulating miR-26a-5p/TLR4 axis, which might offer a promising approach for ALI treatment.

Materials and methods

Acute lung injury mice model

The BALB/c mice (n = 32) were kept in a room maintained at 25℃ with a light/dark cycle of 12 h/12 h, and they were randomly divided into two groups: the Sham group and the ALI group. The ALI mice model was conducted by intratracheally instilling with 10 µg LPS in 50 µL of PBS, and the mice sham group were given an equivalent volume of PBS. Six hours after the infusion of LPS or PBS, the mice were sacrificed, and lung tissues were harvested for RT-qPCR, western blot and HE staining assays. All experimental procedures were approved by the National Institutes of Health Guidelines for the Care and Use of Laboratory Animals.

Measurement of wet/dry ratio of the lungs

10% formalin was used for fixing the lung tissues for one day. Then the tissues were embedded in paraffin, and sliced into 5 µM pieces. Furthermore, the tissue pieces were subjected to haematoxylin-eosin (H&E) staining and observed with a light microscope (Nikon Eclipse TE2000-U, Nikon, Japan). The following standards were used to score the lung injury, no damage or minimal damage = 0; mild damage = 1; moderate damage = 2; severe damage = 3; diffuse injury = 4.

The lung wet/dry (W/D) weight ratio was determined through dividing the wet weight by the dry weight and followed by obtaining the dry weight via incubating at 80℃ for 24 h.

Cell culture and transfection

The Human embryonic lung cells WI-38 and mice lung epithelial TC-1 cell lines were purchased from Cell Bank of Type Culture Collection of Chinese Academy of Sciences (Shanghai, China). Cells were grown in DMEM (Sigma-Aldrich) supplemented with 5% FBS (HyClone, USA), 100 U/mL penicillin and 100 µg/mL streptomycin in a 5% CO2 atmosphere at 37℃. MiR-26a-5p mimics, pcDNA3.1/GAS5, pcDNA3.1/TLR4 and their respective negative control/vector were transfected or co-transfected into WI-38 and TC-1 cells by Lipofectamine 2000 (Invitrogen, USA). After transfection for 48 h, the cells were collected and utilized to explore mRNA and protein expression and cell apoptosis.

Quantitative real-time polymerase chain reaction (RT-qPCR)

Total RNA was extracted from WI-38 and TC-1 cells or tissues with Trizol reagent (Invitrogen; Thermo Fisher Scientific, Inc.). Extracted RNA was reverse-transcribed into complementary DNA (cDNA) by use of a TaqMan MicroRNA Reverse Transcription Kit (Applied Biosystems, Foster City, CA, USA). To quantify GAS5, TLR4, BAX, Bcl-2, IL-1β and TNF-α mRNA, total RNA was reverse-transcribed into cDNAs by use of the Oligo dT primer (TaKaRa). (Amersham Pharmacia Biotech Toronto, ON, Canada). PCR reactions were performed on an ABI 7500 Real-Time PCR System (Applied Biosciences, USA). The expression levels of miR-26a-5p, GAS5, TLR4, BAX, Bcl-2, IL-1β and TNF-α were figured by employing the $2^{-\Delta\Delta Ct}$ method, which were, respectively, standardized to glyceraldehyde 3-phosphate dehydrogenase (GAPDH) and U6. The primers used for RT-qPCR (Bioneer Technology, CA) were as follows:

HBax

qPCR: F : TCATGGGCTGGACATTGGAC,

R : GCGTCCCAAAGTAGGAGAGG;

mBax

qPCR: F : CTGGATCCAAGACCAGGGTG,

R : CTTCCAGATGGTGAGCGAGG;

hBcl-2

qPCR: F : TTTGAGTTCGGTGGGGTCAT,

R : AGAAATCAAACAGAGGCCGCA;

mBcl-2

qPCR: F : AACATCGCCCTGTGGATGAC,

R : TGCACCCAGAGTGATGCAG;

hIL-1β

qPCR: F : TGAGCTCGCCAGTGAAATGA,

R : CATGGCCACAACAACTGACG;

mIL-1β

qPCR: F：TGCCACCTTTTGACAGTGATG,

R：TGATGTGCTGCTGCGAGATT;

hTNF-α

qPCR: F：CTGGGGCCTACAGCTTTGAT,

R：GGCCTAAGGTCCACTTGTGT;

mTNF-α

qPCR: F：ACTGAACTTCGGGGTGATCG,

R：GTTTGCTACGACGTGGGCTA;

hGAPDH

qPCR: F： GCTCTCTGCTCCTCCTGTTC,

R：GACTCCGACCTTCACCTTCC;

mGAPDH

qPCR: F： GGAGAGTGTTTCCTCGTCCC,

R：ATGAAGGGGTCGTTGATGGC;

hTLR4

qPCR: F： GACGGTGATAGCGAGCCAC,

R：TTAGGAACCACCTCCACGCAG;

mTLR4

qPCR: F：CCTGTGGACAAGGTCAGCAA,

R：CTCGGCACTTAGCACTGTCA;

miR-26a-5p: UUCAAGUAAUCCAGGAUAGGCU;

Mock: UCUCCGAACGUGUCACGUU;

Mimic: UUCAAGUAAUCCAGGAUAGGCU;

Inhibitor: AGCCUAUCGAUAUACUUGAA;

qPCR: F：TTCAAGTAATCCAGGA,

R : GTGCAGGGTCCGAGGT;

U6

qPCR: F : CTCGCTTCGGCAGCACA,

R : AACGCTTCACGAATTTGCGT;

hGAS5

qPCR: F : CAGTGTGGCTCTGGATAGCA,

R : TTAAGCTGGTCCAGGCAAGT;

mGAS5

qPCR: F : AGCTGGATAACAGAGCGAGC,

R : GCAAGCCAGCCAAATGAACA.

RNA immunoprecipitation (RIP)

RNA immunoprecipitation assay was conducted by the Magna RIP RNA-Binding Protein Immunoprecipitation Kit (Millipore, USA). Cell lysate was centrifuged for 30 min at 12,000 × *g* and we collected the supernatant. Ago2 antibody (Otwo Biotech, Shenzhen, China) and IgG (Sigma, USA) were respectively cultured with protein G-agarose beads for 2 h at 4°C and then cell lysate supernatant was filled in and cultured overnight at 4°C. RNA was extracted from magnetic beads using TRIzol reagent (Invitrogen) and RT-qPCR was used to detected GAS5 and miR-26a-5p in the precipitates. The IgG antibody group as control.

Western blot analysis

Tissues and cells were collected and lysed in protein lysis buffer (Bio-Rad Laboratories). Then equal amount of proteins samples was separated on SDS-12% PAGE and transferred to polyvinylidene difluoride (PVDF) membranes. Blocked by 5% skimmed milk for 1 h at indoor temperature, and incubated with the primary antibodies against TLR4 (ab22048; Abcam, UK) Bax (ab32503; Abcam), Bcl-2 (ab32124; Abcam) and GAPDH (ab181602; Abcam) were incubated at 4°C for 24h. The membranes were washed by utilizing TBS and then cultured with HRP-conjugated secondary antibodies at room temperature for 1 h. At last, the protein bands were assessed via an ECL kit (Amersham Biosciences, UK) and the intensity was analyzed by employing ImageJ software.

Luciferase reporter assay

The luciferase reporter vectors of wild type TLR4 (pmirGLO-TLR4 -WT) or wild type GAS5 (pmirGLO-GAS5-WT) containing the potential targeted sites of miR-26a-5p and mutant-type TLR4 (or pmirGLO-TLR4 -Mut) or mutant type GAS5 (pmirGLO-GAS5-Mut) were established. Afterwards, TC-1 and WI-38 cells were transfected with the reporter vectors together with miR-26a-5p, anti-miR-26a-5p or Mock by use of Lipofectamine 2000 (Invitrogen). After 48 h, the relative luciferase activities were detected by using luciferase reporter assay system (Promega, USA).

Apoptosis by flow cytometry assay

The apoptosis rate of TC-1 and WI-38 cells was evaluated through Annexin Vfluorescein isothiocyanate/propidium iodide (Annexin V-FITC/PI) apoptosis assay kit (Invitrogen) according to previous procedures (Rieger et al. 2011). In short, TC-1 and WI-38 cells treated with LPS after transfection and then subjected to staining with Annexin V-FITC and PI for 25 min in the dark. Afterwards, the apoptotic cells were investigated by flow cytometry (FACS 420, BD Biosciences, USA). Percentage of apoptosis rate (%) = (number of apoptotic cells/ number of all cells) × 100%.

ELISA

ELISA was performed by applying the ELISA kits for tumor necrosis factor α (TNF-α) and interleukin 1β (IL-1β) (Abcam Biotechnology, Cambridge, MA, USA) in order to detect the concentration of TNF-α and IL-1β in culture supernatant of TC-1 and WI-38 cells, Absorbance was determined via a microplate reader at 450 nm.

Statistical analysis

Data were expressed as means± SD. All experiments were repeated three times. The differences between or among groups were evaluated by Student's test or one-way analysis of variance (ANOVA). *P* value less than 0.05 was considered significant.

Results

TLR4 was upregulated in ALI mice and LPS-induced cells

To investigate the potential role of TLR4 in lung injury, the ALI mice model was built by intratracheally instilling with LPS. Microscopic observation indicated that the alveolar structure in the lung from normal mice was intact without thickening or lymphocyte infiltration (left panel, Figure 1A). Nevertheless, pulmonary lesions in ALI mice were obvious, which presented with pathologically

thickened alveolar walls, collapsed alveoli and plenty of infiltrated red blood cells and inflammatory cells (right panel, Figure 1A), suggesting that our ALI mice model was successfully established. Then RT-qPCR and western blot were conducted to investigate the mRNA and protein expression of TLR4, respectively. The data revealed that TLR4 was prominently upregulated in ALI mice and WI-38 cells (Figure 1B-D). The results above indicated that TLR4 was highly expressed in ALI mice and LPS-induced WI-38 cells.

TLR4 was a direct target of miR-26a-5p

It is reported that miRNAs widely participate in the regulation of biological processes through binding with the 3' untranslated region of the target mRNA(s) (Bushati and Cohen 2007). In order to find out the potential miRNAs might bind with TLR4, we searched RNA22v2 database online websites and found the underlying binding sites between miR-26a-5p and TLR4 of human and mouse (Figure 2A). Next, miR-26a-5p expression was increased by transfection with miR-26a-5p mimics and reduced by transfection with anti-miR-26a-5p in WI-38 and TC-1 cells according to RT-qPCR analysis (Figure 2B). To verify the relationship between miR-26a-5p and TLR4, luciferase assay was then carried out. The result disclosed that luciferase activity of pmirGLO-TLR4-WT was obviously decreased by the introduction of miR-26a-5p mimics but increased by the introduction of miR-26a-5p inhibitor, while no distinct change was detected in pmirGLO-TLR4-Mut group (Figure 2C-D). These findings suggested that miR-26a-5p could bind with TLR4. To further investigate the interaction between miR-26a-5p and TLR4, we conducted the following experiments and discovered that the mRNA and protein expression of TLR4 were declined in the miR-26a-5p mimics group but increased in miR-26a-5p inhibitor group after lung injury (Figure 2E-F). Moreover, miR-26a-5p was remarkably downregulated in ALI mice and WI-38 cell (Figure 2G-H). To sum up, miR-26a-5p could directly bind with TLR4.

MiR-26a-5p overexpression inhibited the production of inflammatory factors via targeting TLR4

Then, we investigated whether the interaction of miR-26a-5p and TLR4 could influence lung injury and inflammation. To begin with, TLR4 expression was effectively increased by adeno-associated virus injection (Figure 3A). H&E staining showed the lung injury was more serious in ALI mice model, miR-26a-5p upregulation alleviated the lung injury by ALI, while this effect was reversed by TLR4 upregulation (Figure 3B-C). Moreover, we found that miR-26a-5p overexpression reduced the lung drying wet ratio, but upregulation of TLR4 abolished the effects (Figure 3C). Furthermore, miR-26a-5p overexpression downregulated the levels of TNF-α and IL-1β, whereas TLR4 upregulation

counteracted the effects in ALI mice, WI-38 and TC-1 cells (Figure 3E-G). Collectively, we draw a conclusion that miR-26a-5p overexpression alleviated lung injury and inhibited production of inflammatory factors via targeting TRL4.

MiR-26a-5p overexpression reduced cell apoptosis by targeting TRL4

To probe whether the interaction of miR-26a-5p and TLR4 was involved in cell apoptosis in ALI, we detected cell apoptosis by flow cytometry assay and the levels of Bax, Bcl-2 and caspase-3. As found in the results, miR-26a-5p overexpression decreased the expression of Bax but increased the expression of Bcl-2 in ALI mice while TLR4 upregulation reversed the effects. (Figure 4A). What's more, the decreased activity of caspase-3 in ALI mice by elevation of miR-26a-5p was reversed by TLR4 overexpression (Figure 4B). Furthermore, miR-26a-5p overexpression reduced cell apoptosis but TLR4 upregulation counteracted the effect in TC-1 and WI-38 cells (Figure 4C). Besides, the level of Bax was increased while the level of Bcl-2 was downregulated by overexpression of miR-26a-5p in TC-1 and WI-38 cells, while TRL4 upreguation inversely changed this effect (Figure 4D-E). As presented in Figure 4F, miR-26a-5p overexpression decreased the caspase-3 activity in TC-1 and WI-38 cells, and TLR4 upregulation abolished the effect. Collectively, above findings indicated that miR-26a-5p overexpression could reduce cell apoptosis through targeting TLR4.

GAS5 could bind with miR-26a-5p in ALI

As some reports show that lncRNAs can function as a sponge of miRNAs to regulate the development of diseases (Fan et al. 2019; Ouyang et al. 2019; Yu et al. 2019). We suspected there might be such an lncRNA that could bind with miR-26a-5p to regulate the progression of ALI. We used DIANA tools and found the potential binding sites between miR-26a-5p and GAS5 of human and mouse (Figure 5A). To verify the relationship between miR-26a-5p and GAS5, luciferase reporter and RIP assay were conducted in WI-38 and TC-1 cells. Luciferase reporter assay suggested that luciferase activity of pmirGLO-GAS5-WT was prominently decreased in miR-26a-5p transfected cells but increased in anti-miR-26a-5p transfected cells, while no significant change was detected in pGLO-GAS5-Mut group (Figure 5B-C). RIP assay indicated that GAS5 and miR-26a-5p were enriched in Ago2 groups but not in IgG groups (Figure 5D-E). All these results proved that GAS5 could bind with miR-26a-5p. Then, we identified that miR-26a-5p overexpression led to a prominent decline of GAS5 expression, and miR-26a-5p downregulation resulted in an increase of GAS5 expression (Figure 5F), suggesting that GAS5 could negatively regulated by miR-26a-5p. Thereafter, the data from RT-qPCR discovered

GAS5 was upregulated in ALI mice and WI-38 cells (Figure 5G-H). To sum up, GAS5 could bind with miR-26a-5p in ALI.

GAS5 accelerated the production of inflammatory factors by sponging miR-26a-5p

To determine whether GAS5/miR-26a-5p axis could exert function in ALI, rescue assays were conducted. H&E staining showed miR-26a-5p upregulation alleviated GAS5 overexpression mediated the lung injury in ALI mice (Figure 6A-B). The lung drying wet ratio was increased after GAS5 upregulation, while miR-26a-5p overexpression abrogated the effect (Figure 6C). Moreover, GAS5 upregulation enhanced the mRNA levels of TNF-α and IL-1β, whereas miR-26a-5p overexpression significantly abrogated the effects in ALI mice, WI-38 and TC-1 cells (Figure 6D-F). These results suggested that GAS5 accelerated the production of inflammatory factor by sponging miR-26a-5p.

GAS5 facilitated cell apoptosis via sponging miR-26a-5p

Furthermore, we evaluated whether the GAS5/miR-26a-5p axis affected the apoptosis of TC-1 and WI-38 cells. We discovered that miR-26a-5p overexpression reversed the effect of GAS5 upregulation on the level of Bax and Bcl-2 (Figure 7A). Afterwards, we carried out ELISA to investigate the activity of caspase-3 in mice, disclosing miR-26a-5p overexpression abolished the promotive effect of GAS5 upregulation on caspase-3 activity (Figure 7B). Additionally, miR-26a-5p overexpression counteracted the aggravated effect of GAS5 upregulation on cell apoptosis in ALI mice (Figure 7C). The level of Bax was increased while the level of Bcl-2 was decreased by GAS5 overexpression in TC-1 and WI-38 cells, but recovered by the upregulation of miR-26a-5p (Figure 7D-E). As presented in Figure 7F, the caspase-3 activity was increased after LPS treatment in TC-1 and WI-38 cells while GAS5 upregulation further increased it but the effect was abolished by miR-26a-5p overexpression. In conclusion, all the findings indicated that GAS5 promoted cell apoptosis via regulating miR-26a-5p.

Discussion

ALI is a severe illness that threats health and lives worldwide because of the high incidence and mortality (Ding et al. 2016). To uncover biomarkers that are more reliable for it is of great significance. Recent studies have widely showed that miRNAs play an essential regulatory role in the progression of ALI. For example, miR-124 alleviates the effect of ALI via suppression of the mitogen-activated protein kinase (MAPK) signaling pathway activation by targeting MAPK14 (Pan et al. 2019). Staphylococcal enterotoxin B-induced microRNA-155 targets SOCS1 to accelerate the acute

inflammatory lung injury (Rao et al. 2014). MiR-21-5p modulates type II alveolar epithelial cell apoptosis in hyperoxic ALI (Qin et al. 2018). However, whether miR-26a-5p exerted function in ALI remains to be elucidated. In present study, TLR4 was identified to be a direct target of miR-26a-5p in TC-1 and WI-38 cells. What's more, miR-26a-5p negatively regulated TLR4. More importantly, miR-26a-5p overexpression inhibited the production of inflammatory factors and reduced cell apoptosis via targeting TLR4. In summary, miR-26a-5p could regulate the development of ALI via targeting TLR4.

Unlike miRNAs, the long noncoding RNAs (lncRNAs) are a group of noncoding RNAs with longer than 200 nucleotides that participate in many biological and physiological processes (Chi et al. 2019). It is widely accepted that lncRNAs are able to act as miRNA "sponges" to compete with mRNAs for miRNAs with shared miRNAs responses elements (MREs) and can regulate miRNAs (Sen et al. 2014). What's more, it is reported that ceRNAs are widely implicated in many biological processes. For example, lncRNA LINC00339 accelerates renal tubular epithelial pyroptosis via modulating the miR-22-3p/NLRP3 axis in calcium oxalate-induced kidney stone (Song et al. 2019b). LncRNA DSCAM-AS1 accelerates breast cancer cell proliferation and suppresses breast cancer cell apoptosis via sponging miR-204-5p and upregulating RRM2 expression (Liang and Li 2019). LncRNA LINC00460 accelerates the progression of head and neck squamous cell carcinoma via sponging miR-612 to up-regulate AKT2 (Xie et al. 2019). In our study, we confirmed that GAS5 could bind with miR-26a-5p by using RNA immunoprecipitation (RIP) and luciferase reporter assay. Previously, a report corroborated that GAS5 suppresses cell proliferation and fibrosis in diabetic nephropathy via the regulation of miR-221/SIRT1 axis (Ge et al. 2019). GAS5 aggravated the progression of atherosclerosis by inhibiting EZH2-mediated ABCA1 transcription in ApoE Mice (Meng et al. 2019). GAS5 triggers the formation of abdominal aortic aneurysm via enhancing the apoptosis of smooth muscle (He et al. 2019b). However, the exact role as well as the regulatory function of GAS5 in ALI was rarely investigated. In current study, we identified that miR-26a-5p negatively regulated the expression of GAS5. Additionally, GAS5 upregulation increased the lung injury scores, the lung wet/dry weight ratio, the levels of proinflammatory factors and apoptosis in ALI, miR-26a-5p elevation counteracted these effects.

In summary, our results proved that GAS5 aggravated ALI through promoting inflammation and cell apoptosis by regulating miR-26a-5p/TLR4 axis, which may provide new insights into the therapeutic strategy.

Author contribution

XC, QJ and YD conceived and designed research. XC and QJ conducted experiments. XC and QJ analyzed data. XC and QJ wrote the manuscript. All authors read and approved the manuscript.

Compliance with ethical standards

All experimental procedures were approved by the National Institutes of Health Guidelines for the Care and Use of Laboratory Animals. All procedures performed in studies involving human participants were in accordance with the ethical standards of the institutional and national research committee and with the 1964 Helsinki declaration and its later amendments or comparable ethical standards.

Conflict of interest

The authors declare that there is no conflict of interest in this study.

Acknowledgement

We thank all participators for their help.

References

Ali FF, Abdel-Hamid HA, Toni ND (2018) H2S attenuates acute lung inflammation induced by administration of lipopolysaccharide in adult male rats General physiology and biophysics doi:10.4149/gpb_2018002

Bartel DP (2004) MicroRNAs: genomics, biogenesis, mechanism, and function Cell 116:281-297 doi:10.1016/s0092-8674(04)00045-5

Bushati N, Cohen SM (2007) microRNA functions Annual review of cell and developmental biology 23:175-205 doi:10.1146/annurev.cellbio.23.090506.123406

Chi Y, Wang D, Wang J, Yu W, Yang J (2019) Long Non-Coding RNA in the Pathogenesis of Cancers

Cells 8 doi:10.3390/cells8091015

Ding XM, Pan L, Wang Y, Xu QZ (2016) Baicalin exerts protective effects against lipopolysaccharide-induced acute lung injury by regulating the crosstalk between the CX3CL1-CX3CR1 axis and NF-kappaB pathway in CX3CL1-knockout mice International journal of molecular medicine 37:703-715 doi:10.3892/ijmm.2016.2456

Fan J, Zhang J, Huang S, Li P (2019) lncRNA OSER1-AS1 acts as a ceRNA to promote tumorigenesis in hepatocellular carcinoma by regulating miR-372-3p/Rab23 axis Biochemical and biophysical research communications doi:10.1016/j.bbrc.2019.10.105

Fan JL, Zhang L, Bo XH (2020) MiR-126 on mice with coronary artery disease by targeting S1PR2 European review for medical and pharmacological sciences 24:893-904 doi:10.26355/eurrev_202001_20074

Favarin DC, de Oliveira JR, de Oliveira CJ, Rogerio Ade P (2013) Potential effects of medicinal plants and secondary metabolites on acute lung injury BioMed research international 2013:576479 doi:10.1155/2013/576479

Ge X et al. (2019) Long noncoding RNA GAS5 inhibits cell proliferation and fibrosis in diabetic nephropathy by sponging miR-221 and modulating SIRT1 expression Aging 11:8745-8759 doi:10.18632/aging.102249

Gonzalez-Guerrero C, Cannata-Ortiz P, Guerri C, Egido J, Ortiz A, Ramos AM (2017) TLR4-mediated inflammation is a key pathogenic event leading to kidney damage and fibrosis in cyclosporine nephrotoxicity Archives of toxicology 91:1925-1939 doi:10.1007/s00204-016-1830-8

He W, Rebello O, Savino R, Terracciano R, Schuster-Klein C, Guardiola B, Maedler K (2019a) TLR4 triggered complex inflammation in human pancreatic islets Biochimica et biophysica acta Molecular basis of disease 1865:86-97 doi:10.1016/j.bbadis.2018.09.030

He X et al. (2019b) Long noncoding RNA GAS5 induces abdominal aortic aneurysm formation by promoting smooth muscle apoptosis Theranostics 9:5558-5576 doi:10.7150/thno.34463

Hu L, Yang H, Ai M (2017) Inhibition of TLR4 alleviates the inflammation and apoptosis of retinal ganglion cells in high glucose 255:2199-2210 doi:10.1007/s00417-017-3772-0

Li Y, Huang J, Yan H, Li X, Ding C, Wang Q, Lu Z (2020) Protective effect of microRNA381 against inflammatory damage of endothelial cells during coronary heart disease by targeting CXCR4 Molecular medicine reports doi:10.3892/mmr.2020.10957

Liang WH, Li N (2019) DSCAM-AS1 promotes tumor growth of breast cancer by reducing miR-204-5p and up-regulating RRM2 58:461-473 doi:10.1002/mc.22941

Lou J et al. (2019) Endothelial cell-specific anticoagulation reduces inflammation in a mouse model of acute lung injury Acta pharmacologica Sinica 40:769-780 doi:10.1038/s41401-018-0175-7

Machackova T et al. (2016) MiR-429 is linked to metastasis and poor prognosis in renal cell carcinoma by affecting epithelial-mesenchymal transition Tumour biology : the journal of the International Society for Oncodevelopmental Biology and Medicine 37:14653-14658 doi:10.1007/s13277-016-5310-9

Meng XD, Yao HH, Wang LM, Yu M, Shi S, Yuan ZX, Liu J (2019) Knockdown of GAS5 Inhibits Atherosclerosis Progression via Reducing EZH2-Mediated ABCA1 Transcription in ApoE(-/-) Mice Mol Ther Nucleic Acids 19:84-96 doi:10.1016/j.omtn.2019.10.034

Ouyang T, Zhang Y, Tang S, Wang Y (2019) Long non-coding RNA LINC00052 regulates miR-608/EGFR axis to promote progression of head and neck squamous cell carcinoma Experimental and molecular pathology:104321 doi:10.1016/j.yexmp.2019.104321

Pan W, Wei N, Xu W, Wang G, Gong F, Li N (2019) MicroRNA-124 alleviates the lung injury in mice with septic shock through inhibiting the activation of the MAPK signaling pathway by downregulating MAPK14 International immunopharmacology 76:105835 doi:10.1016/j.intimp.2019.105835

Qin S, Chen M, Ji H, Liu GY, Mei H, Li K, Chen T (2018) miR215p regulates type II alveolar epithelial cell apoptosis in hyperoxic acute lung injury Molecular medicine reports 17:5796-5804 doi:10.3892/mmr.2018.8560

Rao R, Rieder SA, Nagarkatti P, Nagarkatti M (2014) Staphylococcal enterotoxin B-induced microRNA-155 targets SOCS1 to promote acute inflammatory lung injury Infection and immunity 82:2971-2979 doi:10.1128/iai.01666-14

Rieger AM, Nelson KL, Konowalchuk JD, Barreda DR (2011) Modified annexin V/propidium iodide apoptosis assay for accurate assessment of cell death Journal of visualized experiments : JoVE doi:10.3791/2597

Sen R, Ghosal S, Das S, Balti S, Chakrabarti J (2014) Competing endogenous RNA: the key to posttranscriptional regulation TheScientificWorldJournal 2014:896206 doi:10.1155/2014/896206

Shin NR et al. (2017) Artemisia argyi attenuates airway inflammation in lipopolysaccharide induced acute lung injury model Laboratory animal research 33:209-215 doi:10.5625/lar.2017.33.3.209

Song C et al. (2019a) NETs promote ALI/ARDS inflammation by regulating alveolar macrophage polarization Experimental cell research 382:111486 doi:10.1016/j.yexcr.2019.06.031

Song Z, Zhang Y, Gong B, Xu H, Hao Z, Liang C (2019b) Long noncoding RNA LINC00339 promotes renal tubular epithelial pyroptosis by regulating the miR-22-3p/NLRP3 axis in calcium oxalate-induced kidney stone 120:10452-10462 doi:10.1002/jcb.28330

Tao J et al. (2019) MiR-216a accelerates proliferation and fibrogenesis via targeting PTEN and SMAD7 in human cardiac fibroblasts Cardiovascular diagnosis and therapy 9:535-544 doi:10.21037/cdt.2019.11.06

Xie X, Xiong G, Wang Q, Ge Y, Cui X (2019) Long non-coding RNA LINC00460 promotes head and neck squamous cell carcinoma cell progression by sponging miR-612 to up-regulate AKT2 American journal of translational research 11:6326-6340

Yu PF, Wang Y, Lv W, Kou D, Hu HL, Guo SS, Zhao YJ (2019) LncRNA NEAT1/miR-1224/KLF3 contributes to cell proliferation, apoptosis and invasion in lung cancer European review for medical and pharmacological sciences 23:8403-8410 doi:10.26355/eurrev_201910_19151

Zhang W, Chen L, Jiang Y, Shen Y (2018a) miR-26a-5p Regulates Synovial Fibroblast Invasion in Patients with Rheumatoid Arthritis by Targeting Smad 1 Medical science monitor : international medical journal of experimental and clinical research 24:5178-5184 doi:10.12659/msm.907816

Zhang Y, Su Z, Liu HL, Li L, Wei M, Ge DJ, Zhang ZJ (2018b) Effects of miR-26a-5p on neuropathic pain development by targeting MAPK6 in in CCI rat models Biomedicine & pharmacotherapy = Biomedecine & pharmacotherapie 107:644-649 doi:10.1016/j.biopha.2018.08.005

Figure legends

Figure 1 TLR4 was upregulated in ALI mice and LPS-induced cells. A, H&E staining was conducted to assess the lung injury degree in ALI mice. B-D, The mRNA and protein expression of TLR4 in ALI mice and LPS-induced cells were respectively proved by RT-qPCR and western blot assay. #P< 0.05 compared with Sham group in B; #P< 0.05 compared with control group in C.

Figure 2 TLR4 could bind with miR-26a-5p. A, RNA22v2 database predicted the target of miR-26a-5p on TLR4. B, RT-qPCR assay was conducted to evaluate the efficiency of miR-26a-5p overexpression and miR-26a-5p knockdown in LPS-induced cells. C-D, Luciferase reporter assay was performed to verify the interaction between miR-26a-5p and TLR4. E-F, RT-qPCR and western blot assay were performed to detect the effect of miR-26a-5p overexpression and miR-26a-5p knockdown to the mRNA and protein expression of TLR4 in WI-38 and TC-1 cells. G-H The expression of miR-26a-5p in ALI mice and LPS-treated WI-38 cells was assessed by RT-qPCR assay. $^{\#}P< 0.05$ compared with Mock group in B, C, D, E; $^{\#}P< 0.05$ compared with Sham group in G; $^{\#}P< 0.05$ compared with control group in H.

Figure 3 MiR-26a-5p inhibited the progression of lung injury by regulating TLR4. A, The expression of TLR4 was evaluated by RT-qPCR. B-C, H&E staining was conducted to assess the lung injury degree. D, Statistical analysis detected the lung wet/dry weight ratio. E-G, RT-qPCR was used to detect the levels of TNF-α and IL-1β in ALI mice as well as TC-1 and WI-38 cells. $^{\#}P< 0.05$ compared with AAV-vector group in A; $^{\#}P< 0.05$ compared with Sham + AAV-Mock + AAV-vector group, $^{\&}P< 0.05$ compared with ALI + AAV-Mock + AAV-vector group, $^{@}P< 0.05$ compared with ALI + AAV-miR-26a-5p + AAV-vector group in C, D, E; $^{\#}P< 0.05$ compared with control + Mock + vector group, $^{\&}P< 0.05$ compared with LPS + Mock + vector group, $^{@}P< 0.05$ compared with LPS + miR-26a-5p + vector group in F, G.

Figure 4 MiR-26a-5p overexpression alleviated cell apoptosis by modulating TLR4. A, RT-qPCR was conducted to detect the expression of Bax and Bcl-2 in ALI mice. B, ELISA was used to determine the activity of caspase-3 in ALI mice. C, Flow cytometry assay was utilized to verify the percentage of cell apoptosis. D-E, the expression of Bax and Bcl-2 in cells was calculated by RT-qPCR. F, ELISA was utilized to detect the activity of caspase-3 in cells. $^{\#}P< 0.05$ compared with Sham + AAV-Mock + AAV-vector group, $^{\&}P< 0.05$ compared with ALI + AAV-Mock + AAV-vector group, $^{@}P< 0.05$ compared with ALI + AAV-miR-26a-5p + AAV-vector group in A, B, C; $^{\#}P< 0.05$ compared with control + Mock + vector group, $^{\&}P< 0.05$ compared with LPS + Mock + vector group, $^{@}P< 0.05$ compared with LPS + miR-26a-5p + vector group in D, E, F.

Figure 5 GAS5 regulated miR-26a-5p in ALI. A, The predicted biding sites of miR-26a-5p on GAS5 were detected by DIANA tools. B-E, The interaction between miR-26a-5p and GAS5 was demonstrated by luciferase reporter and RIP assays. F, The efficiency of miR-26a-5p overexpression

and miR-26a-5p knockdown on GAS5 expression were estimated by RT-qPCR. G-H, RT-qPCR assay was conducted to evaluate the level of GAS5 in ALI mice and LPS-induced cells. $^{\#}$P< 0.05 compared with Mock group in B, C, F; $^{\#}$P< 0.05 compared with anti-IgG group in D, E; $^{\#}$P< 0.05 compared with Sham group in G; $^{\#}$P< 0.05 compared with control group in H.

Figure 6 GAS5 promoted the inflammation injuries by targeting miR-26a-5p. A-B, H&E staining was carried out to detect the lung injury degree in ALI. C, Statistical analysis detected the lung wet/dry weight ratio. D-F, RT-qPCR was used to detect the levels of TNF-α and IL-1β. $^{\#}$P< 0.05 compared with Sham + AAV-Mock + AAV-vector group, $^{\&}$P< 0.05 compared with ALI + AAV-Mock + AAV-vector group, $^{@}$ P< 0.05 compared with ALI + AAV-miR-26a-5p + AAV-vector group in C, D; $^{\#}$P< 0.05 compared with control + Mock + vector group, $^{\&}$P< 0.05 compared with LPS + Mock + vector group, $^{@}$ P< 0.05 compared with LPS + miR-26a-5p + vector group in E, F.

Figure 7 GAS5 accelerated cell apoptosis via regulating miR-26a-5p. A, The protein expression of apoptosis genes in ALI mice was assessed by western blot assay. B, ELISA was used to detect the activity of caspase-3 in ALI mice. C, Flow cytometry assay was utilized to verify the percentage of cell apoptosis. D-E, The protein expression of apoptosis genes in cells was calculated by western blot assay. F, The activity of caspase-3 in cells was detected by ELISA. $^{\#}$P< 0.05 compared with Sham + AAV-Mock + AAV-vector group, $^{\&}$P< 0.05 compared with ALI + AAV-Mock + AAV-vector group, $^{@}$ P< 0.05 compared with ALI + AAV-miR-26a-5p + AAV-vector group in A, B; $^{\#}$P< 0.05 compared with control + Mock + vector group, $^{\&}$P< 0.05 compared with LPS + Mock + vector group, $^{@}$ P< 0.05 compared with LPS + miR-26a-5p + vector group in C, D.

Figure 1 TLR4 was upregulated in ALI mice and LPS-induced cells.

Click here to access/download;Figure;Figure 1.tif

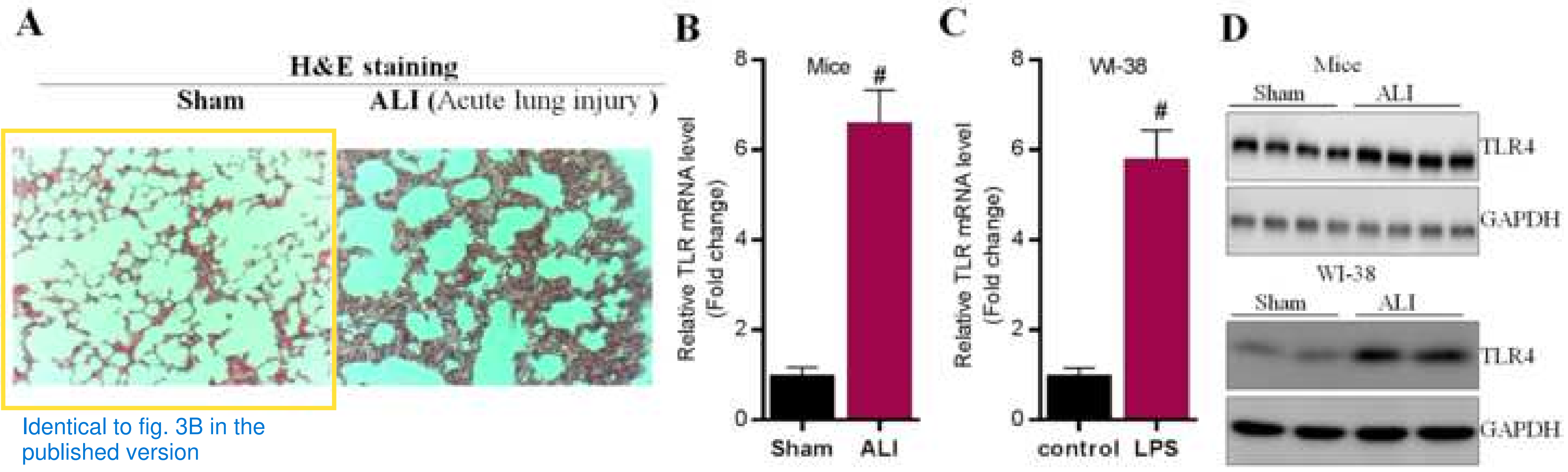

Identical to fig. 3B in the published version

Figure 2 TLR4 could bind with miR-26a-5p.

Click here to access/download;Figure;Figure 2.tif

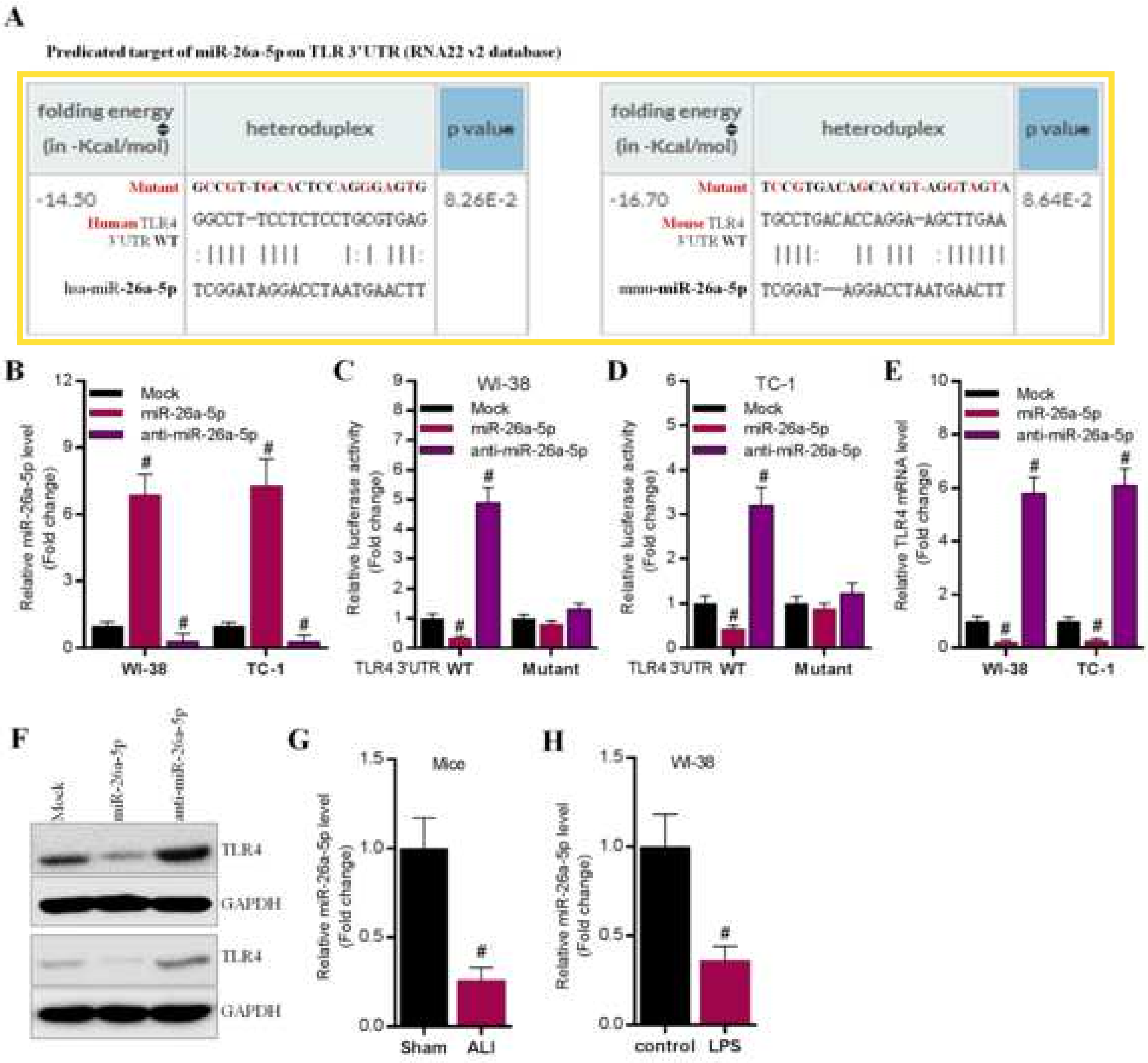

Figure 3 MiR-26a-5p inhibited the progression of lung injury by regulating TLR4.

Click here to access/download;Figure;Figure 3.tif

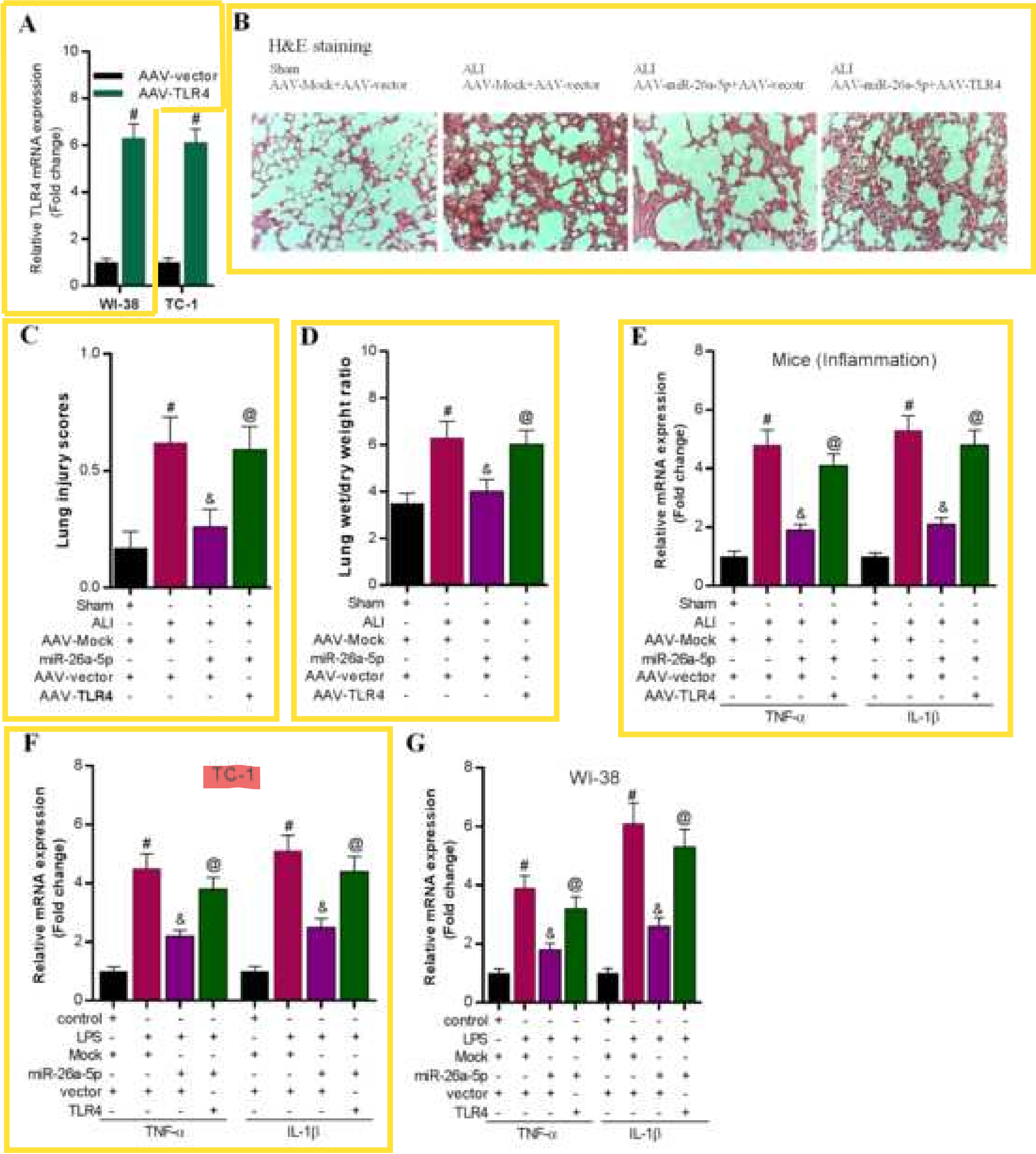

very similar to fig.3G, differences are marked in red

Figure 4 MiR-26a-5p overexpression alleviated cell apoptosis by modulating TLR4.

Click here to access/download;Figure;Figure 4.tif

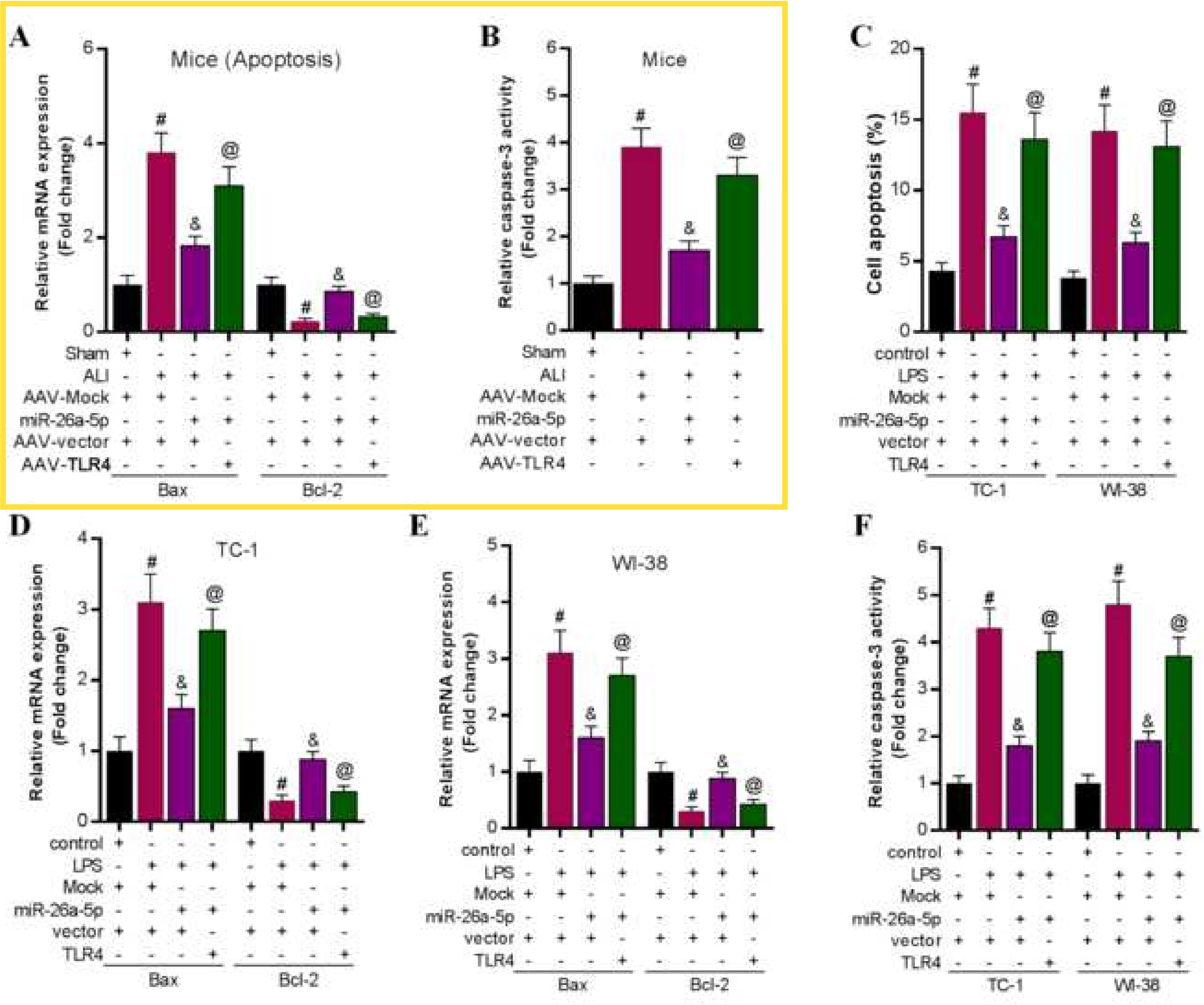

Figure 5 GAS5 regulated miR-26a-5p in ALI.

Click here to access/download;Figure;Figure 5.tif

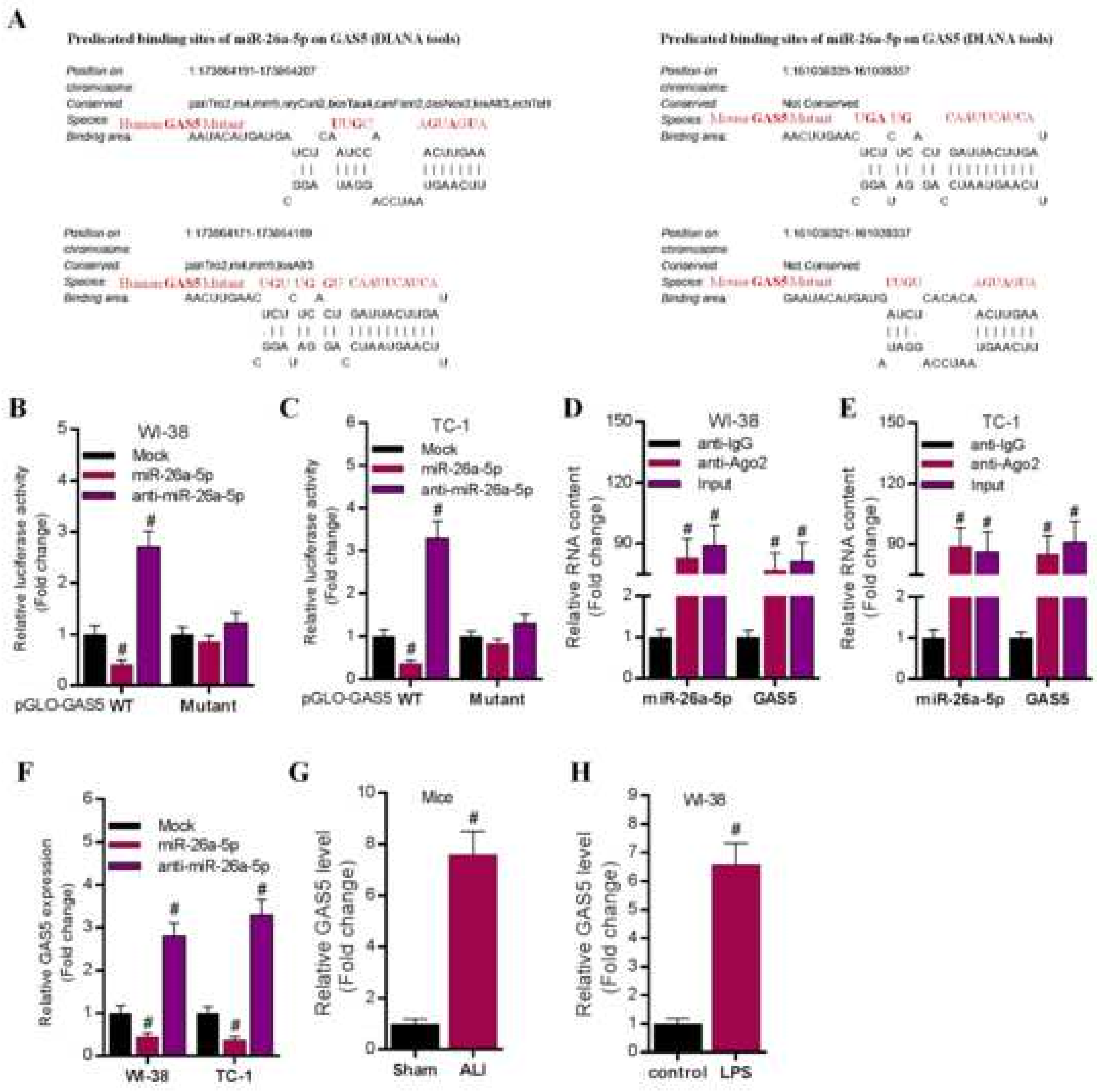

Figure 6 GAS5 promoted the inflammation injuries by targeting miR-26a-5p.

Click here to access/download;Figure;Figure 6.tif

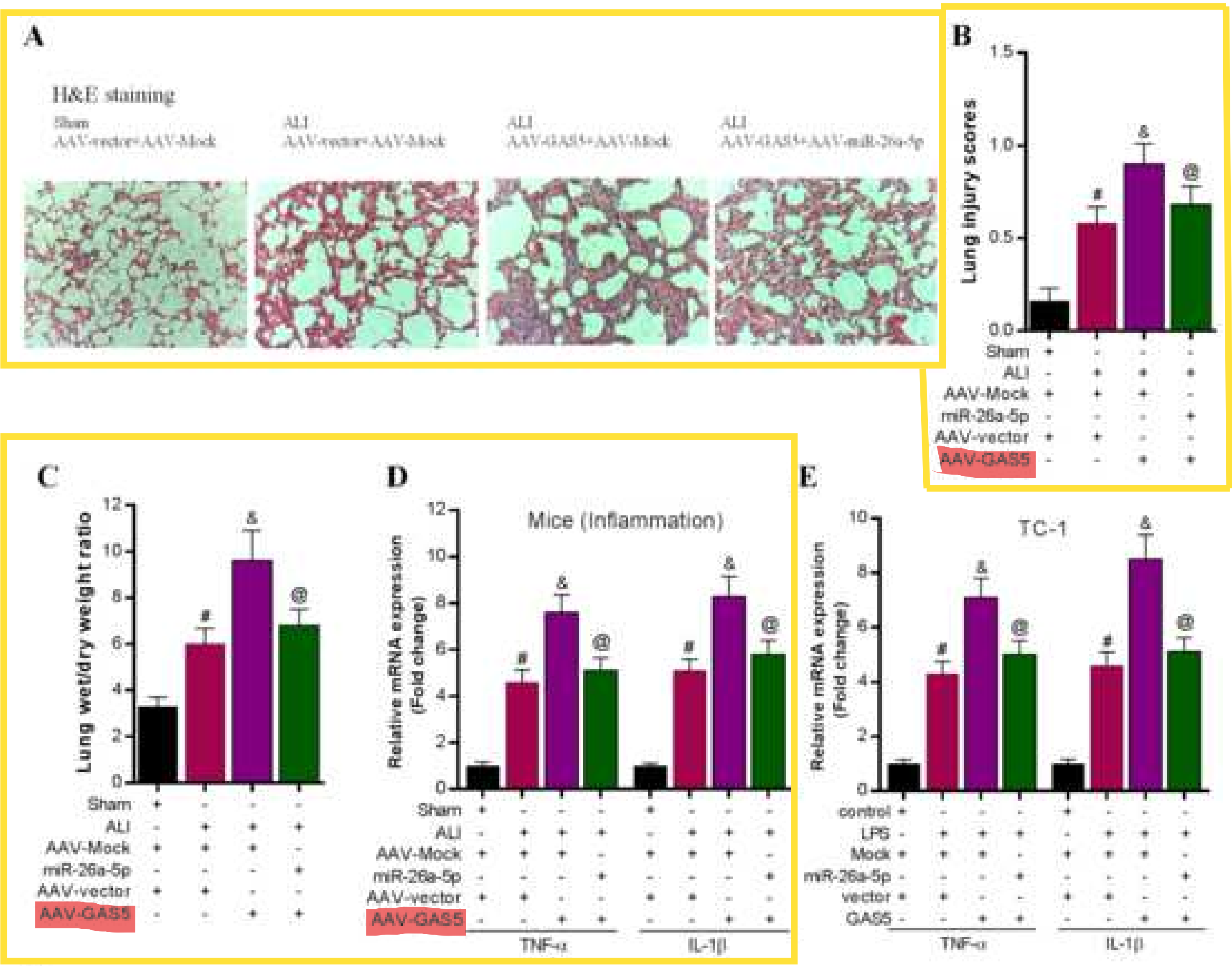

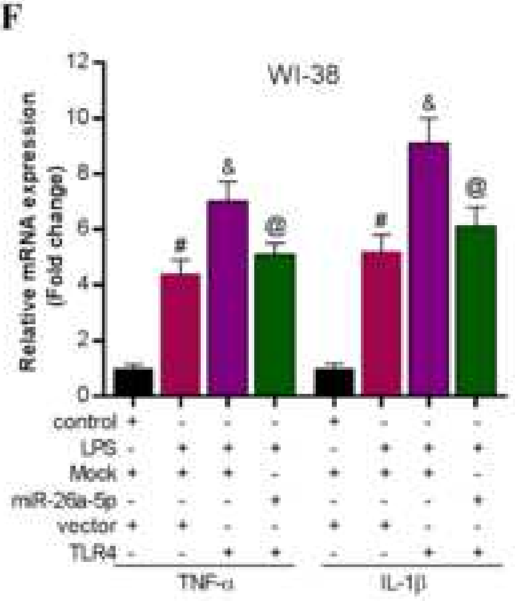

B, C and D are only almost identical to the published version, the differences are marked in red

Figure 7 GAS5 accelerated cell apoptosis via regulating miR-26a-5p.

Click here to access/download;Figure;Figure 7.tif

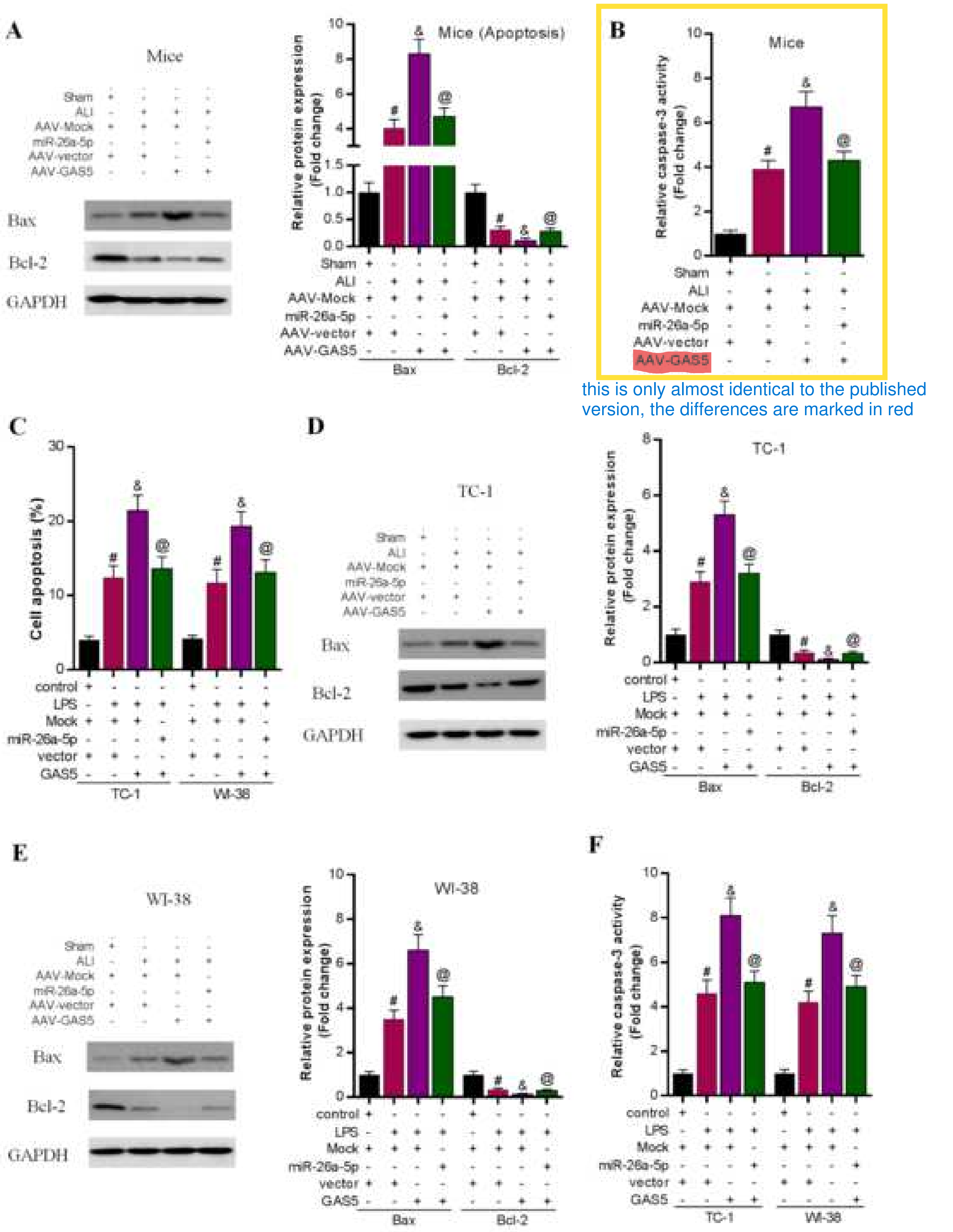

Supplement: Supplementary file 3 — Supplementary file3 (PDF 966 KB) [file 210_2023_2741_MOESM3_ESM.pdf]
