# Supplementary material for: Fake paper identification in the pool of withdrawn and rejected manuscripts submitted to Naunyn–Schmiedeberg’s Archives of Pharmacology
Source: Naunyn Schmiedebergs Arch Pharmacol. 2023 Oct 5;397(4):2171–81. doi: 10.1007/s00210-023-02741-w (PMC10933159; doi:10.1007/s00210-023-02741-w)

Figure S4

Color coding:

Yellow highlighted text	The text is identical in the NSAP version and the published version of this paper.
Red highlighted text	There are differences in the text between the NSAP version and the published version of this paper (different content or different wording).
Yellow bordered figure	This figure is identical in both versions of this paper.

// Naunyn-Schmiedeberg's Archives of Pharmacology

Therapeutic effect of N-Acetyl-Seryl-Aspartyl-Proline and Vasoactive Intestinal Peptide on COPD pathophysiology

--Manuscript Draft--

Manuscript Number:	NSAP-D-21-00369
Full Title:	Therapeutic effect of N-Acetyl-Seryl-Aspartyl-Proline and Vasoactive Intestinal Peptide on COPD pathophysiology
Article Type:	Original Article
Corresponding Author:	Zongxiu Yin<br>Shandong Provincial Hospital<br>CHINA
Corresponding Author Secondary Information:	
Corresponding Author's Institution:	Shandong Provincial Hospital
Corresponding Author's Secondary Institution:	
First Author:	Yanna Yang
First Author Secondary Information:	
Order of Authors:	Yanna Yang
	Ronghua Zhang
	Wei Zhu
	Zongxiu Yin
Order of Authors Secondary Information:	
Funding Information:	
Abstract:	COPD (chronic obstructive pulmonary disease) as an inflammatory respiratory system disease is caused by exposure to cigarette smoke and tobacco in long-term. Anti-inflammatory peptides can control inflammatory response of COPD. Ac-SDKP and VIP as peptide have anti-inflammatory effect and, in this study, the effect of Ac-SDKP and VIP on COPD inflammation was studied.<br>After producing cigarette smoke induced COPD mice model, which was treated with VIP and Ac-SDKP. The levels Antioxidant related factors, (MDA and SOD), Fibrotic factors (HP and TGF-β), pro-inflammatory cytokines (TNF-α, IL-1β and IL-6), Signaling Gene Expression (NF-κB and IκB-α) and inflammation in histopathological examination were studied.<br>MDA, Remodeling factors, pro-inflammatory cytokines, NF-κB and IκB-α gene expression and inflammation in lung tissue were controlled by VIP and Ac-SDKP treatment. These treatments could enhance SOD.<br>VIP and Ac-SDKP as immune-regulating factors had benefit effect in treatment and cure of COPD. The anti-inflammatory, anti-fibrosis and anti-oxidant property of VIP and Ac-SDKP may be effective therapy in COPD (Graphical abstract).
Suggested Reviewers:	Lala Allahverdiyeva<br>Azerbaijan Medical University<br>lallaallahverdiyeva@gmail.com<br>sub specialist of asthma and allergy
	Sonia-Cornelia Badulici<br>Romanian-American University: Universitatea Romano-Americana<br>soniabadulici@gmail.com<br>MD-Phd, sub specialist of allegro-medicine
	Hamid jamoie<br>Masih e Daneshvari Hospital: Masih Daneshvari Hospital

Powered by Editorial Manager® and ProduXion Manager® from Aries Systems Corporation

	helal@modares.ac.ir<br>Sub specialist of respiratory medicine
	Hongyan Wang<br>Duke-NUS: Duke-NUS Medical School<br>hongyannwang@gmail.com<br>Sub specialist of pharmaco-biomedicine
	Wen Zhang<br>Xi\'an Medical University: Xi'an Jiaotong University<br>wenzhang.res@gmail.com<br>Sub specialist of Respiratory diseases
	shamsadin athari<br>Zanjan University of Medical Sciences<br>ss.athari@gmail.com<br>sub-specialist of immunology

Powered by Editorial Manager® and ProduXion Manager® from Aries Systems Corporation

Manuscript

Click here to access/download;Manuscript;COPD - Copy.docx

Click here to view linked References

Therapeutic effect of N-Acetyl-Seryl-Aspartyl-Proline and Vasoactive Intestinal Peptide on COPD pathophysiology

Yanna Yang[1], Ronghua Zhang[1], Wei Zhu[2], Zongxiu Yin[1]*

1. Department of Respiratory and Critical Medicine, Central Hospital Affiliated to Shandong First Medical University, No. 105 Jiefang Road, Jinan City, Shandong Province, 250013, China
2. Department of Ophthalmology, Central Hospital Affiliated to Shandong First Medical University, No. 105 Jiefang Road, Jinan City, Shandong Province, 250013, China

xuyang2019zhong@sina.com

zongxiiu.yin@gmail.com

Abstract

Background: COPD (chronic obstructive pulmonary disease) as an inflammatory respiratory system disease is caused by exposure to cigarette smoke and tobacco in long-term. Anti-inflammatory peptides can control inflammatory response of COPD. Ac-SDKP and VIP as peptide have anti-inflammatory effect and, in this study, the effect of Ac-SDKP and VIP on COPD inflammation was studied.

Methods: After producing cigarette smoke induced COPD mice model, which was treated with VIP and Ac-SDKP. The levels Antioxidant related factors, (MDA and SOD), Fibrotic factors (HP and TGF-β), pro-inflammatory cytokines (TNF-α, IL-1β and IL-6), Signaling Gene Expression (NF-κB and IκB-α) and inflammation in histopathological examination were studied.

Result: MDA, Remodeling factors, pro-inflammatory cytokines, NF-κB and IκB-α gene expression and inflammation in lung tissue were controlled by VIP and Ac-SDKP treatment. These treatments could enhance SOD.

Conclusion: VIP and Ac-SDKP as immune-regulating factors had benefit effect in treatment and cure of COPD. The anti-inflammatory, anti-fibrosis and anti-oxidant property of VIP and Ac-SDKP may be effective therapy in COPD (Graphical abstract).

Keywords: COPD, Peptide, VIP, Inflammation

Introduction

COPD as an inflammatory lung disease causes airflow obstruction with difficulty breathing, cough, wheezing and mucus production Symptoms. It's typically caused by exposure to irritating gases, most often from cigarette smoke and tobacco smoking in long-term. Emphysema and chronic bronchitis are the most common pathologic problem in COPD that causes of airway obstruction. In COPD, inflammation is part of the regular reaction to injury of lung and chronic inflammation in lung leads to initiation of remodeling and other harmful

molecular mechanisms. Currently there are no direct treatment approaches to enhance lung functions of COPD patients. Pharmacological therapies are limited to relieving symptoms and attenuating respiratory system exacerbation. Bronchodilators have side effects to COPD patients, who are elderly and have other associated diseases. Also, corticosteroids are used for treatment of severe stage exacerbation. However, corticosteroids can reduce bone density, induce skin bruising, myopathy, muscle weakness, and respiratory failure in subjects with advanced COPD (1, 2). Anti-inflammatory peptides (AIPs) can control immune and inflammatory response through a notably complex system of factors and may be effective with the lowest side effect to treat of COPD.

Ac-SDKP (N-Acetyl-Seryl-Aspartyl-Proline) as small peptide has an immune-modulatory, anti-inflammatory and anti-fibrosis effects and it is released from its precursor thymosin β4 (Tβ4). Ac-SDKP or tetrapeptide seraspenide exhibits anti-fibrotic and anti-inflammatory effects that are released from thymosin-beta 4 (Tβ4, a small ubiquitous protein with 43 amino acids) by prolyl oligopeptidase and endoproteinase asp-N enzymes. Ac-SDKP inhibits fibrosis by reducing the connective tissue growth factor, deposition of collagen, and TGF-β (3, 4).

Vasoactive intestinal peptide (VIP) as another small peptide has a wide range of bioactivity and biological functions. VIP an octacosapeptide, is one of the major transmitters, involved in a wide range of biological functions and its receptor is widely distributed in the body. In airway, VIP as immuno-reactive nerve factor, acts as neurotransmitter and neuromodulator in the non-adrenergic and non-cholinergic airway nervous system and has influence in many aspects of pulmonary function. It can relax smooth muscle and regulate immune response. It is considered a candidate for pharmaceutical agents against lung diseases, including asthma and COPD. VIP immuno-reactive nerves are present in the smooth muscle and airways glands, and the walls of pulmonary and bronchial vessels. Because of its anti-inflammatory and bronchodilating effects, VIP may be a promising drug to treat asthma and COPD (5, 6). Therefore, Ac-SDKP and VIP may have potential advantages in the treatment of COPD. In this study, the effect of Ac-SDKP and VIP on control of inflammation and treatment of COPD was studied.

Material and methods

Animals and treatments

In this study, 40 male ICR mice (6-7 weeks) were used and housed in an allergen-free condition of the animal center at 22 ± 2°C temperature and 50-60% humidity and 12 h light/dark cycle and also with free access to standard laboratory animal food and water.

Experiment protocols

There were 4 groups (n=10) that include control group, COPD group, COPD group treated with VIP, and COPD group treated with Ac-SDKP. To produce the cigarette smoke-induced mouse model of COPD, all three COPD groups (except

control group) were exposed 5 days (a week) to the mainstream cigarette smoke (5 cigarettes without filter), 4 times a day with a 10 minute smoke free interval between exposures. A 1/6 smoke/air ratio was obtained. Cigarette smoke exposure period was 8 weeks and control group was exposed to room air at the same time. From the 5th week of cigarette smoking stimulation, two groups of COPD mice were treated with Ac-SDKP and VIP for 4 weeks (one time in week) via intra-tracheal administration (7-9).

Antioxidant detection

12 h after the last administration, lung tissue and blood samples were collected. The blood was centrifuged and serum sample separated and set aside at -80 °C for further assay. The superoxide dismutase (SOD) and malondialdehyde (MDA) levels in serum were determined using SOD and MDA kits following the manufacturer's instructions. The absorbance values at 532 nm for MDA and 550 nm for SOD were detected.

Fibrotic factors determining (remodeling)

Content of the hydroxyproline (HP) is an important index of collagen fibers deposition in lung that was measured by a colorimetric modified method. The lung homogenate was hydrolyzed in HCL (6 N) for 24 h at 120°C, then NaOH was added to hydrolyzed sample for neutralization and then, 1 mL chloramine T reagent (0.05 M) was added to the sample and maintained for 20 min at room temperature. The perchloric acid (1.5 mL, 3.15 N) was also added for inactivation. Afterwards, the Ehlrich's solution was added and then incubated for 20 min at 60°C until appearance of reddish color. At 560 nm the absorbance was read and a standard curve of the pure HP was used to extrapolate the HP content values. The result was expressed as the mg of HP per gram of lung tissue (mg/g). Also, in supernatants of the lung tissue homogenate, TGF-β as main fibrotic factor was measured.

Cytokines analysis

The pro-inflammatory cytokines (TNF-α, IL-1β and IL-6) levels in the serum were measured by specific ELISA kits.

Signaling Gene Expression

The lung tissues were rinsed with ice-cold PBS and then 100 mg of it was homogenized with 900 μM PBS. After being centrifuged, the separated cells were collected. The mRNA was extracted using TRI reagent and then, cDNA was synthesized using a cDNA synthesis kit. At least, the expressions of (inhibitor of NF-κB (IκB-α) and nuclear transcription factor-κB (NF-κBp65) genes was studied by real-time PCR with specific primer sequences.

Histological examination

At the end of the experiment, the lung tissues were fixed, then were embedded in paraffin, sliced and stained with H&E for the histopathological examination. The observation was carried out under an optical microscope in a blind manner and the infiltration of inflammatory cells was studied. The scoring system was as

follows: no cells: 0, a few cells: 1, a ring of cells 1 cell layer deep: 2, a ring of cells 2-4 cell layers deep: 3 and a ring of cells more than 4 cell layers deep: 4.

Statistical analysis

The data are showed as the means±SD and analyzed by one-way ANOVA with Tukey multiple comparison test. Less than 0.05 for the P value was considered significant. The graphs were drowned by GraphPad prism.

Result

Antioxidant detection

The oxidative stress indicators were measured and SOD had significant different between treated and non-treated groups and non-treated COPD group had decreased level. In COPD group, MDA (3.72±0.14) was increased significantly in compare with control group (1.39±0.29) and in the VIP and Ac-SDKP treated COPD groups, MDA was decreased significantly (1.90±0.22 and 2.20±0.27 respectively) (Fig. 1).

Remodeling factors

The HP and TGF-β content as an important index of collagen fibers deposition in lung were significantly increased in COPD group (5.02±0.28 mg/g and 317.32±10.01 pg/ml respectively) compared control group (1.59±0.21 mg/g and 89.65±14.64 pg/ml respectively). The HP and TGF-β were significantly decreased with VIP (1.99±0.26 mg/g and 127.32±9.52 pg/ml respectively) and Ac-SDKP (2.24±0.31 mg/g and 194.32±11.54 pg/ml respectively) treatment (Fig. 2).

Cytokines analysis

The pro-inflammatory cytokines levels (TNF-α, IL-1β and -6) in the serum of COPD group (369.32±30.52, 319.65±23.65, 253.48±23.98 pg/ml respectively) were significantly increased compared to control group (239.69±22.76, 176.63±21.09, 189.32±25.63 pg/ml respectively). Treatment with VIP and Ac-SDKP could significantly decrease TNF-α (278.54±26.10 and 239.5±21.99 pg/ml respectively) and IL-1β (221.98±29.53, 209.76±22.65 pg/ml respectively) levels in COPD but IL-6 level decreasing was not significant (Fig. 3).

Signaling Gene Expression

NF-κB and IκB-α gene expression were changed in COPD group compared to control group. Treatment with VIP and Ac-SDKP had no significant effect of IκB-α gene expression ($p>0.05$). But, VIP and Ac-SDKP treatment could control NF-κB gene expression (3.5±0.5 and 4.3±0.7 respectively) that was increased in non-treated COPD group (8.6±1.2) (Fig. 4).

Histopathology

Histology revealed pathologic features of lung in the COPD mice compared to the control group showed that inflammation was increased in lung of the COPD mice (3.6±0.3) compared to the control mice (0.5±0.2). Moreover, an increased number of inflammatory cells in the COPD lungs of the mice were significantly decreased ($p<0.05$) in VIP (1.7±0.5) and Ac-SDKP (2.4±0.1) groups (Figs. 5).

Discussion

COPD is characterized by a progressive bronchial constrictive problem resulting cigarette smoke that contains over than 4000 constituents and is a major causative factor in the COPD development, which leads to cell death via oxidative DNA damage, necrosis and apoptosis. VIP has potential beneficially effects in the control of inflammatory lung diseases such as COPD and asthma. VIP exerts biological functions via the stimulation of protein kinases including the MAP kinase and C/PKC pathways, as well as the adenylate cyclase/PKA pathway. VIP acts as a potent smooth muscle relaxant and induces bronchodilation and its bronchodilatory effect is 100-fold more potent than adrenergic dilation by isoproterenol. In addition, VIP also regulates microvascular permeability and mucus secretion, and inhibites the macromolecules releasing from mucus-secreting glands. Also, VIP induces chloride secretion by epithelium cells of the bronchia and stimulates ciliary motility. However, VIP clinical application has limitation for a number of reasons, including its short plasma half-life after intravenous administration (4, 5, 10, 11). In this study, we used intra-trachea administration form of VIP and therefore, there was not similar problems. With this route, systemic administration cardiovascular side effects are prevented and choosing method of drug administration is critical.

Some anti-inflaming peptides such as Ac-SDKP are strong immunomodulators and with interfering in the signal transduction pathways are involved in inflammatory cytokine producing. Ac-SDKP has anti-fibrotic and anti-inflammatory effects and its anti-inflammatory activity is partially attributed to the inhibition of the bone marrow stem cells differentiation into macrophages, and macrophage activation, as well as of the TNF-α releaseing by activated macrophages and the antifibrotic effect of Ac-SDKP is explained by reducing the TGF-β, connective tissue growth factor, and inhibition of collagen deposition. Angiotensin-converting enzyme can clear Ac-SDKP and fibrosis can be treated successfully by angiotensin-converting enzyme inhibitors and analogs of Ac-SDKP (4, 12, 13). Histopathology features of lung in our study showed that increased inflammation in lung of the COPD mice was controlled by VIP and Ac-SDKP treatment. Also, main remodeling factors, The HP and TGF-β in COPD lung were decreased with VIP and Ac-SDKP and therefore, collagen fibers deposition could be prevented.

Ac-SDKP can inhibit the TNF-α-mediated activation of IKK-β-dependent NF-κB pathway via IKK-β inhibition, thus, inhibiting IκB kinase (4, 14, 15). Ac-SDKP and VIP could control NF-κB gene expression that was increased in COPD mice. Therefore, SDKP and VIP can control immunoo-inflammatory response in lung and can be efctive agents in cure oof COPD. Also, the main pro-inflammatory cytokines, TNF-α and IL-1β in the serum of COPD group were decreased by treatment with VIP and Ac-SDKP. These treatments had weak

effect of on the IL-6 level. The NF-κB is one of the main signaling pathway in cytokine activity.

Ac-SDKP decreases the number of infiltrating inflammatory cells especially macrophages and mast cells, also TGF-β expression and collagen deposition. The macrophages and mast cells are associated with fibrosis and fibrosis is initiated by these cells. In addition, mast cell is an important source of MCP-1 (monocyte chemoattractant protein-1), which can recruit more macrophages to the inflamed site. Thus, macrophages and mast cells inhibition by Ac-SDKP may have anti-fibrotic and anti-inflammatory effect. Indeed, Ac-SDKP inhibits TGF-β signaling, resulting in inhibition of Smad activation, and inhibition of TGF-β by Ac-SDKP is an important factor in mediating anti-fibrotic effect (3, 16, 17).

Although oxidative stress and immune-inflammatory responses play critical roles in the COPD pathogenesis. Tobacco smoke contains ROS and many different chemical components, which mediate the inflammation processes, repairment, and fibrosis. In addition, oxidative stress plays an important role in the inflammation progress. Moreover, oxidative stress affects remodeling and infiltration of surfactants to alveoli. The cigarette exposure induces COPD and provokes severe inflammation mediated by NF-κB-dependent cytokines production. Under normal condition, NF-κB is inactive-cytoplasmic form binding with the IκBs. Nevertheless, cigarette smoking stimulation conduces to the phosphorylation and degradation of IκBα. Also, IKK-mediated IκB-α degradation is a notable key in NF-κB activation that leads to the p65 subunit translocation from the cytoplasm to the nucleus. Consequently, NF-κBp65 binds to promoter regions of the pro-inflammatory cytokine genes, thus induces the transcription of inflammatory mediators including TNF-α, IL-1β and -6 that are responsible for recruitment of cells and lung injury. Moreover, cigarette smoke induces oxidative stress and the anti-oxidation property of treatment can suppress ROS formation, protect antioxidant enzyme system. Also, ROS induce NF-κB through several distinct mechanisms including activation of MAPK and IKK. Therefore, suppression of oxidative stress through suppressing NF-κB pathway is important in treatment of COPD (18, 19). In this study, treatment with Ac-SDKP and also VIP could harness NF-κB pathway and prevent inflammation and oxidative stress. The oxidative stress indicators SOD and MDA could be modulated by both of the VIP and Ac-SDKP and this modulation was benefit in cure or control of COPD.

Ac-SDKP mediated reduction in enzyme involves in collagen and elastin crosslinking. A dysregulation of matrix-metalloprotease (MMPs) and tissue inhibitors of metalloprotease (TIMPs) often result in accumulation of collagen and tissue fibrosis. Ac-SDKP inhibits IL-1 mediated MMP-2 and -9 activation, and increases activities of MMP's inhibitors TIMP-1 and TIMP-2. Also, Ac-SDKP reduces collagen synthesis that is linked with lower p-ERK1/2 and NF-κb activities. In addition to, Ac-SDKP reduces inflammation by decreasing endothelial ICAM-1 expression and decreases TNF-α-induced ICAM-1

expression via canonical IKK-dependent NF-κb inhibition. Therefore, the Ac-SDKP in COPD has therapeutic anti-inflammatory, -fibrotic and -oxidant effects (20-22).

VIP-deficiency is a causative factor in the pathogenesis of the lung diseases and long-acting VIP analogues have strong advantageous for the therapy of lung diseases such as COPD. In addition, the anti-inflammatory and anti-oxidant property of VIP and Ac-SDKP may be alternative therapy in case of COPD. For this purpose, it would be necessary to completely understand the correlation between specific sequences of the amino acid and anti-inflammatory features for more targeted, useable and reproducible effects.

Ethics approval and consent to participate

All methods have been approved by ethical committee of laboratory animal moral department of veterinary medicine (No: 2021VET.MED.ETHIC.A5680032).

Consent for publication

Not Applicable.

Availability of data and materials

Not Applicable.

Competing interests

There is no competing interest.

Funding

Not Applicable.

Authors' contributions

YY, RZ, WZ, ZY have participated in the design, examination, testing, analysis and drafting the manuscript. YY and ZY supervised the study. The authors declare that all data were generated in-house and that no paper mill was used.

Acknowledgements

Not Applicable.

References

1. Yang-ChunPark, MirimJin, Seung-HyungKim, Min-HeeKim, Uk Namgung, YoonYeo. Effects ofinhalablemicroparticleof flowerof Lonicerajaponica in a mouse modelofCOPD. Journal of Ethnopharmacology 2014; 151:123–130
2. Johannes M. van Noort, Malika Bsibsi, Peter J. Nacken, Wouter H. Gerritsen, Sandra Amor, Inge R. Holtman, Erik Boddeke, Ingrid van Ark, Thea Leusink-Muis, Gert Folkerts, Wim E. Hennink, Maryam Amidi. Activation of an immune-regulatory macrophage response and inhibition of lung inflammation in a mouse model of COPD using heat-shock protein alpha B-crystallin-loaded PLGA microparticles. Biomaterials 2013; 34:831-840
3. Saman Rasoul, Oscar A. Carretero, Hongmei Peng, Maria A. Cavasin, Jialong Zhuo, Alicia Sanchez-Mendoza, David R. Brigstock, Nour-Eddine Rhaleb. Antifibrotic effect of Ac-SDKP and angiotensin-converting enzyme inhibition in hypertension. J Hypertens. 2004; 22(3): 593–603.
4. Maryam Dadar, Youcef Shahali, Sandip Chakraborty, Minakshi Prasad, Fatemeh Tahoori, Ruchi Tiwari, Kuldeep Dhama. Antiinflammatory peptides: current knowledge and promising Prospects. Inflammation Research 2018 doi.org/10.1007/s00011-018-1208-x
5. Satomi Onoue, Shizuo Yamada, Takehiko Yajima. Bioactive analogues and drug delivery systems of vasoactive intestinal peptide (VIP) for the treatment of asthma/COPD. Peptide 2007; 28:1640–1650
6. Seyyed Shamsadin Athari, Zahra Pourpak, Gert Folkerts, Johan Garssen, Mostafa Moin, Ian M. Adcock, Masoud Movassaghi, Mehdi Shafiee Ardestani, Seyed Mohammad Moazzeni, Esmaeil Mortaz. Conjugated Alpha-Alumina nanoparticle with vasoactive intestinal peptide as a Nano-drug in treatment of allergic asthma in mice. European Journal of Pharmacology 2016; 791:811–820
7. L. Wollin, M.P.Pieper**.** Tiotropium bromide exerts anti-inflammatory activity in a cigarette smoke mouse model of COPD. Pulmonary Pharmacology & Therapeutics 2010; 23:345-354
8. Metcalfe, H.J., Lea, S., Hughes, D., Khalaf, R., Abbott-Banner, K., Singh, D., 2014. Effects of cigarette smoke on Toll-like receptor (TLR) activation of chronic obstructive pulmonary disease (COPD) macrophages. Clinical and experimental immunology 176, 461-472.
9. Stevenson CS, Birrell M. Moving towards a new generation of animal models for asthma and COPD with improved clinical relevance. Pharmacol Ther 2011; 130:93e105.
10. Abad C, Gomariz RP, Waschek JA. Neuropeptide mimetics and antagonists in the treatment of inflammatory disease: focus on VIP and PACAP. Curr Top Med Chem 2006; 6:151-63.
11. Ohmori Y, Onoue S, Endo K, Matsumoto A, Uchida S, Yamada S. Development of dry powder inhalation system of novel vasoactive intestinal peptide (VIP) analogue for pulmonary administration. Life Sci 2006; 79:138–43.

12. Zhu L, Yang X-P, Janic B, Rhaleb N-E, Harding P, Nakagawa P, et al. Ac-SDKP suppresses TNF-α-induced ICAM-1 expression in endothelial cells via inhibition of IκB kinase and NF-κB activation. Am J Physiol Heart Circ Physiol. 2016; 310(9):1176-83.
13. N.E. Rhaleb, S. Pokharel, U.C. Sharma, H. Peng, E. Peterson, P. Harding, X.P. Yang, and O.A. Carretero. N-acetyl-Ser-Asp-Lys-Pro inhibits interleukin-1beta-mediated matrix metalloproteinase activation in cardiac fibroblasts. Pflugers Archiv : European journal of physiology. 2013; 465:1487-1495.
14. L. Zhu, X.P. Yang, B. Janic, N.E. Rhaleb, P. Harding, P. Nakagawa, E.L. Peterson, and O.A. Carretero. Ac-SDKP suppresses TNF-alpha-induced ICAM-1 expression in endothelial cells via inhibition of IkappaB kinase and NF-kappaB activation. American journal of physiology Heart and circulatory physiology. 2016; 310:1176-1183.
15. Y.H. Liu, M. D'Ambrosio, T.D. Liao, H. Peng, N.E. Rhaleb, U. Sharma, S. Andre, H.J. Gabius, and O.A. Carretero. N-acetyl-seryl-aspartyl-lysyl-proline prevents cardiac remodeling and dysfunction induced by galectin-3, a mammalian adhesion/growth-regulatory lectin. American journal of physiology Heart and circulatory physiology. 2009; 296:404-412.
16. Sharma U, Rhaleb N-E, Pokharel S, Harding P, Rasoul S, Peng H, et al. Novel anti-inflammatory mechanisms of N-Acetyl-Ser-Asp-Lys-Pro in hypertension-induced target organ damage. Am J Physiol Heart Circ Physiol. 2008; 294(3):1226-32.
17. Peng H, Carretero OA, Brigstock DR, Oja-Tebbe N, Rhaleb N-E. Ac-SDKP reverses cardiac fibrosis in rats with renovascular hypertension. Hypertension. 2003; 42(6):1164–70.
18. Rui Zhou, Fen Luo, Hui Lei, Kai Zhang, Jingyan Liu, He He, Jin Gao, Xiayun Chang, Ling He, Hui Ji, Tianhua Yan and Tong Chen, Liujunzi Tang, a famous traditional Chinese medicine, ameliorates cigarette smokeinduced mouse model of COPD. Journal of Ethnopharmacology, 2016; 193:643-651.
19. Kumar N, Nakagawa P, Janic B, Romero CA, Worou ME, Monu SR, et al. The anti-inflammatory peptide Ac-SDKP is released from thymosin-β4 by renal meprin-α and prolyl oligopeptidase. Am J Physiol Renal Physiol. 2016; 310(10):1026-34.
20. Kumar N, Yin C, The anti-inflammatory peptide Ac-SDKP: Synthesis, Role in ACE inhibition, and its therapeutic potential in hypertension and cardiovascular diseases. Pharmacological Research 2018; 134:268-279
21. Tomaki, M., Sugiura, H., Koarai, A., Komaki, Y., Akita, T., Matsumoto, T., Nakanishi, A., Ogawa, H., Hattori, T., Ichinose, M. Decreased expression of antioxidant enzymes and increased expression of chemokines in COPD lung. Pulmonary pharmacology & therapeutics 2007; 20, 596-605.
22. N. Kumar, P. Nakagawa, B. Janic, C.A. Romero, M.E. Worou, S.R. Monu, E.L. Peterson, J. Shaw, F. Valeriote, E.M. Ongeri, J.M. Niyitegeka, N.E. Rhaleb, and O.A. Carretero. The anti-inflammatory peptide Ac-SDKP is released from thymosin-beta4 by renal meprin-alpha and prolyl oligopeptidase. Am J Physiol Renal Physiol. 2016; 310:1026-1034.

Legends

Fig. 1. Antioxidant. The oxidative stress indicators SOD and MDA were measured in COPD, control, VIP and Ac-SDKP treated COPD groups.

Fig. 2. Remodeling factors. The HP and TGF-β content as remodeling factors and important index of collagen fibers deposition were measured in lung

Fig. 3. Cytokines. The TNF-α, IL-1β and IL-6 levels as pro-inflammatory cytokines in the serum of all groups were measured by ELISA.

Fig. 4. Gene Expression. The gene expression of NF-κB and IκB-α were studied in COPD, control, VIP and Ac-SDKP treated groups by Real time PCR.

Figs. 5. Histopathology. Histology revealed pathologic features of lung was done and inflammation in lung of the all mice was studied.

Click here to access/download;Figure;Figure (1).TIF

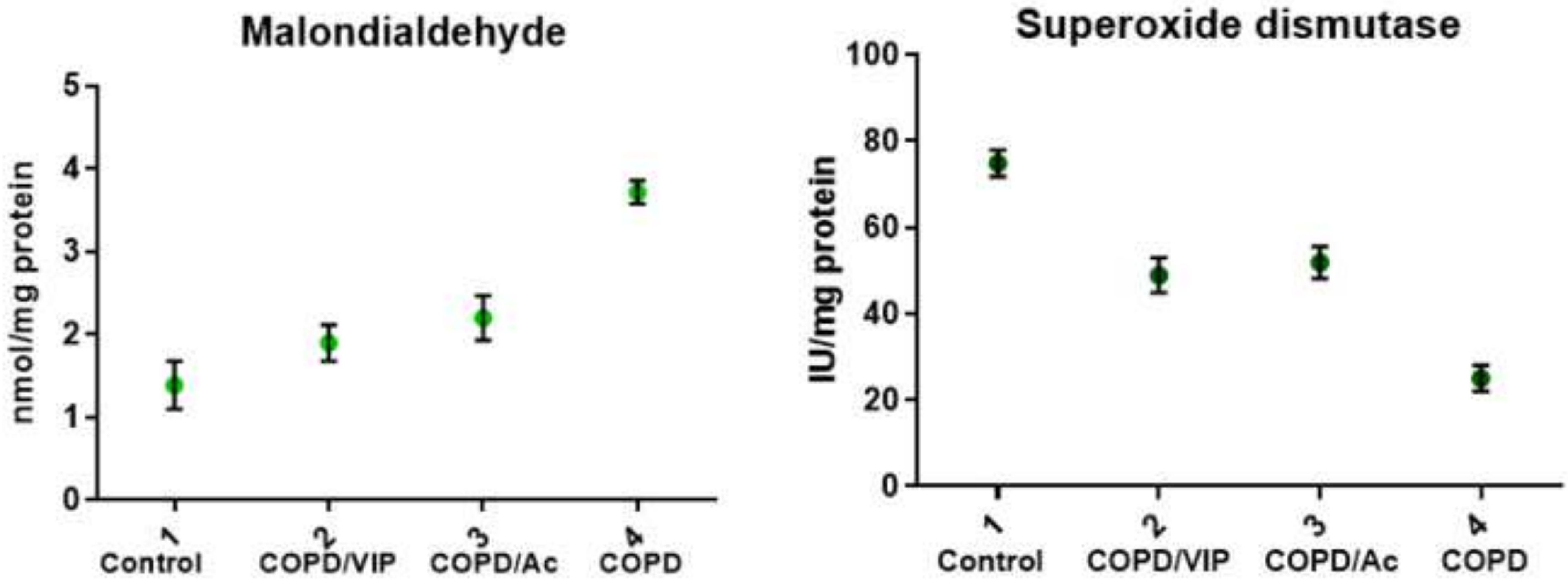

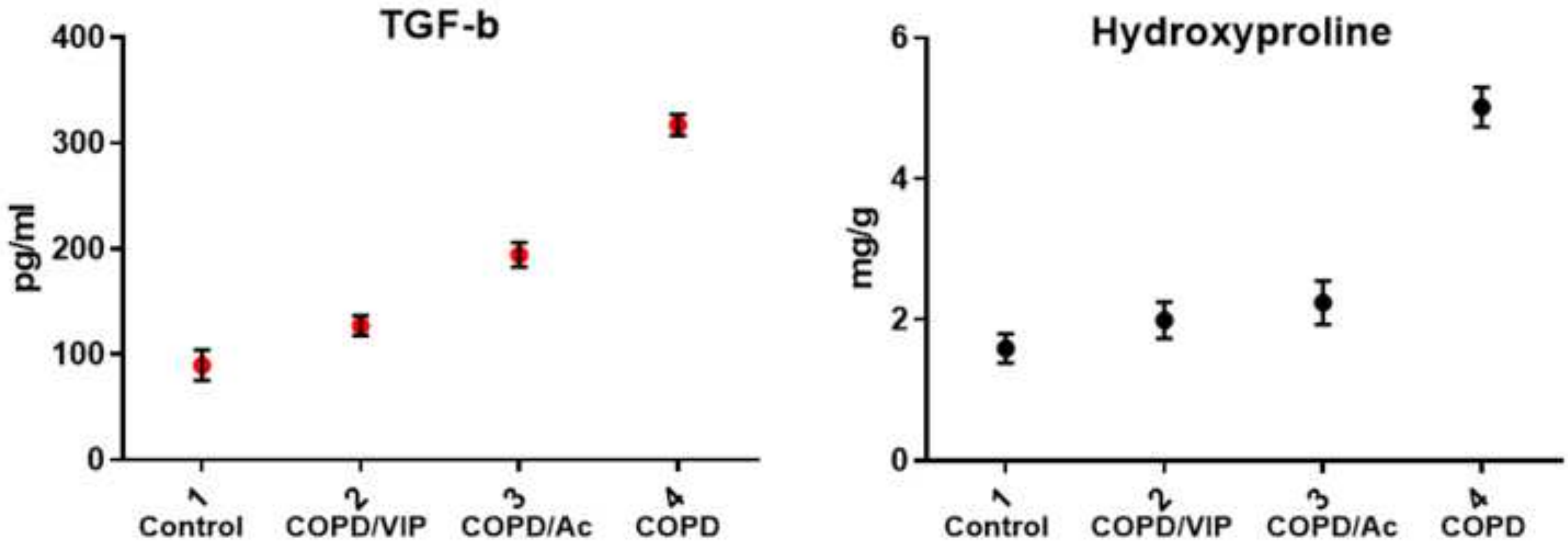
TGF-b
pg/ml
400
300
200
100
0
1
Control
2
COPD/VIP
3
COPD/Ac
4
COPD
Hydroxyproline
mg/g
6
4
2
0
1
Control
2
COPD/VIP
3
COPD/Ac
4
COPD

Click here to access/download;Figure;Figure (3).TIF

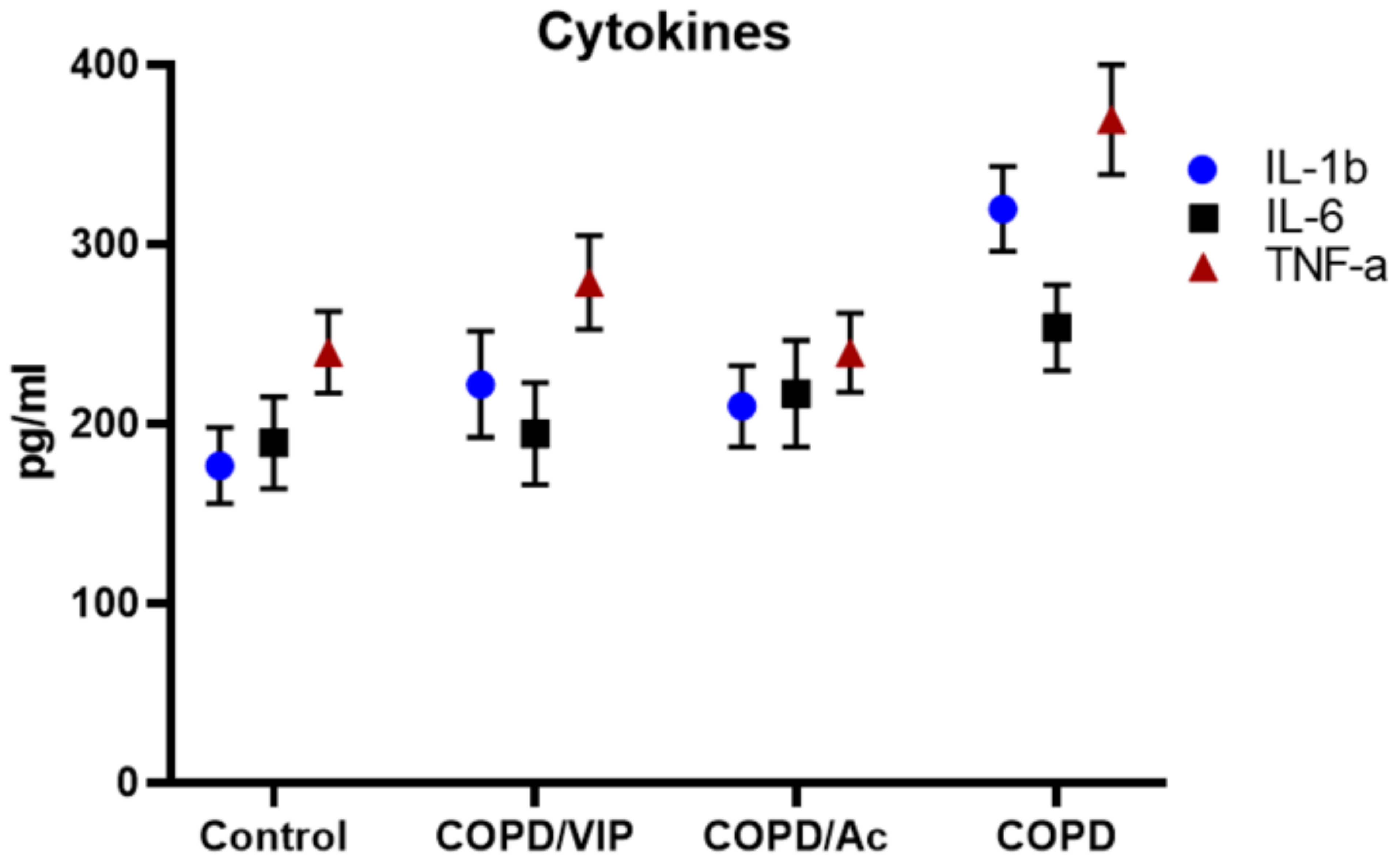

Figure Click here to access/download;Figure;Figure (4).TIF

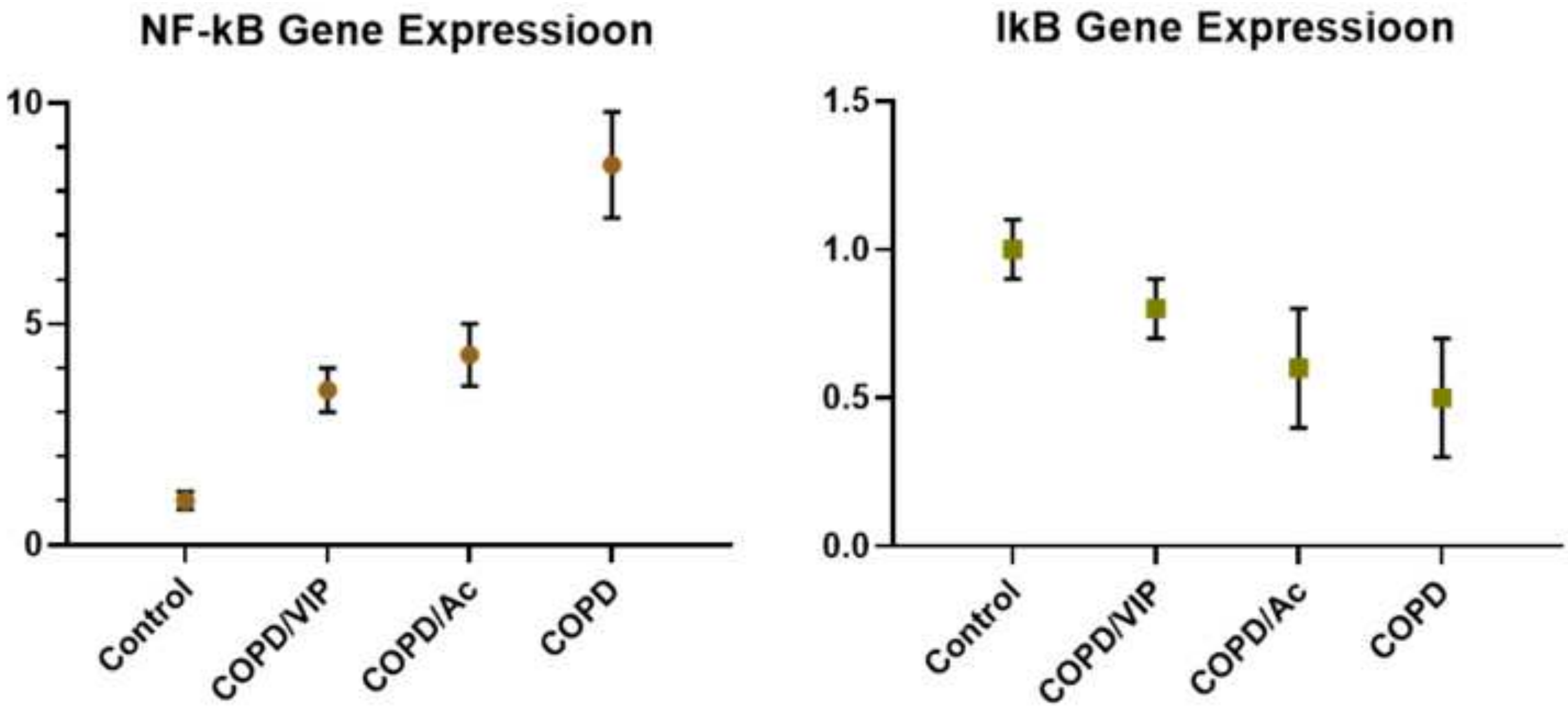

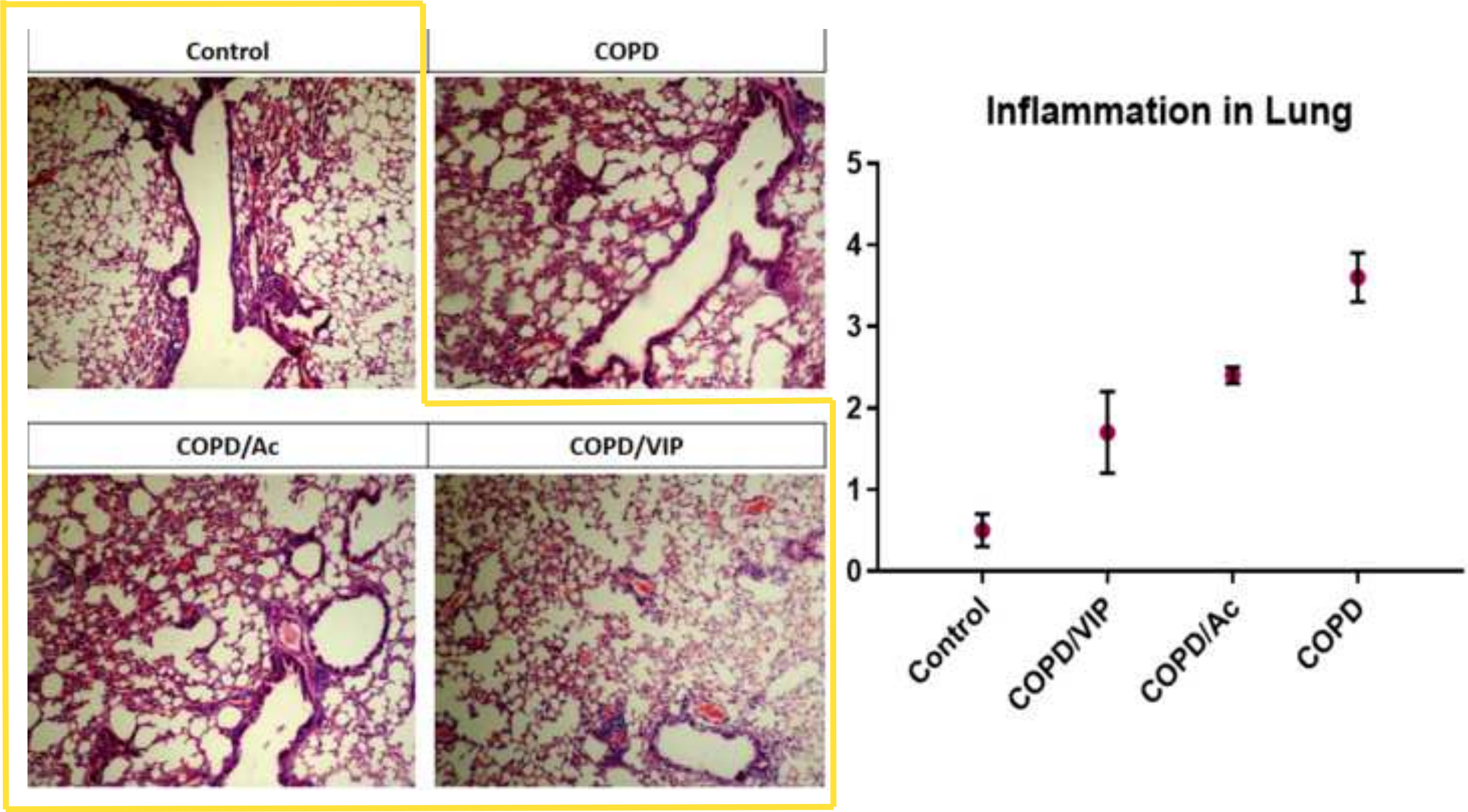
Control
COPD
COPD/Ac
COPD/VIP
Inflammation in Lung
5
4
3
2
1
0
Control
COPD/VIP
COPD/Ac
COPD

Figure Click here to access/download;Figure;Graphical Abstract.TIF

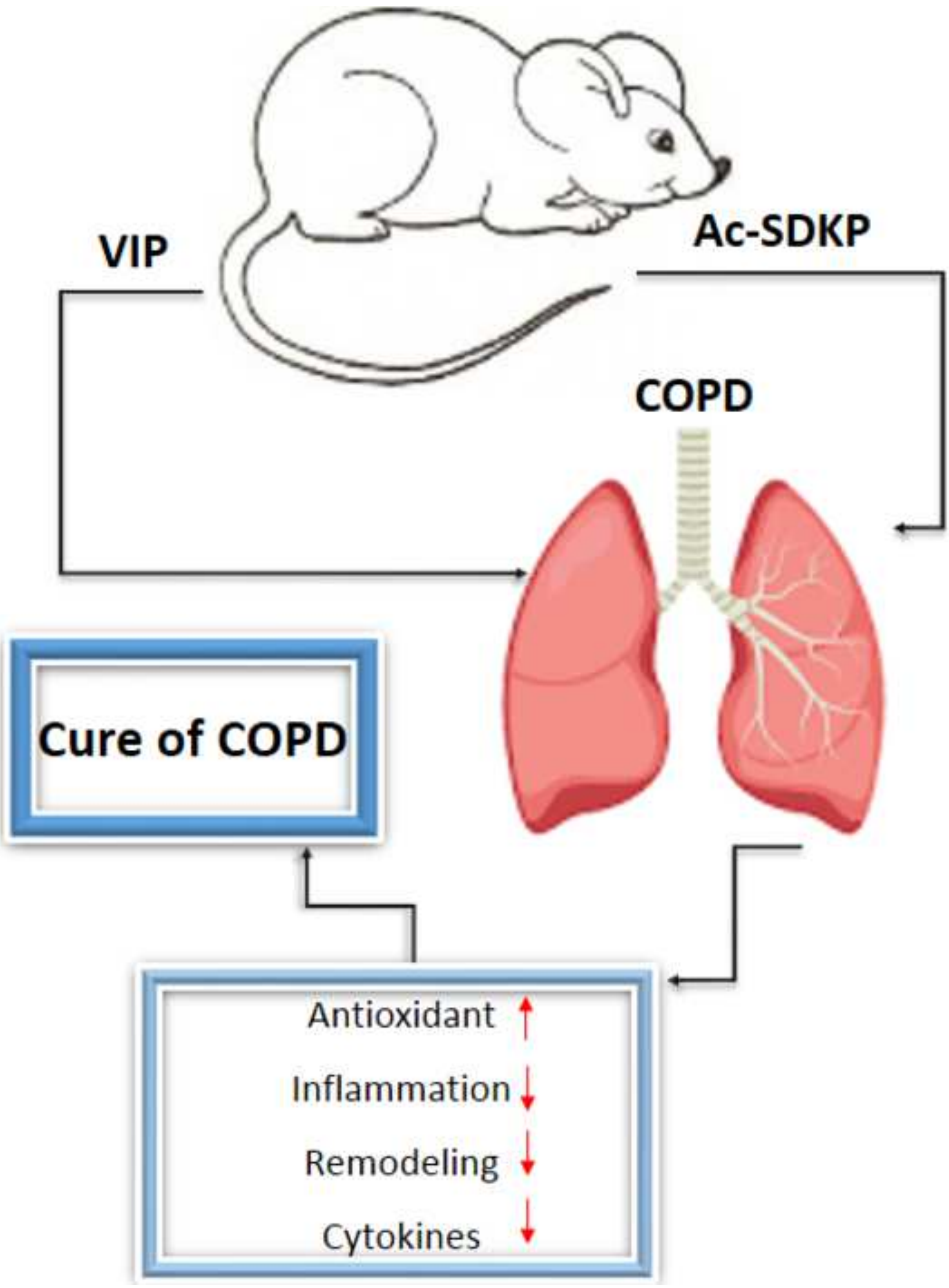

Supplement: Supplementary file 4 — Supplementary file4 (PDF 694 KB) [file 210_2023_2741_MOESM4_ESM.pdf]
