# Supplementary material for: Fake paper identification in the pool of withdrawn and rejected manuscripts submitted to Naunyn–Schmiedeberg’s Archives of Pharmacology
Source: Naunyn Schmiedebergs Arch Pharmacol. 2023 Oct 5;397(4):2171–81. doi: 10.1007/s00210-023-02741-w (PMC10933159; doi:10.1007/s00210-023-02741-w)
Supplement: Supplementary file 5 — Supplementary file5 (PDF 601 KB) [file 210_2023_2741_MOESM5_ESM.pdf]

Figure S5

Color coding:

Yellow highlighted text	The text is identical in the NSAP version and the published version of this paper.
Red highlighted text	There are differences in the text between the NSAP version and the published version of this paper (different content or different wording).
Yellow bordered figure	This figure is identical in both versions of this paper.

Naunyn-Schmiedeberg's Archives of Pharmacology

Dalbergioidin ameliorates doxorubicin-induced renal fibrosis via suppressing the TGF-β signal pathway

--Manuscript Draft--

**Manuscript Number:**	NSAP-D-16-00098
**Full Title:**	Dalbergioidin ameliorates doxorubicin-induced renal fibrosis via suppressing the TGF-β signal pathway
**Article Type:**	Original Article
**Corresponding Author:**	Changliang Xu<br>Nanjing University of Chinese Medicine<br>CHINA
**Corresponding Author Secondary Information:**	
**Corresponding Author's Institution:**	Nanjing University of Chinese Medicine
**Corresponding Author's Secondary Institution:**	
**First Author:**	Daliang Xu
**First Author Secondary Information:**	
**Order of Authors:**	Daliang Xu
	Yang Dong
	Yulian Jin
	Changliang Xu
**Order of Authors Secondary Information:**	
**Funding Information:**	Young Scientists Fund of the National Natural Science Foundation of China (81302829) — Mr Changliang Xu
**Abstract:**	The effect of Dalbergioidin (DAL), a well-known natural product extracted from Uraria crinite (L.) Desv. ex DC. (Fabaceae), was investigated in doxorubicin (DXR)-induced renal fibrosis in mice. Mice were pretreated for 7 days with DAL, followed by a single injection of DXR (10 mg/kg) via the tail vein. Renal function was analyzed at 5 weeks post-DXR treatment. DXR caused nephrotoxicity. In contrast, the symptoms of nephrotic syndrome were greatly improved by the DAL treatment. The indices of renal fibrosis, the phosphorylation of Smad3 and the expression of α-SMA, fibronectin, Col I, E-cadherin, TGF-β and Smad7 in response to DXR were all similarly modified by DAL. The present findings suggest that DAL improved the markers for kidney damage investigated in this model of DXR-induced experimental nephrotoxicity.
**Suggested Reviewers:**	Katrien Beneden<br>kvbenede@vub.ac.be
	Leo A Grunsven<br>lvgrunsv@vub.ac.be

Powered by Editorial Manager® and ProduXion Manager® from Aries Systems Corporation

Manuscript

Click here to view linked References

Click here to download Manuscript Naunyn-Schmiedebergs Archives of Pharmacology.docx

Dalbergioidin ameliorates doxorubicin-induced renal fibrosis via suppressing the TGF-β signal pathway

Daliang Xu[a,#], Yang Dong[a,#], Yulian Jin[a,*], Changliang Xu[b,*]

a. Department of Nephrology, Anhui provincial children's hospital, Hefei, China

b. National Clinical Research Center of Kidney Diseases, Jinling Hospital, Nanjing University School of Medicine, Nanjing, China

# These authors contributed equally to this work and are to be considered first authors

* Corresponding author:

Department of Nephrology, Anhui provincial children's hospital, No.39, Wang Jiang Road, Hefei city, Anhui province, 230051

E-mail address: 113560206@qq.com (Y.-L. Jin).

National Clinical Research Center of Kidney Diseases, Jinling Hospital, Nanjing University School of Medicine, 305 East Zhongshan Road, Nanjing 210016, Jiangsu, China.

E-mail address: how11are22you33@163.com (C.-L Xu).

Keywords

Dalbergioidin; doxorubicin; nephrotoxicity; nephroprotective effects; TGF-β

Abbreviations

Dalbergioidin (DAL); Doxorubicin (DXR); Blood urea nitrogen (BUN); lipid peroxides (LPO); reduced glutathione (GSH); Reactive oxygen species (ROS), Bovine serum albumin (BSA); trichloroacetic acid (TCA); perchloric acid (PCA); 2′7′-dichlorodihydrofluorescein diacetate (DCFH-DA); 2′7′-dichlorofluorescein (DCF); malonyldialdehyde (MDA);thiobarbituric acid (TBA);TBA reactive substances (TBARS)

Abstract

The effect of Dalbergioidin (DAL), a well-known natural product extracted from *Uraria crinite (L.) Desv. ex DC. (Fabaceae)*, was investigated in doxorubicin (DXR)-induced renal fibrosis in mice. Mice were pretreated for 7 days with DAL, followed by a single injection of DXR (10 mg/kg) via the tail vein. Renal function was analyzed at 5 weeks post-DXR treatment. DXR caused nephrotoxicity. In contrast, the symptoms of nephrotic syndrome were greatly improved by the DAL treatment. The indices of renal fibrosis, the phosphorylation of Smad3 and the expression of α-SMA, fibronectin, Col I, E-cadherin, TGF-β and Smad7 in response to DXR were all similarly modified by DAL. The present findings suggest that DAL improved the markers for kidney damage investigated in this model of DXR-induced experimental nephrotoxicity.

Introduction

Doxorubicin (DXR) is an anthracycline glycoside antibiotic that has broad spectrum antitumour activity against a variety of human solid tumours, such as ovarian, breast, lung, uterine and cervical cancers, Hodgkin's disease, soft tissue and primary bone sarcomas, as well as against several other cancer types and haematological malignancies (Bernard, 1967, Carter, 1972, Blum and Carter, 1974). However, DXR does not discriminate between cancer and normal cells and eradicates not only fast-growing cancer cells but also other rapidly growing cells in the body; therefore, its use in chemotherapy has been restricted. DXR has a variety of toxicities including cardiac, hepatic, renal and hematological (Yilmaz et al., 2006, Injac et al., 2008, Mohan et al., 2010, Lai et al., 2011, Desai et al., 2013). Although the mechanism underlying the severe cytotoxicity from DXR is not fully understood, reactive oxygen species (ROS) are assumed to be a key factor. Understanding the events controlling this oxidative injury is very important. DXR treatment leads to the overproduction of hydroxyl radicals, hydrogen peroxide and superoxide anions, which cause membrane lipid peroxidation (Oz and Ilhan, 2006). Therefore, an escalating amount of data suggests that simultaneous treatment with DXR and an antioxidant may alleviate the toxicity.

Uraria crinita (L.) Desv. ex DC. (Fabaceae), which has some of the greatest health benefits, is widely distributed throughout India, Thailand, Indonesia and China. It is used as herbal medicine for a long history. It has bioactive properties, such as antioxidant activity, anti-ulcer effect, osteogenic activity and its roots have also been

used to treat chills, edema, and stomachache through its anti-inflammatory activity(Yen et al., 2001, Mao et al., 2014). The aim of the present study was to investigate the effect of Dalbergioidin (DAL), which is a well-known anthocyanin from *Uraria crinita (L.) Desv. ex DC. (Fabaceae)*, on DXR-induced renal fibrosis in mice. The present study was performed to verify if treatment with DAL is able to counteract renal fibrosis induced by DRX *in vivo*. In addition, the mechanism of DAL action was also investigated.

Methods

Reagents

DAL was purchased from BioBioPha Co., Ltd. SMAD3, p-SMAD3, SMAD7, α-SMA, fibronectin, Col I, E-cadherin, and TGF-β were purchased from Santa Cruz Biotechnology, Inc. Bovine serum albumin (BSA), DXR, sodium hydroxide, ferric nitrate, trichloroacetic acid (TCA) and perchloric acid (PCA) were obtained from Sigma-Aldrich.

Animals

Adult, 8-week old, BALB/c mice (~20 g) were obtained from the Shanghai Laboratory Animal Center, Chinese Academy of Science. The mice were housed in an animal care facility at room temperature (25±1°C) with a 12-h light/dark cycle and were given free access to standard diet and water. Before the treatment, the mice were left for 14 d to acclimatize. Animal care and use were in compliance with the

Provisions and General Recommendation of the Chinese Experimental Animals Administration Legislation and were approved by the Science and Technology Department of Jiangsu Province.

Experimental procedure

The mice were randomly divided into three groups of eight mice each. Group I served as the control group for 42 d. Group II served as the model group and received a single vein injection of DXR (10 mg/kg) on the 7th day. Group III served as the treatment group and was pretreated with DAL (30 mg/kg, i.p) for 42 d, and on the 7th day, a single vein injection of DXR (10 mg/kg) was administered. On the 42nd day, the mice were sacrificed by cervical dislocation, and kidney and blood samples were taken after perfusion to evaluate the various biochemical parameters.

Measurements of urine and plasma

Urine and blood samples were collected as previously described (Liu et al., 2013). Urine albumin, plasma triglyceride levels, plasma urea levels and serum creatinine levels were determined using commercialized kits, an enzyme-linked immunosorbent assay kit (Exocell), a Urea Nitrogen Direct Kit (Stanbio Laboratory), a LabAssay Triglyceride ELISA Kit (Wako) and a Creatinine Liquicolor Kit (Stanbio Laboratory).

Masson-trichrome staining

Paraffin-embedded mouse kidney sections were prepared using a routine procedure.

The kidney sections were also subjected to Masson-trichrome staining to assess collagen deposition and fibrotic lesions.

Determination of GSH in vivo

The effect of the DAL treatment on GSH levels was evaluated using a commercial kit (Cayman Chemical Co.). The kidney tissue homogenate was used for GSH measurements with a GSH Assay Kit following the manufacturer's protocol. The GSH content was determined by comparing GSH levels with the controls and then normalizing those levels to total protein content. GSH content was determined by comparing GSH levels with the controls and then normalizing those levels to total protein content.

Determination of MDA levels in vivo

The lipid peroxidation of the kidney tissue was studied by measuring the malonyldialdehyde (MDA) levels in a colorimetric method involving thiobarbituric acid (TBA) adduct formation. Kidney tissue homogenate was prepared, and the amounts of TBA reactive substances (TBARS), such as MDA, were measured by a reaction with TBA using a commercial TBARS Assay Kit (Cayman Chemical Co.). We followed the manufacturer’s protocol. The MDA levels were determined by comparing the samples with the standards and normalizing those values to total protein content.

Reverse-transcription–polymerase chain reaction (RT–PCR)

Total RNA was isolated from the cells using a commercial TRIzol reagent kit (Invitrogene, USA), and the RNA concentrations were measured spectrophotometrically. The first cDNA synthesis was performed following the manufacturer's instructions (Takara, JPN). The specific primers for fibronectin, α-SMA, E-cadherin, Col III, SMAD7, TGF-β and GAPDH (loading control) were as follows: fibronectin: sense 5'- CGAGGTGACAGAGACCACAA-3', antisense 5'-CTGGAGTCAAGCCAGACACA-3'; α-SMA: sense 5'-TGTGCTGGACTCTGGAGATG-3', antisense 5'-ATGTCACGGACAATCTCACG-3'; E-cadherin: sense 5'- AATGGCGGCAATGCAATCCCAAGA-3', antisense 5'- TGCCACAGACCGATTGTGGAGATA-3'; Col III: sense 5'- AGGCAACAGTGGTTCTCCTG-3', antisense 5'-GACCTCGTGCTCCAGTTAGC-3'; smad7: sense 5'-AGGTGTTCCCCGGTTTCTCCA-3'; antisense: 5'-TTCACAAAGCTGATCTGCACGGT-3'; TGF-β: sense 5'-GCAACATGTGGAACTCTACCAGAA-3', antisense 5'-GACGTCAAAAGACAGCCACTCA-3'; GAPDH: sense 5'-AACTTTGGCATTGTGGAAGG-3', antisense 5'-ACACATTGGGGGTAGGAACA-3'. SYBR green PCR Master Mix was used for real-time PCR analysis.

Western blot analyses

The tissues and cells were homogenized in 10 mM Tris / 1 mM EDTA / protease and phosphatase inhibitor cocktails (1:1000; Sigma–Aldrich) by sonication and subsequently centrifuged at 10000×*g* for 10 min. The supernatant was decanted. Primary antibodies were added to a TBST solution containing 5% non-fat milk at 1:1000 and incubated with membranes at 4 °C overnight. HRP-conjugated sheep anti-rat secondary antibodies were diluted to 1:10,000 in TBST containing 5 % skim milk and incubated with the membranes for 1 h at room temperature. The appropriate HRP-conjugated secondary antibodies were applied, and the blots were incubated with a chemiluminescent substrate (Millipore) and exposed to Kodak X-Omat Blue Film.

ELISA assay

Tissue and plasma TGF-β was measured using a mouse TGF-β ELISA Quantitation Kit according to the manufacturer's protocol (R & D, Inc.,).

Protein assay

The protein content of the supernatant was measured by the Bradford method, using bovine serum albumin (Sigma) as the standard (Bradford, 1976).

Statistical analysis

Differences between the groups were analyzed using analysis of variance (ANOVA) followed by Dunnet's multiple comparisons test. All of the data points are presented

as the treatment groups mean ± standard deviation (SD) of the mean.

Results

Effect of DAL on renal dysfunction

As shown in Fig. 1A, 24 h urinary protein excretion of mice progressively increased after injection of DXR. On 21th day, the urinary protein of DXR-treated mice was significantly higher than that of control mice. Beginning on 28th day, the urinary protein of DXR-treated mice rapidly increase. Treatment with DAL significantly decreased urinary protein at 4 and 5 weeks. The DXR mice developed severe hyperlipidemia (plasma triglyceride: 3.63 ± 0.44 mg/ml) that was less severe in the treatment group (plasma triglyceride: 1.52 ± 0.31 mg/ml) (Fig. 1B). Treatment of mice with DXR caused a significant increase in the BUN and plasma creatinine levels by 2.3- and 4.1-fold, respectively, compared to the control group (Fig. 1C, D). Pre-treatment with DAL for 7 days resulted in the restoration of BUN and plasma creatinine to near the control levels ($p < 0.01$). So, DAL attenuates nephrotoxicity in a mice model of DXR

Effect of DAL on renal fibrosis

Like many other organ systems, the kidney stiffens after injury, a process that is increasingly recognized as an important driver of renal fibrosis (Szeto et al., 2016). To correlate the reduction of kidney injury to the effect of the drug treatments, renal fibrosis was assessed by Masson staining (Fig.2A). Renal fibrosis marker of alpha

smooth muscle actin (α-SMA), fibronectin and the epithelial cell marker of E-cadherin were assessed by western blotting (Criswell and Arteaga, 2007, Zeisberg and Neilson, 2009). Consistent with the albuminuria data, the results from the DXR mice showed marked renal fibrosis evidenced by the increased expression of fibroblasts markers (Fig.2B-D). The treatment of mice with DXR caused a significant increase in the renal protein expression of well-known fibroblasts markers, and increased the expression of E-cadherin in the renal tissue (Fig.2B-D). DAL ameliorates renal fibrosis in a mice model of DXR.

Effect of DAL on kidney redox potential

The elevated reactive oxygen species (ROS) production is one of major primary mechanism that DXR induced cytotoxicity (Zhang et al., 2009, Wang et al., 2015). And ROS is one of TGF-β1-stimulated production (Park et al., 2015). MDA and GSH are used to assess the level of ROS. In this study, there was a significant increase in MDA in the kidney of the DXR group compared to the control group ($p < 0.01$). Compared with the DXR group, the treatment with DAL in DXR-induced nephrotoxicity resulted in a significant reduction in the MDA levels (Fig. 3A). We also measured the GSH concentration as an indicator of cellular redox status in the kidney tissue to investigate the antioxidant action of DAL. After the DXR treatment, the levels of GSH were significantly depleted, as shown in Fig. 3B ($p < 0.01$). DAL administration significantly reversed GSH depletion compared with the DXR group. DAL maintains redox balance of the kidney tissue.

DAL effects of TGF-β signaling pathway

TGF-β is a key mediator in the pathogenesis of renal fibrosis and induces renal scarring largely by activating its downstream Smad signaling pathway (Lan, 2011). Although TGF-β signaling pathway is mediated by Smad2 and Smad3, Smad2 protects against TGF-beta/Smad3-mediated renal fibrosis (Meng et al., 2010). So, phosphorylated Smad3 is the effectors of the TGF-β-mediated renal fibrosis. The levels of phosphorylated Smad3 in the kidney were increased by DXR (Fig.4A). As shown in Fig.4A, the DAL-treated groups exhibited a significant decrease in the phosphorylation level of SMAD3 compared to the DXR groups ($p < 0.01$). Because Collagen I is a target gene of TGF-beta/SMAD3, the mRNA and protein expression of Collagen I in the kidney were evaluated by q-PCR and western blotting. As shown in Fig.4B, Collagen I mRNA and protein levels in the DAL-treated groups showed a significant decrease compared to those in the DXR groups. Smad7 acts as an antagonist of the TGF-β signaling pathway by preventing R-Smads from interacting with their receptors or by competing with Co-Smads for the generation of R-Smad/Co-Smad complexes (Lan et al., 2003, Meng et al., 2012). Smad7 protein levels were reduced after DXR treatment, but this effect was reversed after DAL treatment ($p < 0.01$; Fig.5A, B). DAL suppresses TGF-β signaling pathway in kidney tissue.

Effect of DAL on TGF-β protein expression

TGF-β is a protein that controls proliferation, cellular differentiation, and other functions in most cells. TGF-β is important for the induction of fibrosis and the EMT often associated with chronic phases of inflammatory diseases (Pohlers et al., 2009). As shown in Fig.6, the mRNA and protein levels of TGF-β in the DAL-treated groups showed a significant decrease compared with those in the DXR-treated groups.

Discussion

In the current study, DAL ameliorated the severe nephritic syndrome induced by DXR in mice. Urine albumin and plasma urea and creatinine are the most sensitive markers of nephrotoxicity implicated in the diagnosis of renal injury (Sallie et al., 1991, Khan and Sultana, 2004). DXR treatment significantly increased serum creatinine, BUN and hyperlipidemia. In contrast, treatment with DAL resulted in a significant decrease of these parameters in the DXR-treated animals, thus DAL may offer a considerable nephroprotective effect against DXR toxicity.

Renal fibrosis is a well-known cause of kidney failure in DXR-induced nephropathy (Van Beneden et al., 2013). Several cellular pathways, including fibroblast activation and tubular epithelial-mesenchymal transition, have been identified as the major causes of renal fibrosis conditions (Liu, 2006). In this study, the administration of DAL significantly improved renal fibrosis. One of major mechanisms in the protective action of DAL in this model is likely through the inhibition of fibroblast activation. After 35 days of DXR injection, fibronectin and α-SMA mRNA and protein were markedly up-regulated, and DAL was able to

significantly preserve their expression levels. We also examined the mRNA and protein levels of the epithelial maker E-cadherin. DAL treatment reversed the reduction of E-cadherin. Consistent with these results, histologically, DAL treatment ameliorated DXR-induced renal fibrosis.

TGF-β, which is up-regulated in some studies, plays a pivotal role in the progression of the tubular epithelial-mesenchymal transition in renal fibrosis, and therapeutic intervention targeting TGF-β has been successful and well tolerated in animal models (Liu et al., 2013, Van Beneden et al., 2013, Zhou et al., 2013). Recently, it has been postulated that ROS mediates fibrosis via a TGF-β-dependent pathway (Samarakoon et al., 2013, Montorfano et al., 2014). And also, ROS have emerged in the pathogenesis of DXR-induced nephropathy (Wu et al., 2007, Guo et al., 2008). It has been recommended that a DXR semiquinone plays a major role in DXR nephrotoxicity. Although semiquinones have a short life, they initiate a stream of reactions producing ROS after interacting with molecular oxygen (El-Shitany et al., 2008, Mohan et al., 2010). It has been shown that DXR increases the production of free radicals such as superoxide, hydroxyl radicals and hydrogen peroxide, which have a great ability to react rapidly with lipids and cause LPO (Oz and Ilhan, 2006). LPO is known to be one of the toxic manifestations of DXR ingestion and is determined by measuring MDA levels. Excessive LPO has been reported in the kidneys of DXR-treated mice (Rashid et al., 2013). In the present study, the DXR-treated mice showed increased levels of MDA compared to the control mice. GSH is the most important thiol-containing antioxidant, and it plays a pivotal role in

preventing oxidative damage (Wu et al., 2004, Moskaug et al., 2005). GSH has also been used as a biomarker of oxidative stress in biological systems (Reed and Savage, 1995). The depletion of GSH has been observed in DXR mice (Rashid et al., 2013). In our studies, DAL decreased the concentrations of MDA and increased the level of GSH. The recovering redox balance in the tissue microenvironment is the most likely mechanism by which DAL exerts nephroprotection and inhibits tubular epithelial–mesenchymal transition effects.

Smad3 is a critical downstream mediator responsible for the biological effects of TGF-β, and their related family members regulate the transcription of several hundred genes. In the context of renal fibrosis, Smad3 are strongly activated in both experimental and human kidney diseases (Lan, 2011). Phosphorylated Smad3 is increased in the DXR group. This observation indicates that TGF-β/Smad signaling pathways are activated in DXR-induced nephropathy. However, this phenomenon is reversed by DAL. Collagen I, which is fibrogenic gene, is the downstream targets of the TGF-β/Smad3 signaling pathway. DAL reversed the increase in Collagen I. Furthermore, Smad7, which is an inhibitor of TGF-β/Smad signaling pathways, was up-regulated by DAL treatment. DAL also increases the mRNA and protein expression of TGF-β in DXR-induced nephropathy.

In conclusion, our results demonstrated that DAL had a potent nephroprotective effect on the DXR mice model. The nephroprotective effect of DAL may be mediated by suppressing the TGF-β-induced renal tubular epithelial-to-mesenchymal transition. This is an early stage study of the nephroprotective effects of DAL, and the detailed

mechanisms of action need further clarification.

Acknowledgments

This work was supported by the Young Scientists Fund of the National Natural Science Foundation of China (No. 81302829)

Conflict of interest

The authors declare that they have no competing interests.

References

Bernard J (1967) Acute leukemia treatment. Cancer research 27: 2565-2569

Blum RH, Carter SK (1974) Adriamycin. A new anticancer drug with significant clinical activity. Annals of internal medicine 80: 249-259

Bradford MM (1976) A rapid and sensitive method for the quantitation of microgram quantities of protein utilizing the principle of protein-dye binding. Analytical biochemistry 72: 248-254

Carter SK (1972) Single and combination nonhormonal chemotherapy in breast cancer. Cancer 30: 1543-1555

Criswell TL, Arteaga CL (2007) Modulation of NFkappaB activity and E-cadherin by the type III transforming growth factor beta receptor regulates cell growth and motility. The Journal of biological chemistry 282: 32491-32500

Desai VG, Herman EH, Moland CL, Branham WS, Lewis SM, Davis KJ, George NI, Lee T, Kerr S, Fuscoe JC (2013) Development of doxorubicin-induced chronic cardiotoxicity in the B6C3F1 mouse model. Toxicology and applied pharmacology 266: 109-121

El-Shitany NA, El-Haggar S, El-desoky K (2008) Silymarin prevents adriamycin-induced cardiotoxicity and nephrotoxicity in rats. Food and chemical toxicology : an international journal published for the British Industrial Biological Research Association 46: 2422-2428

Guo J, Ananthakrishnan R, Qu W, Lu Y, Reiniger N, Zeng S, Ma W, Rosario R, Yan SF, Ramasamy R, D'Agati V, Schmidt AM (2008) RAGE mediates podocyte injury in adriamycin-induced glomerulosclerosis. Journal of the American Society of Nephrology : JASN 19: 961-972

Injac R, Boskovic M, Perse M, Koprivec-Furlan E, Cerar A, Djordjevic A, Strukelj B (2008) Acute doxorubicin nephrotoxicity in rats with malignant neoplasm can be successfully treated with fullerenol C60(OH)24 via suppression of oxidative stress. Pharmacological reports : PR 60: 742-749

Khan N, Sultana S (2004) Abrogation of potassium bromate-induced renal oxidative stress and subsequent cell proliferation response by soy isoflavones in Wistar rats. Toxicology 201: 173-184

Lai HC, Yeh YC, Wang LC, Ting CT, Lee WL, Lee HW, Wang KY, Wu A, Su CS, Liu TJ (2011) Propofol ameliorates doxorubicin-induced oxidative stress and cellular apoptosis in rat cardiomyocytes. Toxicology and applied pharmacology 257: 437-448

Lan HY (2011) Diverse roles of TGF-beta/Smads in renal fibrosis and inflammation. Int J Biol Sci 7: 1056-1067

Lan HY, Mu W, Tomita N, Huang XR, Li JH, Zhu HJ, Morishita R, Johnson RJ (2003) Inhibition of renal fibrosis by gene transfer of inducible Smad7 using ultrasound-microbubble system in rat UUO model. J Am Soc Nephrol 14: 1535-1548

Liu S, Jia Z, Zhou L, Liu Y, Ling H, Zhou SF, Zhang A, Du Y, Guan G, Yang T (2013) Nitro-oleic acid protects against adriamycin-induced nephropathy in mice. Am J Physiol Renal Physiol 305: F1533-1541

Liu Y (2006) Renal fibrosis: new insights into the pathogenesis and therapeutics. Kidney international 69: 213-217

Mao YW, Lin RD, Hung HC, Lee MH (2014) Stimulation of osteogenic activity in human osteoblast cells by edible Uraria crinita. J Agric Food Chem 62: 5581-5588

Meng XM, Huang XR, Chung AC, Qin W, Shao X, Igarashi P, Ju W, Bottinger EP, Lan HY (2010) Smad2 protects against TGF-beta/Smad3-mediated renal fibrosis. J Am Soc Nephrol 21: 1477-1487

Meng XM, Huang XR, Xiao J, Chung AC, Qin W, Chen HY, Lan HY (2012) Disruption of Smad4 impairs TGF-beta/Smad3 and Smad7 transcriptional regulation during renal inflammation and fibrosis in vivo and in vitro. Kidney Int 81: 266-279

Mohan M, Kamble S, Gadhi P, Kasture S (2010) Protective effect of Solanum torvum on doxorubicin-induced nephrotoxicity in rats. Food and chemical toxicology : an international journal published for the British Industrial Biological Research Association 48: 436-440

Montorfano I, Becerra A, Cerro R, Echeverria C, Saez E, Morales MG, Fernandez R, Cabello-Verrugio C, Simon F (2014) Oxidative stress mediates the conversion of endothelial cells into myofibroblasts via a TGF-beta1 and TGF-beta2-dependent pathway. Laboratory investigation; a journal of technical methods and pathology

Moskaug JO, Carlsen H, Myhrstad MC, Blomhoff R (2005) Polyphenols and glutathione synthesis regulation. The American journal of clinical nutrition 81: 277S-283S

Oz E, Ilhan MN (2006) Effects of melatonin in reducing the toxic effects of doxorubicin. Molecular and cellular biochemistry 286: 11-15

Park SA, Kim MJ, Park SY, Kim JS, Lee SJ, Woo HA, Kim DK, Nam JS, Sheen YY (2015) EW-7197 inhibits hepatic, renal, and pulmonary fibrosis by blocking TGF-beta/Smad and ROS signaling. Cell Mol Life Sci 72: 2023-2039

Pohlers D, Brenmoehl J, Loffler I, Muller CK, Leipner C, Schultze-Mosgau S, Stallmach A, Kinne RW, Wolf G (2009) TGF-beta and fibrosis in different organs - molecular pathway imprints. Biochimica et biophysica acta 1792: 746-756

Rashid S, Ali N, Nafees S, Ahmad ST, Arjumand W, Hasan SK, Sultana S (2013) Alleviation of doxorubicin-induced nephrotoxicity and hepatotoxicity by chrysin in Wistar rats. Toxicology mechanisms and methods 23: 337-345

Reed DJ, Savage MK (1995) Influence of metabolic inhibitors on mitochondrial permeability transition and glutathione status. Biochimica et biophysica acta 1271: 43-50

Sallie R, Tredger JM, Williams R (1991) Drugs and the liver. Part 1: Testing liver function. Biopharmaceutics & drug disposition 12: 251-259

Samarakoon R, Overstreet JM, Higgins PJ (2013) TGF-beta signaling in tissue fibrosis: redox controls, target genes and therapeutic opportunities. Cellular signalling 25: 264-268

Szeto SG, Narimatsu M, Lu M, He X, Sidiqi AM, Tolosa MF, Chan L, De Freitas K, Bialik JF, Majumder S, Boo S, Hinz B, Dan Q, Advani A, John R, Wrana JL, Kapus A, Yuen DA (2016) YAP/TAZ Are Mechanoregulators of TGF-beta-Smad Signaling and Renal Fibrogenesis. J Am Soc Nephrol 9: 2015050499

Van Beneden K, Geers C, Pauwels M, Mannaerts I, Wissing KM, Van den Branden C, van Grunsven LA (2013) Comparison of trichostatin A and valproic acid treatment regimens in a mouse model of kidney fibrosis. Toxicology and applied pharmacology 271: 276-284

Wang H, Chen X, Su Y, Paueksakon P, Hu W, Zhang MZ, Harris RC, Blackwell TS, Zent R, Pozzi A (2015) p47(phox) contributes to albuminuria and kidney fibrosis in mice. Kidney Int 87: 948-962

Wu G, Fang YZ, Yang S, Lupton JR, Turner ND (2004) Glutathione metabolism and its implications for health. The Journal of nutrition 134: 489-492

Wu H, Wang YM, Wang Y, Hu M, Zhang GY, Knight JF, Harris DC, Alexander SI (2007) Depletion of gammadelta T cells exacerbates murine adriamycin nephropathy. Journal of the American Society of Nephrology : JASN 18: 1180-1189

Yen G-C, Lai H-H, Chou H-Y (2001) Nitric oxide-scavenging and antioxidant effects of Uraria crinita root. Food Chemistry 74: 471-478

Yilmaz S, Atessahin A, Sahna E, Karahan I, Ozer S (2006) Protective effect of lycopene on adriamycin-induced cardiotoxicity and nephrotoxicity. Toxicology 218: 164-171

Zeisberg M, Neilson EG (2009) Biomarkers for epithelial-mesenchymal transitions. The Journal of clinical investigation 119: 1429-1437

Zhang YW, Shi J, Li YJ, Wei L (2009) Cardiomyocyte death in doxorubicin-induced cardiotoxicity. Arch Immunol Ther Exp (Warsz) 57: 435-445

Zhou L, Li Y, Zhou D, Tan RJ, Liu Y (2013) Loss of Klotho contributes to kidney injury by derepression of Wnt/beta-catenin signaling. J Am Soc Nephrol 24: 771-785

Figure Legends

Figure 1. Kidney injury at 5 weeks after DXR injection in different groups of mice as indicated. A, effect of DAL on albuminuria against DXR-induced nephrotoxicity; B, effect of DAL on hyperlipidemia against DXR-induced nephrotoxicity; C, effect of DAL on blood urea nitrogen (BUN) against DXR-induced nephrotoxicity; D, effect of DAL on serum creatinine against DXR-induced nephrotoxicity. The DXR group is compared with the normal control and DAL treatment groups. Values are statistically significant at * $p < 0.01$; the normal control is compared with the DXR and DAL treatment groups. Values are statistically significant at # $p < 0.01$.

Figure 2. Renal fibrosis at 5 weeks after DXR injection in different groups of mice

as indicated. A, kidney sections were subjected to Masson-trichrome staining; B, kidney sections expressed the mRNA of fibronectin, α-SMA, E-cadherin; C, kidney sections expressed the protein of fibronectin, α-SMA, E-cadherin in kidney sections; D, the relative protein expression of fibronectin, α-SMA, E-cadherin in the kidney sections. The DXR group is compared with the normal control and DAL treatment groups. Values are statistically significant at * $p < 0.01$; the normal control is compared with the DXR and DAL treatment groups. Values are statistically significant at # $p < 0.01$.

Figure 3. Redox microenvironment in kidney tissue at 5 weeks after DXR injection in different groups of mice as indicated. A, effect of DAL on kidney MDA levels; B, effect of DAL on kidney GSH levels. The DXR group is compared with the normal control and DAL treatment groups. Values are statistically significant at * $p < 0.01$; the normal control is compared with the DXR and DAL treatment groups. Values are statistically significant at # $p < 0.01$.

Figure 4. TGF-β signaling pathway in kidney tissue 5 weeks after DXR injection in different groups of mice as indicated. A, DAL inhibits the phosphorylation of Smad3; B, DAL inhibits the gene expression of collagen I, which is the target gene of Smad3, and DAL inhibits the protein expression of collagen I which is the target protein expression of Smad3. The DXR group is compared with the normal control and DAL treatment groups. Values are statistically significant at * $p < 0.01$; the normal control is compared with the DXR and DAL treatment groups. Values are statistically significant at # $p < 0.01$.

Figure 5. The expression of Smad7, an inhibitor of the TGF-β signaling pathway, in kidney tissue 5 weeks after DXR injection in different groups of mice as indicated. A, DAL increases the gene expression of Smad7; B, DAL increases the protein expression of Smad7. The DXR group is compared with the normal control and DAL treatment groups. Values are statistically significant at * $p < 0.01$; the normal control

is compared with the DXR and DAL treatment groups. Values are statistically significant at # $p < 0.01$.

Figure 6. The expression of TGF-β in kidney tissue 5 weeks after DXR injection in different groups of mice as indicated. A, DAL decreases the gene expression of TGF-β; B, DAL decreases the protein expression of TGF-β by ELISA; C, DAL decreases the protein expression of TGF-β, as determined by western blotting and immunofluorescence staining. Values are statistically significant at * $p < 0.01$; the normal control is compared with the DXR and DAL treatment groups. Values are statistically significant at # $p < 0.01$.

Figure

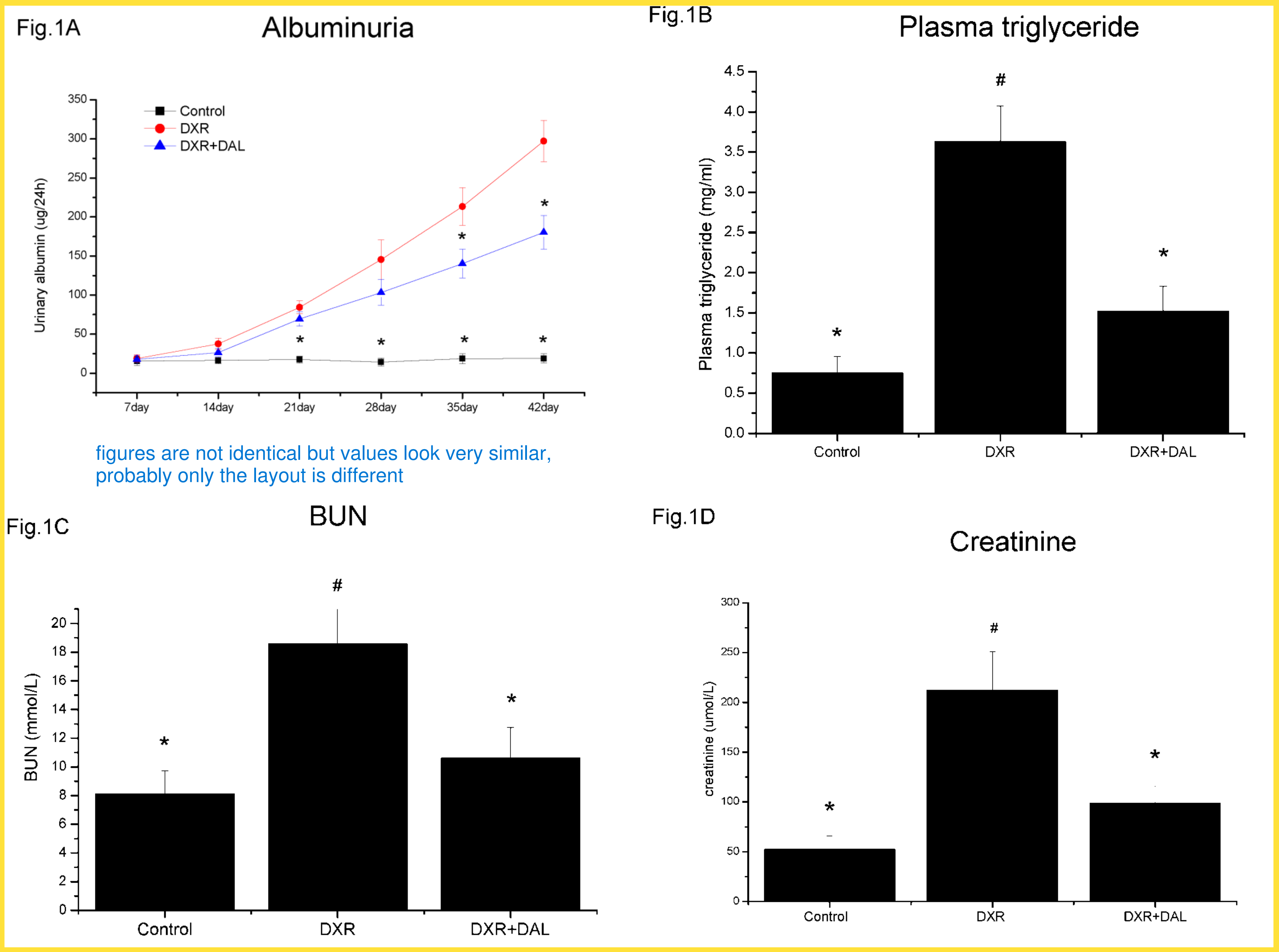

Fig.1A
Albuminuria
Urinary albumin (ug/24h)
Control
DXR
DXR+DAL
7day
14day
21day
28day
35day
42day
Fig.1B
Plasma triglyceride
Plasma triglyceride (mg/ml)
Control
DXR
DXR+DAL
figures are not identical but values look very similar,
probably only the layout is different
Fig.1C
BUN
BUN (mmol/L)
Control
DXR
DXR+DAL
Fig.1D
Creatinine
creatinine (umol/L)
Control
DXR
DXR+DAL

Fig.2 A

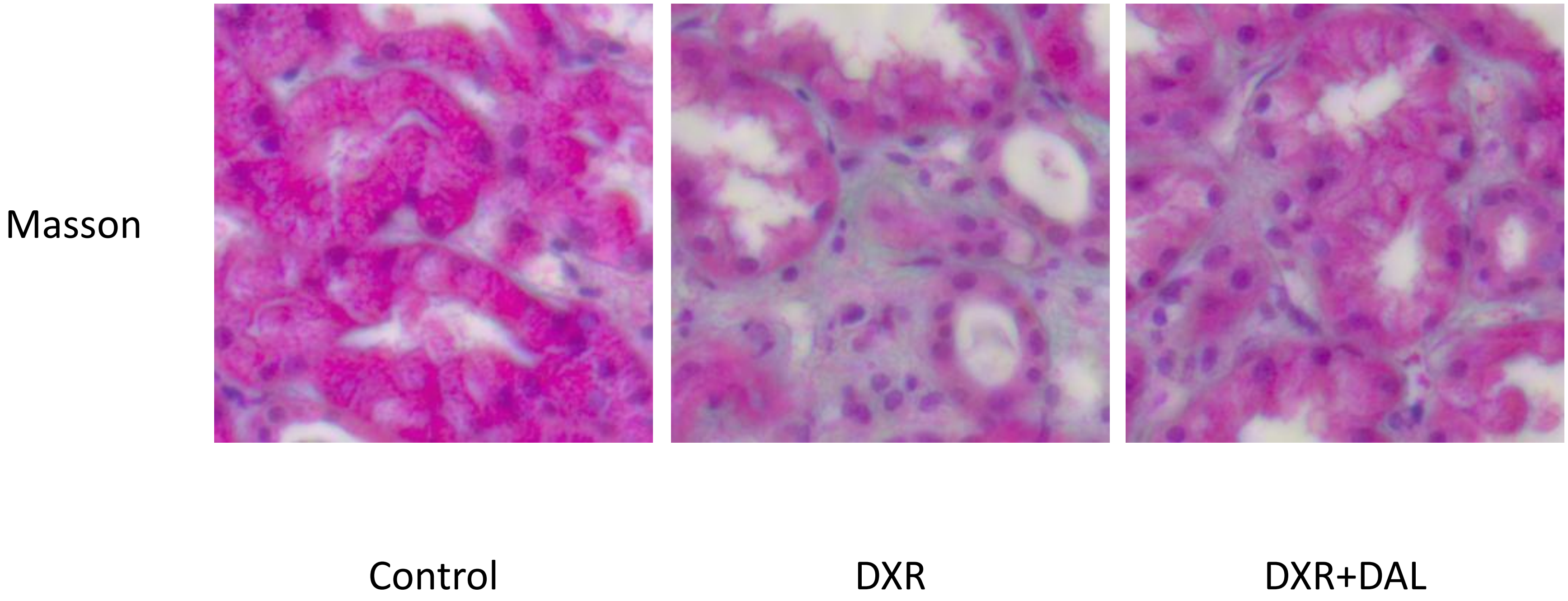

Fig.2 B

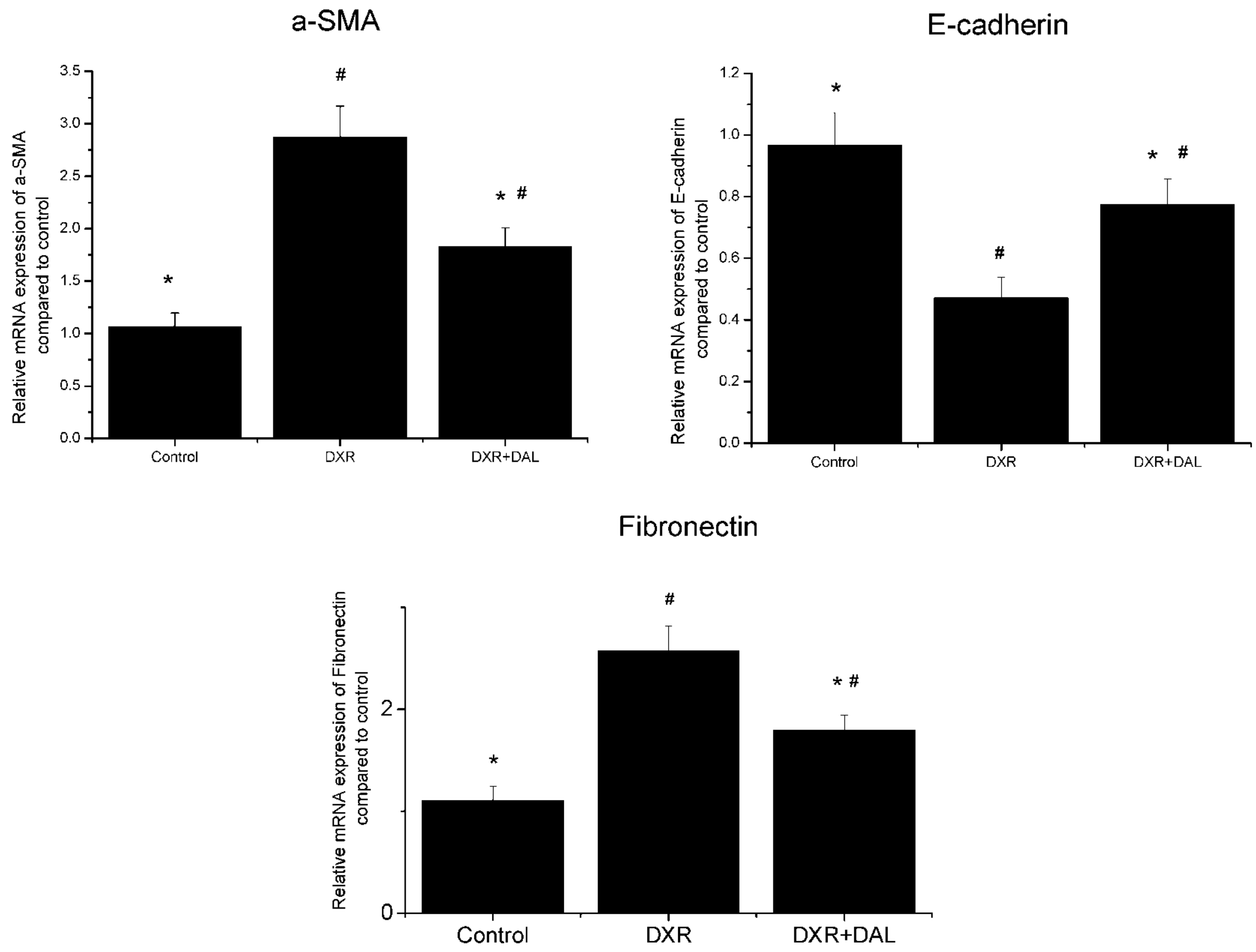
a-SMA
Relative mRNA expression of a-SMA compared to control
3.5
3.0
2.5
2.0
1.5
1.0
0.5
0.0
*
#
* #
Control
DXR
DXR+DAL
E-cadherin
Relative mRNA expression of E-cadherin compared to control
1.2
1.0
0.8
0.6
0.4
0.2
0.0
*
#
* #
Control
DXR
DXR+DAL
Fibronectin
Relative mRNA expression of Fibronectin compared to control
2
0
*
#
* #
Control
DXR
DXR+DAL

Fig.2 C

all four pictures are mirrored
vertically in the published version

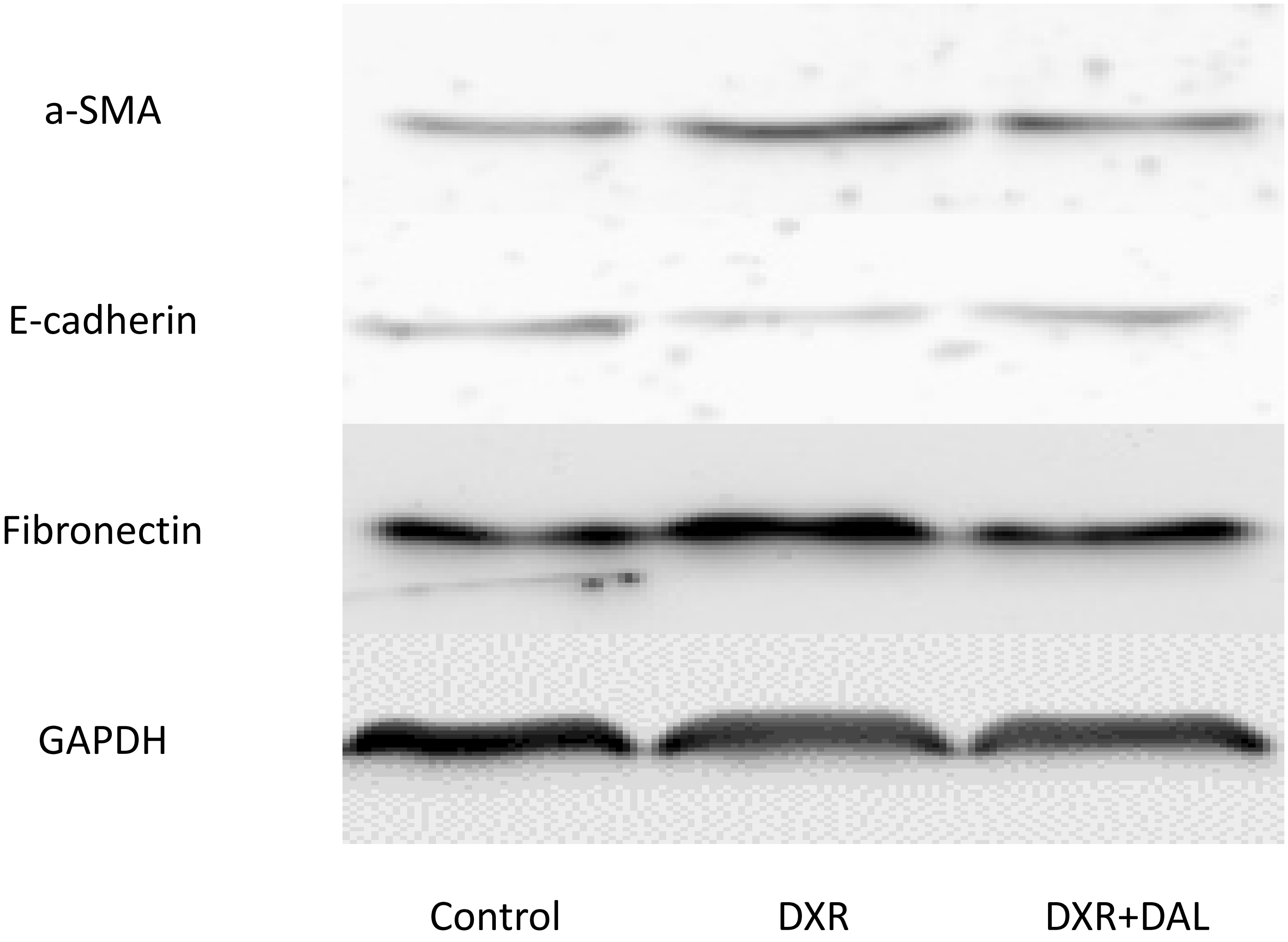

Fig.2 D

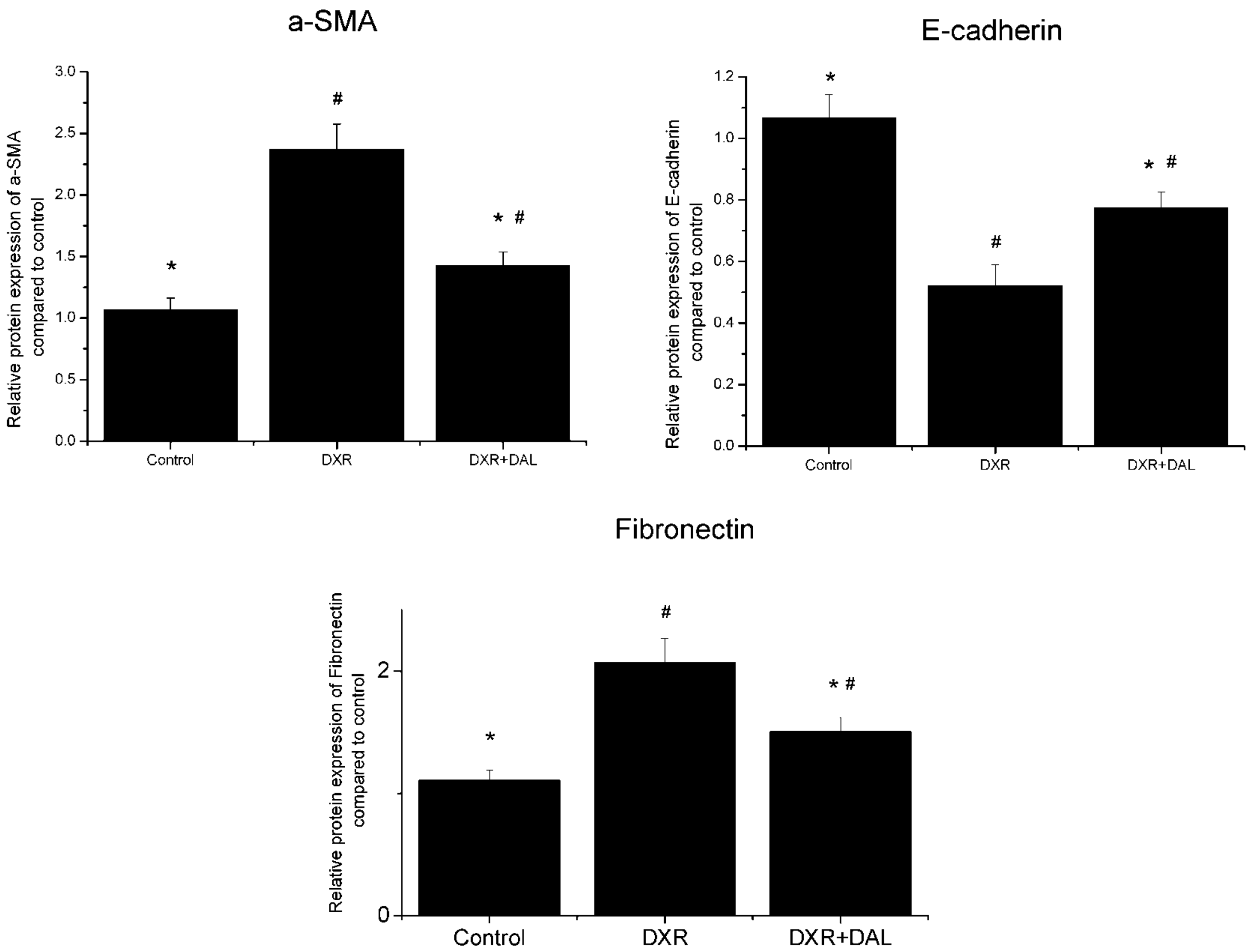
a-SMA
Relative protein expression of a-SMA compared to control
3.0
2.5
2.0
1.5
1.0
0.5
0.0
*
#
* #
Control
DXR
DXR+DAL
E-cadherin
Relative protein expression of E-cadherin compared to control
1.2
1.0
0.8
0.6
0.4
0.2
0.0
*
#
* #
Control
DXR
DXR+DAL
Fibronectin
Relative protein expression of Fibronectin compared to control
2
0
*
#
* #
Control
DXR
DXR+DAL

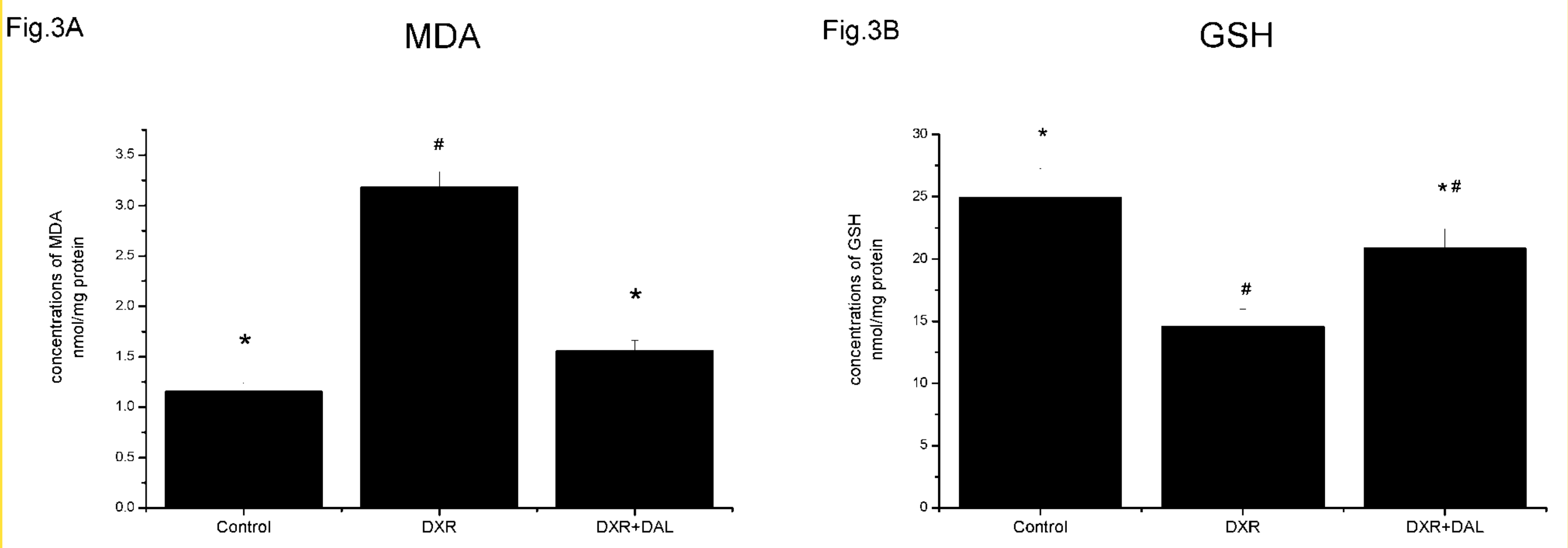
Fig.3A
MDA
concentrations of MDA nmol/mg protein
0.0
0.5
1.0
1.5
2.0
2.5
3.0
3.5
*
#
*
Control
DXR
DXR+DAL
Fig.3B
GSH
concentrations of GSH nmol/mg protein
0
5
10
15
20
25
30
*
#
*#
Control
DXR
DXR+DAL

Fig.4A

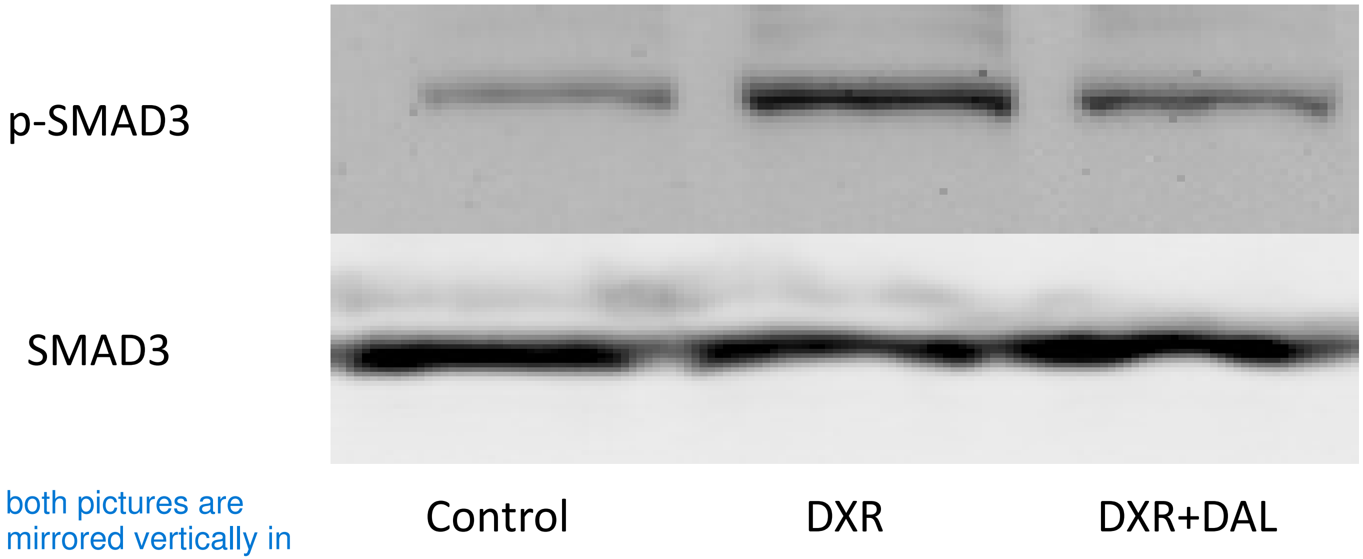

both pictures are mirrored vertically in the published version

Smad3

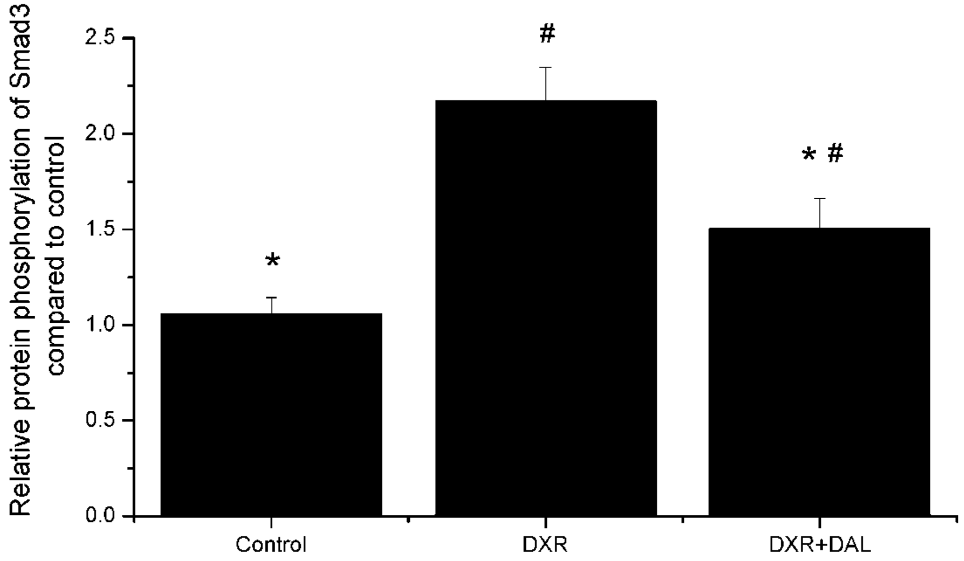

Fig.4B

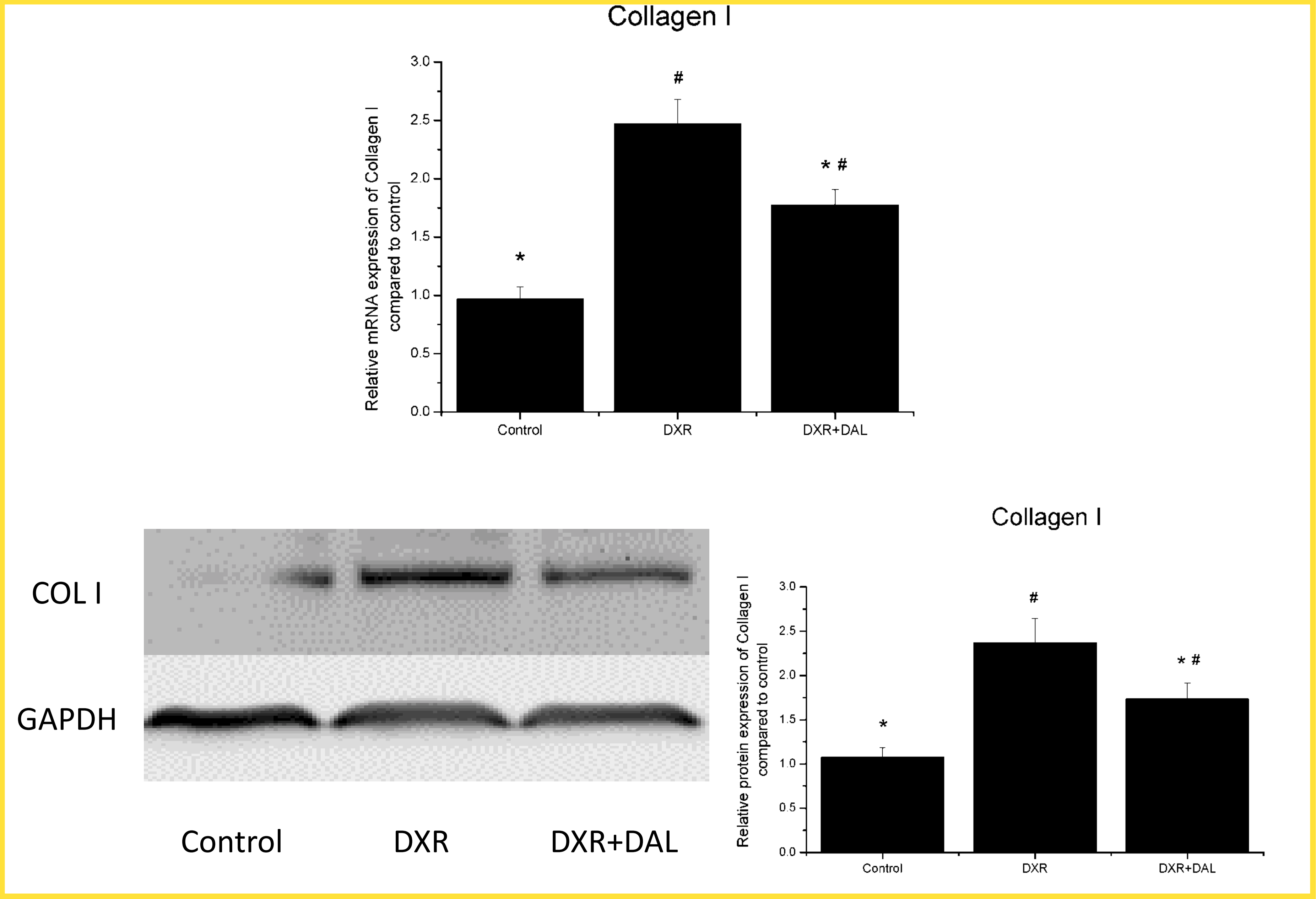
Collagen I
Relative mRNA expression of Collagen I compared to control
3.0
2.5
2.0
1.5
1.0
0.5
0.0
*
#
* #
Control
DXR
DXR+DAL
COL I
GAPDH
Control
DXR
DXR+DAL
Collagen I
Relative protein expression of Collagen I compared to control
3.0
2.5
2.0
1.5
1.0
0.5
0.0
*
#
* #
Control
DXR
DXR+DAL

Fig.5A

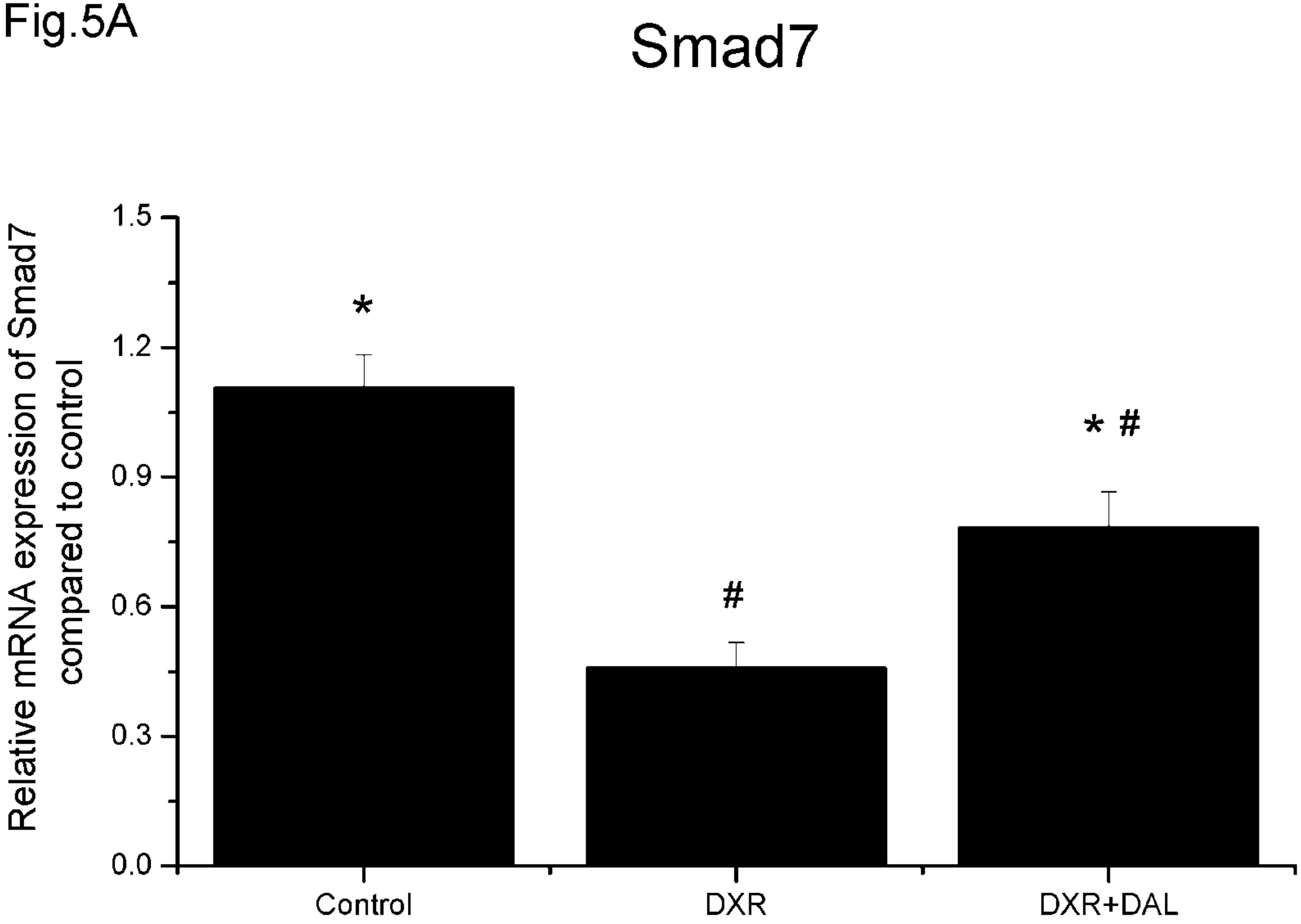

Smad7
Relative mRNA expression of Smad7
compared to control
1.5
1.2
0.9
0.6
0.3
0.0
*
#
* #
Control
DXR
DXR+DAL

Fig.5B

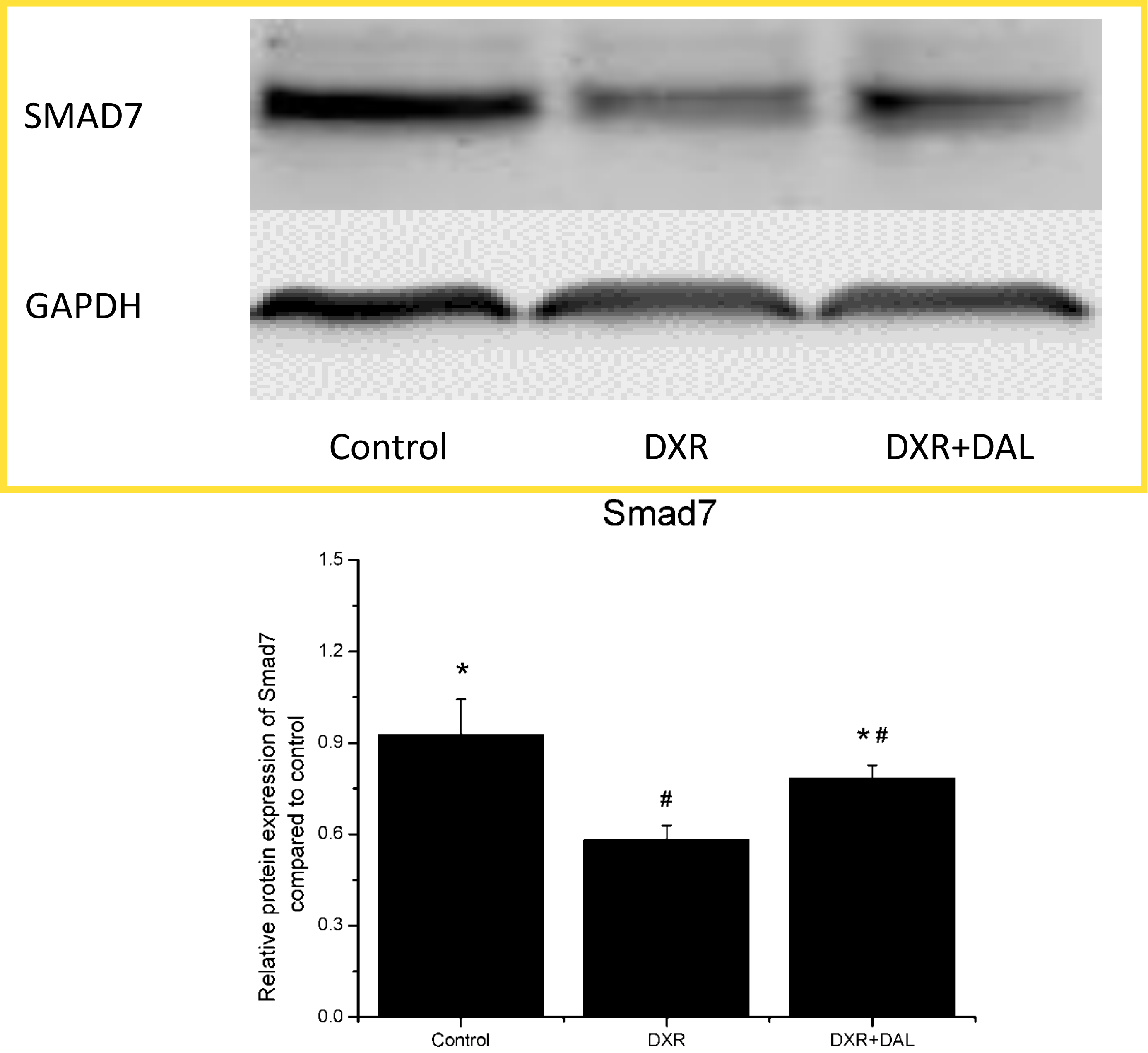
SMAD7
GAPDH
Control
DXR
DXR+DAL
Smad7
Relative protein expression of Smad7 compared to control
1.5
1.2
0.9
0.6
0.3
0.0
*
#
* #
Control
DXR
DXR+DAL

Fig.6A Fig.6B

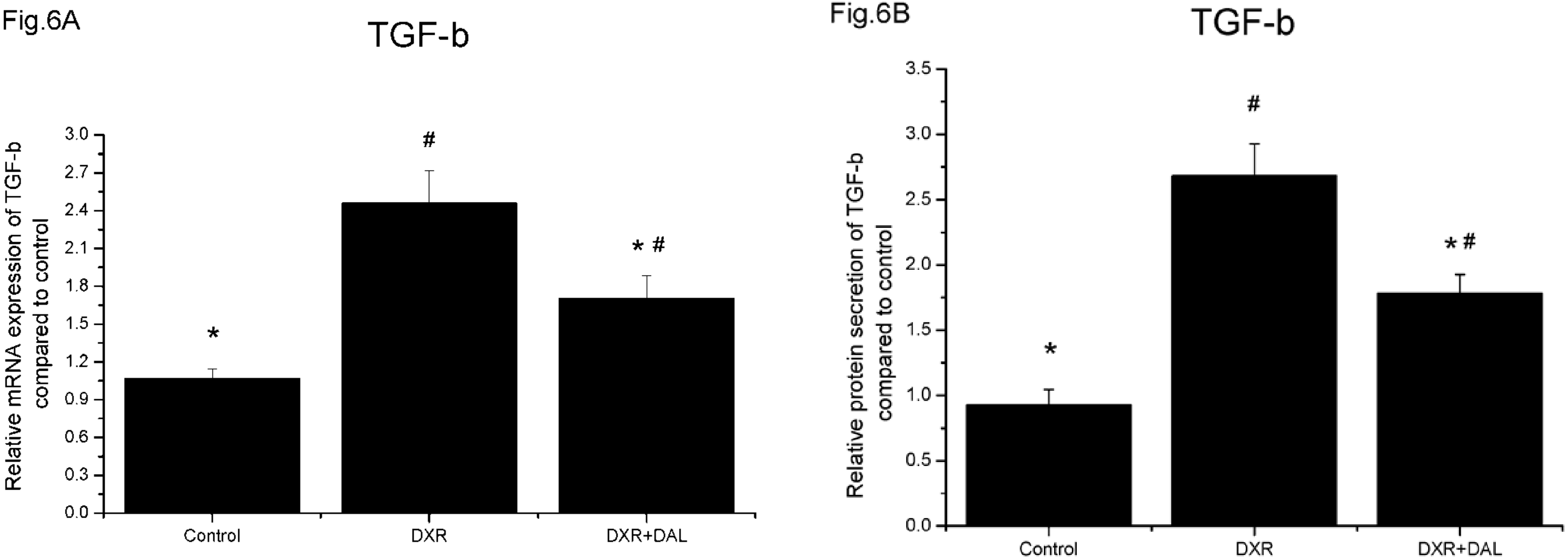
TGF-b
Relative mRNA expression of TGF-b compared to control
3.0
2.7
2.4
2.1
1.8
1.5
1.2
0.9
0.6
0.3
0.0
Control
DXR
DXR+DAL
*
#
* #
TGF-b
Relative protein secretion of TGF-b compared to control
3.5
3.0
2.5
2.0
1.5
1.0
0.5
0.0
Control
DXR
DXR+DAL
*
#
* #

Fig.6C

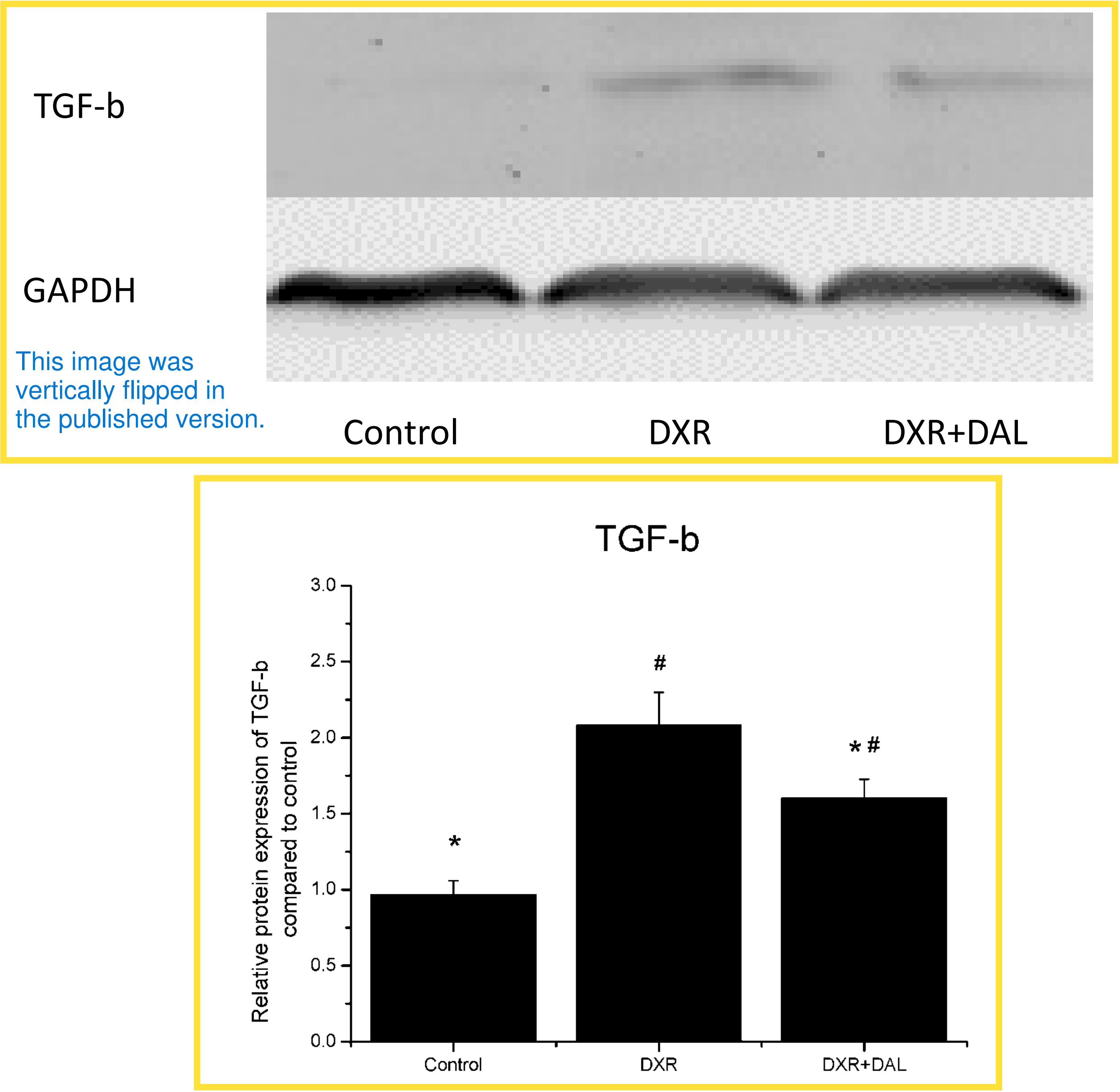
TGF-b
GAPDH
This image was vertically flipped in the published version.
Control
DXR
DXR+DAL
TGF-b
Relative protein expression of TGF-b compared to control
3.0
2.5
2.0
1.5
1.0
0.5
0.0
*
#
*#
Control
DXR
DXR+DAL
