# Supplementary material for: Fake paper identification in the pool of withdrawn and rejected manuscripts submitted to Naunyn–Schmiedeberg’s Archives of Pharmacology
Source: Naunyn Schmiedebergs Arch Pharmacol. 2023 Oct 5;397(4):2171–81. doi: 10.1007/s00210-023-02741-w (PMC10933159; doi:10.1007/s00210-023-02741-w)
Supplement: Supplementary file 6 — Supplementary file6 (PDF 969 KB) [file 210_2023_2741_MOESM6_ESM.pdf]

Figure S6

Color coding:

Yellow highlighted text	The text is identical in the NSAP version and the published version of this paper.
Red highlighted text	There are differences in the content between the NSAP version and the published version of this paper.
Blue highlighted text	The content is identical in both versions of this paper, but the text has been reworded.
Yellow bordered figure	This Figure is not identical in both versions of this paper.

Naunyn-Schmiedeberg's Archives of Pharmacology

Mycotoxin-assisted mitochondrial dysfunction: Cytotoxicity, perspection to cancer therapy

--Manuscript Draft--

Manuscript Number:	NSAP-D-17-00261
Full Title:	Mycotoxin-assisted mitochondrial dysfunction: Cytotoxicity, perspection to cancer therapy
Article Type:	Review
Corresponding Author:	Md. Torequl Islam, PhD (Ongoing)<br>Universidade Federal do Piaui<br>Teresina, Piaui BRAZIL
Corresponding Author Secondary Information:	
Corresponding Author's Institution:	Universidade Federal do Piaui
Corresponding Author's Secondary Institution:	
First Author:	Md. Torequl Islam, PhD (Ongoing)
First Author Secondary Information:	
Order of Authors:	Md. Torequl Islam, PhD (Ongoing)
Order of Authors Secondary Information:	
Funding Information:	
Abstract:	Mitochondria are the powerhouse of cells, dysfunction in which lead many diseases. Mycotoxins are the toxic secondary metabolites of fungi that are capable of causing disease and death in humans and animals. They have a versatile mechanism of actions on biological systems and can be used as lead compounds to treat some diseases, cancer is one of them. This review discusses mycotoxins having effects on mitochondria, especially those are causing mitochondrial dysfunction (MD). A search was made in some electronic databases (PubMed, Science Direct, Scopus, Web of Science, and Google Scholar) for an up-to-date published information. Findings suggest mycotoxins, such as citrinin, alfatoxin and T-2 toxin exerted multi-edged sword-like effects in test systems causing MD. Mycotoxins can induce oxidative stress, even at low concentration/dose, that may be one of the major causes of MD. On the other hand, activation of apoptotic caspases and other proteins by the mycotoxins may link in apoptotic cell deaths. Thus, mycotoxins cause MD, that may link to a number of chronic diseases and can be considered as lead compounds for inducing toxic effects in cells, derived from plants and animals; especially their cytotoxic effects on cancer cells, suggesting possible chemotherapeutic tools.
Suggested Reviewers:	

Powered by Editorial Manager® and ProduXion Manager® from Aries Systems Corporation

Manuscript Click here to download Manuscript MS.docx

Mycotoxin-assisted mitochondrial dysfunction: Cytotoxicity, perspection to cancer therapy

Muhammd Torequl Islam

Post-graduate Program in Pharmaceutical Science, Federal University of Piaui, Teresina (PI)-64.049-550, Brazil.

Correspondence E-mail: rbiotufpi.br@gmail.com

Abstract

Mitochondria are the powerhouse of cells, dysfunction in which lead many diseases. Mycotoxins are the toxic secondary metabolites of fungi that are capable of causing disease and death in humans and animals. They have a versatile mechanism of actions on biological systems and can be used as lead compounds to treat some diseases, cancer is one of them. This review discusses mycotoxins having effects on mitochondria, especially those are causing mitochondrial dysfunction (MD). A search was made in some electronic databases (*PubMed*, *Science Direct*, *Scopus*, *Web of Science*, and *Google Scholar*) for an up-to-date published information. Findings suggest mycotoxins, such as citrinin, alfatoxin and T-2 toxin exerted multi-edged sword-*like* effects in test systems causing MD. Mycotoxins can induce oxidative stress, even at low concentration/dose, that may be one of the major causes of MD. On the other hand, activation of apoptotic caspases and other proteins by the mycotoxins may link in apoptotic cell deaths. Thus, mycotoxins cause MD, that may link to a number of chronic diseases and can be considered as lead compounds for inducing toxic effects in cells, derived from plants and animals; especially their cytotoxic effects on cancer cells, suggesting possible chemotherapeutic tools.

Keywords: biotoxins; chronic diseases; cancer cells; fungal derived; mitochondria.

Introduction

Mitochondria, the key organelle of cell is responsible for the production of cellular energy, problems in which lead excess fatigue and many chronic diseases. Loss of maintenance of the electrical and chemical transmembrane potential of the inner membrane, alterations in the function of the electron transport chain, and reduction in the transport of critical metabolites into mitochondria are the three main molecular mechanisms for a reduced mitochondrial function (Nicolson 2014).

Mitochondrial dysfunction (MD), by means of losing efficiency in the electron transport chain and reductions in the synthesis of high-energy molecules, such as adenosine-5'-triphosphate (ATP), is a characteristic of aging, and essentially, of all chronic diseases (Swerdlow 2011). Neurodegenerative diseases, such as Alzheimer's disease, Parkinson's disease, Huntington's disease, amyotrophic lateral sclerosis, and Friedreich's ataxia are linked to MD (Karbowski and Neutzner 2012). Other MD-related diseases are: cardiovascular diseases, such as atherosclerosis and other heart and vascular conditions (Limongelli et al. 2012); diabetes and metabolic syndrome (Ma et al. 2012); autoimmune diseases, such as multiple sclerosis, systemic lupus erythematosus, and type 1 diabetes (Mao and Reddy 2010); neurobehavioral and psychiatric diseases, such as autism spectrum disorders, schizophrenia, and bipolar and mood disorders (Rossignol and Frye 2012); gastrointestinal disorders (Di Donato 2009); fatiguing illnesses, such as chronic fatigue syndrome and Gulf War illnesses (Norheim et al. 2011); musculoskeletal diseases, such as fibromyalgia and skeletal muscle hypertrophy/atrophy (Breeding et al. 2012); cancer (Sotgia et al. 2011); and chronic infections (Ashida et al. 2011). **Figure 1** shows MD and its related diseases in eukaryotic systems.

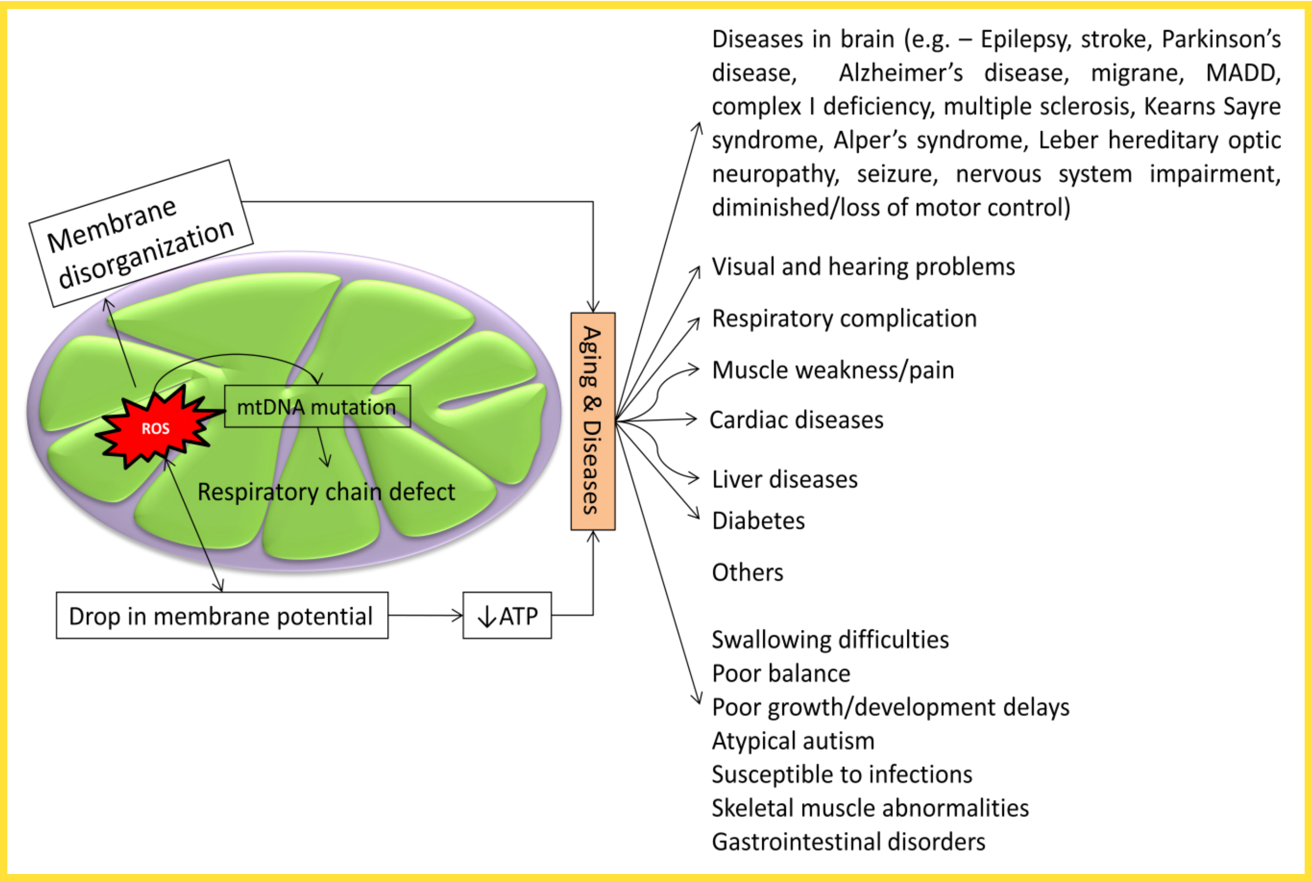

Figure 1. Mitochondrial dysfunction and related diseases.

Biotoxins, such as mycotoxins are secondary metabolites and structurally diverse group of mostly small molecular weight compounds produced by different molds or fungi that have shown serious effects on humans, animals, and crops. These are toxic to human beings and other animals, even in very low concentrations and are thought to be weakening the receiving host, thus are not necessary for the growth (Kakde 2017). Some mycotoxins are harmful to other micro-organisms; penicillin is one of them (Keller et al. 2005). Another example is zearalenone, a toxic non-steroidal mycoestrogen produced by fungi that widely contaminates agricultural products, have been evident to play an important role in the promotion of hormone-dependent tumors, particularly those arising from breast and endometrium (Pazaiti et al. 2011). Thus, these types of compounds contributions in the occurance of various types of diseases, including diseases related to mytochondrial dysfunction. This review will concentrate on mycotoxins affecting mitochondrial functions in test systems and their ultimate effects.

Methodology

To gather up-to-date scientific evidence, a search was made in the following databases: *PubMed*, *Science Direct*, *Scopus*, *Web of Science*, and *Google Scholar*. No language restriction was imposed. Published evidences (*in vitro*, *ex vivo*, and *in vivo*) with mycotoxins, and their derivatives in mitochondrial dysfunction have been considered in this study. Reports other than mycotoxin-induced mitochondrial dysfunction, data duplication, and other effects of mycotoxins are not included in this study.

Findings

Mycotoxins in mitochondrial dysfunction (MD)

In total 858 published articles were observed in the search databases. Thirty three, among them were included in this study. Overall findings have been summarized below.

In an earlier study, cytochalasin B (50 µg/mL) was found to inhibit both basal and adrenocorticortropin hormone (ACTH)-induced steroidogenesis, probably in association with particles, presumably between mitochondria and various sources of cholesterol, are prerequisite for basal steroidogenesis in the in cultured adrenocortical tumor cells (Lorenz and Mattson 1986).

Ochratoxin A from *Aspergillus ochraceus* is a nephrotoxic, hepatotoxic, teratogenic, immunotoxic and phytotoxic mycotoxin. Ochratoxin A (0.5 and 1 mM) and oosporein (0.25-1 mM) are evident to cause nephrotoxicity through either mitochondrial dysfunction or lipid peroxidation. In isolated rat renal proximal tubules, it caused MD and induced proximal tubule cell death (Aleo et al. 1991). On the other hand, in rat trachea, it caused disturbance of Ca^{2+} homeostasis, possibly by inducing toxicity through mitochondrial enzyme inhibition (Chatopadhyay et al. 2014). Moreover, in *Arabidopsis thaliana*, this mycotoxin increased ROS and caused structural damage and MD (Hao et al. 2015). In the same test system, ochratoxin A also triggered accelerated respiration, increased production of mitochondrial ROS, the opening of ROS-dependent mitochondrial permeability transition pores and a decrease in mitochondrial membrane potential as well as the release of cytochrome *c* into the cytosol (Wang et al. 2017).

Citrinin (1.0 mM) depresses the phosphorylation efficiency of rat renal cortical mitochondria, as inferred from the decrease of the respiratory control coefficient and ADP/O ratio. It inhibited almost all enzymes linked to the respiratory chain, especially, NADH oxidase and NADH cytochrome *c* reductase involved with complex I and increases the activity of succinate cytochrome *c* reductase and succinate oxidase. Malate and glutamate dehydrogenases are also inhibited. The inhibitory action of citrinin on phosphorylation efficiency could be related to the effect on complex I; the action on the ATP synthetase complex; the partial inhibition of the transmembrane potential (Chagas et al. 1992a,b). Moreover, in isolated kidney cortex and liver mitochondria, and baby hamster kidney cultured cells, this mycotoxin significantly inhibited the activity of 2-oxoglutarate and pyruvate dehydrogenases in both kidney cortex and liver mitochondria. Citrinin promoted a decrease in the velocity and in the total capacity of Ca^{2+} uptake, in both mitochondria (Chagas et al. 1995).

Moreover, citrinin, in renal cortical and liver mitochondria caused by swelling by the induction of valinomycin-K^+ complex, suggesting that it interfered mitochondrial membrane fluidity and promoted reduction of the amplitude of swelling in the presence of Na^+ ions. This alteration reflects an interference with complex I of the respiratory chain and ATP synthase complex activity without disarranging the inner mitochondrial membrane (Chagas et al. 1995). On the other hand, in rat liver cells, it increased the generation of ROS, stimulated the production of the superoxide anion ($O_2^{\bullet-}$) in the respiratory chain (Ribeiro et al. 1997). Citrinin is also evident for lipid peroxidation in mitochondria (Ribeiro et al. 1998).

T-2 toxin is the most potent trichothecene mycotoxin. In Vero cell line, it induced lipid peroxidation, decreased of macromolecular levels (protein, DNA and RNA), caused DNA fragmentation, caspase-3-dependent apoptosis, and depletion of the mitochondrial membrane potential (Bouaziz et al. 2006). In another study, both T-2 toxin (6×10^{-1} - 6×10^{-5} µM) and deoxynivalenol (0.78 - 100 µM) in rat cardiomyocytes, inhibited mitochondrial electron transport system (ETS) function and caused MD, respectively (Ngampongsa et al. 2013). Liu et al (2014) suggested that, T-2 toxin decreased chondrocytes viabilities in a concentration- and time-dependent manner. Exposure to T-2 toxin can reduce the activities of mitochondrial complexes III, IV and V, $\Delta\Psi m$ and the cellular ATP, while intracellular ROS increased following treatment with T-2 toxin. Furthermore, mitochondrial cytochrome *c* release, caspase-9 and -3 activation and chondrocytes apoptosis were also observed. Oxidative stress and MD are thought to be the main mechanism of T-2 toxin toxicity. In rat hepatocytes, T-2 toxin caused glutathione depletion, ROS overproduction and mitochondrial membrane potential collapse, abnormal caspase-3 activity and apoptosis (Moosavi et al. 2016).

On the other hand, gliotoxin, from *Aspergillus fumigatus*, a secondary metabolite, is known as cytotoxic for mammalian cells. It induced apoptotic cell death by activating the proapoptotic Bcl-2 family member Bak, but not Bax, to elicit the generation of ROS, and caused the mitochondrial release of apoptogenic factors, and activation of caspase-3 (Pardo et al. 2006).

Deoxynivalenol, also known colloquially as "vomitoxin", is a pathogenic mycotoxin produced by *Fusarium culmorum* and *Fusarium graminearum* is currently believed to play a decisive role in the fungal phytopathogenesis as a virulence factor. 0.15 mg of deoxynivalenol and 0.0035 mg zearalenone per kg diet) or a diet containing 15% of *Fusarium* toxin contaminated triticale (4.42 mg of deoxynivalenol and 0.048 mg of zearalenone per kg diet) in pregnant sows during days 35 to 70 of gestation enhanced glycogen and caused an impairment of mitochondria in liver of fetuses (Tiemann et al. 2008). Deoxynivalenol (0-25 µg/mL) in rat (Clone9 and MH1C1), mouse (NBL CL2) and human (WRL68 and HepG2) liver cells, caused cytotoxicity, oxidative stress and MD (Sahu et al. 2010). Additionally, in human colon cancer cells (HT-29) it induced apoptosis by MD and subsequent release of

cytochrome *c* into the cytoplasm and activated caspases, regulated by Bcl-2 family proteins (Ma et al. 2012). In mouse thymic epithelial cell line 1 (MTEC1), deoxynivalenol increased ROS production, and changed mitochondrial membrane potential. Increased apoptotic cell death *via* increasing the protein levels of caspase-3, -8, -9 and poly(ADP-ribose) polymerase (PARP) (Li et al. 2014). Moreover, in *Nicotiana tabacum* BY2 cultured cells, this mycotoxin induced programmed cell death, possibly *via* inducing ROS, MD and a transcriptional down-regulation of the alternative oxidase (*Aox1*) gene as well as regulation of ion channel activities participating in cell shrinkage (Yekkour et al. 2015).

Incubation of isolated rat myocardial mitochondria with butenolide of 100 µg/mL for 60 min resulted in mitochondrial swelling, indicating the occurrence of mitochondrial permeability transition and caused marked oxidative damage in the myocardial mitochondria (Wang et al. 2009), while enniatin B, from *Fusarium* sp. depleted the mitochondrial transmembrane potential, uncoupled oxidative phosphorylation, induced mitochondrial swelling and decreased mitochondrial Ca^{2+} retention capacity (Tonshin et al. 2010).

Zearalenone through oxidative phosphorylation and caused MD pathways, caused endocrine dysfunction in H295R (Busk et al. 2011). In a forty two days zearalenone intoxication on the presence of Ca^{2+} in ovarian cells from beagle bitches, caused apoptosis-*like* changes in the cells, probably *via* excess Ca^{2+} accumulation in the mitochondria, followed by the cell dysfunction and a decrease in or the absence of mitochondrial metabolic activity in oocytes, follicle and interstitial cells (Gajecka and Przybylska-Gornowicz 2012).

Aflatoxin B(1) (0.1 mg/kg body weight) significantly increased in hepatic mitochondrial complexes I-IV in group III, decreased in mitochondrial respiratory induced MD (Shi et al. 2012a). It is also evident to decrease in hepatic mitochondrial SOD, CAT, GSH-Px, GR, and mitochondrial MDA along with a significant induction of mitochondrial antioxidant dysfunction in ducklings (n = 30) (Shi et al. 2012b).

Fumonisin B1 is a *Fusarium* spp. mycotoxin that targets the liver, kidney and brain. In astrocytes and neuroblastoma cells, it inhibited mitochondrial complex I and leads to mitochondrial membrane potential depolarization and calcium deregulation (Domijan et al. 2012). In another study, in the adult male C57BL/6 mice, fumonisin B1 (8 mg/kg, i.p.) injected caused brain hyperexcitability, possibly *via* MD (Poersch et al. 2015).

Patulin, penicillic acid, mycophenolic acid, and roquefortin-C (ROQ-C), identified in *Penicillium roqueforti* were found to exert cytotoxic effects in neuro-2a cells. All of them were evident to increase ROS generation, cause mitochondrial and lysosomal dysfunction and ATP depletion (Malekinejad et al. 2015). Innate immune responses are important for pathogen elimination and adaptive immune response activation. However, excess inflammation may contribute to immunopathology and disease progression (e.g. inflammation-associated hepatocellular carcinoma). Immune modulation resulting from pattern recognition receptor-induced responses were a potential strategy for controlling immunopathology and related diseases. In a study, patulin suppresses MD, Toll-*like* receptor- and RIG-I/MAVS-dependent cytokine production through GSH depletion, the activation of p62-associated mitophagy, and p62-TRAF6 interaction (Tsai et al. 2016). Furthermore, patulin at 8 µM was found to decrease expression of ATP synthase, dstablished mitochondrial membrane potential (MMP) and reduced intracellular ATP level, accompanied by p53, Bax up-regulation and Bcl-2 down-regulation. Thereby, release of cytochrome *c* was augmented, causing caspases-9 and -3, -6 and -7 mediated apoptosis in HEK293 cells (Zhong et al. 2017). An overall finding, including molecular mechanism of actions for MD of each mycotoxin in test system has been shown in **Table 1**.

Table 1. Mycotoxin-induced mitochondrial dysfunction mechanism in test systems

Mycotoxins	Concentration/Dose (Adminstration) & Test systems	Mechanism of mitochondrial dysfunction	References
Aflatoxin B1	0.1 mg/kg (i.g.) in ducklings (n=30)	Dysfunction in hepatic mitochondrial respiratory chain complexes I–IV	Shi et al. 2012a
	0.1 mg/kg (i.g.) in ducklings (n=30)	↑oxidative stress, mitochondrial MDA; ↓SOD, CAT, GPx and GR	Shi et al. 2012b
Butenolide	10-50 µg/mL in rat cardiac cells	↑production of thiobarbituric acid reactive substances	Wang et al. 2009
Citrinin	1 mM in rat liver mitochondria	↑partial dissipation of the transmembrane potential	Chagas et al. 1992

	0.25-1.0 mM in isolated kidney cortex and liver mitochondria, and baby hamster kidney cultured cells	↓Ca^{2+} level in the mitochondrial matrix	Chagas et al. 1995
	0.25-1.0 mM in rat liver cells	↑ROS generation, especially superoxide anion	Ribeiro et al. 1997
	0.1-1.0 mM in rat liver mitochondria	Alteration of redox potential	Ribeiro et al. 1998
Deoxynivalenol	0.1-25 µg/mL in rat (Clone9 and MH1C1), mouse (NBL CL2) and human (WRL68 and HepG2) liver cells	↑oxidative stress	Sahu et al. 2010
	125-2000 ng/mL in human colon cancer cells (HT-29)	↑release of cytochrome *c*, activation of caspases and Bcl-2 family proteins	Ma et al. 2012
	500-2000 ng/mL in mouse thymic epithelial cell line 1 (MTEC1)	Activation of p53; ↑ROS	Li et al. 2014
	50 µg/mL in cultured cells of Nicotiana tabacum BY2	↑ROS generation	Yekkour et al. 2015
Deoxynivalenol and Zearalenone	0.15 mg and 0.0035 mg (dietary), respectively in pregnant sows (n=6)	Impairment of mitochondria	Tiemann et al. 2008
Enniatin B, a mixture of enniatin homologues (A, A1, B, B1) and beauvericin	1-6 µg/mL in human neural (Paju) and murine insulinoma (Min-6) cells	Depletion of mitochondrial membrane potential, efflux of the cytosolic K+ ions	Tonshin et al. 2010
Fumonisin B1	0.5 in µM astrocytes and neuroblastoma cells	Inhibited mitochondrial complex I and leads to mitochondrial membrane potential depolarization and calcium deregulation	Domijan et al. 2012
	8 mg/kg (i.p.) in male C57BL/6 mice	Cnaging of Na^+, K^+-ATPase activity	Poersch et al. 2015
Gliotoxin	0.1-100 µM in mouse cells	↑release of cytochrome *c*	Pardo et al. 2006
Ochratoxin-B	1x 10^{-6} M in rat trachea	Inhibition of mitochondrial enzyme	Chatopadhyay et al. 2014
Aspergillus ochraceus (contains ochratoxin A)	*Arabidopsis thaliana*	↑ROS accumulation	Hao et al. 2015
Ochratoxin A	0.01, 0.25 mM in *Arabidopsis thaliana*	↑ROS generation	Wang et al. 2017
Patulin, penicillic acid, mycophenolic acid, and roquefortin-C	1-100 µM in neuro-2a cells	↑intracellular ATP depletion and ROS generation	Malekinejad et al. 2015
Patulin	1-100 µM in mouse macrophage RAW264.7, HEK293T, and Human hepatocyte NeHepLxHT cells	Suppression of Toll-like receptor (TLR)- and RIG-I/MAVS-dependent cytokine production through GSH depletion, activation of p62-associated mitophagy and p62-TRAF6 interaction	Tsai et al. 2016
	8 µM in HEK293 cells	↑intracellular ROS	Zhong et al. 2017

T-2 toxin and deoxynivalenol	6×10^{-1} - 6×10^{-5} µM and 0.78 - 100 µM, respectively in rat cardiomyocytes	↓mitochondrial ETS function	Ngampongsa et al. 2013
T-2 toxin	20-60 nM in Vero cells	Activation of caspase-3 and depletion of the mitochondrial membrane potential	Bouaziz et al. 2006
	1-100 ng/mL in human chondrocytes	↓activities of mitochondrial complexes III, IV and V, DΨm and the cellular ATP; ↑ ROS generation and apoptotic caspases	Liu et al. 2014
	100-800 ng/mL in rat hepatocytes	↑ ROS generation and apoptotic caspases	Moosavi et al. 2016
Zearalenone	10 µM in H295R cells	↓oxidative phosphorylation	Busk et al. 2011
ATP – adenosine triphosphate, Bcl-2 - B-cell lymphoma 2, CAT – catalase, GPx - glutathione peroxidase, GR - glutathione reductase, i.g. – intragastrically, i.p. – intraperitoneally, MDA – malondialdehyde, RNS – reactive nitrogen species, ROS – reactive oxygen species, SOD – superoxide dismutase			

Discussion

The ability of cells to produce almost all high-energy molecules like- ATP is directly related to the ability of mitochondria. Mitochondrial dysfunction (MD) arises from an inadequate number of mitochondria, an inability to provide necessary substrates to mitochondria, or a dysfunction in their electron transport and ATP-synthesis machinery (Lee et al. 2012). Citrinin (Chagas et al. 1992a,b, 1995), T-2 toxin (Liu et al. 2014) and patulin (Zhong et al. 2017) were found to act in this pathway.

A consequence of the electron transport process is the production of reactive oxygen species (ROS), that are produced as a by-product of oxidative phosphorylation. Mitochondria are the main sources of ROS and the related reactive nitrogen species (RNS), and these can damage cellular lipids, proteins, and DNA (Stadtman 2002). Oosporein (Aleo et al. 1991), citrinin (Ribeiro et al., 1997 1998), ochratoxin A (Aleo et al. 1991; Hao et al. 2015; wang et al. 2017), T-2 toxin (Moosavi et al. 2016), gilotoxin (Pardo et al. 2006) and deoxynivalenol (Li et al. 2014; Yekkour et al. 2015) were found to augment ROS, leading to cause MD *via* increasing oxidative stress in the test systems. Accumulation of ROS and down-regulation of physiological antioxidant have a dual effect of oxidative stress. Aflatoxin B(1) was found to act through this way in ducklings (Shi et al. 2012a,b). Molecular pathways of MD and cellular events has been shown in **Figure 2**.

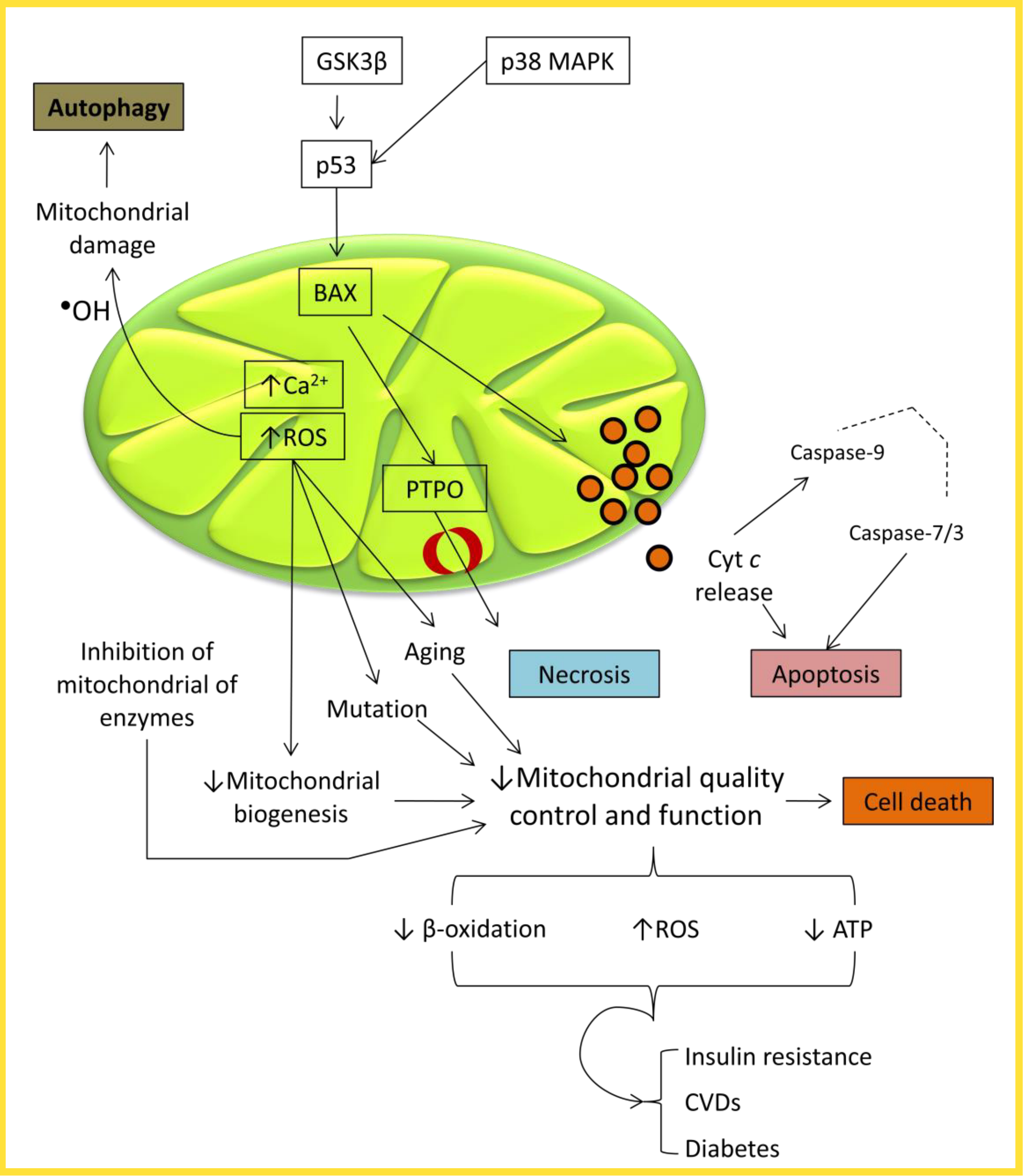

Figure 2. Molecular pathways of mitochondrial dysfunction and cellular events. [ATP – adinosine triphosphate, BAX - Bcl-2-associated X protein, CVDs – cardiovascular diseases, GSK3β - glycogen synthase kinase-3 beta, MAPK - mitogen-activated protein kinase, PTPO - permeability transition pore, ROS – reactive oxygen species]

The electron transport process can also induce uncoupling proteins, resulting in a controlled leak of protons back across the proton gradient of the inner mitochondrial membrane into the mitochondrial matrix (Nicholls 2010). This leak results in reduced ATP production while it still consumes excess oxygen (Duchen and Szabadkai 2010). In the presence of a controlled proton leak, excess oxygen consumption and the resulting ROS production can result in inappropriate damage to mitochondrial membrane lipids, such as the very ROS/RNS-sensitive cardiolipin, an inner mitochondrial membrane phospholipid (Spiteller 2010). Disturbance in the mitochondrial matrix and membrane is also evident with the mytotoxins – citrinin (Chagas et al. 1995), butenolide (Wang etal. 2009; Tonshin et al. 2010), fumonisin B1 (Domijan et al. 2012) and patulin (Zhong et al. 2017).

MD is directly related to excess fatigue, considered as a multidimensional sensation that is perceived to be a loss of overall energy and an inability to perform even simple tasks without exertion (Nicolson and Settineri 2011).

Generally, moderate to severe fatigue involves cellular energy systems and are related to loss of mitochondrial function and diminished production of ATP (Booth et al. 2012). In aging and chronic diseases, oxidative damage to mitochondrial membranes impairs mitochondrial function (Huang and Manton 2004). Almost all the mycotoxins shown in this review were found to exert cytotoxic effects in test systems through probable pathways that may cause MD.

In a recent study, citrinin at 0.1 to 1000 nM has been reported for neuroprotective property by abolishing glutamate-induced excitotoxic effects in rat cortical neurons (Nakajima et al. 2016). In a previous study, citrinin at 0.625-40 µM, showed an anti-inflammatory potential by inhibiting lipopolysaccharide-induced nitric oxide (NO) synthesis *via* downregulation of inducible NO synthase (iNOS) expression in RAW264 cells (Sugiyama et al. 2013). From this review, it is possible to say that mycotoxins are potent ROS inducer. This behavior is common for strong antioxidants-mediated pro-oxidative effects (Islam et al. 2017). At high concentration/doses such antioxidants can induce pro-oxidative effects in test systems, like- vitamin C, retonoic acid and its derivatives, antioxidant polyphenols and other essential oils, and so on.

Conclusions

Mycotoxins are not only for killing other microorganisms but also cancer cells in plants and animals. Multi-edged sword like mechanisms and strong effects in test systems suggest that mycotoxins can be a potential target in the prevention and treatment of cancers. Like some established chemotherapeutic agents, mycotoxins can be used in combination in killing cells.

Targeting mitochondria in the treatment is one of the best choices, especially in cancer therapy. Moreover, the option mitophagy is a known phenomena in distroying malfunctioning mitochondria in cells, which can be targeted mitochondria in the cancer cells.

Taken into together, mycotoxins are the lead compounds to induce toxicity in biological systems and can be targeted in cancer therapy. However, these are essentially known as toxins for biological systems. Therefore, more researche are appreciated to evaluate their safety potentials before considering them as chemotherapeutic agents.

Conflicts of interest

None declared.

Acknoledgement

I am owed to the PNPD/CAPES, Post-graduate Program in Pharmaceutical Science, Federal University of Piaui, Brazil for funding and hosting of this project.

References

Aleo MD, Wyatt RD, Schnellmann RG (1991) Mitochondrial dysfunction is an early event in ochratoxin A but not oosporein toxicity to rat renal proximal tubules. Toxicol Appl Pharmacol 107(1):73-80. PMID: 1987662.

Ashida H, Mimuro H, Ogawa M, Kobayashi T, Sanada T, Kim M, Sasakawa C (2011) Cell death and infection: a doubleedged sword for host and pathogen survival. J Cell Biol 195(6):931-942. doi: 10.1083/jcb.201108081.

Booth NE, Myhill S, McLaren-Howard J (2012) Mitochondrial dysfunction and the pathophysiology of Myalgic Encephalomyelitis/Chronic Fatigue Syndrome (ME/CFS). Int J Clin Esp Med 5(3):208-220. PMID: 22837795.

Bouaziz C, Abid-Essefi S, Bouslimi A, El Golli E, Bacha H (2006) Cytotoxicity and related effects of T-2 toxin on cultured Vero cells. Toxicon 48(3):343-352. doi: 10.1016/j.toxicon.2006.06.004.

Breeding PC, Russell NC, Nicolson GL (2012) Integrative model of chronically activated immune-hormonal pathways important in the generation of fibromyalgia. Br J Med Pract 5(3):a524-a534.

Browne SE, Beal MF (2002) Toxin-induced mitochondrial dysfunction. Int Rev Neurobiol 53:243-279. PMID: 12512343.

Busk ØL, Ndossi D, Verhaegen S, Connolly L, Eriksen G, Ropstad E, Sørlie M (2011) Relative quantification of the proteomic changes associated with the mycotoxin zearalenone in the H295R steroidogenesis model. Toxicon 58(6-7):533-542. doi: 10.1016/j.toxicon.2011.08.015.

Chagas GM, Campello AP, Klüppel ML (1992b) Mechanism of citrinin-induced dysfunction of mitochondria. I. Effects on respiration, enzyme activities and membrane potential of renal cortical mitochondria. J Appl Toxicol 12(2):123-129. PMID: 1556379.

Chagas GM, Oliveira BM, Campello AP, Klüppel ML (1992a) Mechanism of citrinin-induced dysfunction of mitochondria. II. Effect on respiration, enzyme activities, and membrane potential of liver mitochondria. Cell Biochem Funct 10(3):209-216. doi: 10.1002/cbf.290100311.

Chagas GM, Oliveira MA, Campello AP, Kluppel ML (1995) Mechanism of citrinin-induced dysfunction of mitochondria. IV$^-$-Effect on Ca^{2+} transport. Cell Biochem Funct 13(1):53-59. doi: 10.1002/cbf.290130110.

Chagas GM, Oliveira MB, Campello AP, Klüppel ML (1995) Mechanism of citrinin-induced dysfunction of mitochondria. III. Effects on renal cortical and liver mitochondrial swelling. J Appl Toxicol 15(2):91-95. PMID: 7782564.

Chatopadhyay P, Tariang B, Agnihotri A, Veer V (2014) Synergism of ochratoxin B and calcium-channel antagonist verapamil caused mitochondrial dysfunction. Toxicol Mech Meth 24(6):428-32. doi: 10.3109/15376516.2014.936543.

Di Donato S (2009) Multisystem manifestations of mitochondrial disorders. J Neurol 256(5):693-710. doi: 10.1007/s00415-009-5028-3.

Domijan AM, Kovac S, Abramov AY (2012) Impact of fumonisin B1 on glutamate toxicity and low magnesium-induced seizure activity in neuronal primary culture. Neurosci 202:10-16. doi: 10.1016/j.neuroscience.2011.12.005.

Domijan A-M, Kovac S, Abramov Ay (2012) Impact of fumonisin b1 on glutamate toxicity and low magnesium-induced seizure activity in neuronal primary culture. Neurosci 202(2012):10-16. doi:10.1016/j.neuroscience.2011.12.005.

Duchen MR, Szabadkai G (2010) Roles of mitochondria in human disease. Essays Biochem 47:115-137. PMID: 20533904.

Gajecka M, Przybylska-Gornowicz B (2012) The low doses effect of experimental zearalenone (ZEN) intoxication on the presence of Ca^{2+} in selected ovarian cells from pre-pubertal bitches. Pol J Vet Sci 15(4):711-720. PMID: 23390761.

Hao J, Wu W, Wang Y, Yang Z, Liu Y, Lv Y, Zhai Y, Yang J, Liang Z, Huang K, Xu W (2015) *Arabidopsis thaliana* defense response to the ochratoxin A-producing strain (Aspergillus ochraceus 3.4412). Plant Cell Rep 34(5):705-719. doi: 10.1007/s00299-014-1731-3.

Huang H, Manton KG (2004) The role of oxidative damage in mitochondria during aging: a review. Front Biosci 9:1100-1117. PMID: 14977532.

Islam MT, Streck L, Alencar MVOB, Silva SWC, Machado KC, Machado KC, Júnior ALG, Paz MFCJ, Mata AMOF, Sousa JMC, Junior JSC, Rolim HML, Silva-Junior AA, Melo-Cavalcante AAC (2017) Evaluation of toxic, cytotoxic and genotoxic effects of phytol and its nanoemulsion. Chemosphere 177(2017):93-101. doi: 10.1016/j.chemosphere.2017.02.145.

Kakde UB (2017) Mycotoxins and its Impact on Human Populations. MOJ Bioequiv Bioavailab 3:00048.

Karbowski M, Neutzner A (2012) Neurodegeneration as a consequence of failed mitochondrial maintenance. Acta Neuropathol 123(2):157-171. doi: 10.1007/s00401-011-0921-0.

Keller NP, Turner G, Bennett JW (2005) Fungal secondary metabolism-from biochemistry to genomics. Nat Rev Microbiol 3:937-947. doi: 10.1038/nrmicro1286.

Lee J, Giordano S, Zhang J (2012) Autophagy, mitochondria and oxidative stress: cross-talk and redox signaling. Biochem J 441(2):523-540. doi: 10.1042/BJ20111451.

Li D, Ma H, Ye Y, Ji C, Tang X, Ouyang D, Chen J, Li Y, Ma Y (2014) Deoxynivalenol induces apoptosis in mouse thymic epithelial cells through mitochondria-mediated pathway. Environ Toxicol Pharmacol 38(1):163-171. doi: 10.1016/j.etap.2014.05.015.

Limongelli G, Masarone D, D'Alessandro R, Elliott PM (2012) Mitochondrial diseases and the heart: an overview of molecular basis, diagnosis, treatment and clinical course. Future Cardiol 8(1):71-88. doi: 10.2217/fca.11.79.

Liu J, Wang L, Guo X, Pang Q, Wu S, Wu C, Xu P, Bai Y (2014) The role of mitochondria in T-2 toxin-induced human chondrocytes apoptosis. PLoS One 9(9):e108394. doi: 10.1371/journal.pone.0108394.

Lorenz S, Mattson P (1986) Cinemicrographic observations of cultured adrenocortical tumor cells. Dynamic responses to ACTH and cytochalasin B. Virchows Arch B Cell Pathol Incl Mol Pathol 52(3):221-236. PMID: 2879379.

Ma Y, Zhang A, Shi Z, He C, Ding J, Wang X, Ma J, Zhang H (2012) A mitochondria-mediated apoptotic pathway induced by deoxynivalenol in human colon cancer cells. Toxicol In Vitro 26(3):414-420. doi: 10.1016/j.tiv.2012.01.010.

Ma ZA, Zhao Z, Turk J (2012) Mitochondrial dysfunction and beta-cell failure in type 2 diabetes mellitus. Exp Diabetes Res 2012:703538. doi:10.1155/2012/703538.

Malekinejad H, Aghazadeh-Attari J, Rezabakhsh A, Sattari M, Ghasemsoltani-Momtaz B (2015) Neurotoxicity of mycotoxins produced in vitro by Penicillium roqueforti isolated from maize and grass silage. Hum Exp Toxicol 34(10):997-1005. doi: 10.1177/0960327114565493.

Mao P, Reddy PH (2010) Is multiple sclerosis a mitochondrial disease? Biochim Biophys Acta 1802(1):66-79. doi: 10.1016/j.bbadis.2009.07.002.

Moosavi M, Rezaei M, Kalantari H, Behfar A, Varnaseri G (2016) l-carnitine protects rat hepatocytes from oxidative stress induced by T-2 toxin. Drug Chem Toxicol 39(4):445-450. doi: 10.3109/01480545.2016.1141423.

Nakajima Y, Iguchi H, Kamisuki S, Sugawara F, Furuichi T, Shinoda Y (2016) Low doses of the mycotoxin citrinin protect cortical neurons against glutamate-induced. J Toxicol Sci 41(2):311-319. doi: 10.2131/jts.41.311.

Ngampongsa S, Hanafusa M, Ando K, Ito K, Kuwahara M, Yamamoto Y, Yamashita M, Tsuru Y, Tsubone H (2013) Toxic effects of T-2 toxin and deoxynivalenol on the mitochondrial electron transport system of cardiomyocytes in rats. J Toxicol Sci 38(3):495-502. PMID: 23719927.

Nicholls DG (2010) Mitochondrial ion circuits. Essays Biochem 47:25-35. doi: 10.1042/bse0470025.

Nicolson GL (2014) Mitochondrial Dysfunction and Chronic Disease: Treatment With Natural Supplements. Integrat Med 13(4):35-43. PMCID: PMC4566449.

Nicolson GL, Settineri R (2011) Lipid Replacement Therapy: a functional food approach with new formulations for reducing cellular oxidative damage, cancer-associated fatigue and the adverse effects of cancer therapy. Funct Foods Health Dis 1(4):135-160. doi: 10.1007/s10555-010-9245-0.

Norheim KB, Jonsson G, Omdal R (2011) Biological mechanisms of chronic fatigue. Rheumatol (Oxford) 50(6):1009-1018. doi: 10.1093/rheumatology/keq454.

Pardo J, Urban C, Galvez EM, Ekert PG, Müller U, Kwon-Chung J, Lobigs M, Müllbacher A, Wallich R, Borner C, Simon MM (2006) The mitochondrial protein Bak is pivotal for gliotoxin-induced apoptosis and a critical host factor of *Aspergillus fumigatus* virulence in mice. J Cell Biol 174(4):509-519. doi: 10.1083/jcb.200604044.

Pazaiti A, Kontos M, Fentiman IS (2011) ZEN and the art of breast health maintenance. Int J Clin Pract 66:28-36. doi: 10.1111/j.1742-1241.2011.02805.x.

Poersch AB, Trombetta F, Souto NS, de Oliveira Lima C, Braga AC, Dobrachinski F, Ribeiro LR, Soares FA, Fighera MR, Royes LF, Oliveira MS, Furian AF (2015) Fumonisin B1 facilitates seizures induced by pentylenetetrazol in mice. Neurotoxicol Teratol 51:61-67. doi: 10.1016/j.ntt.2015.08.007.

Ribeiro SM, Campello AP, Chagas GM, Klüppel ML (1998) Mechanism of citrinin-induced dysfunction of mitochondria. VI. Effect on iron-induced lipid peroxidation of rat liver mitochondria and microsomes. Cell Biochem Funct 16(1):15-20. doi: 10.1002/(SICI)1099-0844(199803)16:1<15::AID-CBF756>3.0.CO;2-P.

Ribeiro SM, Chagas GM, Campello AP, Klüppel ML (1997) Mechanism of citrinin-induced dysfunction of mitochondria. V. Effect on the homeostasis of the reactive oxygen species. Cell Biochem Funct 15(3):203-209. doi: 10.1002/(SICI)1099-0844(199709)15:3<203::AID-CBF742>3.0.CO;2-J.

Rossignol DA, Frye RE (2012) Mitochondrial dysfunction in autism spectrum disorders: a systematic review and meta-analysis. Mol Psychiatr 17(3):290-314. doi: 10.1038/mp.2010.136.

Sahu SC, O'Donnell MW Jr, Wiesenfeld PL (2010) Comparative hepatotoxicity of deoxynivalenol in rat, mouse and human liver cells in culture. J Appl Toxicol 30(6):566-573. doi: 10.1002/jat.1527.

Shi D, Guo S, Liao S, Su R, Guo M, Liu N, Li P, Tang Z (2012a) Protection of selenium on hepatic mitochondrial respiratory control ratio and respiratory chain complex activities in ducklings intoxicated with aflatoxin B_1. Biol Trace Elem Res 145(3):312-317. doi: 10.1007/s12011-011-9195-6.

Shi D, Guo S, Liao S, Su R, Pan J, Lin Y, Tang Z (2012b) Influence of selenium on hepatic mitochondrial antioxidant capacity in ducklings intoxicated with aflatoxin B_1. Biol Trace Elem Res 145(3):325-329. doi: 10.1007/s12011-011-9201-z.

Sotgia F, Martinez-Outschoorn UE, Lisanti MP (2011) Mitochondrial oxidative stress drives tumor progression and metastasis: should we use antioxidants as a key component of cancer treatment and prevention? BMC Med 9:62-67. doi: 10.1186/1741-7015-9-62.

Spiteller G (2010) Is lipid peroxidation of polyunsaturated acids the only source of free radicals that induce aging and age-related diseases? Rejuvenation Res 13(1):91-103. doi: 10.1089/rej.2009.0934.

Stadtman E (2002) Introduction to serial reviews on oxidatively modified proteins in aging and disease. Free Radic Biol Med 32(9):789. PMID: 11978481.

Sugiyama K-I, Yamazaki R, Kinoshita M, Kamata Y, Tani F, Minai Y, Sugita-Konishi Y (2013) Inhibitory effect of citrinin on lipopolisaccharide-induced nitric oxide production by mouse macrophage cells. Mycotoxin Res 29(4):229-234. doi: 10.1007/s12550-013-0175-x.

Swerdlow RH (2011) Brain aging, Alzheimer's disease, and mitochondria. Biochim Biophys Acta 1812(12):1630-1639. doi: 10.1016/j.bbadis.2011.08.012.

Tiemann U, Brüssow KP, Dannenberger D, Jonas L, Pöhland R, Jäger K, Dänicke S, Hagemann E (2008) The effect of feeding a diet naturally contaminated with deoxynivalenol (DON) and zearalenone (ZON) on the spleen and liver of sow and fetus from day 35 to 70 of gestation. Toxicol Lett 179(3):113-117. doi: 10.1016/j.toxlet.2008.04.016.

Tonshin AA, Teplova VV, Andersson MA, Salkinoja-Salonen MS (2010) The Fusarium mycotoxins enniatins and beauvericin cause mitochondrial dysfunction by affecting the mitochondrial volume regulation, oxidative phosphorylation and ion homeostasis. Toxicol 276(1):49-57. doi: 10.1016/j.tox.2010.07.001.

Tsai WT, Lo YC, Wu MS, Li CY, Kuo YP, Lai YH, Tsai Y, Chen KC, Chuang TH, Yao CH, Lee JC, Hsu LC, Hsu JT, Yu GY (2016) Mycotoxin Patulin Suppresses Innate Immune Responses by Mitochondrial Dysfunction and p62/Sequestosome-1-dependent Mitophagy. J Biol Chem 291(37):19299-19311. doi: 10.1074/jbc.M115.686683.

Wang Y, Peng X, Yang Z, Zhao W, Xu W, Hao J, Wu W, Shen XL, Luo Y, Huang K (2017) iTRAQ Mitoproteome Analysis Reveals Mechanisms of Programmed Cell Death in *Arabidopsis thaliana* Induced by Ochratoxin A. Toxins (Basel) 9(5). pii: E167. doi: 10.3390/toxins9050167.

Wang YM, Liu JB, Peng SQ (2009) Effects of Fusarium mycotoxin butenolide on myocardial mitochondria *in vitro*. Toxicol Mech Meth 19(2):79-85. doi: 10.1080/15376510802322802.

Yekkour A, Tran D, Arbelet-Bonnin D, Briand J, Mathieu F, Lebrihi A, Errakhi R, Sabaou N, Bouteau F (2015) Early events induced by the toxin deoxynivalenol lead to programmed cell death in Nicotiana tabacum cells. Plant Sci 238:148-57. doi: 10.1016/j.plantsci.2015.06.004.

Zhong Y, Jin C, Gan J, Wang X, Shi Z, Xia X, Peng X (2017) Apigenin attenuates patulin-induced apoptosis in HEK293 cells by modulating ROS-mediated mitochondrial dysfunction and caspase signal pathway. Toxicon 137:106-113. doi: 10.1016/j.toxicon.2017.07.018.
