# Supplementary material for: Fake paper identification in the pool of withdrawn and rejected manuscripts submitted to Naunyn–Schmiedeberg’s Archives of Pharmacology
Source: Naunyn Schmiedebergs Arch Pharmacol. 2023 Oct 5;397(4):2171–81. doi: 10.1007/s00210-023-02741-w (PMC10933159; doi:10.1007/s00210-023-02741-w)
Supplement: Supplementary file 7 — Supplementary file7 (PDF 5820 KB) [file 210_2023_2741_MOESM7_ESM.pdf]

Figure S7

Color coding:

Yellow highlighted text	The text is identical in the NSAP version and the published version of this paper.
Red highlighted text	There are differences in the content between the NSAP version and the published version of this paper.
Blue highlighted text	The content is identical in both versions of this paper, but the text has been reworded.
Yellow bordered figure	This Figure is not identical in both versions of this paper.

Naunyn-Schmiedeberg's Archives of Pharmacology

The Signaling of Protease Activated Receptor-2 Activating Peptide-Induced Contraction in Cat Esophageal Smooth Muscle Cells

--Manuscript Draft--

**Manuscript Number:**	NSAP-D-16-00139R1
**Full Title:**	The Signaling of Protease Activated Receptor-2 Activating Peptide-Induced Contraction in Cat Esophageal Smooth Muscle Cells
**Article Type:**	Original Article
**Corresponding Author:**	Uy Dong Sohn, Ph.D.<br>Chung-Ang University, College of Pharmacy<br>Seoul, KOREA, REPUBLIC OF
**Corresponding Author Secondary Information:**	
**Corresponding Author's Institution:**	Chung-Ang University, College of Pharmacy
**Corresponding Author's Secondary Institution:**	
**First Author:**	Hyun Su Ha
**First Author Secondary Information:**	
**Order of Authors:**	Hyun Su Ha
	Young Sil Min
	Phyu Phyu Khin
	Su Jin Kim
	Seung In Um
	Jin Hyung Bae
	Se Eun Lee
	Sang Beom Han
	Ji Hoon Jeong
	In Kyeom Kim
	Uy Dong Sohn, Ph.D.
**Order of Authors Secondary Information:**	
**Funding Information:**	
**Abstract:**	Abstract<br>Purpose;Protease-activated receptors (PARs) are a family of G protein-coupled receptors with a unique activation mechanism involving proteolytic cleavage of the extracellular N-terminal domain of the receptor. PAR1, PAR3 and PAR4 are thrombin receptors, while PAR2 is a receptor for trypsin, tryptase and other proteases. The effects of PAR2 vary depending on the organs and species. In the esophagus, PAR2 has a contractile effect on smooth muscle. Here, we investigate the signaling pathways of PAR2-activating peptide (PAR2-AP) induced contraction in cat esophageal smooth muscle cells.<br>Methods;The length of freshly isolated smooth muscle cells and permeabilized cells from feline esophagus were measured by scanning micrometry. The involvement of molecular mechanism was identified by western blot analysis.<br>Results;The responses to PAR2-AP were initial and sustained contractions, depending on time. The maximum contraction of the initial phase occurred at 60 seconds. The PAR2-AP induced contraction was mediated by Gαi1, Gαi3 and Gαq protein activation, leading to phospholipase-c (PLC) and myosin light chain kinase (MLCK) activation. 20-kDa myosin light chain (MLC20) was phosphorylated by PAR2-AP. Rho kinase-2

Powered by Editorial Manager® and ProduXion Manager® from Aries Systems Corporation

	(ROCK-2), an activator of CPI-17, was increased by PAR2 receptor activation. As a result, CPI-17 was phosphorylated.<br>Conclusions;PAR2-AP produced an initial contraction mediated by Gαi1, Gαi3 and Gαq protein activation, resulting in PLC and MLCK activation. The sustained contraction was mediated by the Rho/Rho kinase pathway.
**Response to Reviewers:**	see attachment

Powered by Editorial Manager® and ProduXion Manager® from Aries Systems Corporation

Dear Associate Editor,

Rennolds S Ostrom, Ph.D.

Naunyn-Schmiedeberg's Archives of Pharmacology

We accepted the reviewer's comments and we revised the manuscripts and gave response to the reviewers' comments in red color point by point. We hope our manuscript can be acceptable. And, we would like to say sorry for our delayed reply because our university summer vacation period.

Reviewers' comments:

Reviewer #1: Synopsis

In this manuscript, the researchers describe experiments to examine the intracellular signal transduction of PAR2 activation in esophageal smooth muscle cells from cats. PAR2 is a GPCR with many effects on cells including modulating directly and indirectly the motor tone of smooth muscle cells. Experiments describe the use of cultured esophageal smooth muscle cells isolated from cats with the PAR2-activing peptide SLIGKV, and the serine protease trypsin, with various combinations of pharmacological inhibitors. SLIGKV is known to activate human and rodent PAR2. A main premise is that the SLIGKV is equivalent to the native (wild-type) cat PAR2 "tethered ligand," The average (mean) length of cells from separate pools of samples after different treatments is used to estimate 'cell contractility.' The authors conclude that SLIGKV contracted esophageal smooth muscle cells through time-dependent mechanisms involving various Galpha/PKC/MLCK and separately by Rho/Rho kinase pathway.

The manuscript presents research an interesting area of pharmacology. The clinical significance of the research is mentioned briefly in the manuscript. However, the experimental model has limitations that will limit the study's usefulness for other researchers. The main assay ('scanning micrometry') employed in the studies is not an accurate approach and appears to be susceptible to high variable baseline for studying smooth muscle contractility. There is uncertainty about the validity of using cultured cells to study the contraction of smooth muscle cells. There is no evidence demonstrating that SLIGKV actually activates PAR2; i.e. the molecular nature of the feline PAR2 protein/gene sequence for the 'tethered ligand' is not necessarily conserved with rodents and humans nor is its selectivity and specificity. The conclusions drawn by the authors overstate the results. While the specific peptide SLIGKV may induce shape changes in the cells examined in this study, the

experiments failed to demonstrate the activity was selective or specific to PAR2. Given the off-target effects of the early-class PAR2-activating peptides in particular, the study did not achieve its claimed objective.

Specific Comments

Abstract

1. The text of the abstract and title does not accurately reflect the methods of the study. This study used primary cultured cells not freshly isolated cells. The study examined the effects of a peptide that activates human and rodent PAR2, but it cannot be presumed to be an activator of feline PAR2. In fact recent a

recent study published the finding that trypsin can activate PAR1 in some cells when PAR2 is absent.

Answer; In this study, we used freshly isolated cells. In this study, we used PAR-2 agonist to see whether cat esophageal smooth muscle give contractile response or not.

Introduction

2. The quality of the writing and presentation requires some attention to correct grammar and readability. For examples, in line 52 (page 1 of intro) starting 'Activation …" the relevant content is reversed. It would be appropriate to cite relevant references in several passages of text. E.g. line 18. Line 56. In the context of PAR pharmacology, it is important that the authors convey that 'agonists' of PARs can be enzymes or synthetic ligands, and the term 'agonist' needs to be qualified when discussing either type of activator. Relevant context should be added to qualify the content in line 11 (page 2 of intro) i.e. to identify the cell type.

Answer; We had some careless mistakes when writing the manuscripts. But we made corrections on page 3, line 22;

“. Activation of the Gq protein triggers the PLCβ pathway, leading to the activation of PARs, which play a central role in producing smooth muscle contractions(Kim et al., 2014).”

Methods

3. There are many details missing that would prevent a knowledgeable researcher from replicating this study. In particular it is unclear whether the research studies were conducted in 'freshly isolated cells' as inferred in the abstract or in first-passage cultured cells as indicated in the (starting line 54 on page 1 of Methods).

Answer; this research study used freshly isolated cells and we made corrections.

4. Please provide the ages of cats, the housing conditions, diet, and suppliers for the animals.

Answer; As usual, most research studies using cats choose the cats depending on their weight, supplied by Han-Lym Lab Animal Co., Ltd. The animals were group-housed in cages in a room controlled for temperature (24-25℃), and were fed a normal laboratory diet (Samtako Bio). Cats were fasted for 24 hrs prior to experiment, but were allowed free access to tap water throughout. We described about this on page 5, line 22.

“Male cats weighing approximately 3kg were used, supplied by Han-Lym Lab Animal Co., Ltd. The animals were group-housed in cages in a room controlled for temperature (24-25℃), and were fed a normal laboratory diet (Samtako Bio). Cats were fasted for 24 hrs prior to experiment, but were allowed free access to tap water throughout.”

5. Please clarify whether independent experiments were conducted. The methods indicate triplicate measurements were made and thus, suggests n=1.

Answer; we did independent experiments by 5 times, suggesting n=5. We explained about this on page 9,

line 13.
"Data represent the mean values from five experiments."

6. SLIGKV is one of the low potency PAR2 activators, and effectively replaced by newer pharmacological tools. Do the authors have evidence of the effects of other newer PAR2 agonists in their model? In the methods, the authors need to be clear when referring to PAR2 activating peptide that they mean SLIGKV.

Answer; we only used SLIGKV, PAR-2 activator. And PAR2 activating peptide refers to SLIGKV. On Page 5, line 13, it was described as "PAR2 activating peptide (PAR2-AP), SLIGKV(Known as PAR2-AP)".

7. There is a large variability in the baseline measurement of cell length. Please clarify whether length refers to the longest measured axis of the cell. Line 30 of page 2 of Methods should indicate number of observations. The Method section indicates that the authors recorded a baseline length based on pooled control group of cells. Please clarify the treatment protocol for 'control group' and the baseline lengths of the separate control groups if any (that is, presence of each inhibitor without PAR2-AP).

Answer; we described about the baseline measurement and explained about the protocol in more details from page 7, line 22 to page 8, line 2.

"Every 50 isolated cells were counted for using as control. The length of isolated cells treated with a contractile agent was measured at random. The average length of treated cell group was then compared with the average length of untreated cells group. Control value of each experiment was 90-100 μm, after unless stated all counting experiments. Contraction was expressed as the percentage decrease in the mean cell length from the control. Percentage decrease was calculated as:

$$\textbf{Cell contraction (\%)} = 100 - \frac{\text{mean cell length of experimental group}}{\text{mean cell length of control group}} \times 100$$

"

8. Please clarify the protocol used for time-course for PAR2-AP induced contractions. The Figures imply repeated measurements on the same cells over time but the methods is unclear whether separate pools of cells were used by stopping the reaction at different time points.

Answer; We explained about the protocol in more details in material and methods part on page 8, line 3.
"The time course of contraction upon the addition of agonists consists of a peak contraction followed by a

lower sustained plateau. Contraction in the present study refers to the initial peak contraction that occurred at 60 seconds upon the addition of agonists (trypsin or PAR2-AP)."

9. Please clarify the type of surface that cells were attached (plastic, glass, coatings) and concentration of collagenase used to detach cells. Please provide details of the contents of the cytosolic buffer.

Answer; We used coatings as the surface for cell attachment. The contents of the cytosolic buffer was described on page 7, line 7.
"20 mM NaCl, 100 mM KCl, 5.0 mM $MgSO_4$; 0.96 mM NaH_2PO_4; 1.0 mM EGTA and 0.48 mM $CaCl_2$ and 2% bovine serum albumin. The cytosolic buffer was equilibrated with 95% O_2-5% CO_2 to maintain pH 7.2 at 31°C."

10. Do the authors have evidence to demonstrate the effects of a positive control contractile agonist in their preparation?
11. Did the authors run molecular weight ladders? It is unclear how the normalization targets were quantified in the Western blots. Were the membranes stripped and re-probed or divided for separate reactions or run independently? Please indicate the amount of protein loaded in each lane.

Answer; We described the molecular weight of the proteins in figure and described how we did protein assay on page 8, line 13.
"The protein concentration of the supernatant in each reaction vial was measured spectophotometrically using the Bio-Rad assay. Absorption was monitored at 590 nm. The amount of protein loaded was 30 μm."

12. Student's t-test is appropriate for only two groups. Several figures indicate comparisons were made between groups containing more than two.

Answer; For comparison of two groups, we used Student's t-test. For, comparisons for groups containing more than two, we used one way ANOVA test.

Results
13. Representative images of the freshly dispersed smooth muscle cells before and after tissue culture would improve the quality of the manuscript.

Answer; we provided the representative images of the freshly isolated smooth muscle cells before and after tissue culture in figure section.

14. Representative images should include molecular weight ladders and or lanes containing positive control samples.

Answer; we added the molecular weight of the samples in figure.

15. Figure 8 is unnecessary and could be omitted.

Answer; we omitted the unnecessary figure 8.

16. Are the 'contractions' of the ESMC completely reversible with time?

Answer; the contraction of the ESMC are reversible with time.

Discussion

17. It would be appropriate to discuss the limitations of the experimental approach with respect to interpretation of data and broad applicability for other researchers, especially those that study PARs, and smooth muscle.

Answer; we discussed about this in more detail in discussion part on page 16, line 2.

"PAR-2 is an important target of drug in inflammation. But immoderate suppression of PAR2 induces esophageal smooth muscle cell relaxation and then promotes gastroesophageal reflux. Relaxation of esophageal smooth muscle is one of gastroesophageal reflux reason. This study was conducted in protein level. Thus, further more studies are necessary at gene or animal level."

18. It would be appropriate to clarify the potential clinical significance of the findings in the context of GERD.

Answer; yes, we discussed about this in discussion part on page 16, line 2.

"PAR-2 is an important target of drug in inflammation. But immoderate suppression of PAR2 induces esophageal smooth muscle cell relaxation and then promotes gastroesophageal reflux. Relaxation of esophageal smooth muscle is one of gastroesophageal reflux reason. This study was conducted in protein level. Thus, further more studies are necessary at gene or animal level."

Manuscript
Click here to download Manuscript Naunyn 0709.doc
Click here to view linked References

The Signaling of Protease Activated Receptor-2 Activating Peptide-Induced Contraction in Cat Esophageal Smooth Muscle Cells

Authors and Affiliations:

Hyun Su Ha[1#], Young Sil Min[2#], Phyu Phyu Khin[1], Su Jin Kim[1], Seung In Um[1], Jin Hyung Bae[1], Se Eun Lee[1], Sang Beom Han[1], Ji Hoon Jeong[3], Inkyeom Kim[4] and Uy Dong Sohn[1]*

[1]College of Pharmacy, Chung-Ang University, Seoul 156 -756, Republic of Korea. [2]Department of Medical Plant Science, Jung Won University, Chungbuk, Republic of Korea. [3]Department of Pharmacology, College of Medicine, Chung-Ang University, Seoul 156-756, Republic of Korea. [4]Department of pharmacology, School of Medicine, Kyungpook National University, Daegu 700-842, Republic of Korea

Running Title: PAR-2 Contraction in Smooth Muscle Cells

[#] **;** equally contributed.

Acknowledgements

This research was supported by the Basic Science Research Program through the National Research Foundation of Korea (NRF), funded by the Ministry of Education, Science and Technology [Grant 2011-0012139].

Corresponding author:
Uy Dong Sohn
Department of Pharmacology, College of Pharmacy, Chung-Ang University, Seoul 156-756, Republic of Korea
Tel: +82-2-820-5614, Fax: +82-2-826-8752
E-mail: udsohn@cau.ac.kr

Abstract

Purpose; Protease-activated receptors (PARs) are a family of G protein-coupled receptors with a unique activation mechanism involving proteolytic cleavage of the extracellular N-terminal domain of the receptor. PAR1, PAR3 and PAR4 are thrombin receptors, while PAR2 is a receptor for trypsin, tryptase and other proteases. The effects of PAR2 vary depending on the organs and species. In the esophagus, PAR2 has a contractile effect on smooth muscle. Here, we investigate the signaling pathways of PAR2-activating peptide (PAR2-AP) induced contraction in cat esophageal smooth muscle cells.

Methods; The length of freshly isolated smooth muscle cells and permeabilized cells from feline esophagus were measured by scanning micrometry. The involvement of molecular mechanism was identified by western blot analysis.

Results; The responses to PAR2-AP were initial and sustained contractions, depending on time. The maximum contraction of the initial phase occurred at 60 seconds. The PAR2-AP induced contraction was mediated by $G_{\alpha i1}$, $G_{\alpha i3}$ and $G_{\alpha q}$ protein activation, leading to phospholipase-c (PLC) and myosin light chain kinase (MLCK) activation. 20-kDa myosin light chain (MLC_{20}) was phosphorylated by PAR2-AP. Rho kinase-2 (ROCK-2), an activator of CPI-17, was increased by PAR2 receptor activation. As a result, CPI-17 was phosphorylated.

Conclusions; PAR2-AP produced an initial contraction mediated by $G_{\alpha i1}$, $G_{\alpha i3}$ and $G_{\alpha q}$ protein activation, resulting in PLC and MLCK activation. The sustained contraction was mediated by the Rho/Rho kinase pathway.

Keywords; PAR2-AP, cat esophageal smooth muscle cells, contraction, G proteins

Introduction

Protease-activated receptors (PARs) are a family of G protein-coupled receptors with a unique activating mechanism involving proteolytic cleavage of the extracellular N-terminal domain of the receptor(Kawabata, 2002). While PAR1, PAR3 and PAR4 are activated by thrombin, PAR2 is not. PAR2 is considered to be a receptor for trypsin and tryptase (Nystedt et al., 1994, Hollenberg et al., 1997, Molino et al., 1997, Kawabata, 2002), and many endogenous and exogenous proteinases are now known to activate PAR2. In the gastrointestinal (GI) tract, PAR1 and PAR2 are the major PAR subtypes and are considered to be key molecules in the regulation of GI function and in the pathogenesis of GI diseases.

One of the functions of PAR2 in the GI tract is the regulation of exocrine secretion. PAR2 agonists evoke salivation *in vivo* and secretion of amylase and mucin in rat parotid (Kawabata et al., 2000). Another role of PARs is the modulation of smooth muscle motility (Sekiguchi et al., 2006). Both PAR1 and PAR2 activating peptides induce contraction in mouse gastric longitudinal smooth muscle strips. When pretreated with carbachol, the peptides induced transient relaxation in the same muscle strip (Cocks et al., 1999, Sekiguchi et al., 2006). Agonists of PAR2 evoke transient relaxation followed by contraction. It has been confirmed that responses to PAR2 agonists completely disappear in PAR2-deficient mice (Sekiguchi et al., 2006). In rat duodenam, PAR1 agonists produce relaxation followed by choline-induced contraction, while PAR2 agonists induce persistent contraction (Kawabata et al., 1999). In guinea pig lower esophageal sphincter, PAR1 and PAR2 mediated relaxation, but PAR4 did not (Huang, 2007). In human esophageal mucosae, PAR1 and PAR2 agonists produce contraction (Chang et al., 2010).

The mechanism for the modulation of GI motility by PARs is also highly complex and involves multiple pathways. Activation of the Gq protein triggers the PLCβ pathway, leading to the activation of PARs, which play a central role in producing smooth muscle contractions(Kim et al., 2014).

PAR2 has been demonstrated to induce proinflammatory and neuroinflammatory effects. PAR2 gene expression is upregulated in the mucosa of patients with gastroesophageal reflux disease (GERD)

(Kandulski et al., 2010). Inflammatory cells such as mast cells are abundant sources of proteases that activate PARs. Mast cells play an important role in the recruitment of neutrophiles in GERD (Morganstern et al., 2008). Thus it is possible that inflammatory cell degranulation results in protease release, which in turn induces the contraction of esophageal smooth muscle by PARs.

Recent studies have shown that the contractile response to PAR2-activating peptide consists of two phases (Sriwai et al., 2013). An initial phase is mediated by the formation of IP_3 and Ca^{2+}/calmodulin-dependent activation of myosin light chain kinase (MLCK), leading to the phosphorylation of MLC_{20}. A sustained phase is mediated by a Ca^{2+}-independent mechanism and inhibition of MLC phosphatase (Murthy and Makhlouf, 1996). Inhibition of MLC phosphatase consists of two pathways. The first is the phosphorylation of myosin phosphatase (MYPT1), the regulatory subunit of MLC phosphatase, and the second is the PKC-dependent phosphorylation of CPI-17, an endogenous inhibitor of MLC phosphatase.

The aim of this study is to identify the signaling pathway to which these receptors are coupled in freshly isolated smooth muscle cells of cat esophagus. Trypsin and the synthetic peptide SLIGKV were used to activate PAR2s and identify the downstream signaling pathways. Selective G-protein antibodies were used to identify the coupling of specific G-proteins to effector enzymes (Kwon et al., 2015).

Materials and Methods

Reagents

G protein antibodies ($G_{\alpha i1}$, $G_{\alpha i2}$, $G_{\alpha i3}$, $G_{\alpha q}$, $G_{\alpha s}$, $G_{\alpha o}$ and G_{β}) and PLC isozymes antibodies (β1, β3 and γ1) were purchased from Santa Cruz Biotechnology (Santa Cruz, CA, USA); Chelerythrine chloride from Research Biochemicals (Natick, MA, USA); goat anti-rabbit IgG-HRP from Bethyl Laboratories Inc. (Eccles Avenue, CA, USA); rainbow molecular weight marker from Amersham (Arlington Heights, IL, USA); enhanced chemiluminescence (ECL) agent from PerkinElmer Life Sciences (Boston, MA, USA); sodium dodecyl sulfate (SDS) sample buffer from Owl scientific Inc. (Woburn, MA, USA); nitrocellulose membrane, Tris/Glycine/SDS buffer and Tris/Glycine buffer from BioRad (Richmond, CA, USA); phosphate-buffered saline (PBS) from Roche Diagnostics Co. (Indianapolis, IN, USA); Restore^{TM} Western blot stripping buffer from Pierce (Rockford, IL, USA); and 4-(2-hydroxyethyl)-1-piperazine-N'-2-ethane sulfonic acid (HEPES), trypsin, PAR2 activating peptide (PAR2-AP), SLIGKV(Known as PAR2-AP), collagenase type F, ammonium persulfate, ponceau S, bovine serum albumin (BSA), leupeptin, aprotinin, β-mercaptoethanol, N,N,N',N'–tetramethylethylene diamine (TEMED), ethylene glycol-bis-(β-aminoethylether)- N,N,N',N'-tetraacetic acid (EGTA), ethylenediamine tetraacetic acid (EDTA), phenylmethyl-sulfonylfluoride (PMSF) and other reagents from Sigma Chemical Co. (St. Louis, MO, USA).

Preparation of dispersed muscle cells

Esophageal smooth muscle cells were isolated as previously described (Biancani et al., 1987, Nam et al., 2013). Male cats weighing approximately 3kg were used, supplied by Han-Lym Lab Animal Co., Ltd. The animals were group-housed in cages in a room controlled for temperature (24-25℃), and were fed a normal laboratory diet (Samtako Bio). Cats were fasted for 24 hrs prior to experiment, but were allowed free access to tap water throughout. Muscle strips were incubated overnight in normal potassium-HEPES buffer containing 1 mg/ml papain, 1 mM dithiothreitol, 1 mg/ml BSA and 0.5 mg/ml

collagenase (type F, Sigma) and equilibrated with 95% O_2-5% CO_2 to maintain pH 7.0 at 31°C. The composition of the normal potassium-HEPES buffer was 1 mM $CaCl_2$, 250 µM EDTA, 10 mM glucose, 10 mM HEPES, 4 mM KCl, 131 mM NaCl, 1 mM $MgCl_2$ and 10 mM taurine. Next day we warmed up the tissue at room temperature for 30 min and incubated the tissue in a water bath at 31°C for 30 min. After incubation, the digested tissue was poured out over a 360-µm Nitex filter, rinsed in collagenase-free HEPES buffer to remove any trace of collagenase, and then incubated in this solution at 31°C, gassed with 95% O_2-5% CO_2. The cells were allowed to dissociate freely for 10 to 20 min. Suspensions of single muscle cells were harvested by filtration through 500-µm Nitex mesh. Before beginning the experiment, the cells were kept at 31°C for at least 10 min to relax the cells. Throughout the entire procedure, care was taken not to agitate the fluid in order to avoid cell contraction in response to mechanical stress.

The isolated smooth muscle cells from the esophagus were resuspended in DMEM, containing penicillin (100units/ml), streptomycin (0.1 mg/ml), amphotericin-b (0.25µg/ml) and 10% fetal bovine serum (DMEM-10). The muscle cells were plated at a concentration of 10^4cells/ml and incubated at 37°C in a CO_2 incubator. DMEM-10 medium was replaced every three days for 2-3 weeks until confluence was attained. All experiments were carried out on cells in the isolated cells (Murthy et al., 2003). Animal experiments were approved by the Institutional Animal Care and Use Committee of Chung-Ang University, in accordance with the guide for the Care and Use of Laboratory Animals in Seoul, South Korea.

Identification of dispersed esophageal smooth muscle cells

To identify the dispersion of esophageal smooth muscle in collagenase buffer, the dissected smooth muscle strips were observed by microscopy every 5 minutes, following the overnight treatment. It was observed that esophageal smooth muscle cells were separated from the esophageal smooth muscle strips. Following identification of the separated esophageal smooth muscle cells, the incubation was stopped to

prepare the dispersed muscle cell suspension. Freshly isolated esophageal smooth muscle cells were presented as spindle-shaped, with a diverse length range of 55 – 135μm. Each 50 isolated cells were counted for the use of average value for each experiment (control baseline value 93± 5μm)

Preparation of permeabilized smooth muscle cells

Cells were permeabilized to diffuse agents such as G-protein and PLC isozyme antibodies that do not diffuse across the intact cell membrane. The preparation process of permeabilized cells does not affect the contraction of the cells (Horowitz et al., 1996, Sohn et al., 1997, Cao et al., 2001, Shim et al., 2002, Murthy et al., 2003). After completion of the enzymatic phase of the digestion process, the partly digested muscle tissue was washed with an enzyme-free cytosolic buffer of the following composition: 20 mM NaCl, 100 mM KCl, 5.0 mM $MgSO_4$; 0.96 mM NaH_2PO_4; 1.0 mM EGTA and 0.48 mM $CaCl_2$ and 2% bovine serum albumin. The cytosolic buffer was equilibrated with 95% O_2-5% CO_2 to maintain pH 7.2 at 31°C. Muscle cells dispersed spontaneously in this medium. The cytosolic buffer contained 0.48 mM $CaCl_2$ and 1 mM EGTA, yielding 0.18 mM free Ca^{2+}. After exposure to saponin, the cell suspension was spun at 350 g, and the resulting pellet was washed with saponin-free modified cytosolic buffer that contained antimycin A (10 μM), ATP (1.5 mM) and an ATP-regenerating system that consisted of creatine phosphate (5 mM) and creatine phosphokinase (10 units/ml). After the cells were washed free of saponin, they were resuspended in modified cytosolic buffer.

Measurement of contraction by scanning micrometry

The contraction of isolated muscle cells was measured by scanning micrometry (Sohn et al., 1995). An aliquot of cell suspension containing 10^4muscle cells/ml was added to HEPES medium containing the test agents. The reaction was terminated by the addition of acrolein(1% final concentration). Every 50 isolated cells were counted for using as control. The length of isolated cells treated with a contractile agent was measured at random. The average length of treated cell group was then compared with the average length of untreated cells group. Control value of each experiment was 90-100 μm, after unless stated all counting experiments. Contraction was expressed as the percentage decrease in the mean cell

length from the control. Percentage decrease was calculated as:

$$\text{Cell contraction (\%)} = 100 - \frac{\text{mean cell length of experimental group}}{\text{mean cell length of control group}} \times 100$$

The time course of contraction upon the addition of agonists consists of a peak contraction followed by a lower sustained plateau. Contraction in the present study refers to the initial peak contraction that occurred at 60 seconds upon the addition of agonists (trypsin or PAR2-AP).

Western blotting analysis

Phosphorylated MLC_{20} was determined by immunoblotting analysis using a phospho-specific antibody(Murthy et al., 2003, Huang et al., 2005). Previously frozen samples of dispersed muscle cells were homogenized in buffer containing 20mM Tris-HCl (pH 7.4), 0.5mM EDTA, 0.5mM EGTA, 1% (w/v) Triton X-100, 0.01% (w/v) SDS, 10μg/ml leupeptin, 10μg/ml aprotinin, 1mM PMSF, 10μl/ml phosphatase inhibitor cocktail-3, and 0.7μg/ml β-mercaptoethanol. Samples of the homogenates were then centrifuged for 10 minutes at 4°C, and the supernatants collected. The protein concentration of the supernatant in each reaction vial was measured spectrophotometrically using the Bio-Rad assay (Bio-Rad, Richmond, CA). Absorption was monitored at 590 nm. Aliquots were subjected to electrophoresis on an SDS-polyacrylamide gel. A prestained molecular mass marker, to permit molecular mass determination, was also run in an adjacent lane in loading buffer (25mM Tris (pH 8.3), 192mM glycine, and 0.1% SDS), using a power supply (Power Pac 1000, Bio-Rad, Melville, NY, USA). The separated proteins were transferred to a 0.45-μm nitrocellulose membrane in transfer buffer (25mM Tris (pH 8.3), 192mM glycine, and 20% (v/v) methanol), using a power supply (Power Pac 1000, Bio-Rad, Melville, NY, USA). To confirm uniformity of gel loading, the blot was stained with Ponceau S. After confirmation, the membrane was washed with TBS, and then incubated in TBS buffer containing 3% BSA and 0.05% Tween20 (TBST) for 3hours at room temperature to block nonspecific binding. After washing three times with TBS for 15 minutes, the membrane was incubated with an antibodiy (1:1000

dilution) agonist MLC_{20} (Ser^{19}/Thr^{18}) in a TBST solution containing 3% BSA at 4°C overnight. The membrane was washed twice for 5 minutes with TBST and incubated with a horseradish peroxidase-conjugated secondary antibody (1:5000 dilution) for 1 hour at room temperature. The immunoreactive bands, detected by enhanced chemiluminescent agent s (ECL; Perkin Elmer, Walthem, MA, USA), were developed using X-ray film, and scanned and analyzed densitometrically using the Scion Image software. After detecting phosphoryalted MLC_{20}, the membrane stripped and re-proved for total MLC_{20}. Phosphorylation of MLC_{20}, CPI-17 and the increase in ROCK were calculated as the ratios of phosphorylated MLC_{20} to total MLC_{20}, phosphorylated CPI-17 to GAPDH and ROCK to GAPDH. These antibodies have shown previous success in feline experiments (Cao et al., 2001, Ijzer et al., 2009, Nam et al., 2013).

Analysis of data

Data represent the mean values from five experiments. The data are expressed as the mean ± S.E.M. and analyzed with a Student's *t*-test. A $P < 0.05$ was considered statistically significant.

Results

Effects of trypsin on esophageal smooth muscle cells

The esophageal cells under microscopy were isolated with digestion process (Fig. 1A). Freshly isolated smooth muscle cells were treated for 60 seconds with 10^{-8} to 10^{-5} M trypsin, which induced smooth muscle cell contraction in a concentration-dependent manner (Fig. 1B). The maximal contraction occurred at 10^{-5} M and the maximal response occurred at 60 seconds. The trypsin-induced contraction was maintained for 5 minutes (Fig. 1C) shows the effects of trypsin over time. The initial contraction and sustained contraction induced by trypsin were inhibited by the PAR2 antagonist FSLLRY-NH2 (Fig. 1D & E). Trypsin has been shown to trigger PAR2-activation (Vergnolle et al., 1998).

PAR2-AP induces contraction of isolated esophageal smooth muscle cells

The effects of PAR2-AP are different depending on the organ and species. In the present study, freshly isolated feline esophageal smooth muscle cells were stimulated for 60 seconds, with 10^{-10} to 10^{-5} M PAR2-AP. The response to PAR2-AP was concentration-dependent, and the maximal response was observed at 10^{-5} M (Fig. 2A). Fig. 2B presents the time-course of PAR2-AP induced contraction. The maximal contraction was at 60 seconds and the response was sustained for up to 5 minutes. The contractile effects of PAR2-AP were similar to trypsin-induced contraction. In the concentration-response and time-course data, PAR2s in esophageal smooth muscle cells was activated by PAR2-AP at a final concentration of 10^{-6} M for 60 seconds in most of the later experiments (Fig. 2B).

Characterization of G-protein subtypes involved in PAR2-AP induced contraction

PAR receptors have been shown to be coupled to a PTX-sensitive G-protein. In this study, we pre-incubated dispersed muscle cells for 60 minutes with PTX (400ng/ml), which reduced the PAR2-AP-induced initial contraction by 54.77±6.9%, indicating that the initial contraction is partially coupled to a

PTX-sensitive G-protein (Fig. 3A). We have previously shown that $G_{\alpha i1}$, $G_{\alpha i2}$, $G_{\alpha i3}$, G_{β} (40kDa), $G_{\alpha o}$ (40kDa), $G_{\alpha q}$ (42kDa) and $G_{\alpha s}$ (46kDa) proteins exist in feline esophageal smooth muscle cells. To identify the specific G protein involved in the PAR2-AP induced contraction, the cells were permeabilized with saponin in cytosolic medium-, containing each G protein antibody (1:200) dilution, to allow diffusion of the antibodies into the cells. These antibodies inhibited receptor-induced activation of G proteins by binding to their terminal peptide regions that interact with the receptor. Antibodies against $G_{\alpha i1}$, $G_{\alpha i3}$ and $G_{\alpha q}$ inhibited the PAR2-AP-induced initial contraction, while antibodies to $G_{\alpha 0}$, $G_{\alpha i2}$, $G_{\alpha s}$ and G_{β} did not. Our data suggest that the PAR2-AP-induced initial contraction is mediated by $G_{\alpha i1}$, $G_{\alpha i3}$ and $G_{\alpha q}$ proteins (Fig. 3B). PTX reduced the PAR2-AP-induced sustained contraction by 66.32±10.3%, indicating that the sustained contraction is partially coupled to a PTX-sensitive G-protein (Fig. 3C). Antibodies against $G_{\alpha i1}$ and $G_{\alpha i3}$ inhibited the PAR2-AP-induced sustained contraction, while antibodies against $G_{\alpha 0}$, $G_{\alpha i2}$, $G_{\alpha s,}$ $G_{\alpha q}$ and G_{β} did not (Fig. 3D).

Pathways mediating the PAR2-AP induced initial contraction

The initial (60 second) contraction induced by PAR2-AP in smooth muscle cells was decreased by the PLC inhibitor U-73122 (31.8 ± 6.3% inhibition) and the MLCK inhibitor ML-9 (44.1 ± 6.3% inhibition). However, it was not inhibited by the Rho kinase inhibitor Y27632 or the PKC inhibitor chelerythrine (Fig. 4A). These results suggest that the PAR2-AP induced initial contraction is mediated by PLC and MLCK in dispersed esophageal smooth muscle cells (ESMC).

Pathways mediating the PAR2-AP-induced sustained contraction

The sustained contraction in dispersed smooth muscle cells was measured 4 minutes after treatment with PAR2-AP. The response to PAR2-AP was partly inhibited by Y27632 (47.2 ± 14.5% inhibition), FIPI (53.5 ± 25.1% inhibition) and chelerythrine(41.1 ± 17.3% inhibition), but not by U73122 or ML-9(Fig. 4B). These results indicate that PAR2-AP induced sustained contraction mediated by Rho kinase,

PLD and PKC in dispersed ESMC.

PLCβ1 mediates PAR2-AP induced contraction

In our previous study, we demonstrated the presence of immunoreactive bands at 150kDa using antibodies against PLCβ1 and PLCβ3, and at 145kDa using an antibody against PLCγ1, in dispersed ESMC. To determine which PLC isozyme mediated the PAR2-AP induced contraction, we carried out incubation of permeabilized smooth muscle cells for 1hour with specific antibodies against PLC β1, PLCβ3, and PLCγ1 (1:200). Only PLCβ1 inhibited the PAR2-AP-induced contraction significantly. No other PLC-specific antibody had a significant effect on contraction (Fig. 5A). These results suggest that the PAR2-AP induced initial contraction is mediated by PLCβ1. In the sustained phase, No PLC-specific antibody had an effect (Fig. 5B).

PAR2-AP induced an increase in Rho kinase-2

Rho kinase-1(ROCK-1) and Rho kinase-2(ROCK-2) detection experiments were performed to determine whether PAR2-AP induced upregulation of ROCK-1 or ROCK-2. ROCK-2 was increased by PAR2-AP (Fig. 6), while ROCK-1 was not increased by PAR2 activation (data not shown).

PAR2-AP induced phosphorylation of MLC_{20} and CPI-17

Smooth muscle contraction and relaxation regulate by MLC_{20} phosphorylation(Regulation of Smooth Muscle Contraction by Small GTPases.

An MLC_{20} phosphorylation experiment was performed to determine whether PAR2-AP induced the phosphorylation of MLC_{20} in ESMC. MLC_{20} phosphorylation by PAR2-AP, as measured using a phospho-specific antibody (MLC_{20} Ser19/ Thr 18), was rapid (within 60 seconds) and sustained (4 minutes) (Fig. 7A). MLC phosphatase was inhibited by the phosphorylation of CPI-17, an inhibitor of its catalystic subunit (Somlyo and Somlyo, 2000), It is known that thrombin is involved in the

phosphorylation of CPI-17 at Thr38 in vascular smooth muscle cells. A CPI-17 phosphorylation experiment was performed to determine whether PAR2 activation produced phosphorylation of CPI-17. CPI-17 phosphorylation by PAR2-AP, as measured using a phospho-specific antibody (Thr 38), was rapid (within 60 seconds) and sustained (4 minutes) (Fig. 7B).

Discussion

In the present study, the effects of PAR2 activation on the contraction of feline esophageal smooth muscle cells were investigated. We tested whether PAR2 activation could induce contraction in feline esophageal smooth muscle cells. The contraction showed concentration dependency that reached maximal contraction at 10^{-5} M. The contractile response to PAR2 activation was evoked initially (in 60 seconds) and sustained to 5 minutes. One of the objectives of the present study was to investigate how the signaling time differed between the initial and sustained contraction.

G-proteins transduce the effect of ligand-binding to cell surface receptor into intracellular signals. The expression of PAR2 in smooth muscle cells of the GI tract is consistent with their expression in other cell types of the GI tract, such as enteric neurons(Gao et al., 2002), myenteric glia(Garrido et al., 2002), epithelial cells of intestine(Kunzelmann et al., 2002, Cottrell et al., 2004), endothelial cells, and vascular smooth muscle cells of the gut(D'Andrea et al., 1998, Kawabata, 2003). Pertussis toxin (PTX) is a well-known inhibitor of the G_i protein. In the present study, PTX inhibited the PAR2-AP-induced initial and sustained contractions. Studies in various cell lines suggest that PAR2 is coupled to both PTX-sensitive and PTX-insensitive G proteins. However, which G protein isoform mediates the PAR2 effect has not been identified. In a previous study, it has been shown that $G_{\alpha i1}$, $G_{\alpha i2}$, $G_{\alpha i3}$, G_{β} (40kDa), $G_{\alpha o}$ (40kDa), $G_{\alpha q}$ (42kDa), and $G_{\alpha s}$ (46kDa) proteins exist in feline esophageal smooth muscle cells. In the present study, isolated smooth muscle cells were permeabilized with saponin to allow the penetration of G protein antibodies into the cytoplasm. Following binding of specific antibodies to their C-terminal tails, G proteins are not activated by signals from receptor-binding, therefore do not transduce the subsequent

signals to their effectors. These data suggest that activation of the PAR2 induced initial contraction was mediated by PTX-sensitive $G_{\alpha i1}$ and- $G_{\alpha i3,}$ and also by the PTX–insensitive G_q protein. Activation of $G_{\alpha i1}$ and $G_{\alpha i3}$ inhibits adenylyl cyclase_(AC)(Billington and Penn, 2003), whereas activation of G_q leads to the activation of PLC β(Malbon, 2005). In addition, the sustained contraction induced by PAR2-AP was mediated by PTX-sensitive $G_{\alpha i1}$ and $G_{\alpha i3,}$ which activated PLD and Rho A in sequence. During the initial contraction, $G_{\alpha i1}$ and $G_{\alpha i3}$ inhibit the production of cAMP, which reduces MLCK activation(Puetz et al., 2009), whereas, $G_{\alpha i1}$ and $G_{\alpha i3}$ inhibit MLC phosphatase through the Rho A pathway during the sustained contraction.

Activation of PAR2 receptors in esophageal smooth muscle cells induced a concentration-dependent muscle contraction, consisting of an initial phase and a sustained phase. The initial contraction induced by PAR2-AP was inhibited by the PLC inhibitor, U73122 and the MLCK inhibitor, ML-9. It was, however, not affected by the Rho kinase or PKC inhibitors, indicating that the initial contraction induced MLCK-dependent MLC_{20} phosphorylation, resulting in cell shortening.

Consistent with a previous study (Biancani et al., 1987), the sustained contraction was Ca^{2+} independent and involved the activation of Rho A, which resulted in the inhibition of MLC phosphatase and the stimulation of MLC_{20} phosphorylation. The signals involved in the dual activation of Rho kinase and PKC led to the inhibition of MLC phosphatase. Rho kinase and PKC phosphorylated CPI-17, an endogenous suppressive protein of MLC phosphatase. Similarly, the sustained contraction induced by PAR2-AP was inhibited by the Rho kinase inhibitor, Y27632, the PLD inhibitor, FIPI, and the PKC inhibitor, chelerythrine. This data indicates that the sustained contraction was mediated by the activation of Rho kinase, phospholipase D, and PKC.

The present study provides experimental evidence that PAR2-AP induced the initial contraction, mediated by MLCK-dependent MLC_{20} phosphorylation. Smooth muscle cells treated with PAR2-AP for 240 seconds (4 minutes) displayed significantly increased immunoreactive protein bands, compared with untreated cells. These bands corresponded to the phosphospecific Ser 19/Thr 18 MLC_{20}. This data

suggests that PAR2 receptor activation induced MLC_{20} phosphorylation. The sustained contraction was mediated by PKC, PLD, and Rho kinase. Rho kinase (ROCK) is a downstream effector enzyme of Rho GTPase, which increases ROCK activity when bound. ROCK is a serine/threonine protein kinase that has been identified as a GTP-Rho binding protein. PLD, PKC, and Rho kinase caused activation of CPI-17, an endogenous MLC phosphatase inhibitor by phosphorylation(Puetz et al., 2009). Rho kinase has two isoforms, ROCK-1 and ROCK-2(Nakagawa et al., 1996, Fukata et al., 2001). ROCK-1 is mainly expressed in the lung, liver, spleen and kidney, while ROCK-2 is distributed mostly in the brain and heart. Various studies have suggested that the RhoA/ROCK pathway mediates tonic contraction of several smooth muscles, including the esophageal sphincter (Harnett et al., 2005, Buyukafsar et al., 2006). PAR2-AP increased phosphoform of CPI-17 and ROCK-2 indicating that PAR2-AP induced an increase in ROCK-2 production and CPI-17 phosphorylation.

In the GI tract, activated phospholipases (PLC) induce contraction by producing second messengers through the degradation phospholipids. PLC plays an important role in contraction initiated by G protein coupled receptor binding (e.g. muscarinic receptors). PAR2-AP has been shown to stimulate PI hydrolysis in a concentration-dependent manner in colon smooth muscle cells(Sriwai et al., 2013). However, which isoform produced the formation of IP_3 was not demonstrated. A previous study demonstrated the presence of immunoreactive protein bands corresponding to the 150kDa PLC-β1 and PLC-β3 antibodies and the 145kDa PLC-γ1 antibody (Yang et al., 2000). Only PLC-β1 reduced the PAR2-AP induced contraction. These data emphasized the role of PLC-β1 in mediating the esophageal smooth muscle contraction-induced by PAR2 receptor activation.

In conclusion, we report that the esophageal smooth muscle cell contraction by PAR-2 receptor activation consists of two phases, an initial phase and a sustained phase. The initial contraction was mediated by G protein activation. $G_{\alpha q}$ proteins activated PLCβ1 and resulted in formation of IP3. $G_{\alpha i1}$ and $G_{\alpha i3}$ proteins reduced cAMP by inhibition of Adenyl cyclase. Consequently, MLCK phosphorylated MLC20.

On the other hand, Rho kinase, PLD, and PKC were activated by $G_{\alpha i1}$ and $G_{\alpha i3}$. This activation induced CPI-17 phosphorylation, resulting in the inhibition of MLC phosphatase during the sustained contraction. These results suggest that PAR-2 receptor-mediated smooth muscle cell contraction is a novel target for the treatment of GERD. PAR-2 is an important target of drug in inflammation. But immoderate suppression of PAR2 induces esophageal smooth muscle cell relaxation and then promotes gastroesophageal reflux. Relaxation of esophageal smooth muscle is one of gastroesophageal reflux reason. This study was conducted in protein level. Thus, further more studies are necessary at gene or animal level.

REFERENCES

Biancani P, Hillemeier C, Bitar KN, Makhlouf GM (1987) Contraction mediated by Ca2+ influx in esophageal muscle and by Ca2+ release in the LES. The American journal of physiology 253: G760-766

Billington CK, Penn RB (2003) Signaling and regulation of G protein-coupled receptors in airway smooth muscle. Respiratory research 4: 2

Buyukafsar K, Akca T, Nalan Tiftik R, Sahan-Firat S, Aydin S (2006) Contribution of Rho-kinase in human gallbladder contractions. European journal of pharmacology 540: 162-167

Cao W, Chen Q, Sohn UD, Kim N, Kirber MT, Harnett KM, Behar J, Biancani P (2001) Ca2+-induced contraction of cat esophageal circular smooth muscle cells. American journal of physiology Cell physiology 280: C980-992

Chang BS, Chang JC, Huang SC (2010) Proteinase-activated receptors 1 and 2 mediate contraction of human oesophageal muscularis mucosae. Neurogastroenterol Motil 22: 93-97, e32

Cocks TM, Sozzi V, Moffatt JD, Selemidis S (1999) Protease-activated receptors mediate apamin-sensitive relaxation of mouse and guinea pig gastrointestinal smooth muscle. Gastroenterology 116: 586-592

Cottrell GS, Amadesi S, Grady EF, Bunnett NW (2004) Trypsin IV, a novel agonist of protease-activated receptors 2 and 4. J Biol Chem 279: 13532-13539

D'Andrea MR, Derian CK, Leturcq D, Baker SM, Brunmark A, Ling P, Darrow AL, Santulli RJ, Brass LF, Andrade-Gordon P (1998) Characterization of protease-activated receptor-2 immunoreactivity in normal human tissues. J Histochem Cytochem 46: 157-164

Fukata Y, Amano M, Kaibuchi K (2001) Rho-Rho-kinase pathway in smooth muscle contraction and cytoskeletal reorganization of non-muscle cells. Trends in pharmacological sciences 22: 32-39

Gao C, Liu S, Hu HZ, Gao N, Kim GY, Xia Y, Wood JD (2002) Serine proteases excite myenteric neurons through protease-activated receptors in guinea pig small intestine. Gastroenterology 123: 1554-1564

Garrido R, Segura B, Zhang W, Mulholland M (2002) Presence of functionally active protease-activated receptors 1 and 2 in myenteric glia. J Neurochem 83: 556-564

Harnett KM, Cao W, Biancani P (2005) Signal-transduction pathways that regulate smooth muscle function I. Signal transduction in phasic (esophageal) and tonic (gastroesophageal sphincter) smooth muscles. American journal of physiology Gastrointestinal and liver physiology 288: G407-416

Hollenberg MD, Saifeddine M, al-Ani B, Kawabata A (1997) Proteinase-activated receptors: structural requirements for activity, receptor cross-reactivity, and receptor selectivity of receptor-activating peptides. Can J Physiol Pharmacol 75: 832-841

Horowitz A, Clement-Chomienne O, Walsh MP, Morgan KG (1996) Epsilon-isoenzyme of protein kinase C induces a Ca(2+)-independent contraction in vascular smooth muscle. The American journal of physiology 271: C589-594

Huang J, Zhou H, Mahavadi S, Sriwai W, Lyall V, Murthy KS (2005) Signaling pathways mediating gastrointestinal smooth muscle contraction and MLC20 phosphorylation by motilin receptors. American journal of physiology Gastrointestinal and liver physiology 288: G23-31

Huang SC (2007) Protease-activated receptor-1 (PAR1) and PAR2 but not PAR4 mediate relaxations in lower esophageal sphincter. Regul Pept 142: 37-43

Ijzer J, Kisjes JR, Penning LC, Rothuizen J, van den Ingh TS (2009) The progenitor cell compartment in the feline liver: an (immuno)histochemical investigation. Veterinary pathology 46: 614-621

Kandulski A, Wex T, Monkemuller K, Kuester D, Fry LC, Roessner A, Malfertheiner P (2010)

Proteinase-activated receptor-2 in the pathogenesis of gastroesophageal reflux disease. Am J Gastroenterol 105: 1934-1943

Kawabata A (2002) PAR-2: structure, function and relevance to human diseases of the gastric mucosa. Expert Rev Mol Med 4: 1-17

Kawabata A (2003) [Physiological functions of protease-activated receptor-2]. Nihon Yakurigaku Zasshi 121: 411-420

Kawabata A, Kuroda R, Nishikawa H, Kawai K (1999) Modulation by protease-activated receptors of the rat duodenal motility in vitro: possible mechanisms underlying the evoked contraction and relaxation. Br J Pharmacol 128: 865-872

Kawabata A, Morimoto N, Nishikawa H, Kuroda R, Oda Y, Kakehi K (2000) Activation of protease-activated receptor-2 (PAR-2) triggers mucin secretion in the rat sublingual gland. Biochem Biophys Res Commun 270: 298-302

Kim YH, Ahn DS, Joeng JH, Chung S (2014) Suppression of peripheral sympathetic activity underlies protease-activated receptor 2-mediated hypotension. Korean J Physiol Pharmacol 18: 489-495

Kunzelmann K, Schreiber R, Konig J, Mall M (2002) Ion transport induced by proteinase-activated receptors (PAR2) in colon and airways. Cell Biochem Biophys 36: 209-214

Kwon TH, Jung H, Cho EJ, Jeong JH, Sohn UD (2015) The Signaling Mechanism of Contraction Induced by ATP and UTP in Feline Esophageal Smooth Muscle Cells. Mol Cells 38: 616-623

Malbon CC (2005) G proteins in development. Nature reviews Molecular cell biology 6: 689-701

Molino M, Barnathan ES, Numerof R, Clark J, Dreyer M, Cumashi A, Hoxie JA, Schechter N, Woolkalis M, Brass LF (1997) Interactions of mast cell tryptase with thrombin receptors and PAR-2. J Biol Chem 272: 4043-4049

Morganstern JA, Wang MY, Wershil BK (2008) Direct evidence of mast cell participation in acute acid-induced esophageal inflammation in mice. Journal of pediatric gastroenterology and nutrition 46: 134-138

Murthy KS, Makhlouf GM (1996) Opioid mu, delta, and kappa receptor-induced activation of phospholipase C-beta 3 and inhibition of adenylyl cyclase is mediated by Gi2 and G(o) in smooth muscle. Molecular pharmacology 50: 870-877

Murthy KS, Zhou H, Grider JR, Brautigan DL, Eto M, Makhlouf GM (2003) Differential signalling by muscarinic receptors in smooth muscle: m2-mediated inactivation of myosin light chain kinase via Gi3, Cdc42/Rac1 and p21-activated kinase 1 pathway, and m3-mediated MLC20 (20 kDa regulatory light chain of myosin II) phosphorylation via Rho-associated kinase/myosin phosphatase targeting subunit 1 and protein kinase C/CPI-17 pathway. The Biochemical journal 374: 145-155

Nakagawa O, Fujisawa K, Ishizaki T, Saito Y, Nakao K, Narumiya S (1996) ROCK-I and ROCK-II, two isoforms of Rho-associated coiled-coil forming protein serine/threonine kinase in mice. FEBS letters 392: 189-193

Nam YS, Suh JS, Song HJ, Sohn UD (2013) Signaling pathway of lysophosphatidic Acid-induced contraction in feline esophageal smooth muscle cells. The Korean journal of physiology & pharmacology : official journal of the Korean Physiological Society and the Korean Society of Pharmacology 17: 139-147

Nystedt S, Emilsson K, Wahlestedt C, Sundelin J (1994) Molecular cloning of a potential proteinase activated receptor. Proc Natl Acad Sci U S A 91: 9208-9212

Puetz S, Lubomirov LT, Pfitzer G (2009) Regulation of smooth muscle contraction by small GTPases. Physiology (Bethesda) 24: 342-356

Sekiguchi F, Hasegawa N, Inoshita K, Yonezawa D, Inoi N, Kanke T, Saito N, Kawabata A (2006) Mechanisms for modulation of mouse gastrointestinal motility by proteinase-

activated receptor (PAR)-1 and -2 in vitro. Life Sci 78: 950-957

Shim JO, Shin CY, Lee TS, Yang SJ, An JY, Song HJ, Kim TH, Huh IH, Sohn UD (2002) Signal transduction mechanism via adenosine A1 receptor in the cat esophageal smooth muscle cells. Cellular signalling 14: 365-372

Sohn UD, Han B, Tashjian AH, Jr., Behar J, Biancani P (1995) Agonist-independent, muscle-type-specific signal transduction pathways in cat esophageal and lower esophageal sphincter circular smooth muscle. J Pharmacol Exp Ther 273: 482-491

Sohn UD, Harnett KM, Cao W, Rich H, Kim N, Behar J, Biancani P (1997) Acute experimental esophagitis activates a second signal transduction pathway in cat smooth muscle from the lower esophageal sphincter. The Journal of pharmacology and experimental therapeutics 283: 1293-1304

Somlyo AP, Somlyo AV (2000) Signal transduction by G-proteins, rho-kinase and protein phosphatase to smooth muscle and non-muscle myosin II. The Journal of physiology 522 Pt 2: 177-185

Sriwai W, Mahavadi S, Al-Shboul O, Grider JR, Murthy KS (2013) Distinctive G Protein-Dependent Signaling by Protease-Activated Receptor 2 (PAR2) in Smooth Muscle: Feedback Inhibition of RhoA by cAMP-Independent PKA. PLoS One 8: e66743

Vergnolle N, Macnaughton WK, Al-Ani B, Saifeddine M, Wallace JL, Hollenberg MD (1998) Proteinase-activated receptor 2 (PAR2)-activating peptides: identification of a receptor distinct from PAR2 that regulates intestinal transport. Proc Natl Acad Sci U S A 95: 7766-7771

Yang SJ, An JY, Shim JO, Park CH, Huh IH, Sohn UD (2000) The mechanism of contraction by 2-chloroadenosine in cat detrusor muscle cells. The Journal of urology 163: 652-658

Figure Legends

Fig 1. The contractile response of feline esophageal smooth muscle to trypsin.

A) The esophageal cells under microscopy were isolated with digestion process. From the left to right side in dispersing process was shown. Images were captured by 20 x 10 magnifications. **B)** Cells were stimulated for 60 seconds with the indicated concentration of trypsin. **C)** Cells were incubated with trypsin (10^{-5} M) for the indicated times. **D)** Cells pretreated with 1uM and 10uM FSLLRY for 10 minutes were incubated with trypsin (10^{-5} M) for 60 seconds. **E)** Cells pretreated with 1uM and 10uM FSLLRY for 10 minutes were incubated in trypsin (10^{-5} M) for 5 minutes. Data are expressed as the mean ± S.E.M. of five independent experiments. *P < 0.05 versus control; **P < 0.01 versus control.

Fig. 2. The contractile response of feline esophageal smooth muscle cells to PAR2-AP.

A) Cells were stimulated for 60 seconds with the indicated concentration of PAR2-activating peptide (PAR2-AP). **B)** Cells were incubated with PAR2-AP (10^{-5} M) for the indicated times. Data are expressed as the mean ± S.E.M. of five independent experiments.

Fig. 3. Inhibition of PAR2-AP induced contraction in permeabilized esophageal smooth muscle cells by antibodies against G protein isoforms.

A) Cells were incubated with 400 ng/ml Pertussis toxin (PTX) for 1 hour and then treated with PAR2-AP (10^{-6} M) for 1 minute. **B)** Dispersed esophageal smooth muscle cells were incubated with G protein antibodies (1:200) for 1 hour and then treated with PAR2-activating peptide (10^{-6} M) for 1 minute. **C)** Cells were incubated with 400 ng/ml Pertussis toxin (PTX) for 1 hour and then treated with PAR2-AP (10^{-6} M) for 5 minutes. **D)** Dispersed esophageal smooth muscle cells were incubated with G protein antibodies (1:200) for 1 hour and then treated with PAR2-activating peptide (10^{-6} M) for 5 minutes. Results are expressed as the percentage decrease in cell length compared with the control. Data are

expressed as the mean ± S.E.M. of six independent experiments. *P < 0.05 versus control; **P < 0.01 versus control.

Fig. 4. Inhibition of the PAR2-AP induced contraction of feline esophageal smooth muscle cells. Cells were incubated with a PLC inhibitor (U73122, 10^{-6} M), a Rho kinase inhibitor (Y27632, 10^{-6} M), and a MLCK inhibitor (ML-9, 10^{-5} M) for 10 minutes and a PKC inhibitor (chelerythrine, 10^{-5} M) for 1 minute, and then treated with PAR2-AP (10^{-6} M).

A) Contraction was measured after 60 seconds. **B)** Contraction was measured after 240 seconds. Muscle contraction was measured by scanning micrometry. Data are expressed as the mean ± S.E.M. of six independent experiments. *P < 0.05 versus control; **P < 0.01 versus control.

Fig. 5. Inhibition of PAR2-AP induced contraction in permeabilized esophageal smooth muscle cells against antibodies to PLC isoforms.

A) Cells were incubated with a PLC antibody (1:200) for 1 hour and then treated with PAR2-AP (10^{-6} M) for 1 minute. **B)** Cells were incubated with a PLC antibody (1:200) for 1 hour and then treated with PAR2-AP (10^{-6} M) for 5 minutes. Results are expressed as a percentage decrease in cell length compared with the control. Data are expressed as the mean ± S.E.M. of six independent experiments. *P < 0.05 versus control.

Fig. 6. PAR2 activation induced the upregulation of ROCK-2. Cells were incubated with PAR2-AP (10^{-6} M) for 60 seconds and 240 seconds (4 minutes).

ROCK-2 upregulation was determined using a ROCK-2-specific antibody and confirmed by Western blotting analysis. Western blotting of GAPDH is shown a loading. Data are expressed as the mean ± S.E.M. of five independent experiments. *P < 0.05 versus control; **P < 0.01 versus control.

Fig. 7. PAR2 activation induced the phosphorylation of MLC_{20} and CPI-17.

Cells were incubated with PAR2-AP (10^{-6} M) for 60 seconds and 240 seconds (4 minutes). A) 20kDa-Myosin light chain phosphorylation was determined using a phosphor-specific Ser 19/Thr 18-MLC_{20} ($pMLC_{20}$) antibody and confirmed by Western blotting analysis. B) CPI-17 phosphorylation was determined using a phosphor-specific Thr 38-CPI-17 antibody and confirmed by Western blotting analysis. Data are expressed as the mean ± S.E.M. of six experiments. *P < 0.05 versus control; **P < 0.01 versus control.

Figure

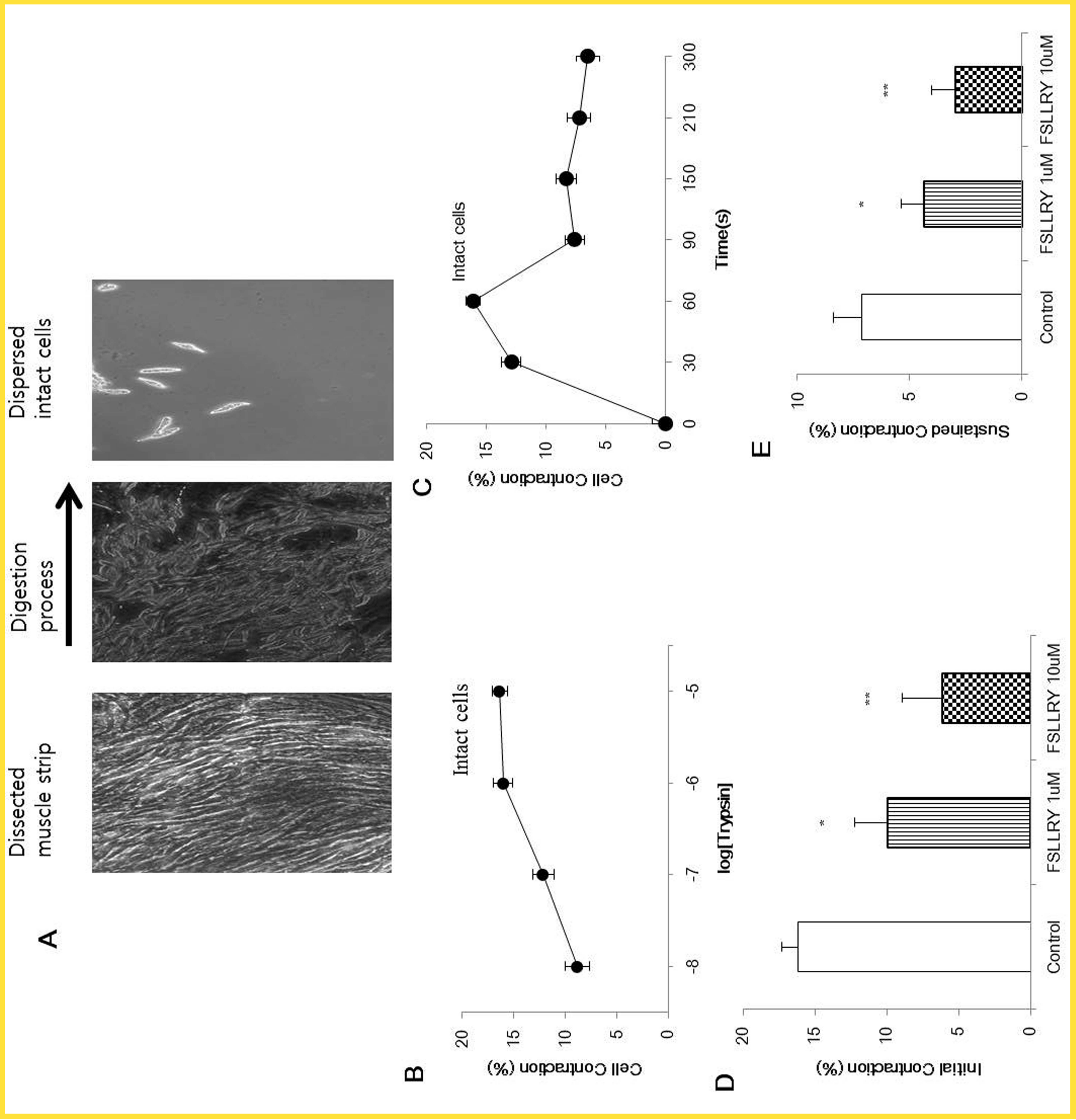

A
Dissected muscle strip
Digestion process
Dispersed intact cells
B
Cell Contraction (%)
Intact cells
log[Trypsin]
C
Cell Contraction (%)
Intact cells
Time(s)
D
Initial Contraction (%)
Control
FSLLRY 1uM
FSLLRY 10uM
E
Sustained Contraction (%)
Control
FSLLRY 1uM
FSLLRY 10uM

Figure

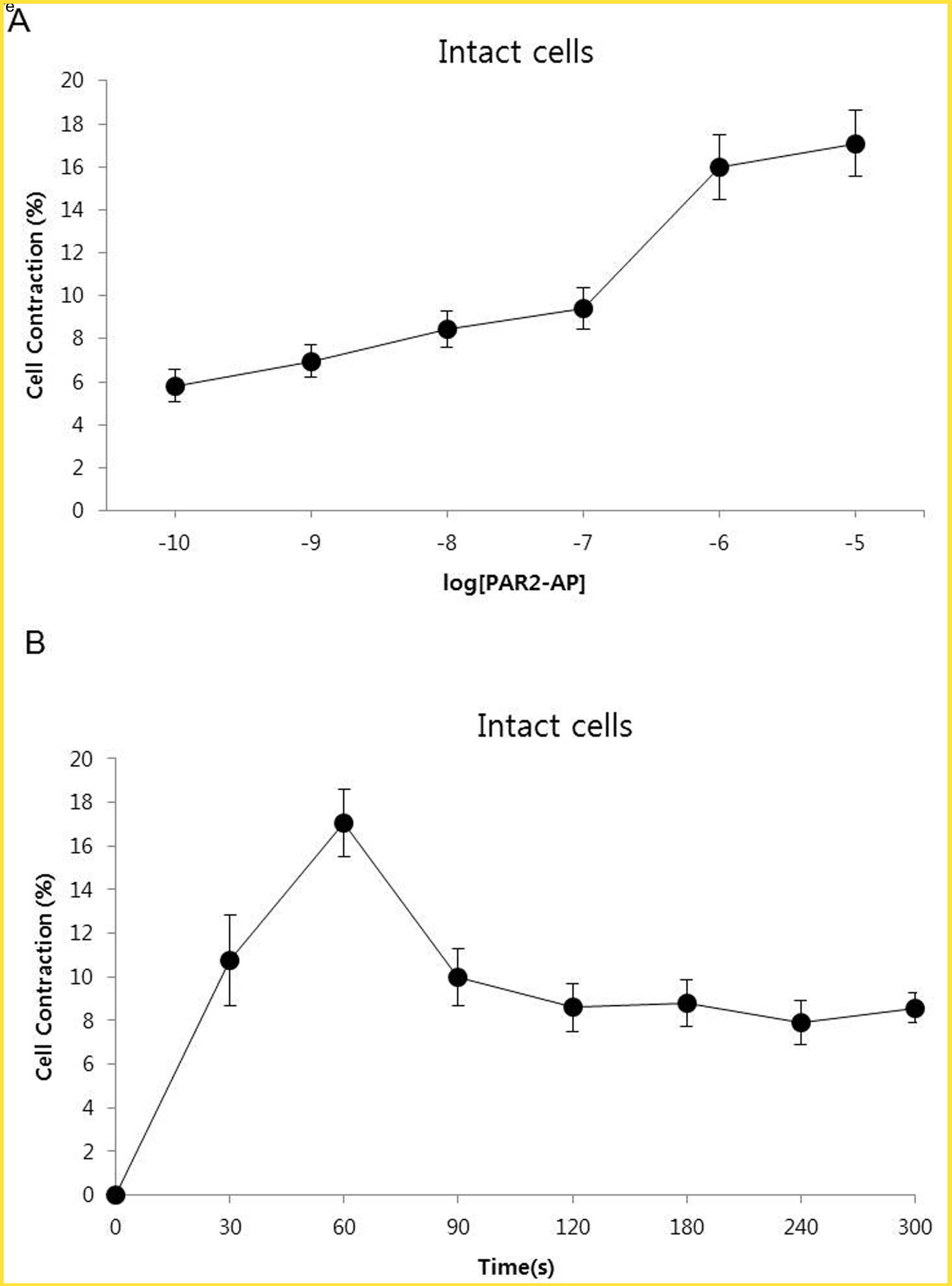
A
Intact cells
Cell Contraction (%)
20
18
16
14
12
10
8
6
4
2
0
-10
-9
-8
-7
-6
-5
log[PAR2-AP]
B
Intact cells
Cell Contraction (%)
20
18
16
14
12
10
8
6
4
2
0
0
30
60
90
120
180
240
300
Time(s)

Figure

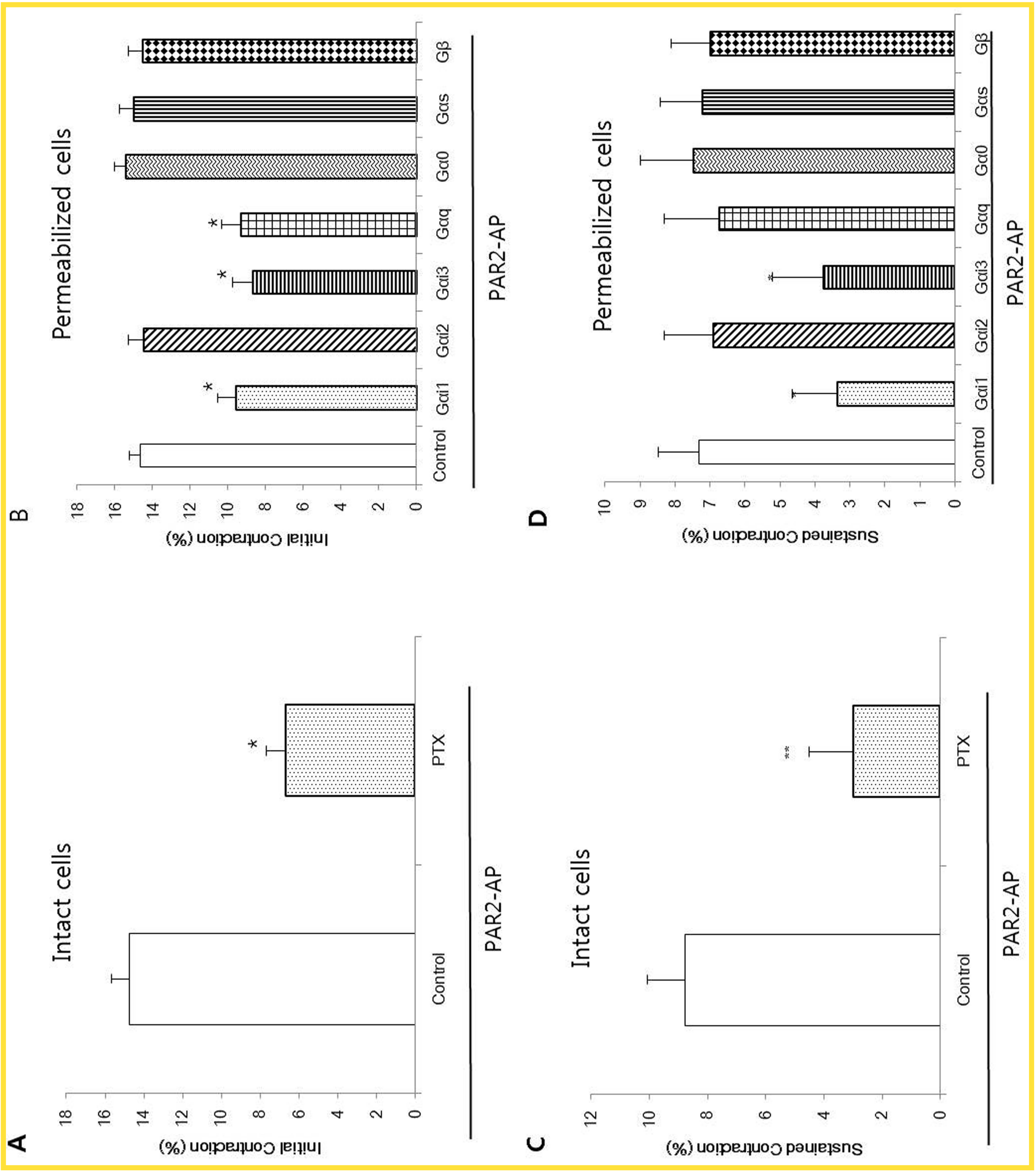
A
Intact cells
Initial Contraction (%)
18
16
14
12
10
8
6
4
2
0
Control
PTX
*
PAR2-AP
B
Permeabilized cells
Initial Contraction (%)
Control
Gαi1
Gαi2
Gαi3
Gαq
Gα0
Gαs
Gβ
PAR2-AP
C
Intact cells
Sustained Contraction (%)
12
10
8
6
4
2
0
Control
PTX
**
PAR2-AP
D
Permeabilized cells
Sustained Contraction (%)
10
9
8
7
6
5
4
3
2
1
0
Control
Gαi1
Gαi2
Gαi3
Gαq
Gα0
Gαs
Gβ
PAR2-AP

Figure

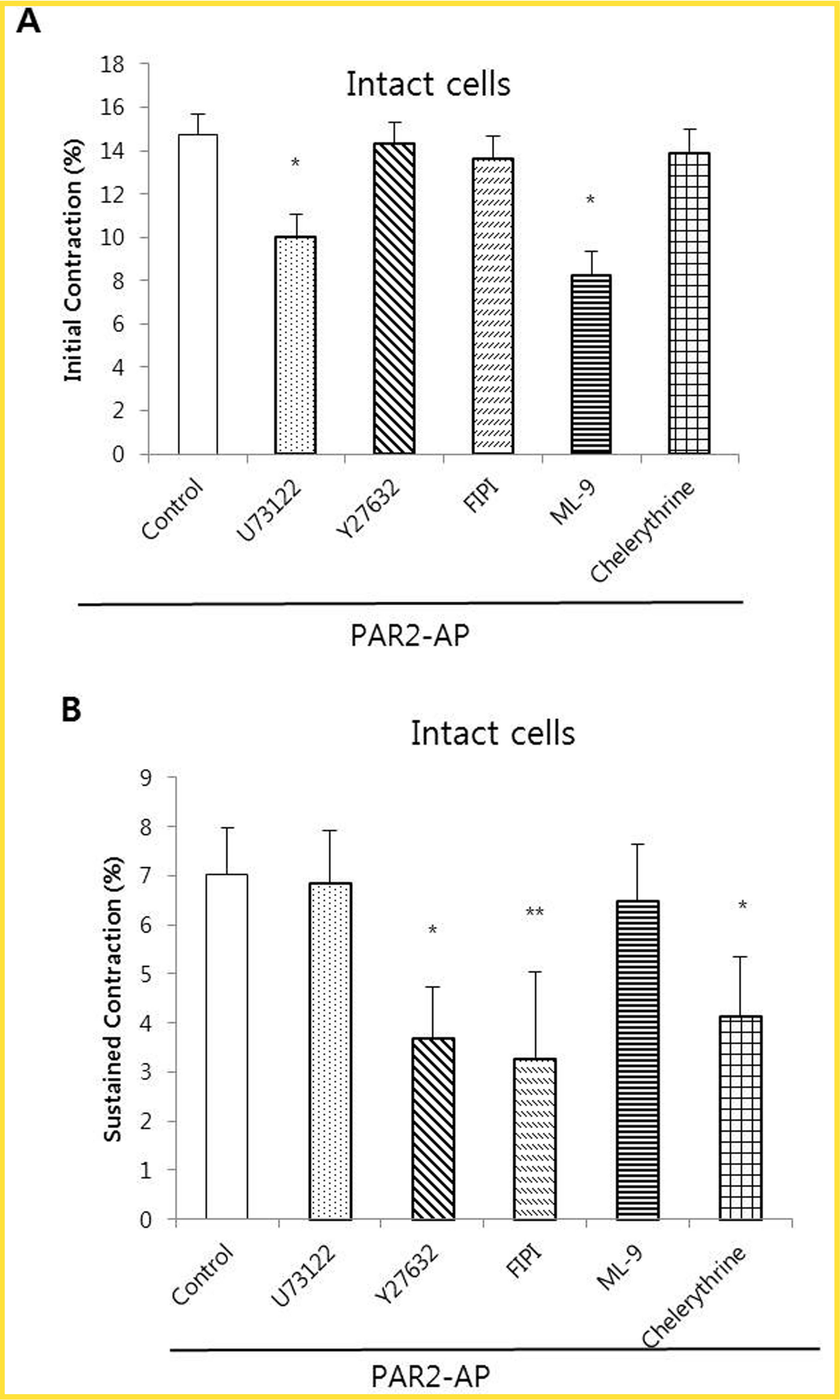
A
Intact cells
Initial Contraction (%)
18
16
14
12
10
8
6
4
2
0
*
*
Control
U73122
Y27632
FIPI
ML-9
Chelerythrine
PAR2-AP
B
Intact cells
Sustained Contraction (%)
9
8
7
6
5
4
3
2
1
0
*
**
*
Control
U73122
Y27632
FIPI
ML-9
Chelerythrine
PAR2-AP

Figure

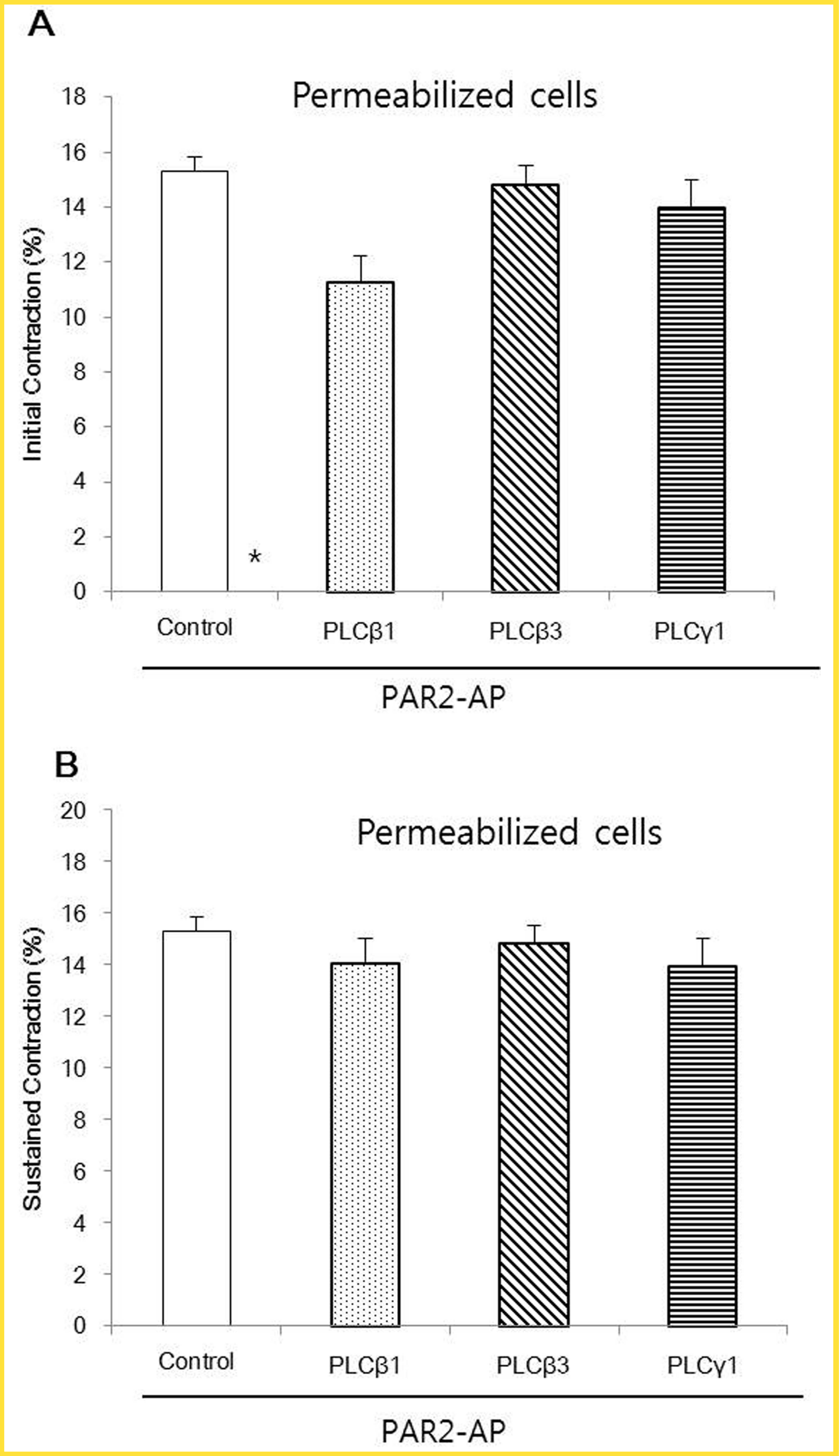
A
Permeabilized cells
Initial Contraction (%)
18
16
14
12
10
8
6
4
2
0
*
Control
PLCβ1
PLCβ3
PLCγ1
PAR2-AP
B
Permeabilized cells
Sustained Contraction (%)
20
18
16
14
12
10
8
6
4
2
0
Control
PLCβ1
PLCβ3
PLCγ1
PAR2-AP

Figure

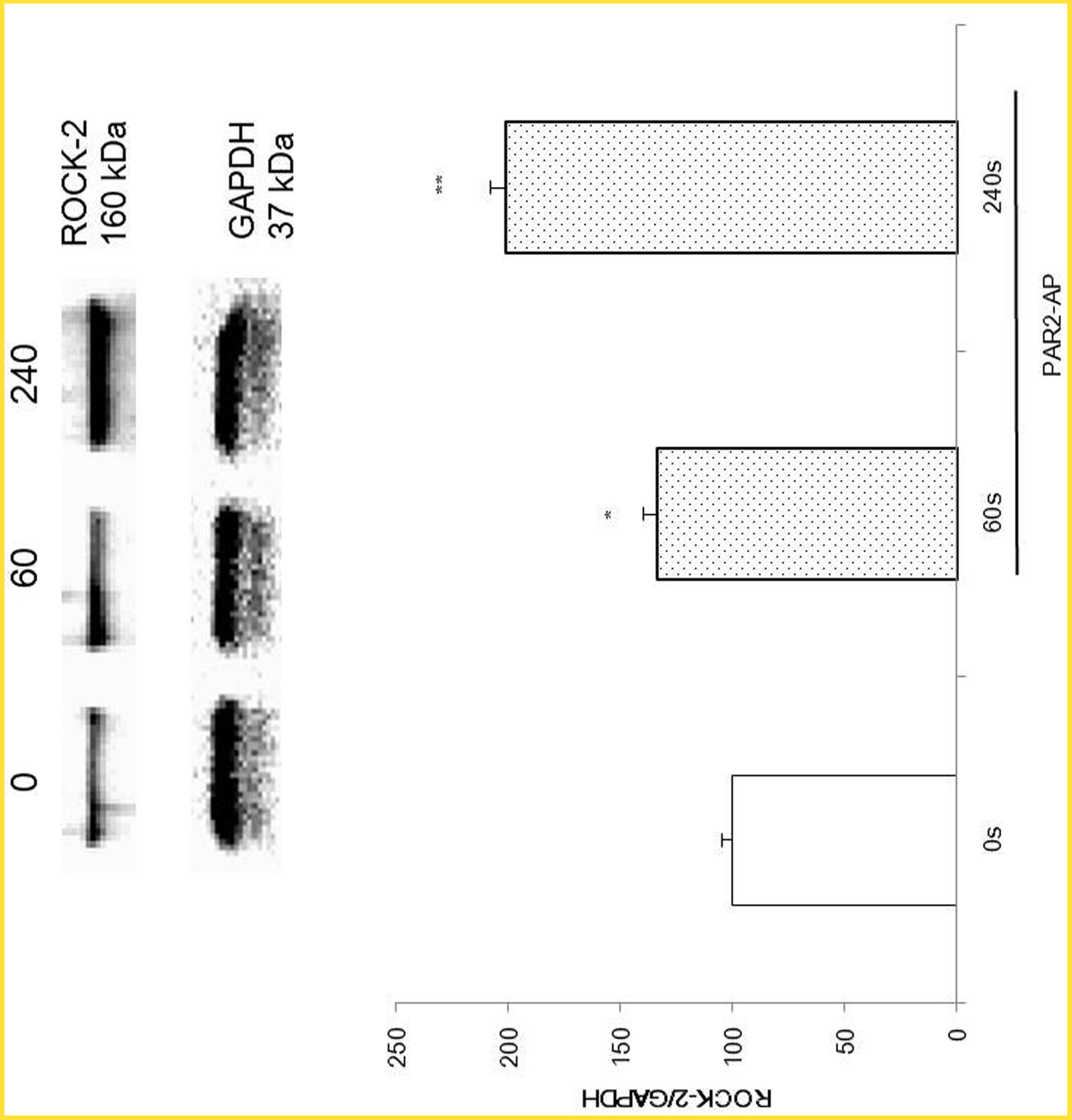
0
60
240
ROCK-2 160 kDa
GAPDH 37 kDa
ROCK-2/GAPDH
0
50
100
150
200
250
*
**
0s
60s
240s
PAR2-AP

Figure

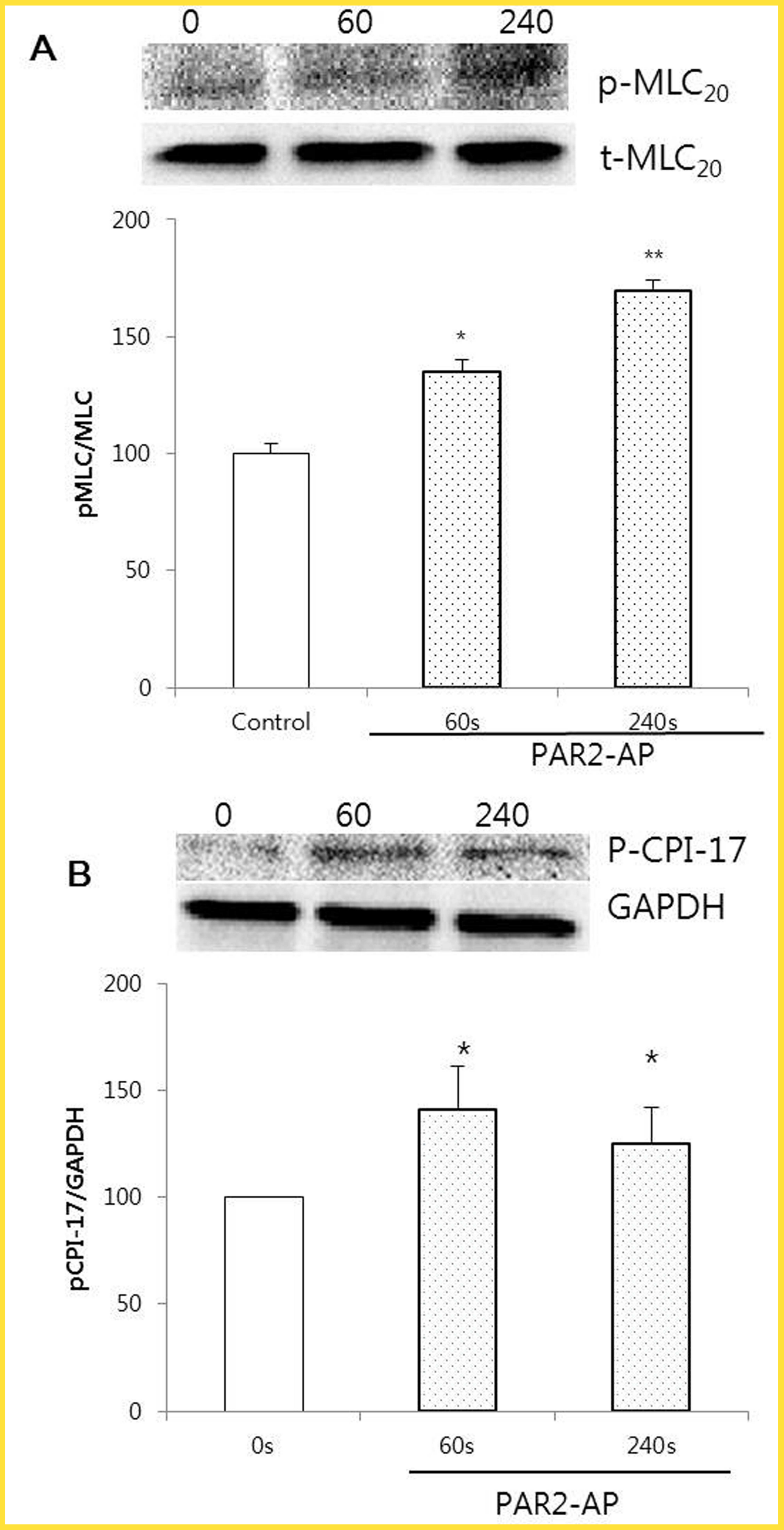
A
0
60
240
p-MLC20
t-MLC20
pMLC/MLC
200
150
100
50
0
*
**
Control
60s
240s
PAR2-AP
B
0
60
240
P-CPI-17
GAPDH
pCPI-17/GAPDH
200
150
100
50
0
*
*
0s
60s
240s
PAR2-AP
