# Supplementary material for: Fake paper identification in the pool of withdrawn and rejected manuscripts submitted to Naunyn–Schmiedeberg’s Archives of Pharmacology
Source: Naunyn Schmiedebergs Arch Pharmacol. 2023 Oct 5;397(4):2171–81. doi: 10.1007/s00210-023-02741-w (PMC10933159; doi:10.1007/s00210-023-02741-w)
Supplement: Supplementary file 8 — Supplementary file8 (PDF 607 KB) [file 210_2023_2741_MOESM8_ESM.pdf]

Figure S8

Color coding:

Yellow highlighted text	The text is identical in the NSAP version and the published version of this paper.
Red highlighted text	There are differences in the text between the NSAP version and the published version of this paper (different content or different wording).
Yellow bordered figure	This figure is identical in both versions of this paper.

Naunyn-Schmiedeberg's Archives of Pharmacology

Naringenin attenuates cerebral ischemia-reperfusion injury through inhibiting oxidative stress and inflammation in diabetic rats

--Manuscript Draft--

**Manuscript Number:**	NSAP-D-19-00523
**Full Title:**	Naringenin attenuates cerebral ischemia-reperfusion injury through inhibiting oxidative stress and inflammation in diabetic rats
**Article Type:**	Original Article
**Corresponding Author:**	Prabhakar orsu<br>GITAM Institute of Pharmacy<br>INDIA
**Corresponding Author Secondary Information:**	
**Corresponding Author's Institution:**	GITAM Institute of Pharmacy
**Corresponding Author's Secondary Institution:**	
**First Author:**	Prabhakar orsu
**First Author Secondary Information:**	
**Order of Authors:**	Prabhakar orsu
	Sharon Amulya Orsu
	Elishamma O
	Ravi P
**Order of Authors Secondary Information:**	
**Funding Information:**	
**Abstract:**	Aim<br><br>Ischemic stroke is one of the important complications of diabetes. Diabetes exacerbate cerebral injury after ischemia and reperfusion. This study was designed to investigate whether the naringenin has a cerebroprotective action against the ischemic reperfusion injury via anti-oxidant and anti-inflammatory mechanisms in diabetic rats.Methods<br><br>Diabetes was induced by Streptozocine (50 mg/kg) intraperitoneal injection at once. Medial carotid artery occlusion (30 min) and reperfusion (3 hr) was employed to induce cerebral infarction in diabetic rats. The animals were divided in to groups as: normal, sham, ischemia-reperfusion and naringenin treated (50, 100, 150 and 200 mg/kg).These were used for evaluation of percentage of cerebral infarction. Further, 200 mg/kg dose was selected for the estimation of inflammatory biomarkers such as Tumor necrosis factor-α, Interlukin-6, Interlukin-10 and oxidative stress biomarkers such as malondialdehyde, superoxide dismutase, and catalase were estimated and histopathological changes were studied.Results<br><br>Dose dependent reduction in percentage of cerebral infarction was observed in narigenin treated groups. With Naringenin 200 mg/kg dose, inflammatory and oxidative stress markers like Tumor necrosis factor-α, Interlukin-6, myeloperoxidase and malondialdehyde levels were distinctively reduced and there was a remarkable increased levels of anti-inflammatory and anti-oxidant markers like Interlukin-10,catalase, and superoxide dismutase.Conclusion<br><br>Collectively, these findings demonstrate that the mechanism(s) responsible for a cerebroprotective effect of naringenin against the ischemic reperfusion injury in the diabetic rats involves anti-oxidant and anti-inflammatory actions.

Powered by Editorial Manager® and ProduXion Manager® from Aries Systems Corporation

**Suggested Reviewers:**	raja s<br>sraja61@gmail.com

Powered by Editorial Manager® and ProduXion Manager® from Aries Systems Corporation

Manuscript
Click here to view linked References
Click here to access/download;Manuscript;naringenin Manuscript AP.doc

Naringenin attenuates cerebral ischemia-reperfusion injury through inhibiting oxidative stress and inflammation in diabetic rats

Orsu Prabhakar*[1] Orsu.SharonAmulya[2], Elishamma.O[2], Ravi.P[2]
*[1] GITAM Institute of Pharmacy, Rushikonda,Visakhapatnam, India,530045.
[2]AD research institute of Stroke and Neurological disorders, Visakhapatnam, India 530045

* Corresponding author
Orsu.Prabhakar
GITAM Institute of Pharmacy
Visakhapatnam, Andhra Pradesh, India.530045
Email: orsuprabhakar@gmail.com
Mobile: 91-9912988579.

Abstract

Aim: Ischemic stroke is one of the important complications of diabetes. Diabetes exacerbate cerebral injury after ischemia and reperfusion. This study was designed to investigate whether the naringenin has a cerebroprotective action against the ischemic reperfusion injury via anti-oxidant and anti-inflammatory mechanisms in diabetic rats.

Methods: Diabetes was induced by Streptozocine (50 mg/kg) intraperitoneal injection at once. Medial carotid artery occlusion (30 min) and reperfusion (3 hr) was employed to induce cerebral infarction in diabetic rats. The animals were divided in to groups as: normal, sham, ischemia-reperfusion and naringenin treated (50, 100, 150 and 200 mg/kg).These were used for evaluation of percentage of cerebral infarction. Further, 200 mg/kg dose was selected for the estimation of inflammatory biomarkers such as Tumor necrosis factor-α, Interlukin-6, Interlukin-10 and oxidative stress biomarkers such as malondialdehyde, superoxide dismutase, and catalase were estimated and histopathological changes were studied.

Results: Dose dependent reduction in percentage of cerebral infarction was observed in narigenin treated groups. With Naringenin 200 mg/kg dose, inflammatory and oxidative stress markers like Tumor necrosis factor-α, Interlukin-6, myeloperoxidase and malondialdehyde levels were distinctively reduced and there was a remarkable increased levels of anti-inflammatory and anti-oxidant markers like Interlukin-10,catalase, and superoxide dismutase.

Conclusion: Collectively, these findings demonstrate that the mechanism(s) responsible for a cerebroprotective effect of naringenin against the ischemic reperfusion injury in the diabetic rats involves anti-oxidant and anti-inflammatory actions.

Keywords: Diabetes: Ischemia-reperfusion injury: Inflammation: Naringenin: Oxidative stress.

1. Introduction

Stroke is the second leading cause of death and long-term disability in the world (Donkor 2018). Diabetes is the major risk factor in ischemic stroke. The diabetes in ischemic reperfusion state can increase the inflammation and oxidative stress induced by reperfusion (Shukla et al. 2017). There is an increased mortality rate in patients with diabetes associated cerebrovascular accident (ischemic stroke and intra cerebral hemorrhage) and are at more risk of suffering with organ damage and ischemic events (Chen et al. 2016). In acute stroke, thrombolysis plays as life saving therapy and helps in reperfusion. Although reperfusion is needed in ischemic stroke, it can exaggerate the condition causing further damage through inflammation and reactive oxygen species released during reperfusion. This can be further augmented by diabetes making the condition more worsen. These implications associated with reperfusion injury have made the active attention to it. The pathological aspects of reperfusion injury are related with oxidative stress, leukocyte infiltration, damage to blood brain barrier, inflammation, nitric oxide release, platelet activation and apoptosis (Maiocchi et al. 2018). Worsening of clinical and laboratory outcomes are seen in ischemic reperfusion injury patients with diabetes (Canbaz et al. 2003). The intervention with anti-inflammatory and anti-oxidant agents was thought to be beneficial in treating cerebral ischemia reperfusion injury. A polyphenol compound, Naringenin which is extracted from grape skin was reported to be having many beneficial properties like anti-oxidant, anti-inflammatory, anti aging, antidiabetic and controlling apoptosis (Shahidi et al. 2018; Yahfoufi et al. 2018; Prithviraj 2018). In the present study we made an attempt to investigate the protective role of Naringenin in cerebral ischemia reperfusion injury in Wistar diabetic rats by employing medial carotid arteries occlusion for 20 min and reperfusion for 3 hours.

2. Material and Methods

2.1 Chemicals:

Rat TNF-α ELISA kit (Assyrapro, USA), Rat IL-6 ELISA kit (Eaab, USA), Rat IL-10 ELISA kit (Assyrapro, USA), Rat IL-6 ELISA kit (Eaab, USA), Naringenin and Streptozocine (STZ) procured from Sigma Aldrich, India, Phenazine methosulphate (PMS) (Loba chemicals, India), Thiobarbituric acid (TBA)(Loba chemicals, India),Nicotinamide adenine dinucleotide phosphate reduced form (NADPH) (Sisco chemicals, India), Nitoblue tetrazolium (NBT) (SD fine chemicals, India), O-di anisidine di hydrochloride (Sigma Aldrich, India), 2,3,5-triphenyltetrazolium chloride (TTC) (Sigma Aldrich, India). Other chemicals used were of analytical grade supplied from local agencies.

2.2 Animals:

Adult Wistar rats (220–310 g) were obtained from Gentox Bio Pvt. Ltd., Hyderabad, Andhra pradesh, India. Animals were maintained under a 12/12-hr light/dark cycle, in an ambient temperature (24±1°C) colony room. Animals were provided with a constant supply of food and water. Animal care followed the official governmental guidelines in compliance with the CPCSEA, New Delhi and experimental protocols were conducted at GITAM Institute Pharmacy with the approval of the Institutional Ethical Committee (Ref no:1287/PO/Re/S/09/CPCSEA) of GITAM deemed to be University, Visakhapatnam, India.

2.3. Experimental procedure

2.3.1 Induction of diabetes:

Diabetes was induced in the rats by a single dosage of Streptozotocin (STZ) (50mg/kg, i.p.) and they were also administered with 50% w/v sucrose solution. After 6 days of STZ injection the animals were subjected to cerebral ischemia- reperfusion injury, followed by collection of blood samples from the tail vein of rats for the estimation of glucose levels. The blood glucose levels of > 250mg/dl in rats were considered to be diabetic and such rats are included in the study. Spectrophotometrical estimation of serum glucose is employed by using commercially available kits (J. Mitra & Co. Ltd. New Delhi, India).

2.3.2. Experimental Induction of Focal Cerebral Ischemia

The occlusion of right MCA was carried out using an intraluminal filament model (Longa 1989). Rats were anesthetized by using thiopental sodium (30 mg/kg). Cervical vertebrae and the common carotid arteries were then exposed and their separation from vagus nerve was performed. The tip of 4–0 nylon monofilament was rounded with the use of an open flame followed by insertion into the external carotid artery and advanced into the internal carotid artery until a slight resistance was felt, which indicates the filament has passed beyond the proximal segment of the anterior cerebral artery. The origin of MCA was blocked by this intraluminal filament and blood flow from the internal carotid artery, anterior cerebral artery and the posterior cerebral artery. The rectal temperature was maintained at 37± 0.5°C with a feedback-controlled heating-pad. Animals which preserved righting reflex or which convulsed during the ischemic episode were excluded from the study. After 30 min of induced ischemia the filament was slowly withdrawn until tip reached external carotid artery. Animals were then closely monitored in their cages until they recovered from anesthesia. In sham-operated rats, the external carotid artery was surgically prepared for insertion of the filament, however the filament was not inserted.

2.3.3 Measurement of percentage cerebral infarct volume:

Naringenin was dissolved in 5% DMSO and administered intraperitoneally 5 min before reperfusion. Diabetic Rats were randomly divided into groups: sham, I/R (Ischemia-reperfusion), I/R+ vehicle and I/R+NAG (Naringenin treated) (50, 100, 150, 200 mg/kg). Each group consists of 6 animals.

The coronal sections of 2 mm thickness were taken from the quickly removed brains after the predetermined time point of ischemia-reperfusion. Each slice was immersed in a 1.0% solution of 2, 3, 5-triphenyltetrazolium chloride (TTC) for 30min. Pale necrotic infarcted tissue and healthy, normal tissue stained dark red were separated and weighed. The % infarction was calculated (Orsu et al. 2013).

2.3.5 Histology:

As previously stated, the brain tissues were obtained and prepared for histological examination. Each brain was postfixed in a 10% formalin solution. After that brain tissue was embedded with paraffin and coronal sections were made in to 5-µm thickness. To assess the histopathological change, the sections were further subjected to hematoxylin and eosin staining.

2.3.5 Estimation of oxidative stress and inflammation markers

In selected group of animals, naringenin (50, 100, 150 and 200 mg/ kg) was used for estimation of oxidative inflammation parameters. Brain tissues were homogenated and the suppernate was used for the estimation of MDA (malondialdehyde) (Okhawa et al. 1979), SOD (superoxide dismutase) (Kakkar et al. 1984), CAT (catalase) (Aebi 1974), MPO (myeloperoxidase) (Mullane et al. 1985), IL-10 (Liu et al. 2009), IL-6 (Saito et al. 1996) and TNF-α (Liu et al. 2009).

2.4 Statistical analysis

All values were expressed as mean ± SEM and analyzed by one-way analysis of variance (ANOVA) followed by Tukeys t test ($P<0.05$) using prism software 5.0.

3. Results

.

3.1 Effect of Naringenin on cerebral infarction volume

There was a significant increase in percent cerebral infarction in I/R (59%) as evident from TTC stained sections as compared to sham control group (6 %). Naringenin at 50,100,150 and 200 mg/kg significantly reduced the infarction volume (37,30,19 and 11 %) was observed as compare to I/R . Naringenin produced a dose dependent effect by reducing cerebral infarction volume. Results were shown in Table-1

3.2 Histology:

Electron microscopy was used to study the structural changes of tissue. I/R rats showed abnormal structural tissue changes in terms of dens eosinophilic cytoplasm and triangular nucleus. In contrast, these abnormal structural changes were recovered significantly with Naringenin treatment (50,100,150 and 200 mg/kg). Histological changes were shown in Fig1.

3.2. Effect of Naringenin on Brain oxidative stress parameters

Oxidative stress markers were messured by spectrophotometricaly. I/R rats showed significant increased levels of MDA and significant declined levels of SOD, and CAT as compared to sham control group. However, naringenin treated rats showed significant reduced levels of MDA and siginificant increased levels of SOD and CAT as compared to I/ R. Results were shown in Table-2

3.3 Effect of Naringenin on brain inflammatory parameters

Inflammatory markers were measured by ELISA. I/R rats showed remarkable increased levels of TNF-alpha, IL-6 and MPO and IL-10 levels were significantly declined as compared to sham control. In constrat, Naringenin treated rats showed TNF-alpha, IL-6 and MPO levels were distinctively declined and IL-10 levels were significantly increased as compare to I/R. Results were shown in Table-3

4. Discussion

The present study was involved to investigate the possible protective mechanisms of naringenin against ischemia-reperfusion injury in diabetic rats. The clinical outcomes are more worsened in diabetic associated ischemic stroke. Both diabetes and ischemia-reperfusion are involved in release of ROS and inflammation. This can lead to further serious pathological events in the ischemic tissue injury.

Few studies have demonsteted that anti-inflammatory and anti oxidant agents may be useful in limiting the reperfusion injury (Khan et al. 2004; Lakhan et al. 2009). Recently Naringenin showed neuroprotective role in rats (Chen et al. 2019; Selcuk et al. 2014). Recent researchers also suggested that medial carotid arteries occlusion (MCA) can induce brain ischemia in rats (Tosaki et al. 1994). MCA occlusion and reperfusion is followed by the pathological events such as inflammation and free radicals generation to cause tissue apoptosis in diabetic rats. Naringenin may be used in wide variety of conditions including diabetes, cancer, cardiovascular and neurological disorders (Bahare et al. 2019). Naringenin exerts multiple biological effects, including anti ageing, anti oxidant, anti-inflammatory, antiproliferative and antiplatelet effects (Prithviraj 2018). Recent studies have identified that administration of naringenin is involved in activation of adenosine receptors $A_1$and A_2, activation of the mitogen-activated protein kinase (MAPK) pathway and myocardial induction of vascular endothelial growth factor, Flk-1, endothelial nitric oxide synthase, thioredoxin-1 and Hemeoxigenase-1 (Suganya et al. 2016). Some studies suggest that Naringenin can impart considerable protection against ischemic injury by activating multitudes of cytoprotective signaling pathways in cerebrum and myocardium (Yahfoufi et al. 2018; Suganya et al. 2016). Naringenin has shown several beneficial effects on cardiovascular system after focal ischemia and reperfusion injury by improving left ventricular functions and reducing the size of infarct. It also reduced blood glucose level and cardiomyocyte cell death in conjunction with increased Mn-SOD activity in diabetic rat myocardium (Qiong et al. 2016).

Evaluating the area of infarction in brain gives the estimate of cerebral damage and thus the consequences of cerebral ischemia which leads to neurological impairment can be determined. The size of infarct was determined by staining of coronal sections with TTC. TTC helps in distinguishing the viable cells as deep red color and infarct tissue as unstained cells (pale whitish tissue). In the present study we noticed the percentage of infarction in I/R diabetic rats was significantly increased, whereas a significant decreased percentage is seen in Naringenin treated diabetic rats. A dose dependent reduction in percent infarction was observed in Naringenin (50, 100, 150, 200 mg/kg) treated rats. These results were in accordance with the earlier reports (Orsu et al. 2013). In the histological examination, the presence of dense eosinophilic cytoplasm and dark stained triangular nuclei represent the ischemic cells (Della-Morte et al. 2009). The presence of these ischemic cells manifestations was diminished in naringenin treated rats as shown in Figure-1. These results of limiting the cerebral infarction suggest the cerebroprotective properties of naringenin.

The diabetes in ischemia-reperfusion injury can worsen the exposure of brain cells to free radicals by oxidative metabolism and inflammation. Lipid peroxidation is generated by free radicals and initiates the release of end product, malondialdehyde (MDA) which determines the oxidative stress. SOD and CAT are the most important endogenous anti-oxidative enzymes which play a key role in scavenging the free radicals. However, excess free radicals in the ischemic condition limit the levels of SOD and CAT altering the anti-oxidative defensive mechanism. This is supported by well known fact that involvement of increased levels of free radicals in diabetes associated with ischemia. When compared to normal rats the oxidative stress is greater in ischemic rats associated with diabetes. In the present study, we identified remarkable reduction of SOD, CAT and significant increase in levels of MDA in I/R diabetic rats. In contrast, SOD, CAT levels were increased and MDA levels were decreased significantly in naringenin treated diabetic rats which demonstrates the strengthened oxidative defense mechanisms and reduced lipid peroxidation by naringenin. In supporting this, several studies have reported the modulatory effect of naringenin on lipid peroxidation and antioxidant enzymes following CNS injuries such as ischemia/hypoxia (Alam et al. 2014; Yousuf et al. 2007). Therefore we suggest that naringenin has cerebroprotective action against cerebral ischemia and reperfusion injury through anti oxidative mechanism.

The scientific evidence suggests that neuronal injury and cerebral infarction can be a consequence of post-ischemic inflammation. During the inflammation there is an involvement of endogenous mediators (cytokine and chemokines) and circulating leukocytes. Cytokines and chemokines act as primary and secondary mediators respectively in attracting the leucocytes in the inflammatory condition (Gouwy et al. 2005). In addition, cytokines and chemokines are produced by activated astrocyes and microglia (Rock et al. 2004). All these factors collectively appear to be responsible for accumulation of inflammatory cells in injured brain tissue. After ischemic reperfusion injury the important cytokines like TNF-α, IL-1β and IL-6 initiate the inflammatory mediators and induce expression of other cytokines. Increased levels of TNF-α, IL-6 and IL-1β are observed in ischemic brain and considered as a part of tissue damaging response in ischemia and reperfusion injury (Lakhan et al. 2009; Yasuda et al. 2011). The expression of TNF alpha and IL-1β in the injured brain tissues is inhibited by IL-10 (Lakhan et al. 2009). The activity of IL-10 as an anti inflammatory cytokine in cerebral ischemia was reported by many studies (Liu et al. 2009). In the present study, we noticed a significant increased MPO, IL-6 and TNF-α levels with decreased IL-10 levels in I/R diabetic rats. In contrast TNF-α, IL-6 and MPO levels were significantly decreased and IL-10 levels increased in naringenin treated diabetic rats. These results were in accordance with the earlier reports (Yasuda et al. 2011; Yousuf et al. 2007). The potent anti- inflammatory effect of naringenin is supported by the significant attenuated MPO, IL-6, TNF-α levels and increased IL-10 levels in ischemia-reperfusion injury. Therefore naringenin is thought to be having cerebroprotective action through anti inflammatory effect.

5. Conclusion

The present study reveals that the cerebroprotective activity of naringenin by declining cerebral infarct percentage and modification of histological abnormalities. Naringenin has also showed suppressing effects against oxidative stress and inflammation markers which were elevated by cerebral ischemia-reperfusion injury. These findings suggests that naringenin has a protective potential effect against cerebral ischemic stroke via anti oxidant and anti inflammatory mechanism(s)and further supports the possible use of naringenin as a therapeutic agent to ameliorate cerebral infarction.

Author contribution statement

OP conceived and designed research. OP,OS and EO conducted experiments. OP contributed new reagents or analytical tools. OP,OS,EO and RP analyzed data. OP,OS wrote the manuscript. All authors read and approved the manuscript.

Acknowledgement:

The authors express sincere thanks to Gitam Institute of Pharmacy for timely support in accomplishment of this study

Compliance with Ethical Standards

This study was approved by the animal Institutional ethical committee of GITAM Deemed to be University, India. Approved Reference No: IAEC/GIP-1287/OP-F.

Conflict Of Interest: Not Applicable.

SOURCE OF SUPPORT: Nil

4. References

Aebi H (1974) Catalase:Methods in enzymatic analysis Vol.II (ed.H.U. Bergmer) 673-84.

Alam M A, Subhan N, Rahman M. M, Uddin S. J, Reza H. M Sarker S. D (2014) Effect of citrus flavonoids, naringin and naringenin, on metabolic syndrome and their mechanisms of action. Advances in nutrition (Bethesda, Md.) *5*(4): 404–417. doi:10.3945/an.113.005603
.

Bahare Salehi , Patrick Valere Tsouh Fokou, Mehdi Sharifi-Rad, Paolo Zucca, Raffaele Pezzani, Natália Martin, Javad Sharifi-Rad (2019) The Therapeutic Potential of Naringenin: A Review of Clinical Trial. Pharmaceuticals 12;11. doi:10.3390/ph12010011.

Canbaz S, Duran E (2003) Ischaemia-reperfusion studies and diabetes mellitus. Br J Anaesth 91:158-9.

Chen C, Wei YZ, He XM, Li DD, Wang GQ, Li JJ, Zhang F (2019) Naringenin Produces Neuroprotection Against LPS-Induced Dopamine Neurotoxicity via the Inhibition of Microglial NLRP3 Inflammasome Activation Front Immunol. 10:936. doi: 10.3389/fimmu.2019.00936.

Chen R, Ovbiagele B, Feng W (2016) Diabetes and Stroke: Epidemiology, Pathophysiology, Pharmaceuticals and Outcomes. The American journal of the medical sciences 351(4): 380–386. doi:10.1016/j.amjms.2016.01.011.

Della-Morte D, Dave KR, DeFazio RA et al (2009) Resveratrol pretreatment protects rat brain from cerebral ischemic damage via a sirtuin 1-uncoupling protein 2 pathway. Neuroscience 159: 993-1002.

Donkor E. S (2018) Stroke in the 21st Century: A Snapshot of the Burden, Epidemiology, and Quality of Life. Stroke research and treatment 2018: 3238165. doi:10.1155/2018/3238165.

Gouwy M, Struyf S, Proost P, Van Damme J (2005) Synergy in cytokine and chemokine networks amplifies the inflammatory response. Cytokine Growth Factor Rev 16: 561-80.

Kakkar P, Das B, Viswanathan PN (1984) A modified spectrophotometric assay of superoxide dismutase. Ind J Bio Chem Biophys 21: 130-2.

Khan M, Siphon B, Jatana M, Giri S et al (2004) Administration of N-acetylcysteine after focal cerebral ischemia protects brain and reduces inflammation in a rat model of experimental stroke. J Neurosci Res 76: 519-7.

Lakhan SE, Kirchgessner A, Hofer M (2009) Inflammatory mechanisms in ischemic stroke: therapeutic approaches. J Transl Med 7: 97.

Liu N, Chen R, Du H, Wang J, Zhang Y, Wen J (2009) Expression of IL-10 and TNF-alpha in rats with cerebral infarction after transplantation with mesenchymal stem cells. Cell Mol. Immunol 6: 207-13.

Longa EZ, Weinstein PR, Carlson S, Cummins R (1989) Reversible middlecerebral artery occlusion without craniectomy in rats. Stroke 20:84– 91.

Maiocchi S, Alwis I, Wu MCL, Yuan Y, Jackson SP (2018) Thromboinflammatory Functions of Platelets in Ischemia-Reperfusion Injury and Its Dysregulation in Diabetes. Semin Thromb Hemost 44(2):102-13.

Mullane KM, Bruce Smith ND (1985) Myeloperoxidase Activity as a Quantitative Assessment of Neutrophil Infiltration into lschemic Myocardium. Journal of Pharmacological methods 14: 157-7.

Okhawa H, Ohishi N, Yagi K (1979) Assay of lipid peroxides in animal tissue by thiobarbituric acid reaction. Anal Biochem 95: 351–8.

Orsu P, Murthy BV, Akula A (2013) Cerebroprotective potential of resveratrol through anti-oxidant and anti-inflammatory mechanisms in rats. J Neural Transm. 120(8): 1217-23. doi: 10.1007/s00702-013-0982-4.

Prithviraj Karak (2018) Biological activities of flavonoids: an overview. International journal of pharmaceutical sciences and research 3: 1567-74.

Qiong You, Ziyun Wu, Bin Wu, Chang Liu (2016) Naringin protects cardiomyocytes against hyperglycemia-induced injuries in vitro and in vivo. Journal of endocrinology 230(2): JOE-16-0004.

Rock RB, Gekker G, Hu S et al (2004) Role of microglia in central nervous system infections. Clin Microbiol Rev 17: 942-64.

Saito K, Suyama K, Nishida K, Sei Y, Basile AS (1996) Early increases in TNF-alpha, IL-6 and IL-1 beta following transient cerebral ischemia in gerbil brain. Neurosci Lett 206:149–2.

Shahidi F, Yeo J (2018) Bioactivities of Phenolics by Focusing on Suppression of Chronic Diseases: A Review. International journal of molecular sciences 19(6): 1573. doi:10.3390/ijms19061573.

Selcuk Kara, Baran Gencer, Turan Karaca, Hasan Ali Tufan, Sedat Arikan, Ismail Ersan, Ihsan Karaboga, Volkan Hanci (2014) Protective Effect of Hesperetin and Naringenin against Apoptosis in Ischemia/Reperfusion-Induced Retinal Injury in Rats. The Scientific World Journal Article vol. 2014 ID 797824: 8 pages. https://doi.org/10.1155/2014/797824.

Shukla V, Shakya A. K, Perez-Pinzon M. A, & Dave K. R (2017) Cerebral ischemic damage in diabetes: an inflammatory perspective. Journal of neuroinflammation 14(1): 21. doi:10.1186/s12974-016-0774-5.

Yahfoufi N, Alsadi N, Jambi M, Matar C (2018) The Immunomodulatory and Anti-Inflammatory Role of Polyphenols Nutrients 10(11): 1618. doi:10.3390/nu10111618.

Suganya N, Bhakkiyalakshmi E, Sarada D.V.L, Ramkumar K.M (2016) Reversibility of endothelial dysfunction in diabetes: role of polyphenols. British Journal of Nutrition 116(2):223-46.

Tosaki A, Szerdahelyi P, Joo F (1994). Treatment with ranitidine of ischemic brain edema. Eur J Pharmacol. 264: 455-8.

Yasuda Y, Shimoda T, Uno K et al (2011) Temporal and sequential changes of glial cells and cytokine expression during neuronal degeneration after transient global ischemia in rats. J Neuroinflammation 8:70.

Yousuf S, Atif.F, Ahmad M et al (2007) Selenium plays a modulatory role against cerebral ischemia-induced neuronal damage in rat hippocampus. Brain Res 1147: 218–25.

Figure

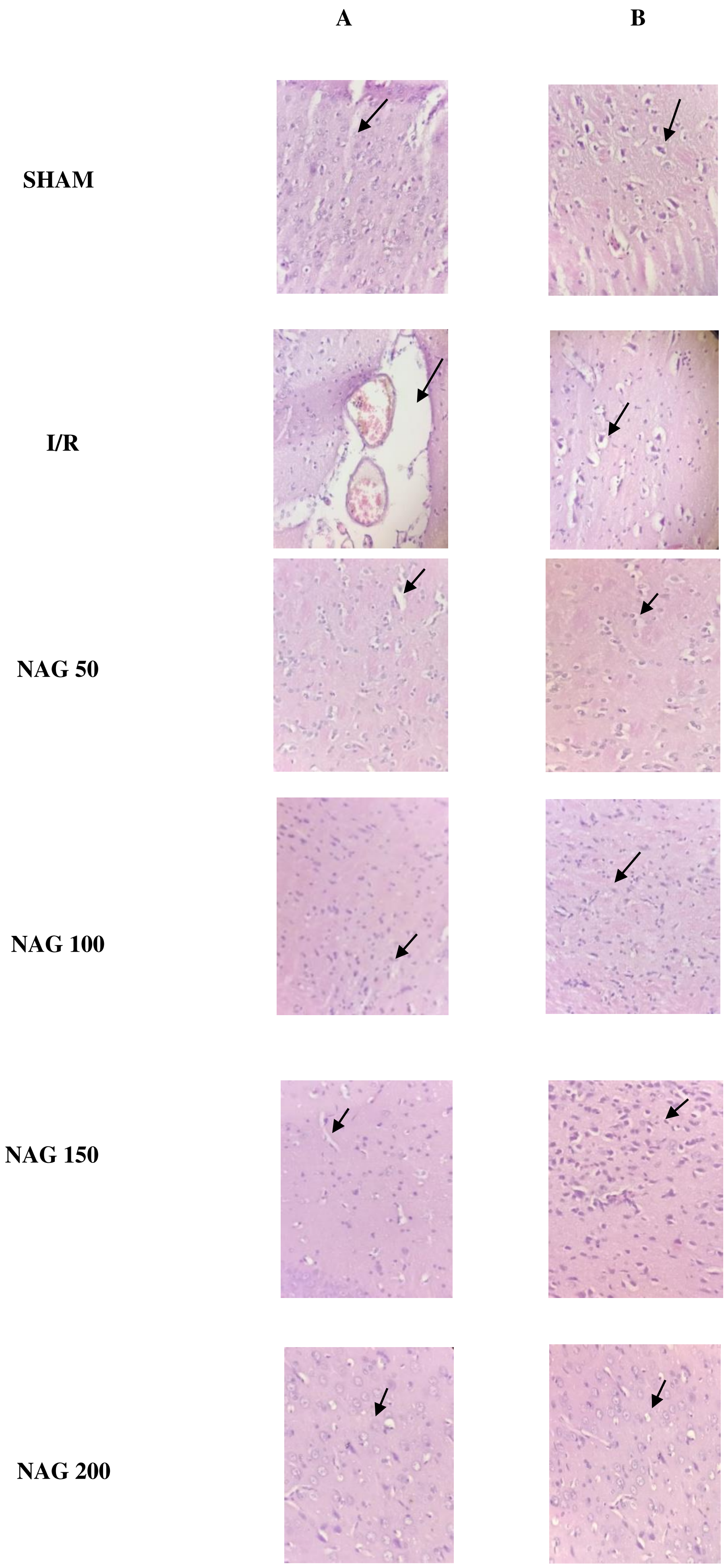

Fig: 1 Histology of brain coronal sections of diabetic rat:

A: indicates images showing eosinophlic cytoplasm and B: indicates images showing neuron nucleus
I/R indicate ischemia and reperfusion. NAG indicates Naringenin treated (50,100,150 and 200 mg/kg i.p.)
In I/R, appearance of triangular nucleus and dense eosinophilic cytoplasm and significant recovery from formation of dense eosinophilic and triangular nucleus in Naringenin treated group.

Table 1: Effect of Naringenin on percentage cerebral infarction in diabetic rats

Groups (n=6)	Percentage cerebral Infarction
Normal	0
Sham control	6.11± 0.47
I/R	59.06± 0.74*
Vehicle treated	59.32± 0.60
**Naringenin** (50 mg/kg, i.p.)	36.91± 0.98*
**Naringenin** (100 mg/kg, i.p.)	29.61± 1.02*
**Naringenin** (150 mg/kg, i.p.)	18.62± 0.61*
**Naringenin** (200 mg/kg, i.p.)	10.87± 0.87*

Data represent the mean±SEM, the asterisk indicates P≤0.05, statistically significant difference from control group, I/R indicates ischemia and reperfusion. No of animals used in each group =6

Table 2: Effect of naringenin on cerebral oxidative stress bio markers in diabetic rats

Assesments	Normal	Sham control	I/R	Vehicle Treated	Naringenin (200 mg/kg, *i.p.*) Treated
**MDA (nMol/gm of wet tissue)**	149.6±0.49	200.42±1.96	801.46±2.26*	802.83±2.92	189.36±5.46*
**SOD (Units/mg of protein)**	11.76±0.32	10.68±0.45	5.58±0.50*	5.09± 0.63	10.19±0.76*
**CAT (Units/mg of protein)**	124.65±1.02	110.62±4.65	36.53±3.46*	35.28± 1.68	112.79±3.06*

Data represent the mean±SEM, the asterisk indicates P≤0.05, statistically significant difference from control group, I/R indicates ischemia and reperfusion, MDA indicates Malondialdehyde, SOD indicates Superoxide dismutase, CAT indicates catalase. No of animals used in each group =6

Table 3: Effect of Naringenin on cerebral inflammatory markers in diabetic rats

Assesments	Normal	Sham control	I/R	Vehicle Treated	Naringenin (200 mg/kg, *i.p.*) Treated
**MPO (Units/gm of wet tissue)**	3.603± 0.205	5.632± 0.425	96.94± 0.748*	97.02± 0.910	4.96± 1.910*
**TNF-α (ng/mg of tissue)**	0.206± 0.006	0.286± 0.081	0.407± 0.012*	0.410± 0.036	0.093± 0.080*
**IL-6 (ng/mg of tissue)**	0.336± 0.042	0.342± 0.051	0.809± 0.056*	0.810± 0.066	0.297± 0.028*
**IL-10 (ng/mg of tissue)**	3.080± 0.064	2.672± 0.063	0.895± 0.068*	0.870± 0.043	1.976± 0.23*

Data represent the mean±SEM, the asterisk indicates $P \leq 0.05$, statistically significant difference from control group, I/R indicates ischemia and reperfusion, MPO indicates Myeloperoxidase, TNF-α indicates Tumor necrosis factor-alpha, IL-6 indicates Interleukin-6, IL-10 indicates Interleukin-10. No of animals used in each group =6
