# Supplementary material for: Fake paper identification in the pool of withdrawn and rejected manuscripts submitted to Naunyn–Schmiedeberg’s Archives of Pharmacology
Source: Naunyn Schmiedebergs Arch Pharmacol. 2023 Oct 5;397(4):2171–81. doi: 10.1007/s00210-023-02741-w (PMC10933159; doi:10.1007/s00210-023-02741-w)
Supplement: Supplementary file 9 — Supplementary file9 (PDF 1152 KB) [file 210_2023_2741_MOESM9_ESM.pdf]

Figure S9

Color coding:

Yellow highlighted text	The text is identical in the NSAP version and the published version of this paper.
Red highlighted text	There are differences in the text between the NSAP version and the published version of this paper (different content or different wording).
Yellow bordered figure	This figure is identical in both versions of this paper.
Red bordered figure	This figure is different in both versions of this paper.

Naunyn-Schmiedeberg's Archives of Pharmacology

The protective effect of vitexin compound B-1 against rat cerebral I/R injury is related to modulation of miR-92b/NOX4 pathway

--Manuscript Draft--

**Manuscript Number:**	NSAP-D-17-00097R1
**Full Title:**	The protective effect of vitexin compound B-1 against rat cerebral I/R injury is related to modulation of miR-92b/NOX4 pathway
**Article Type:**	Original Article
**Corresponding Author:**	Jun Peng<br>Central South University<br>Changsha, Hunan CHINA
**Corresponding Author Secondary Information:**	
**Corresponding Author's Institution:**	Central South University
**Corresponding Author's Secondary Institution:**	
**First Author:**	Bin Tan
**First Author Secondary Information:**	
**Order of Authors:**	Bin Tan
	Zhong-Bao Yang
	Meng-Xuan Tang
	Jing Tian
	Jing-Jie Peng
	Xiao-Jie Zhang
	Jie Yang
	Xiu-Ju Luo
	Jun Peng
**Order of Authors Secondary Information:**	
**Funding Information:**	National Natural Science Foundation of China (81603107) — Dr Zhong-Bao Yang
	National Natural Science Foundation of China (81573430) — Dr Xiu-Ju Luo
	National Natural Science Foundation of China (81373409) — Professor Jun Peng
	Natural Science Foundation Hunan Province in China (2015JJ2156) — Dr Xiu-Ju Luo
**Abstract:**	Recent studies uncovered that vitexin compound B-1 (VB-1), a novel member of the vitexins family, is able to protect nerve cells against hypoxia/reoxygenation (H/R)-induced oxidative injury through suppressing NADPH oxidase (NOX) expression. The aims of this study are to investigate whether VB-1 is able to protect rat brain against ischemia/reperfusion (I/R) injury and whether its effect on NOX4 expression is related to modulation of certain miRNAs expression. SD rats were subjected to 2h of cerebral ischemia following by 24h of reperfusion to establish an I/R injury model, which showed an increase in neurological deficit score and infarct volume concomitant with upregulation of NOX4 expression, increase in NOX activity and downregulation of miR-92b. Administration of VB-1 reduced I/R-induced cerebral injury accompanied by a

Powered by Editorial Manager® and ProduXion Manager® from Aries Systems Corporation

	reverse in NOX4 and miR-92b expression as well as NOX activity. Similar results were achieved in a nerve cell hypoxia/reoxygenation (H/R) injury model. Next, we evaluated the association of miR-92b with NOX4 by its mimics in the H/R model. H/R treatment increased the nerve cells apoptosis concomitant with upregulation of NOX4 and NOX activity while downregulation of miR-92b, mimicking our in vivo findings. All these effects were reversed in the presence of miR-92b mimics, confirming a function of miR-92b in suppressing NOX4 expression. Based on these observations, we conclude that the protective effect of VB-1 against rat cerebral I/R injury is related to modulation of miR-92b/NOX4 pathway.
**Response to Reviewers:**	see attachment

Powered by Editorial Manager® and ProduXion Manager® from Aries Systems Corporation

Ref.: Ms. No. NSAP-D-17-00097

Reviewers' comments:

Reviewer #1: The authors investigated the molecular mechanism underlying the protective effect of vitexin compound B-1 on nerve cells against hypoxia/reoxygenation-induced injury. In an animal model as well as in NG108-15 cells, VB-1 was demonstrated to protect cells against H/R injury through up-regulation of miR-92b and ensuing down-regulation of NOX4. They concluded that VB-1 has a potential value for the treatment of ischemic stroke.

All data are clearly presented and the manuscript is well written. There are, however some issues to be addressed:

1) The reason why authors only focused on NOX4 is not clear. Is it just because targeting miRNAs were not identified for NOX2 by bioinformatics analysis? Authors should at least show the expression pattern of NOX2 and NOX1 under H/R injury.

Response: Thank you very much for raising this important issue. Yes, we focused on NOX4-relevant miRNAs in this study is because targeting miRNAs were not identified for NOX2 by bioinformatics analysis, although its expression was up-regulated under the condition of H/R in our previous study (Yang ZB et al. Naunyn Schmiedebergs Arch Pharmacol. 2014; 387: 861-871). As for NOX1, there was no significant change under such condition. We have clarified this issue in the revised "Discussion" (Please see Page 15, Line 11-14).

2) Authors should show the entire blot for NOX4 protein (Figs. 2B & 4C).

Response: Following your suggestion, the entire blot for NOX4 protein (Figs. 2B & 4C) was showed below.

Fig.2B. The entire blot for NOX4 protein

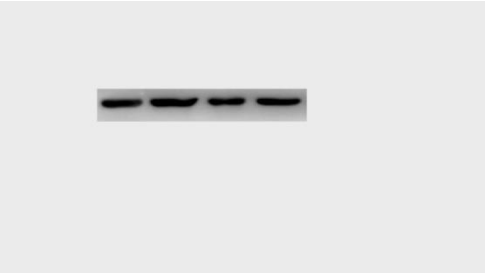

Fig.4C. The entire blot for NOX4 protein

As shown in Fig.2B, the NOX4 antibody is very specific, and there is only one band in the blot for NOX4. In recent 3 years, we have performed lots of WB for NOX4, and the NOX4 antibody worked very well (sc-30141, Santa Cruz, CA, USA) (see below). Thus, we are very confident in the WB for NOX4. To save the antibody, some of my students narrowed the blot (like Fig. 4C) as possible as they can. Recently, my colleagues told me that some reviewers often asked for the entire blot for some proteins. It is the first time for me to be asked for the entire blot for WB. I will require all the students in my lab to strictly follow the standard protocol for all Western blots.

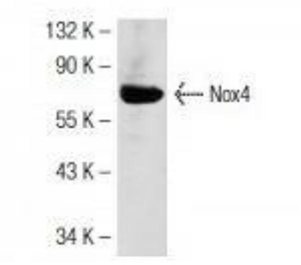

The WB image is from datasheet offered by Santa Cruz Biotechnology

3) NG108-15 is a mouse neuroblastoma x rat glioma hybrid. Which cell lineage NOX4 mRNA and protein were derived from (Fig. 4)? Why did the authors use the hybrid?

Response: Thank you very much for raising this important issue. The primers in table 1 were designed for rat NOX4 mRNA. However, it was very likely the primers for rat NOX4 mRNA also worked for mouse NOX4 mRNA because the forward primer for NOX4 was exactly same between rat and mouse while there was only a different base pair in the reverse primer between rat (5'-TGGGTGGTTTCCTAATGCTGA-3') and mouse (5'-TGGGTGGTTTTCTAATGCTGA-3'). The product size of PCR for rat and mouse was the same. In addition, the NOX4 antibody also worked for both rat and mouse NOX4 protein according to information provided by the seller. Thus, the detection for NOX4 mRNA and protein might be derived from both cell lineages (rat glioma and mouse neuroblastoma). To address this issue, we have added "rat" to table 1 and revised the "Discussion" accordingly (Please see Page 16, Line 13-22).

The best choice for cell experiments is to use primary nerve cells. However, it is difficult for us to do this. As a backup, we chose the cell line to perform the cell experiments. There are three available cell lines in my lab. They are PC12, SH-SY5Y and NG108-15 cell lines. PC 12 derives from the rat pheochromocytoma (not the real nerve cells) while SH-SY5Y derives from human neuroblastoma (not match with the species in animal experiments). Although NG108-15 is a hybrid, it actually worked like a pure rat nerve cells since the PCR primers and antibody for NOX4 worked for both rat and mouse.

Reviewer #2: The authors have presented the neuroprotective effect of VB-1 (vitexin compound B-1) in the rat MCAO ischemia/reperfusion (I/R) model and argued the molecular mechanism of neuroprotection by VB-1 is on NOX-4 inhibition through the regulation of miR92b expressions. The authors have previously reported the protective effect of VB-1 in vitro on PC12 cells from the hypoxia stress in this journal (2014, 387:861-871, DOI 10.1007/s00210-014-1006-0). They conducted in vivo study with rats to reveal that the administration of VB-1 can dramatically decrease the infarct volume of the brain following the MCA occlusion. This is a plausible explanation that VB-1 maintains the level of miR92b expression level even after the I/R stress resulting in the concomitant NOX-4 expressions.

From the pharmacological standpoint, the argument that VB-1 protects both the brain in vivo and neural cells (NG108-15) in vitro extends far beyond the conception of the mechanism of VB-1 action since VB-1 may not reach directly to the brain neuron as the blood-brain barrier (BBB) may not allow VB-1 getting through it. Although the authors insist that the phenotypic reactions in

the rat brain and the cultured cells are similar in miR92b and NOX4 expressions, it is difficult to extend the argument that VB-1 works on these two different materials in the same molecular mechanism. The authors should argue these two lines of results (in vivo and in vitro) independently to clarify the point of interest in this manuscript.

Response: We fully understand your concerns on our results in vivo. To address your concern, firstly, we have replaced the title "Vitexin compound B-1 protects rat brain from ischemia/reperfusion injury through modulation of miR-92b/NOX4 pathway" with "The protective effect of vitexin compound B-1 against rat cerebral I/R injury is related to modulation of miR-92b/NOX4 pathway". Now, we realize that the previous title is not appropriate because the mechanisms responsible for the beneficial effects of VB-1 on cerebral I/R or nerve cell H/R injury are multiple and complicated, particularly in vivo. The miR-92b/NOX4 pathway might be just an involved one in both vivo and in vitro. Secondly, we have added a passage in the "Discussion" section to interpret the likely different actions of VB-1 on the miR-92b/NOX4 pathway between in vivo and in vitro studies (Please see Page 17, Line 17-22).

The following comments are for further improvements of this manuscript.

1. *The authors use the term "arbitrary unit" in the vertical axis title (Figs 2, 3, and 4). This is not appropriate term to use since the values have normalized by the value with the same measurement units.*

Response: We totally agree with your opinion. We have replaced the term "arbitrary unit" with " normalized to β-actin" or " normalized to U6".

2. The inhibitory effect of VB-1 on NADPH oxidase activities following an I/R cycle is too subtle to conclude the neuroprotective effect of VB-1 is through the regulation of NOX activities.

Response: We fully understand your concern on the inhibitory effect of VB-1 on NADPH oxidase activities. It looks so subtle might be related to the unit we used (μmol NADPH/min/mg). To address your concern, we have replaced the unit (μmol NADPH/min/mg) with "μmol NADPH/min/g". In addition, we have replaced the title "Vitexin compound B-1 protects rat brain from ischemia/reperfusion injury through modulation of miR-92b/NOX4 pathway" with "The protective effect of vitexin compound B-1 against rat cerebral I/R injury is related to modulation of miR-92b/NOX4 pathway" and revised the relevant contents in the article accordingly (Please see Page 2, Line 17-19; Page 18, Line 1-3). For example, we have replaced the conclusion "VB-1 can protect the rat brain against I/R injury through modulation of miR-92b/NOX4 pathway" with "The protective effect of VB-1 against rat cerebral I/R injury is related to modulation of miR-92b/NOX4 pathway".

3. Although an I/R cycle inhibits the miR-92b level dramatically in Figure 3A, the level of miR-92b following an I/R cycle in the same figure B is not compatible with A. Additional comment should be noted the scale on the vertical axis.

Response: Thank you very much for your carefulness and we are sorry for our carelessness. There are 2 errors in Figure 3. Firstly, there were mistakes in the scale on the vertical axis in Fig.3A and Fig. 3B. Since the original scale in the figure was not clear, we manually replaced it with a favor one, but we made a mistake. We have already corrected it. Secondly, the sample size for Fig.3A

should be 3 but not 7. We have already corrected it.

As for the incompatible miR-92b levels in the I/R group between Figure 3A and Figure 3B, we present our explanations as follows: Firstly, Figure 3A and Figure 3B were two independent experiments, there were systematic errors between the two experiments. Secondly, the sample size in Figure 3A was 3 while it was 7 in Figure 3B. The big difference in sample size amplified the systematic errors between the two independent experiments.

4. In the materials and methods section, it was read that the mimics were transfected into cells. Figure 4A has a bar indicating the expression level of miR92b with the transfection of the mimic at the same height with the control. The authors should clarify this.

Response: Thank you very for raising this import issue. It was true that the expression level of miR-92b in the "H/R+mimics" group was close to that in the control group in Figure 4A. To explain this phenomenon, we checked the experiments for transfection efficiency of miR-92b mimics. As shown in the Supplemental Figure 1, compared with control, the expression levels of miR-92b in cells transfected with mimics at 5, 10 or 20 nM for 12h were increased by 2-6 folds under normoxia. However, the situation completely changed after H/R treatment. One possible explanation was that the transfection condition under H/R was not optimized, resulting in low transfection efficiency. Another possible explanation might be related to the competing endogenous RNA (ceRNA), which shares miRNAs responses elements and acts as modulator of miRNA by influencing the available level of miRNA (Tay et al. Nature. 2014; 505: 344-352). It was likely that the change in ceRNA level after H/R treatment led to the decrease in the level of miR-92b. Nevertheless, more studies are needed before drawing a firm conclusion. We have clarified this point in the revised "Discussion" (Please see Page 17, Line 5-16).

5. The authors claimed Hoechst assay as an apoptosis detection assay; however, this is not an appropriate term. The microscopic images do not segregate the morphological changes in nucleus from apoptosis to necrosis. The alternative method such as the TUNEL assay should be applied to confirm the results.

Response: Thank you very much for raising this important issue. It is true that Hoechst 33258 is not a specific dye for apoptotic cell. Although it is not easy to segregate the morphological changes in nucleus from apoptosis to necrosis by Hoechst staining, we think the cells showing nuclear condensation and brightness could be regarded as apoptotic cells. We have emphasized this in the description for Hoechst assay (Please see Page 9, Line 4-5). To compensate the limitation of Hoechst assay in apoptosis detection, we measured the caspase-3 activity at the same time. Nevertheless, TUNEL assay or Hoechst/Propidium iodide (PI) double staining assay is more reliable in detection of apoptosis. We will consider such methods in our future studies.

6. The supplementary Materials section has a panel as figure S1, this is the identical image with figure 1 in their previous publication (as mentioned above).

Response: Yes, the Figure S1 is the identical image with Figure 1 in our previous publication (Yang ZB, et al. Naunyn Schmiedebergs Arch Pharmacol. 2014, 387: 861-871). Since it is just a chemical structure for VB-1 and neolignan but not a experimental result, we thus present it as a supplementary figure to help authors to follow up. Now we are aware that it is inappropriate to use

a published material although it is just a chemical structure. Therefore, we have replaced the chemical structure image with the citation (Please see Page 3, Line 22; Page 4, Line 1).

Manuscript
Click here to download Manuscript Tan B-MS-R.doc
Click here to view linked References

The protective effect of vitexin compound B-1 against rat cerebral I/R injury is related to modulation of miR-92b/NOX4 pathway

Bin Tan[1, 2], Zhong-Bao Yang[1, 3], Meng-Xuan Tang[4], Jing Tian[1], Jing-Jie Peng[1], Xiao-Jie Zhang[1], Jie Yang[5], Xiu-Ju Luo[1, 4†], Jun Peng[1†]

[1]*Department of Pharmacology, Xiangya School of Pharmaceutical Sciences, Central South University, Changsha 410078, China.*

[2]*Department of Pharmacology, Xiang-Nan University, Chenzhou 423000, China.*

[3]*Department of Pharmacy of Affiliated Changsha Hospital of HuNan Normal University, Changsha 410006, China*

[4]*Department of Laboratory Medicine, Xiangya School of Medicine, Central South University, Changsha 410013，China.*

[5]*Department of Neurology, Xiangya Hospital, Central South University, Changsha 410008, China.*

[†] **Correspondence and request for reprints to**:

Dr. Associate professor Xiu-Ju Luo, PhD

Department of Laboratory Medicine, Xiangya School of Medicine, Central South University, Changsha 410013，China.

Email: xjluo22@csu.edu.cn

Or

Professor Jun Peng, MD, PhD

Department of Pharmacology, Xiangya school of Pharmaceutical Sciences, Central South University. No.110 Xiang-Ya Road, Changsha, 410078, China.

Tel: 0086-731-82355080；Fax: 0086-731-82355078；

E-mail: Junpeng@csu.edu.cn

Abstract:

Recent studies uncovered that vitexin compound B-1 (VB-1), a novel member of the vitexins family, is able to protect nerve cells against hypoxia/reoxygenation (H/R)-induced oxidative injury through suppressing NADPH oxidase (NOX) expression. The aims of this study are to investigate whether VB-1 is able to protect rat brain against ischemia/reperfusion (I/R) injury and whether its effect on NOX4 expression is related to modulation of certain miRNAs expression. SD rats were subjected to 2h of cerebral ischemia following by 24h of reperfusion to establish an I/R injury model, which showed an increase in neurological deficit score and infarct volume concomitant with upregulation of NOX4 expression, increase in NOX activity and downregulation of miR-92b. Administration of VB-1 reduced I/R-induced cerebral injury accompanied by a reverse in NOX4 and miR-92b expression as well as NOX activity. Similar results were achieved in a nerve cell hypoxia/reoxygenation (H/R) injury model. Next, we evaluated the association of miR-92b with NOX4 by its mimics in the H/R model. H/R treatment increased the nerve cells apoptosis concomitant with upregulation of NOX4 and NOX activity while downregulation of miR-92b, mimicking our in vivo findings. All these effects were reversed in the presence of miR-92b mimics, confirming a function of miR-92b in suppressing NOX4 expression. Based on these observations, we conclude that the protective effect of VB-1 against rat cerebral I/R injury is related to modulation of miR-92b/NOX4 pathway.

Key words: hypoxia/reoxygenation; miR-92b; NADPH oxidase; vitexin compound B-1; oxidative injury

Introduction

Ischemic stroke arises from critical blood flow reduction due to occlusion of brain vessels, leading to substantial disability or mortality. Rapid restoration of blood supply (refer as reperfusion) by means of thrombolysis or mechanical recanalization (either spontaneous or therapeutic) is the first and essential step to save the injured cells. However, in many cases, restoration of blood flow can cause 'reperfusion injury', which further exacerbates the ischemic injury(Li et al. 2017). There is plenty of evidence showing that ischemia/reperfusion (I/R) injury in the brain involves oxidative stress, inflammation, and excitotoxicity, etc (Chamorro et al. 2016). Of the multiple mechanisms, oxidative stress-induced cellular damage (necrosis and apoptosis) following I/R contributes greatly to brain injury(Rodrigo et al. 2013; Zhang et al. 2015). Therefore, drugs with good antioxidative activities may have potential therapeutic value for ischemic stroke.

Vitexins are a mixture of lignan compounds found in Vitex Negundo seed(Zhou et al. 2009), *Phyllostachys nigra* bamboo leaves (Lee et al. 2010), chaste tree (Hajdu et al. 2007), *Pennisetum* millet, Hawthorn and Passion flower(Ma et al. 2010; Zucolotto et al. 2012). Vitexins possess various pharmacological effects, such as antioxidative activity (Borghi et al. 2013; Lee et al. 2015), anti-inflammatory activity(Dong et al. 2013; Wang et al. 2017), antinociceptive activity(Borghi et al. 2013; Zhu et al. 2016), anticancer effect(Yang et al. 2013), and antidepressant-like effect(Can et al. 2013). In Zhou et al's study, a single vitexin named vitexin compound B-1 (VB-1), or 6-hydroxy- 4- (4-hydroxy-3-methoxyphenyl)-3-hydroxymethyl-7-methoxy-3,4- dihydro -2- naphthaldehyde, was purified from the seeds of Chinese herb Vitex Negundo (Yang et al. 2014a), which shows different general structure

and belongs to the class of neolignan (Zhou et al. 2009). Recently, we have demonstrated that VB-1 protects the PC12 cells against hypoxia/reoxygenation(H/R) injury through suppression of NADPH oxidase (NOX) expression and subsequent reduction of reactive oxygen species (ROS) production(Yang et al. 2014a). However, the mechanisms for the functions of VB-1 in regulation of NOX expression need to be determined.

miRNAs are small (~21 nt) non-coding RNAs that participate in the post-transcriptional regulation of target mRNAs, usually resulting in translational repression or target degradation and gene silencing(Rupaimoole and Slack 2017). Accumulating evidence suggests that many drugs, including Chinese herbal medicine, exert their actions through regulation of miRNA expression (Rupaimoole and Slack 2017; Yang et al. 2015). Thus, it is likely that VB-1 suppresses NOX expression in H/R-treated PC12 cells by up-regulating certain miRNAs. After conducting the bioinformatic analysis, we predicted a number of potential miRNAs, including miR-92a, miR-92b, miR-25 and miR-9, which might target NOX4 (Figure S2). To verify the relationship between NOX4 and the selected miRNAs, we examined their levels in brain tissue of rats following I/R treatment. Among the screened miRNAs, only miRNA-92b expression was down-regulated. Based on these results as well as the previous report, we hypothesize that VB-1 may protect rat brain from I/R injury through targeting miR-92b/NOX4 pathway.

In the present study, we aimed to explore whether VB-1 was able to ameliorate I/R injury by enhancing miR-92b expression in rat brain subjected to middle cerebral artery occlusion (MCAO). To corroborate the findings in vivo study, we established a nerve cell model of hypoxia-reoxygenation (H/R) injury in vitro to mimic the condition of I/R injury in

vivo. Our data suggest that the neuroprotective effect of VB-1 against oxidative injury is involved in the regulation of miR-92b/NOX4 pathway.

Materials and methods

Experiments in animals

Male Sprague-Dawley rats weighing 200-250g were provided by the Laboratory Animal Center, Xiangya School of Medicine, Central South University, China. The rats were starved for 24h before the experiments, with free access to tap water. The study was carried out following the Guide for the Care and Use of Laboratory Animals, published by the National Institutes of Health (NIH Publication, 8th edition, 2011), the ARRIVE guidelines (Animal Research: Reporting In Vivo Experiments) and experiments were approved by the Central South University Veterinary Medicine Animal Care and Use Committee.

To establish the I/R injury model, rats were subjected to MCAO as we described previously (Fu et al. 2014; Yang et al. 2014b). Briefly, under anesthetic condition (sodium pentobarbital,60 mg/kg, i.p.), the left common carotid artery (CCA) was exposed and clipped with artery clamp. The external carotid artery (ECA) was isolated and ligatured. A nylon suture with a blunted tip (0.40 mm diameter) was introduced through a tiny incision in ECA and then into internal carotid artery (ICA). The middle cerebral artery (MCA) was occluded by nylon suture at the position of 18-20 mm distal from a carotid bifurcation. The nylon suture was left there for 2h and then withdrawn for 24h reperfusion. The rats were returned to cages with free access to water and food after incision was closed. Rats from the sham group underwent the same procedure except that the nylon suture was inserted only 7 mm above the carotid bifurcation.

To investigate the effect of VB-1 on I/R injury, the rats were randomly allocated to 7 groups (n=14 per group): the control group, rats received no surgery; the sham group, rats underwent surgical procedures with no ischemic insult; the I/R group, rats were subjected to 2h of ischemia followed by 24h of reperfusion; the VB-1 low (L), middle (M) or high (H) dose plus I/R group, rats treated with VB-1 (99.1% purity, provided by Department of Medicinal Chemistry, Central South University) at low, middle or high dose (5, 10, or 20 mg/kg, i.g., respectively) 30 min before ischemia; the vehicle group, rats were treated with equal volume of VB-1 vehicle (carboxymethyl cellulose sodium, i.g.) 30 min before ischemia. At the end of reperfusion, neurological deficit score was assessed first, and then the brain tissues of 7 rats from each group were saved for infarct volume measurement, whereas the brain tissues (dissected from ischemic boundary area) of the remaining 7 rats from each group were collected for molecular studies (the expression of miRNA, mRNA or protein; activity of caspase-3). Four rats in total were excluded from the study because 2 of them died before the planned end point while 2 of them did not give rise to infarcts due to surgical failure.

Assessment of neurological deficit score and infarct volume

Twenty-four hours after reperfusion, neuroscore assessment was performed by an investigator blinded to the experimental groups according to a five-point neurological deficit score (0=no deficit, 1=failed to extent the left forepaw, 2=decreased grip strength of left forepaw, 3=circling to left by pulling the tail, 4=spontaneous circling).

The absence or presence of infarction was determined by 2, 3, 5-triphenyltetrazolium chloride (TTC) staining. After neurological function assessment, rats' brains were rapidly

removed and were cut into coronal sections (~3 mm thickness) with the aid of a brain matrix. Sections were immersed in 2% TTC for 10 minutes at 37°C, followed by incubation with 4% paraformaldehyde overnight, and then analyzed with the imaging software (Image J, NIH, USA). The infarct volume (in mm^3) of each section was equal to infarct area (in mm^2) multiply by the section thickness (3 mm). The total infarct volume of each brain was then calculated by summing up the infarct volumes of all sections. To eliminate the effect of edema on the accuracy of infarct volume assay, the final infarct volume was corrected by following equation: corrected infarct volume = total infarct volume × (right hemisphere volume/left hemisphere volume). Right hemisphere refers to no-ischemic hemisphere of brain while left hemisphere refers to ischemic contralateral side (see definition in Figure 1B).

Experiments in cell cultures

NG108-15 neural cells (1×10^4 cells/cm^2) were maintained in DMEM, supplemented with 10% fetal bovine serum, penicillin (100 IU/ml), streptomycin (100 mg/ml), and L-glutamine (2 mM), and cultured in a humidified incubator with 95% air/5% CO2 for 2 days. To establish the H/R injury model, NG108-15 cells were incubated in serum free DMEM for 24 h and then subjected to 5h-hypoxia (O2/N2/CO2, 1/94/5) in the pre-conditioned hypoxic medium followed by 20h-reoxygenation, where the hypoxic medium was switched to the fresh medium upon for reoxygenation.

To further confirm the effect of VB-1 on miR-92b expression, NG108-15 cells were randomly divided into 5 groups (6 individual experiments per group): the control group, without treatment; the H/R group, cells were incubated under hypoxic condition for 5h

followed by 20h of reoxygenation; the VB-1 at low dose (L), middle dose (M) or high dose (H) plus H/R group, VB-1 (10^{-8}M, 10^{-7} M or 10^{-6} M) was added to culture medium before the cells were subjected to H/R; the vehicle plus H/R group, equal volume of DMSO (0.5%, final concentration) was added to culture medium before the cells were subjected to H/R. At the end of experiments, cells were collected for measurement of miR-92b expression.

To verify the role of miR-92b in regulation of NOX4 expression, miR-92b mimics (Ribobio) were transfected into NG108-15 with Hiperfect transfection reagent (Qiagen). The procedures for transfection were performed according to the manufacturer's instructions. Cells were divided into four groups: the control group, without any treatment; the H/R group, cells were subjected to 5h of hypoxia followed by 20h of reoxygenation; the miR-92b mimics group, cells were transfected with miR-92b mimics before subjecting to H/R treatment; the mimics (-) control group, cells were transfected with mimics negative control before subjecting to H/R treatment. In the present study, the mimics negative control is based on Caenorhabditis elegans miRNAs not found in humans, rats or mice. Before the above-mentioned experiments, the miRNA mimics used in the present study was confirmed to work as expected (Figure S1). At the end of experiments, cells were collected for Hoechst staining or measurements of miR-92b and NOX4 expressions, NOX and caspase-3 activities.

Measurement of cellular apoptosis

H/R-induced NG108-15 cell apoptosis was evaluated by Hoechst staining. The experiment was performed according to the manufacturer's instruction (Beyotime, Shanghai，China). Briefly, the cells were fixed in 4% paraformaldehyde for 15 min, washed with PBS, and then

incubated with 1g/ml of Hoechst 33258 (a bisbenzimide cell-permeant dye showing bright blue fluorescence after binding to DNA) at room temperature for 5 min. After washing twice with PBS, the stained cells were imaged under a fluorescent microscope (excitation, 350 nm; emission, 460 nm). The cells showing nuclear condensation and brightness were regarded as apoptotic cells. Twenty random high - power fields from each sample were chosen and blindly analyzed. The number of apoptotic cells was presented as percentage of the total cells.

Measurement of NOX and caspase-3 activities

The assays for NOX and caspase-3 activities were conducted following the manufacturer's instructions (GENMED or Beyotime, Shanghai, China). The NOX activity was determined by measuring the NADPH-dependent superoxide dismutase (SOD)-inhibitable cytochrome c reduction. Briefly, the reaction buffer (900 μl) containing the oxidized cytochrome C and the NOX substrate (NADPH) in a quartz cuvette was incubated at 30℃ for 3 min, and then an aliquot (100 μl) of the supernatant from NG108-15 lysates was added to the reaction mixture and incubated at 30℃ for 15 min. The change of absorbance at 550 nm was monitored by a spectrophotometer. A buffer blank was measured in each assay, and SOD-inhibitable cytochrome c reduction in buffer blank was subtracted from each sample. The NOX activity was calculated as SOD-inhibitable cytochrome c reduction and expressed as O_2^- in nmol/mg/min. For caspase-3 activity assay, mixture of 10 μl of NG108-15 cell lysate with 90 μl of reaction solution containing caspase-3 substrate (Ac-DEVD-pNA) was incubated at 37 °C for 60 min, and then the absorbance at 405 nm was observed. The enzyme activity was expressed as U/g protein and 1 U of enzyme was presented as the amount of enzyme required

to cleave 1.0 nmol Ac-DEVD-pNA per h at 37 °C.

Measurement of miRNAs and NOX4 mRNA expression

Real-time PCR was conducted to quantify the NOX4 mRNA and miRNAs expression in brain tissue (from ischemic boundary area) or NG108-15 cells. Total RNA was isolated by using TRIzol reagent (TakaRa, Dalian, China). 500 ng of RNA was reversely transcribed into cDNA with a transcription Kit following the manufacturer's instructions (TaKaRa, Dalian, China). Quantitative PCR was conducted by using SYBR Premix Ex Taq (TaKaRa Dalian, China) in an ABI 7300 Real-time PCR system. Briefly, a 10-μl reaction mixture containing 2 μl of cDNA template, 5 μL of SYBR Master mix, 0.20 μl of ROX, 2.4 μl of H_2O, and 0.20 μl of each primer was amplified by the following procedures: denaturing at 95°C for 10 min and 40 cycles of the amplification step (denaturation at 95°C for 15 seconds, annealing and extension at 60°C for 31 seconds). All amplification reactions were performed in triplicate and the averages of the threshold cycles were used to interpolate curves using the 7300 System SDS Software. Results were expressed as the ratio of NOX4 mRNA to β-actin mRNA. The primers for NOX4 and β-actin are listed in Table 1. Bulge-Loop miRNA Primers provided by RiboBio Co., Ltd (Guangzhou, China) were used for detection of miRNAs (miR-92a, miR-92b, miR-25, miR-9), and U6 was used as an internal control.

Western blot analysis for NOX4 protein levels

Brain tissues or NG108-15 cells were homogenized in ice-cold lysis buffer (250 mM Tris-HCl, pH 6.8, 20% vol/vol glycerol, 4% SDS and Roche EDTA-free protease inhibitor cocktail),

sonicated for 1 minute, and then centrifuged for 10 minutes at 15000g. Western blot was performed according to standard techniques. Briefly, samples containing 20 - 60 μg of protein were subjected to SDS-PAGE (10% gel), and the proteins were transferred to polyvinylidene fluoride membranes. Blots were incubated with rabbit anti-NOX4 (Santa Cruz, CA, USA) followed by horseradish peroxidase-conjugated secondary antibodies. The signals of bands were determined using an ECL kit (GE Healthcare) through Molecular Imager ChemiDocXRS System (Bio-Rad) and analyzed with Image J 1.43 (National Institutes of Health). Antibody against β-actin (Beyotime, Nanjing, China) was chosen as a loading control.

Statistical analysis

SPSS software (Version 19.0, SPSS, Inc., Chicago, IL, USA) was used for statistical analysis. Data were expressed as mean ± S.E.M. Differences in values among the multiple groups were determined by the analysis of variance with Bonferroni's multiple comparison tests. χ^2 test was used to compare the data of frequency distribution between groups. Differences were considered significant when $P < 0.05$.

Results

VB-1 treatment improves neurological function and reduces infarct volume in MCAO rats

The neurological function in MCAO rat model is commonly evaluated by a 5-point rating scale of neurological deficit score. Compared to the control group, there was no significant

change in neurological function in the sham group, while there was an obvious increase in neurological deficit score in the MCAO rats. VB-1 treatment dramatically improved neurological function in the MCAO rats in a dose-dependent manner, while the vehicle of VB-1 did not display such effect (Figure 1A). Consistent with the changes in neurological deficit score, there were no cerebral infarcts in rats in both control and sham groups, but the infarct volumes in the MCAO rats were markedly increased compare to rats in the sham group. VB-1 treatment significantly decreased the infarct volumes in the MCAO rats, while the vehicle of VB-1 had no such effect (Figure 1 B and C).

VB-1 treatment suppresses NOX4 expression in MCAO rats

As shown in Figure 2 A and B, compared to the control rats, the mRNA and protein expressions of NOX4 in brain tissues were not significantly changed in rats from the sham group, while they were dramatically elevated in rats subjected to I/R treatment. The upregulation of NOX4 in MCAO rats was remarkably attenuated by VB-1 at both middle and high dosages (10 or 20 mg/kg). In agreement with the results of NOX4 expression, there was a significant increase in cerebral NOX activity in MCAO rats compared with that in the sham group, which was decreased in the presence of VB-1 (Figure 2C.). The vehicle of VB-1 had no such effect on NOX expression and activity.

VB-1 treatment reverses miR-92b expression in MCAO rats and H/R-treated NG108-15 cells

Based on bioinformatic analysis by Target Scan 7.1 software and miRbase 21, at least 4

miRNAs (miR-92a, miR-92b, miR-9 and miR-25) were predicted to target NOX4. Thus, these 4 miRNAs levels in rat brain tissue were examined. Among them, only miR-92b level was decreased in the MCAO rats, while there were no significant changes in miR-92a, miR-9 and miR-25 expressions (Figure 3A). We therefore focused on miR-92b in our following experiments. As expected, VB-1 treatment significantly reversed the miR-92 expression in the MCAO rats at both middle and high dosages (Figure 3B). Similarly, miR-92b expression was down-regulated in H/R-treated NG108-15 cells compared to that in the control cells, which was reversed in the presence of VB-1 at both middle and high dosages (Figure 3C). The vehicle of VB-1 did not show effect on miR-92b expression in rat cerebra or in the cultured nerve cells.

miR-92b mimics block the upregulation of NOX-4 in H/R-treated NG108-15 cells

In cultured NG108-15 cells, miR-92b expression was down-regulated following H/R treatment, which was consistent with the results in the I/R-treated animals, and the down-regulation of miR-92b was reversed by the miR-92b mimics (Figure 4A). In agreement with the results of the animal experiments, both mRNA and protein expressions of NOX4 were significantly up-regulated in nerve cells after H/R treatment concomitant with the elevated NADPH oxidases activity, and these phenomena were reversed in the presence of miR-92b mimics (Figure 4, B-D).

miR-92b mimics decrease the apoptotic ratio in H/R-treated NG108-15 cells

To evaluate H/R-induced NG108-15 cell injury, cellular apoptosis (Hoechst staining and

caspase3 activity) was assessed. The results of Hoechst staining showed that the percentage of apoptotic cells in the H/R group was dramatically elevated compared with that in the control group, and the increased apoptosis was attenuated by treating the cells with miR-92b mimics (Figures 5A and 5B). Consistent with the results of Hoechst staining, H/R significantly increased caspase 3 activity, this increase was reversed in the presence of miR-92b mimics (Figure 5C). The negative control of mimics had no such effect.

Discussion

In this study, by using a rat model of cerebral I/R injury or a nerve cell model of H/R injury, we evaluated the protective effect of VB-1 on ischemic brain or hypoxic NG108-15 cells and explored whether these effects were involved in the regulation of miR-92b/NOX4 pathway. Our results clearly showed that I/R or H/R treatment obviously caused neurological dysfunction, brain tissue or cell injury (such as cellular necrosis and apoptosis) concomitant with a decrease in miR-92b expression and an upregulation in NOX4; these effects were dramatically attenuated by VB-1 or miR-92b mimics. To the best of our knowledge, this is the first study to demonstrate that the protective effect of VB-1 on ischemic rat brain is through targeting the miR-92b/NOX-4 pathway.

VB-1, isolated from vitexins, belongs to the class of neolignan and represents the most abundant lignan, accounting for 38% of the total isolated vitexins (Zhou et al. 2009). To date, many studies have revealed dual functions of vitexins (protective or toxic effect) depending on cell type. They can induce cell autophagy and apoptosis in cancer cells while prevent cell apoptosis in cardiomyocytes or nerve cells(Che et al. 2016; Dong et al. 2013; He et al. 2016;

Yang et al. 2014a; Zhang et al. 2017; Zhou et al. 2009). It has been shown that protective effects of vitexins on cardiomyocytes or nerve cells are related to their pharmacological actions of anti-inflammation and anti-oxidation (Che et al. 2016; Rosa et al. 2016). Recently, we have reported that VB-1 is able to protect the PC12 cells from H/R-induced injury through suppressing NOX expression and ROS production. However, it remains unknown whether VB-1 could also exert beneficial effects on nerve cells in vivo. The results from the present study showed that VB-1 significantly attenuated neurological deficit score and infarct volume in I/R-treated rats in a dose-dependent manner, accompanied by a decrease in NOX4 expression and NOX activity, indicating that VB-1 might have potential therapeutic value in reduction of brain injury following I/R through its antioxidative property.

Our recent study has shown that the expressions of NOX2 and NOX4 in H/R-treated PC12 cells were dramatically up-regulated, while there was no significant change in NOX1 expression (mRNA and protein), and VB-1 exerted antioxidative effect through suppressing NOX expression (NOX2 and NOX4) (Yang et al. 2014a). However, it is not known whether the effect of VB-1 on NOX expression is direct or indirect. Accumulating evidence suggests that the action of many drugs, including Chinese herbal medicine, can be mediated through regulation of miRNA expression. As we mentioned before, miRNAs play a key role in the regulation of gene expression. Through the interaction between miRNA and target mRNA, miRNAs can cause mRNA cleavage and/or translational repression and thus reduce the final protein output. It is likely that VB-1 decreases NOX expression through affecting certain miRNAs levels. Based on the bioinformatic analysis, we identified 4 NOX4-relevant miRNAs (miR-92a, miR-92b, miR-25 and miR-9). We found that NOX4 mRNA possesses highly

conserved sites (target regions) for these 4 miRNAs (Figure S2) . However, potential targeting miRNAs were not identified for NOX2 by bioinformatic analysis. Thus, we focused on NOX4-relevant miRNAs in this study.

To evaluate the correlation between the selected miRNAs and NOX4 expression, we measured their levels in brain tissues. The results showed that miR-92b expression was remarkably down-regulated following I/R, while there was no significant change in the remaining miRNAs (miR-92a, miR-25 and miR-9), indicating that up-regulation of NOX4 in I/R-treated rat brain might due to, at least partially, the decrease of miR-92b expression. Based on these results, it is reasonable to predict that the inhibitory effect of VB-1 on NOX4 expression is related to modulating miR-92b expression. We indeed found that VB-1 treatment dramatically increased miR-92b expression in I/R-treated rat brain or in H/R-treated NG108-15 cells concomitant with a reversion in NOX4 expression, confirming the hypothesis we mentioned above. It is noteworthy that NG108-15 is a mouse neuroblastoma × rat glioma hybrid. Although the primers in table 1 were designed for rat NOX4 mRNA, it was very likely the primers for rat NOX4 mRNA also worked for mouse NOX4 mRNA because the forward primer for NOX4 was exactly same between rat and mouse while there was only a different base pair in the reverse primer between rat (5’-TGGGTGGTTT**C**CTAATGCTGA-3’) and mouse (5’-TGGGTGGTTT**T**CTAATGCTGA-3’). The product size of PCR for rat and mouse was the same. In addition, the NOX4 antibody also worked for both rat and mouse NOX4 protein according to information provided by the seller. Thus, the detection for NOX4 mRNA and protein in this study might be derived from both cell lineages (rat glioma and mouse neuroblastoma).

To further verify the role of miR-92b in suppression of NOX4 expression, miR-92b mimics were applied to the nerve cell model of H/R injury. The results showed that miR-92b mimics were able to reverse the upregulation of NOX4 in H/R-treated NG108-15 cells, accompanied by a reduction in NOX activity and cellular apoptosis, supporting that NOX4 is a target gene of miR-92b. To our surprise, the expression level of miR-92b in the "H/R+mimics" group was close to that in the control group in Figure 4A. To explain this phenomenon, we checked the experiments for transfection efficiency of miR-92b mimics. As shown in the Figure S1, compared with control, the expression levels of miR-92b in cells transfected with mimics at 5, 10 or 20 nM for 12h were increased by 2~6 folds under normoxia. However, the situation completely changed after H/R treatment. One possible explanation was that the transfection condition under H/R was not optimized, resulting in low transfection efficiency. Another possible explanation might be related to the competing endogenous RNA (ceRNA), which shares miRNAs responses elements and acts as modulator of miRNA by influencing the available level of miRNA (Tay et al. 2014). It was likely that the change in ceRNA level after H/R treatment led to the decrease in the level of miR-92b. Nevertheless, more studies are needed before drawing a firm conclusion.

It is worth to point out that although VB-1 shows similar effects on miR-92b/NOX4 pathway in both animal and cell experiments, it does not mean that the action of VB-1 on miR-92b/NOX4 pathway in vivo is same as that in vitro. We could not rule out the possibility that VB-1 might act indirectly on miR-92b/NOX4 pathway in vivo since it may not directly reach the brain neuron due to the block of the blood brain barrier. More studies are needed before clarification of this issue.

In summary, the results presented in this study demonstrate for the first time that the protective effect of VB-1 against rat cerebral I/R injury is related to modulation of miR-92b/NOX4 pathway. VB-1 might have potential therapeutic value for patients with ischemic stroke.

Acknowledgements

This work was supported by National Natural Science Foundation of China (No. 81603107 to Zhong-Bao Yang, No. 81573430 to Xiu-Ju Luo, No.81373409 to Jun Peng) and Natural Science Foundation of Hunan Province in China (No.2015JJ2156 to Xiu-Ju Luo).

References

Borghi SM, Carvalho TT, Staurengo-Ferrari L, Hohmann MS, Pinge-Filho P, Casagrande R, Verri WA, Jr. (2013) Vitexin inhibits inflammatory pain in mice by targeting TRPV1, oxidative stress, and cytokines. J Nat Prod 76: 1141-1149.

Can OD, Demir Ozkay U, Ucel UI (2013) Anti-depressant-like effect of vitexin in BALB/c mice and evidence for the involvement of monoaminergic mechanisms. Eur J Pharmacol 699: 250-257.

Chamorro A, Dirnagl U, Urra X, Planas AM (2016) Neuroprotection in acute stroke: targeting excitotoxicity, oxidative and nitrosative stress, and inflammation. Lancet Neurol 15: 869-881.

Che X, Wang X, Zhang J, Peng C, Zhen Y, Shao X, Zhang G, Dong L (2016) Vitexin exerts cardioprotective effect on chronic myocardial ischemia/reperfusion injury in rats via inhibiting myocardial apoptosis and lipid peroxidation. Am J Transl Res 8: 3319-3328.

Dong LY, Li S, Zhen YL, Wang YN, Shao X, Luo ZG (2013) Cardioprotection of vitexin on myocardial ischemia/reperfusion injury in rat via regulating inflammatory cytokines and MAPK pathway. Am J Chin Med 41: 1251-1266.

Fu SH, Zhang HF, Yang ZB, Li TB, Liu B, Lou Z, Ma QL, Luo XJ, Peng J (2014) Alda-1 reduces cerebral ischemia/reperfusion injury in rat through clearance of reactive aldehydes. Naunyn Schmiedebergs Arch Pharmacol 387: 87-94.

Hajdu Z, Hohmann J, Forgo P, Martinek T, Dervarics M, Zupko I, Falkay G, Cossuta D, Mathe I (2007) Diterpenoids and flavonoids from the fruits of Vitex agnus-castus and

antioxidant activity of the fruit extracts and their constituents. Phytother Res 21: 391-394.

He M, Min JW, Kong WL, He XH, Li JX, Peng BW (2016) A review on the pharmacological effects of vitexin and isovitexin. Fitoterapia 115: 74-85.

Lee EB, Kim JH, Cha YS, Kim M, Song SB, Cha DS, Jeon H, Eun JS, Han S, Kim DK (2015) Lifespan Extending and Stress Resistant Properties of Vitexin from Vigna angularis in Caenorhabditis elegans. Biomol Ther (Seoul) 23: 582-589.

Lee HJ, Kim KA, Kang KD, Lee EH, Kim CY, Um BH, Jung SH (2010) The compound isolated from the leaves of Phyllostachys nigra protects oxidative stress-induced retinal ganglion cells death. Food Chem Toxicol 48: 1721-1727.

Li S, Hafeez A, Noorulla F, Geng X, Shao G, Ren C, Lu G, Zhao H, Ding Y, Ji X (2017) Preconditioning in neuroprotection: From hypoxia to ischemia. Prog Neurobiol.

Ma LY, Liu RH, Xu XD, Yu MQ, Zhang Q, Liu HL (2010) The pharmacokinetics of C-glycosyl flavones of Hawthorn leaf flavonoids in rat after single dose oral administration. Phytomedicine 17: 640-645.

Rodrigo R, Fernandez-Gajardo R, Gutierrez R, Matamala JM, Carrasco R, Miranda-Merchak A, Feuerhake W (2013) Oxidative stress and pathophysiology of ischemic stroke: novel therapeutic opportunities. CNS Neurol Disord Drug Targets 12: 698-714.

Rosa SI, Rios-Santos F, Balogun SO, Martins DT (2016) Vitexin reduces neutrophil migration to inflammatory focus by down-regulating pro-inflammatory mediators via inhibition of p38, ERK1/2 and JNK pathway. Phytomedicine 23: 9-17.

Rupaimoole R, Slack FJ (2017) MicroRNA therapeutics: towards a new era for the

management of cancer and other diseases. Nat Rev Drug Discov 16: 203-222.

Tay Y, Rinn J, Pandolfi PP (2014) The multilayered complexity of ceRNA crosstalk and competition. Nature 505: 344-352.

Wang F, Yin J, Ma Y, Jiang H, Li Y (2017) Vitexin alleviates lipopolysaccharideinduced islet cell injury by inhibiting HMGB1 release. Mol Med Rep 15: 1079-1086.

Yang SH, Liao PH, Pan YF, Chen SL, Chou SS, Chou MY (2013) The novel p53-dependent metastatic and apoptotic pathway induced by vitexin in human oral cancer OC2 cells. Phytother Res 27: 1154-1161.

Yang ZB, Luo XJ, Ren KD, Peng JJ, Tan B, Liu B, Lou Z, Xiong XM, Zhang XJ, Ren X, Peng J (2015) Beneficial effect of magnesium lithospermate B on cerebral ischemia-reperfusion injury in rats involves the regulation of miR-107/glutamate transporter 1 pathway. Eur J Pharmacol 766: 91-98.

Yang ZB, Tan B, Li TB, Lou Z, Jiang JL, Zhou YJ, Yang J, Luo XJ, Peng J (2014a) Protective effect of vitexin compound B-1 against hypoxia/reoxygenation-induced injury in differentiated PC12 cells via NADPH oxidase inhibition. Naunyn Schmiedebergs Arch Pharmacol 387: 861-871.

Yang ZB, Zhang Z, Li TB, Lou Z, Li SY, Yang H, Yang J, Luo XJ, Peng J (2014b) Up-regulation of brain-enriched miR-107 promotes excitatory neurotoxicity through down-regulation of glutamate transporter-1 expression following ischaemic stroke. Clin Sci (Lond) 127: 679-689.

Zhang HF, Li TB, Liu B, Lou Z, Zhang JJ, Peng JJ, Zhang XJ, Ma QL, Peng J, Luo XJ (2015) Inhibition of myosin light chain kinase reduces NADPH oxidase-mediated oxidative

injury in rat brain following cerebral ischemia/reperfusion. Naunyn Schmiedebergs Arch Pharmacol 388: 953-963.

Zhang S, Guo C, Chen Z, Zhang P, Li J, Li Y (2017) Vitexin alleviates ox-LDL-mediated endothelial injury by inducing autophagy via AMPK signaling activation. Mol Immunol 85: 214-221.

Zhou Y, Liu YE, Cao J, Zeng G, Shen C, Li Y, Zhou M, Chen Y, Pu W, Potters L, Shi YE (2009) Vitexins, nature-derived lignan compounds, induce apoptosis and suppress tumor growth. Clin Cancer Res 15: 5161-5169.

Zhu Q, Mao LN, Liu CP, Sun YH, Jiang B, Zhang W, Li JX (2016) Antinociceptive effects of vitexin in a mouse model of postoperative pain. Sci Rep 6: 19266.

Zucolotto SM, Fagundes C, Reginatto FH, Ramos FA, Castellanos L, Duque C, Schenkel EP (2012) Analysis of C-glycosyl flavonoids from South American Passiflora species by HPLC-DAD and HPLC-MS. Phytochem Anal 23: 232-239.

Figure legends

Figure 1. Effects of VB-1 on neurological deficit score and infarct following cerebral ischemia/reperfusion A. Neurological deficit scores (n = 14). B. Representative image of triphenyltetrazolium chloride-stained brain tissues from each group. C. Infarct volume in each group (n=7). All values were expressed as means ±S.E.M. I/R: ischemia/reperfusion; +VB-1(L), (M) or (H): ischemia/reperfusion + vitexin compound B-1(5 mg/kg) , (10 mg/kg) or (20 mg/kg); +Vehicle: ischemia/reperfusion + vehicle of VB-1(carboxymethyl cellulose sodium). $^{**}P$<0.01 vs Sham; $^{\#}P$<0.05, $^{\#\#}P$<0.01 vs I/R.

Figure 2. Effects of VB-1 on NOX4 expression and NOX activity in brain following ischemia/reperfusion A. NOX4 mRNA expression in brain. B. NOX4 protein expression in brain. Top, representative images of Western blot; bottom, ratio of optical density between NOX4 and β-actin. C. NOX activity in brain. All values were expressed as means ±S.E.M. (n = 7 per group). I/R: ischemia/reperfusion; +VB-1(L), (M) or (H): ischemia/reperfusion + vitexin compound B-1(5 mg/kg) , (10 mg/kg) or (20 mg/kg); +Vehicle: ischemia/reperfusion + vehicle of VB-1(carboxymethyl cellulose sodium). $^{**}P$<0.01 vs Sham; $^{\#}P$<0.05, $^{\#\#}P$<0.01 vs I/R.

Figure 3. NOX4-relevant miRNAs expressions in brain or nerve cells following ischemia/reperfusion or hypoxia/reoxygenation A. NOX4-relevant miRNAs expressions in brain (n=3 per group). B. miR-92b expression in brain (n=7 per group). C. miR-92b expression in NG108-15 cells (n=6 per group). All values were expressed as means ±S.E.M. I/R: ischemia/reperfusion; +VB-1(L), (M) or (H): ischemia/reperfusion + vitexin compound B-1(5 mg/kg), (10 mg/kg) or (20 mg/kg); +Vehicle: ischemia/reperfusion + vehicle of

VB-1(carboxymethyl cellulose sodium); H/R: hypoxia/reoxygenation; +VB-1(L), (M) or (H): hypoxia/reoxygenation + vitexin compound B-1(10^{-8} M), (10^{-7} M) or (10^{-6} M); +Vehicle: hypoxia/reoxygenation + vehicle of VB-1 (DMSO). $^{**}P$<0.01 vs Sham; ^{++}P<0.01 vs Control; $^{\#}P$<0.05, $^{\#\#}P$<0.01 vs I/R or H/R.

Figure 4. Effects of miR-92b mimics on NOX4 expression and NOX activity in nerve cells following hypoxia/reoxygenation A. miR-92b expression in NG108-15 cells. B. NOX4 mRNA expression in NG108-15 cells. C. NOX4 protein expression in NG108-15 cells. Top, representative images of Western blot; bottom, ratio of optical density between NOX4 and β-actin. E. NOX activity in NG108-15 cells. All values were expressed as means ±S.E.M. (n=6 per group). H/R: hypoxia/reoxygenation; + mimics: hypoxia/reoxygenation+ miR-92b mimics; +miRNA (-): hypoxia/reoxygenation+ miRNA negative control. $^{**}P$<0.01 vs Control; $^{\#\#}P$<0.01 vs H/R.

Figure 5. Effects of miR-92b mimics on hypoxia/reoxygenation-induced cellular apoptosis A. Representative images of Hoechst staining from each group. The apoptotic cells are indicated by arrows. B. Percentage of apoptotic cells per total number of NG108-15 cells in each group. C. Caspase 3 activity in NG108-15 cells from each group. All values were expressed as means ±S.E.M. (n = 6 in each group). H/R: hypoxia/reoxygenation; + mimics: hypoxia/reoxygenation+miR-92b mimics; +miRNA (-): hypoxia/reoxygenation+ miRNA negative control. $^{**}P$<0.01 vs Control; $^{\#\#}P$<0.01 vs H/R.

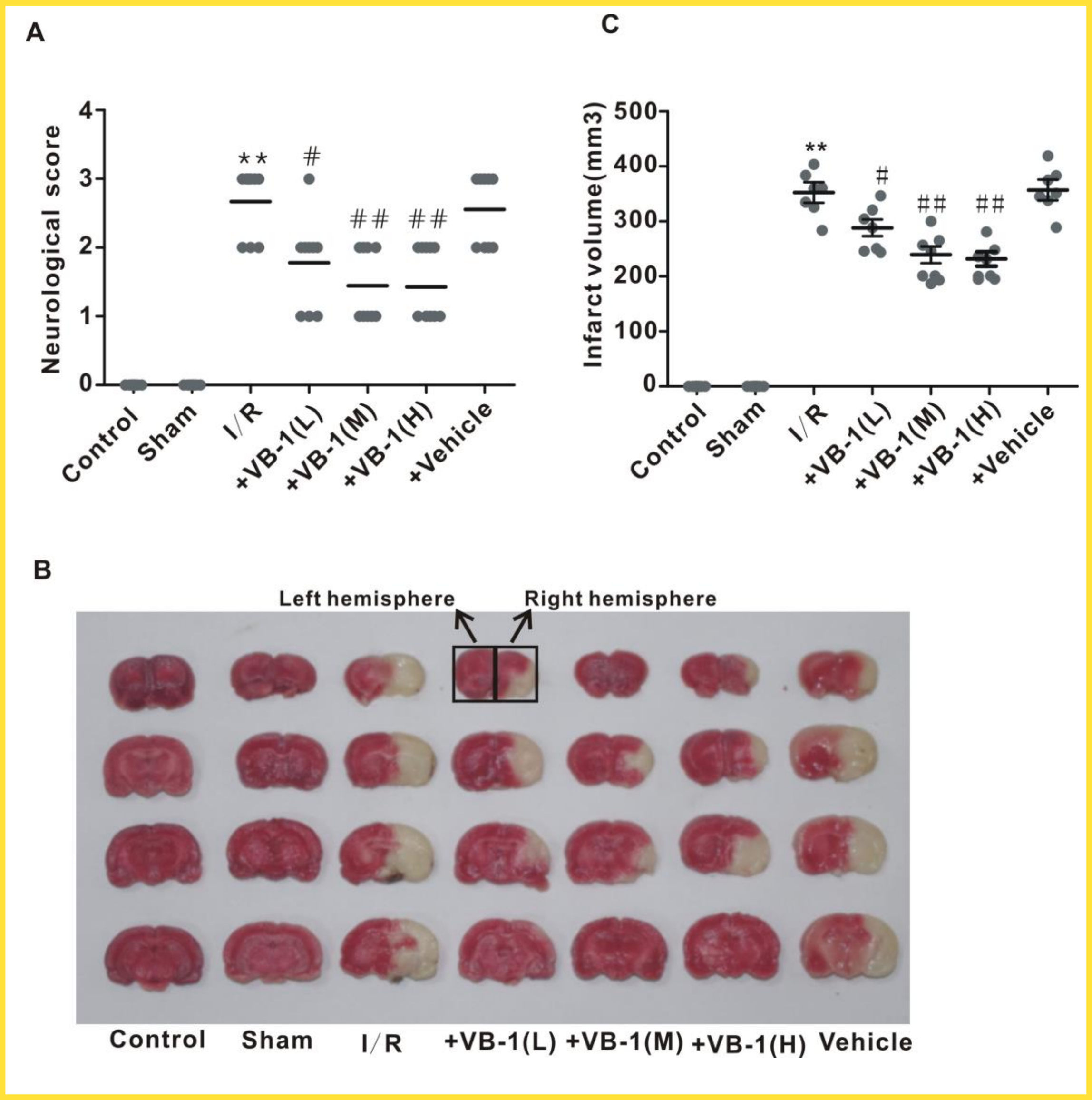
A
C
B
Neurological score
Infarct volume(mm3)
Control
Sham
I/R
+VB-1(L)
+VB-1(M)
+VB-1(H)
+Vehicle
Left hemisphere
Right hemisphere
Vehicle

Figure 1

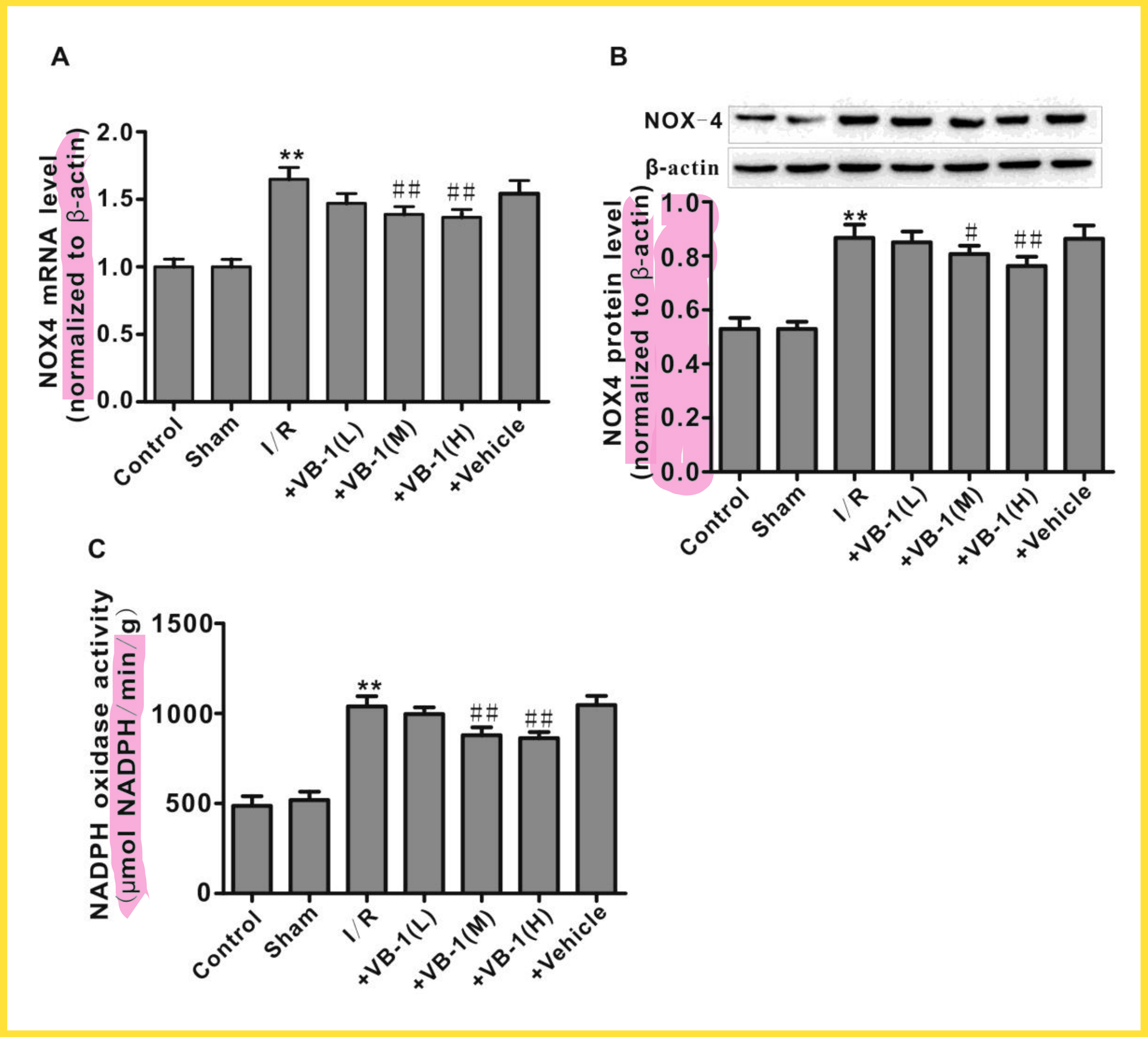
A
NOX4 mRNA level (normalized to β-actin)
2.0
1.5
1.0
0.5
0.0
**
##
##
Control
Sham
I/R
+VB-1(L)
+VB-1(M)
+VB-1(H)
+Vehicle
B
NOX-4
β-actin
NOX4 protein level (normalized to β-actin)
1.0
0.8
0.6
0.4
0.2
0.0
**
#
##
Control
Sham
I/R
+VB-1(L)
+VB-1(M)
+VB-1(H)
+Vehicle
C
NADPH oxidase activity (μmol NADPH/min/g)
1500
1000
500
0
**
##
##
Control
Sham
I/R
+VB-1(L)
+VB-1(M)
+VB-1(H)
+Vehicle

Figure 2

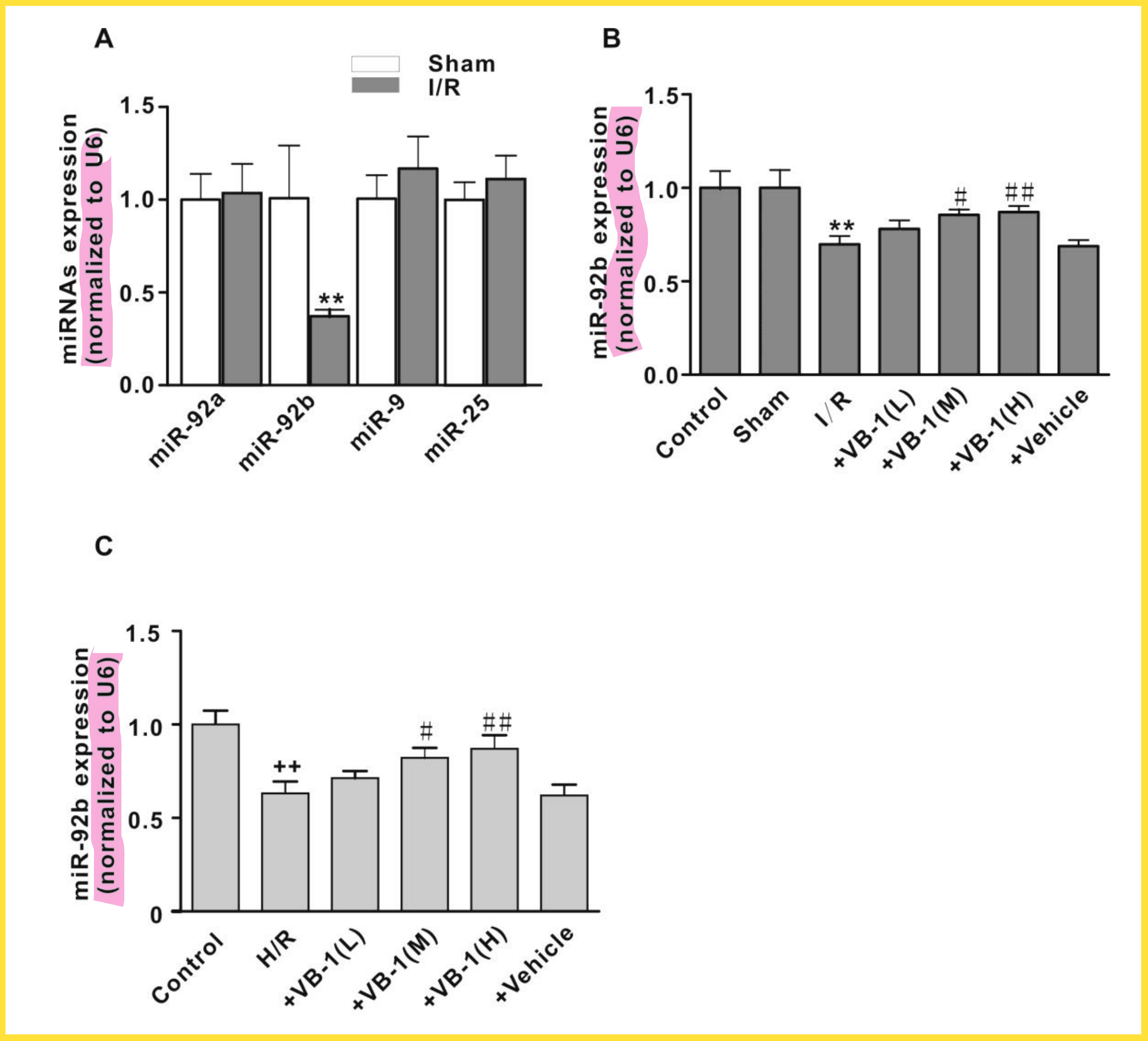
A
Sham
I/R
miRNAs expression
(normalized to U6)
1.5
1.0
0.5
0.0
**
miR-92a
miR-92b
miR-9
miR-25
B
miR-92b expression
(normalized to U6)
1.5
1.0
0.5
0.0
**
#
##
Control
Sham
I/R
+VB-1(L)
+VB-1(M)
+VB-1(H)
+Vehicle
C
miR-92b expression
(normalized to U6)
1.5
1.0
0.5
0
++
#
##
Control
H/R
+VB-1(L)
+VB-1(M)
+VB-1(H)
+Vehicle

Figure 3

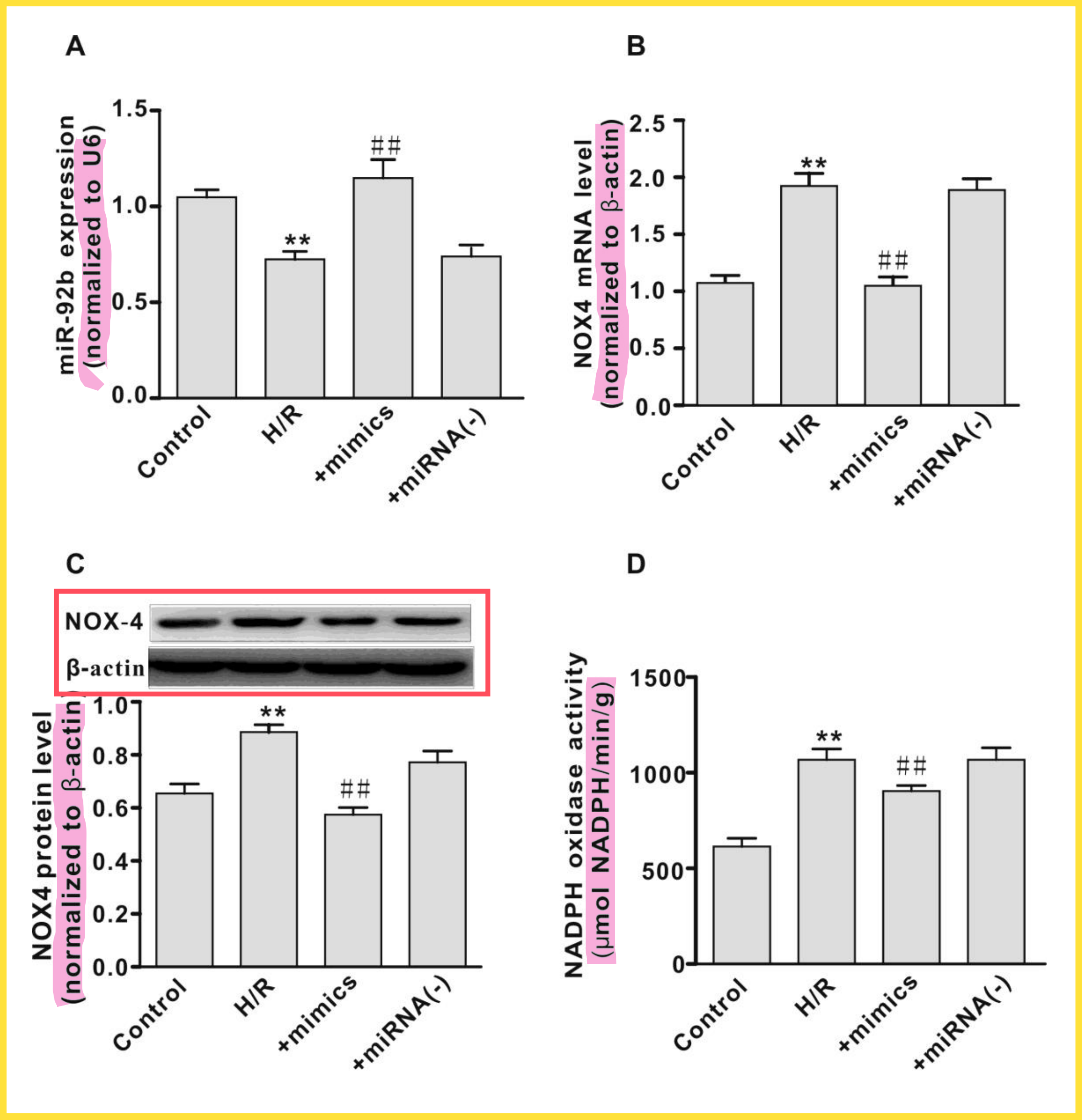

A
miR-92b expression
(normalized to U6)
1.5
1.0
0.5
0.0
**
##
Control
H/R
+mimics
+miRNA(-)
B
NOX4 mRNA level
(normalized to β-actin)
2.5
2.0
1.5
1.0
0.5
0.0
**
##
Control
H/R
+mimics
+miRNA(-)
C
NOX-4
β-actin
NOX4 protein level
(normalized to β-actin)
1.0
0.8
0.6
0.4
0.2
0.0
**
##
Control
H/R
+mimics
+miRNA(-)
D
NADPH oxidase activity
(μmol NADPH/min/g)
1500
1000
500
0
**
##
Control
H/R
+mimics
+miRNA(-)

Figure 4

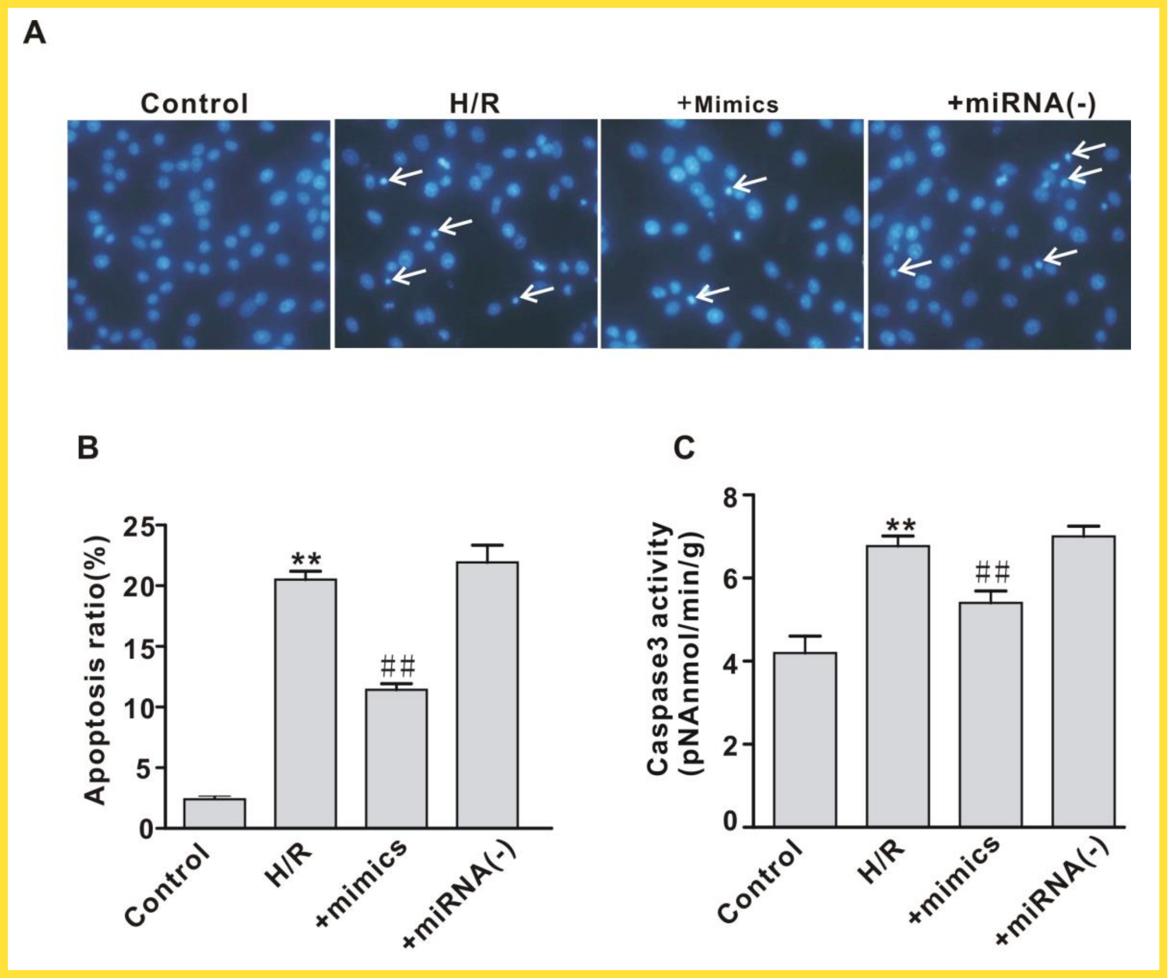
A
Control
H/R
+Mimics
+miRNA(-)
B
Apoptosis ratio(%)
25
20
15
10
5
0
**
##
Control
H/R
+mimics
+miRNA(-)
C
Caspase3 activity
(pNAnmol/min/g)
8
6
4
2
0
**
##
Control
H/R
+mimics
+miRNA(-)

Figure 5

Table

Table 1 Primers for real-time PCR

Gene (rat)	Forward primer	Reverse primer	Product size (bp)
NOX4	5'-TTGGATTTCTGGACCTTTGTG-3'	5'-TGGGTGGTTTCCTAATGCTGA-3'	205
β-actin	5'-CCCATCTATGAGGGTTACGC-3'	5'-TTTAATGTCACGCACGATTTC-3'	150

Click here to access/download
Supplementary Material
Tan B-Electronic Supplementary Material-R.doc
