# Supplementary material for: Fake paper identification in the pool of withdrawn and rejected manuscripts submitted to Naunyn–Schmiedeberg’s Archives of Pharmacology
Source: Naunyn Schmiedebergs Arch Pharmacol. 2023 Oct 5;397(4):2171–81. doi: 10.1007/s00210-023-02741-w (PMC10933159; doi:10.1007/s00210-023-02741-w)
Supplement: Supplementary file 10 — Supplementary file10 (PDF 947 KB) [file 210_2023_2741_MOESM10_ESM.pdf]

Figure S10

Color coding:

Yellow highlighted text	The text is identical in the NSAP version and the published version of this paper.
Red highlighted text	There are differences in the content between the NSAP version and the published version of this paper.
Blue highlighted text	The content is identical in both versions of this paper, but the text has been reworded.
Yellow bordered figure	This figure is very similar in both versions of this paper.

Naunyn-Schmiedeberg's Archives of Pharmacology
“Vascular Dementia: Experimental models and it’s mechanism”
--Manuscript Draft--

Manuscript Number:	NSAP-D-19-00247
Full Title:	“Vascular Dementia: Experimental models and it’s mechanism”
Article Type:	Review
Corresponding Author:	Nidhi Tiwari, M.Pharma<br>Kumaun University Nainital<br>INDIA
Corresponding Author Secondary Information:	
Corresponding Author's Institution:	Kumaun University Nainital
Corresponding Author's Secondary Institution:	
First Author:	Nidhi Tiwari, M.Pharma
First Author Secondary Information:	
Order of Authors:	Nidhi Tiwari, M.Pharma
	Jyoti Upadhyay
	Bhuwan Chandra Joshi
	Mahendra Rana
Order of Authors Secondary Information:	
Funding Information:	
Abstract:	VaD occurs due to cerebrovascular insufficiency which leads to decreased in the circulation to the brain resulting in mental disabilities. The main causes of vascular cognitive impairment (VCI) are severe hypoperfusion, stroke, high blood pressure (hypertension), large vessel disease (cortical), small vessel disease (subcortical vascular dementia), strategic infarct, haemorrhage (microbleed), cerebral autosomal dominant arteriopathy with subcortical infarcts and leukoencephalopathy (CADASIL) and cerebral amyloid angiopathy (CAA) as a result decreases the cerebrovascular perfusion. Many metabolic disorders such as diabetes mellitus, dyslipidemia and hyperhomocysteinemia related with vascular dementia. The rodent experimental models provide better prospective for investigation of molecular mechanism of new drugs. Plethora of experimental models are available that mimic the pathological conditions and leads to vascular dementia. This review article gives an update on understanding the basis of vascular dementia, risk factors, pathophysiology, mechanism, advantages, limitations and modification of various available rodent experimental models for vascular dementia. The research focus can be moved forward in this unestablished area.
Suggested Reviewers:	Ajudhia Nath Kalia, Ph.D.<br>Professor<br>ankalia_47@rediffmail.com
	Atish Prakash, PostDoctoral<br>atishprakash@gmail.com
	Minky Mukhija, Ph.D.<br>Assistant Professor<br>minkymukhija@gmail.com
	A.K.S Rawat, Ph.D.<br>Professor<br>rawataks@rediffmail.com

Powered by Editorial Manager® and ProduXion Manager® from Aries Systems Corporation

Manuscript
Click here to access/download;Manuscript;Manuscript_UP.doc
Click here to view linked References

Vascular Dementia: Experimental models and it's mechanism

Nidhi Tiwari[1], Jyoti Upadhyay[1], Bhuwan Chandra Joshi[1], Mahendra Rana[2]

[1]Research Scholar, Department of Pharmaceutical Sciences, Bhimtal, Kumaun University (Nainital), Uttarakhand, India

[2]Assistant Professor, Department of Pharmaceutical Sciences, Bhimtal, Kumaun University (Nainital), Uttarakhand, India

*Corresponding Author:

Nidhi Tiwari

Research Scholar

Department of Pharmaceutical Sciences

Bhimtal, Kumaun University (Nainital)

Uttarakhand-293136 (INDIA)

Email: tiwarinidhi0893@gmail.comContact no: +91-9458357488

ABSTRACT

VaD occurs due to cerebrovascular insufficiency which leads to decreased in the circulation to the brain resulting in mental disabilities. The main causes of vascular cognitive impairment (VCI) are severe hypoperfusion, stroke, high blood pressure (hypertension), large vessel disease (cortical), small vessel disease (subcortical vascular dementia), strategic infarct, haemorrhage (microbleed), cerebral autosomal dominant arteriopathy with subcortical infarcts and leukoencephalopathy (CADASIL) and cerebral amyloid angiopathy (CAA) as a result decreases the cerebrovascular perfusion. Many metabolic disorders such as diabetes mellitus, dyslipidemia and hyperhomocysteinemia related with vascular dementia. The rodent experimental models provide better prospective for investigation of molecular mechanism of new drugs. Plethora of experimental models are available that mimic the pathological conditions and leads to vascular dementia. This review article gives an update on understanding the basis of vascular dementia, risk factors, pathophysiology, mechanism, advantages, limitations and modification of various available rodent experimental models for vascular dementia. The research focus can be moved forward in this unestablished area.

Keywords: Angiopathy, CADASIL, Dementia, Hypoperfusion, Homocysteine

Abbreviations:

ADMA: Asymmetric dimethylarginine; BCCAS: Bilateral common carotid artery stenosis; BCCAo: Bilateral common carotid artery occlusion; BDNF: Brain-derived neurotrophic factor; CCA: Common carotid artery; CAA: Cerebral amyloid angiopathy; CADASIL: Cerebral autosomal dominant arteriopathy with subcortical infarcts and leukoencephalopathy; CBF: Cerebral blood flow; Cu/Zn-SOD:Copper/Zinc-superoxide dismutase; CVD: Cerebrovascular disease; DOCA: Deoxycorticosterone acetate; eNOS: Endothelial nitric oxide synthase; GABA: Gamma-amino butyric acid; HCA: Homocysteic acid; HE: Hematoxylin and eosin stain; HFD: High fat diet; HHcy: Hyperhomocysteinemia; IDDM: Insulin-dependent diabetes mellitus; IFN-γ: Interferon-gamma; IGF:Insulin-like growth factor; IHD: Ischemic heart disease; ILs: Interleukins; LDH: Lactate dehydrogenase; LMICs: Low and middle-income countries; MMP: Matrix metalloproteinase; MPO: Myeloperoxidase; MWM: Morris water maze; NADPH: Nicotinamide adenine dinucleotide phosphate; NF-κB: Nuclear Factor kappa-light-chain-enhancer of activated B cells; NIDDM: Non-insulin dependent diabetes mellitus; NMDA: N-methyl D-aspartate; ROS: Reactive oxygen species; SHRSP: Spontaneously Hypertensive Stroke Prone Rat; TNF-α:Tumor necrosis factor; UCCAo: Unilateral common carotid artery; VaD: Vascular dementia; VCI: Vascular cognitive impairment; WMH: White matter hyperintensities; WMLs: White matter lesions

Content

1. Introduction

1.1 Epidemiology

1.2 Risk factors

2. Pathophysiology and molecular mechanisms of VaD

3. Vascular dementia animal models

3.1 Vessel occlusion models

3.1.1 2-VO or BCCAo model

3.1.2 4-VO model

3.1.3 Unilateral CCA occlusion (UCCAO) animal model

3.1.4 Bilateral CCA stenosis (BCCAS) model

3.2 Models based on risk factors

3.2.1 Hypertension

3.2.2 Diabetic rats and mouse

3.2.3 Hyperhomocysteinemia (HHcy) induced VaD

3.2.4 Age

3.3 M5R (-/-) transgenic mice

3.4 CADASIL

3.5 Cerebral amyloid angiopathy

3.6 High fat diet induced VaD

3.7 Multiple infarcts models

3.7.1 Thromboembolic multiple infarcts VaD

3.7.2 Micro-spheres induced multiple infarcts VaD

3.7.3 Cholesterol crystals induced multiple infarcts VaD

4. Conclusion

References

1. Introduction

Dementia is a continuous neurodegenerative disorder affecting several cognitive domains mainly complex attention, executive functions, language, perceptual-motor, social cognition, learning and memory (Ratnatunge and Silva 2015). Nearly affects 1-2% of the population between the age of 65-75 yrs (vanderFlier and Scheltens 2005). It is also accompanied by the development of significant behavioral problems at any time during the course of illness and their frequency increases as the disease progression (Regier and Gitlin 2017; Burke et al. 2017).

VaD is a neurodegenerative disease and is the second most common cause of dementia after Alzheimer's disease (Reiger and Gitlin.2017). It is also associated with extensive morbidity and mortality (Tiwari et al. 2018; Ikram et al. 2017). The primary causes of cognitive impairment are cerebrovascular disease (CVD) and neurodegenerative pathologies including Alzheimer’s diseases (Toledo et al. 2013; Gorelick et al. 2011). However, unlike AD, there are no accomplished treatments for vascular dementia. The advancement in this area has been intricate due to lack information about the classification and its models. From this review we likely to relate cognitive and mechanistic relationship of experimental models including advantages and disadvantages.

1.1 Epidemiology

Dementia is an increasingly prevalent disease which is estimated worldwide affects approximately 36000000 persons (Alzheimer’s Society 2017). It occurs generally in the person above age 60-65 years (5%) and 75-80 years (20%) (Mallia et al. 2018). In the year 2001, approximately 60.1% people with dementia are living in developing countries (Rizzi et al. 2014) and predictable to rise 71.2% by 2040 (Rizzi et al. 2014; Dua et al. 2011). Further, in 2013 nearly 44.4 million people with dementia worldwide and it was estimated that by the end of 2015, there will be 9.9 million new cases of dementia. The observed dementing people in 2015 will almost double every 20 years (Wimo et al. 2013, Duthey 2013). It is also documented that low and middle income countries (LMICs) have a higher prevalence of dementia and expected to increase 58% in 2015, 63% in 2030 and 68% in 2050 (Prince et al. 2016; Chan et al. 2013). Globally, VaD is more common in men in contrast

with an AD that predominates in women. The prevalence of overall dementia are 32.3, AD 14.6, VaD 9.5, Lewy bodies dementia 1.4, mixed type 3.8, and other types 3.1 (Rizzi et al. 2014).

1.2 Vascular Risk factors

The vascular risk factors related to dementia can be divided into two groups:

(i) Modifiable: It includes those that reduce cerebrovascular disease (CVD) (Kling et al. 2013) e.g. hypertension, peripheral vascular disease (PVD), diabetes mellitus (DM), white matter lesions (WMLs), smoking, hyperlipidemia, ischemic heart disease (IHD), metabolic syndrome, sleep apnoea etc (Eizaguirre et al. 2017; Emdin et al. 2016).

(ii) Non-modifiable: It includes gender, age, genetics (family history of stroke/VaD), lower education, recurrent and multiple infarcts etc. (Mallia et al. 2018; Korczyn 2016). The major contributing factor involved in pathogenesis of VaD is cerebrovascular endothelial dysfunction (Kamat et al. 2015; Marshall 2012).These risk factors and characterstics features have been summarized in Figure.1.

2. Pathophysiology and mechanisms of VaD

VaD is a neurodegenerative diseases characterized by its pathological features which include WMLs, astrocytic gliosis, and loss of oligodendrocytes (Wallin et al. 2018; Flanagan et al. 2015; Nakaji et al. 2006). VaD is due to chronic hypoperfusion characterized by oxidative stress, hypoxia, generation of various mediators of inflammation resulting in progression of memory and cognition impairment (Tiwari et al. 2018; Farkas et al. 2007; Rufa et al. 2005). Oxidative stress is considered to be linked to mitochondrial dysfunction, apoptosis, vascular blood brain barrier (BBB) leakage, enhance neuroinflammatory response, endothelial dysfunction (Luca et al. 2015; Gill et al. 2010), hippocampal damage as a result of increase in the production of free radicals and reactive oxygen species, thereby modulates nitric oxide synthase (NOS) pathway (Zhang et al. 2014; Li et al. 2013). Moreover, oxidative stress imbalances the ratio of reactive oxygen species (ROS) and antioxidants results in the damage of endothelial, glial, neuronal cells, as well as neurovascular uncoupling which further

reduces cerebral blood flow (Freeman and Keller 2012; Liu and Zhang 2012). It has been documented that cerebral white matter (WM) is extremely vulnerable to oxidative stress-induced damage and poor blood flow in deep structures due to its limited blood supply into the brain (Luca et al. 2015; Back et al. 2002). Moreover, periventricular white matter, basal ganglia, and hippocampus are also extremely affected by hypoperfusion induced lesions in VaD (Kalaria et al. 2016; Venkat et al. 2015).

Thus, before developing a good animal model of VaD, it is essential to know the pathology and mechanisms underlying VaD. It is hypothesized that the decrease in cerebral blood flow (CBF) is the main culprit which is responsible for the origin of VaD. However, there is still a wide scope is needed to understand the mechanistic pathway and pathological characteristics associated with VaD

Figure 2. Summarizes the mechanistic pathway for the pathogenesis of VaD.

3. Vascular dementia animal models

There are various models to demonstrate vascular abnormalities that lead to VaD. It includes vessel occlusion bilateral common carotid arteries occlusion (BCCAo), 2VO (vessel-occlusion), 4VO, unilateral common carotid arteries (UCCAo), and bilateral common carotid arteries stenosis (BCCAS) models. Models with risk factors like hypertension, diabetes, hyperhomocysteinemia, age, showing a strong relationship with cognitive impairment. Multiple infarct type of dementia can be produced by thromboembolic, microspheres/microbeads, and cholesterol crystals. It concludes the relevancy of the multiple infarction VaD models. Previous studies showed positive correlation between high-fat diet (HFD) and cognitive deficits in hippocampal-dependent learning and memory process.

The various experimental models of VaD discussed in Table1.

3.1 Vessel occlusion models

In this model, blood vessels are being obstructed to provide a constant reduction in the regional CBF and various nutrients to the brain region resulting in the development of dementia (Farkas et al. 2007).

Mechanism

VaD is caused by ischemic hypoxia damage to the brain (Wang 2014). BCCAo in rats resulting in reduced blood flow and the cholinergic dysfunction under condition of chronic cerebral hypoperfusion (Kaundal et al. 2018; Wakita et al.1994). Disturbances in cholinergic neurons is due to enhance oxidative stress which consequently produces hypoglycemia i.e. decreased glucose supply to the brain and decrease the availability of adenosine tri-phosphate (ATP) resulting in the energy-dependent ion pumps (Briede and Duburs in press; Plaschke 2005), depolarization of neurons, and the generation of ROS (Farkas et al. 2007). The formation of ROS initiates lipid peroxidation and decreases activities of antioxidants i.e. Cu/Zn-superoxide dismutase (Cu/Zn-SOD) and glutathione (GSH) (Nita et al. 2001). Chronic hypoperfusion resulting in the succession of ischemic brain injury due to release of various cytokines viz. interleukins (ILs) dysregulation and cerebrospinal fluid (CSF) IL-6 level increases (Chamorro and Hallenbeck 2006; Wada-Isoe et al. 2004; Wilson et al. 2002). Further, various inflammatory biomarkers like α-1-antichymotrypsin and C-reactive protein increases in VaD (Engelhart et al. 2004).

The vessel occlusion model is associated with the cognitive impairment of VaD shown in Figure 3.

Some of the methods used to produce VaD are discussed below:

3.1.1 2 VO (Two vessel occlusion) or BCCAo model

2-VO or BCCAo model is a well-established and commonly used model to produce subcortical ischemic vascular dementia (Xi et al. 2014; Jiwa et al. 2010). This model is used only when moderate but insistent reduction of CBF is needed in the region of forebrain (oligemia) (Kastner et al. 2005). 2VO model is a global chronic cerebral hypoperfusion, in which both the common carotid arteries of rats are subjected to surgically ligation (occlusion) to produce chronic global hypoperfusion state induced VaD (Kitamura et al. 2012; Hachinski et al. 2006).

Procedure:

The detailed procedure of BCCAo/2VO as illustrate below:

Animal- Swiss albino male mice (25-30g) and Wistar rats, *Anesthesia*- ketamine (80 mg/kg) and xylazine (20 mg/kg) (Stasiak et al. 2014). After anaesthetized, a small incision is made in the neck region and both the common carotid arteries are ligated (Tiwari et al. 2018; Hobbenaghi et al. 2014). It is documented by Kim et. al. both carotid arteries is ligated for 15 min with aneurysm clips. After 15 minutes, both clips are removed, followed by reperfusion of blood flow. If needed, antibiotics are applied to the mice and returned to their home cage (Kim et al. 2009). Another method showed repeated cerebral ischemia is the most suitable and best model for the development of VaD. The carotid arteries were occluded for 10 min and then relaxed for 10 min, and repeated for three times (Tiwari et al. 2018; Wang 2014). To overcome increase in blood pressure at the time of occlusion, sodium nitroprusside is injected intraperitoneally (3.5 mg/kg) (Wang 2014).

Morris water maze (MMW), radial arm maze test (7 days post surgery), object recognition task and holeboard tests determined working and reference memory (Stasiak et al. 2014; de Bortoli et al. 2005). The locomotor activity remained intact in this model (Xi et al. 2014; Kudo et al. 1993). The brain is dissected after BCCAo at different time point intervals such as 2, 6, 12, 24, 72 hr and 7th day for PCR and western blot analysis (Ranjithkumar et al. 2015).

3.1.2 4-Vessel Occlusion (4-VO) model

In this model ischemia can be produced by clamping of both carotid arteries and vertebral arteries of rats (4-VO) usually for 10–20 min. The neuronal loss can be take place in hippocampal area mainly CA1 region, Cortex and Thalamus (Yamaguchi et al. 2005; Petito et al. 1998).

The detailed procedure as follows in mice and rats:

Procedure in Mice

Animal- Swiss Albino Mice (25-30gm), *Anesthesia*- Avertin (2,2,2-tribromoethanol; 125 mg/kg, *i.p.*) (Akdemira et al. 2014). After anesthesia, both common carotid arteries are occluded, using micro-clip applicator for 10-15 minutes (Akdemira et al. 2014). Monitoring of mice is necessity during surgery for proper breathing and

ambulation (0.1 mg/kg s.c). However, it is also essential to maintain core body temperature 36.5–37.5 Celsius by using a heating pad and overhead lamp (Akdemira et al. 2014; Yamaguchi et al. 2005). The radial 8-arm maze task is checked for spatial working memory. This impairment may also be due to the transient imbalance of neurotransmitters such as acetylcholine (Ach) and nor-epinephrine (NA) (Chung et al. 2002).

Note: It is reported that in the Pulsinelli and Brierley's four-vessel occlusion (4VO) model is used for brain ischemia research. There are some problems for arranging a surgery to ligate the basilar artery for collateral circulation (Ostrowski et al. 2005). So to overcome these limitations there are various modified models which is safe, quick to induce bilateral hemispheric ischemia and is minimally invasive (Venkat et al. 2015; Liu et al. 2012). An improved 4-vessel occlusion (I4VO) rat model decreases the collateral circulation by using a one-stage aperture thoracic superior approach. Previous studies showed 1-stage modified model is an anterior approach for making bilateral hemispheric ischemia in which vertebral arteries (VAs) and CCAs can be completely occluded at the same time for the desired duration. Modified 4VO method decreased cerebral blood flow to 12% to 14% of baseline levels (Walker and Rosenberg 2010).

Procedure in Rats

Animal- Male Sprague-Dawley rats (340-390g), *Anesthesia*- Chloralose (40 mg/kg IP) and urethane (400 mg/kg IP) (Yamaguchi et al. 2005).After anesthetized both carotid arteries are exposed and incision is made in the cervical region. Both carotid arteries are ligated with microvascular clips for 30 min and then incision is closed with suture (Colbourne et al. 1999). All the test is performed after 30 days of 4-VO. Spatial learning memory is assessed by Morris water maze (MWM) (Li et al. 2012).

3.1.3 Unilateral CCA occlusion (UCCAO) model

In this model short-term memory impairment is produced by right carotid artery occlusion. Previous studies reported C57BL6 mice showed reduction in the cerebral blood flow (50–70%) in the ipsilateral hemisphere (Kitamura et al. 2012; Shibata et al. 2004). The detailed procedure of UCCAo, as illustrated below:

Procedure

Animal: Twelve-week-old male mice (24 to 26 g), Strain: C57BL/6J, *Anesthesia:* induced by 5% isoflurane and for maintenance use mixture of 30% O_2/70% N_20 (Yoshizaki et al. 2008). A midline cervical incision is made and right CCAs is ligated with 6-0 silk sutures. After surgery animals are kept in their cages with free access to food and water (Yoshizaki et al. 2008). Memory and learning impairment is assessed by Novel object recognition task. Other parameters such as open field and elevated maze test are also used to ensure the impairment in cognitive function (Venkat et al. 2015).

3.1.4 Bilateral CCA stenosis (BCCAS) model

Bilateral common carotid artery stenosis (BCCAS) is a frequently used most promising model of chronic cerebral hypoperfusion induced subcortical ischemic vascular dementia (Roberts et al. 2018; Bink et al. 2013). The BCCAS model also exhibits characteristic features of hypo perfusion vascular cognitive impairment such as WM rarefaction, gliosis, and working memory impairment (Hattori et al. 2016; Nishio et al. 2010). The detailed procedure of BCCAS as summarized below.

Procedure:

Animal: Mice (3-month-old male strain C57Bl/6) (Roberts et al. 2018) and Wistar Kyoto Rat (20 wk old) (Matin et al. 2016), *Anesthesia:* 5% isoflurane or ketamine/xylazine (Patel et al. 2017). In this model occlusion were made by placing micro-coils usually for 30 min around CCAs. It is essential to select the inner inner core diameter of the micro-coil as it determines the extent of the cerebral damage (Nishio et al. 2010; Shibata et al. 2007). WM lesions are observed after 7 days in rats and 14 days in mice (Shibata et al. 2004). Cognitive dysfunction is observed by 5-6 months after BCAS. The characteristics features changes are hippocampal atrophy, Matrix metalloproteinases activation, gliosis and BBB disruption (Nishio et al. 2010; Nakaji et al. 2006). Memory and learning deficit is evaluated by radial arm and Barnes maze (Nishio et al. 2010; Shibata et al. 2007).

Table 2 highlights advantages and limitations of vessel occlusion model

3.2 VaD Models based on risk factors

3.2.1 Hypertension

The multifactorial disorder hypertension is characterized by elevated systemic blood pressure that may result in severe vascular endothelial dysfunction and related dementia (Dikalov and Dikalove 2016; Coffman 2011). Previous studies reported that stroke-prone spontaneously hypertensive rats (SHRSP) is the suitable model for essential hypertension-induced vascular consequences and related dementia (Kimura et al. 2000). Stroke-prone rats developing hypertension during age 8-9 weeks, reaching severe hypertension at age 12 weeks (Hainsworth and Markus 2008).

Mechanism

Hypertension contributes to cognitive deficit via a several mechanisms: hypertrophy, enhance vascular resistance, endothelial dysfunction and contraction of vascular smooth muscles leading to a reduced lumen size (Sierra et al. 2012). Hypertension is likely to cause various alterations in macrovascular and microvascular levels (Scuteri et al. 2013; Triantafyllidi et al. 2009). These changes decrease blood supply to the brain and increase production of ROS, activates arginase expression in the endothelial cell of coronary artery (Zhang et al. 2004; Faraco and Iadecola 2013; Iadecola 2013). Arginase is an enzyme that helps in the conversion of L-arginine to urea and ornithine. L-arginine acts as a substrate for both of the enzymes endothelial nitric oxide synthases (eNOS) and arginase (Tousoulis et al. 2002). Increase in arginase expression leads to decrease in L-arginine availability to eNOS, thereby decrease nitric oxide (NO) synthesis (Romero et al. 2008). Previous studies showed that increased in the arginase activity contributes to vascular endothelium dysfunction in various pathological conditions, (Chandra et al. 2012; Chen et al. 2001) probably leads to VaD.

Mechanism based on specific model described as follows:

1: **DOCA salt induced hypertension** decreases activity of plasma rennin due to sodium retention (Gavras et al. 1975). These changes contribute to increase in superoxide anion or free radical results in vascular endothelial dysfunction, key factor in VaD (Jimenez et al. 2007; Wu et al. 2001). Previous studies documented endothelin-1 (ET-1) is potent vasoconstrictor and enhance vascular $O_2^{\bullet-2}$ production by activating ETA/NADPHoxidase pathway (Dai and Dai 2010). Further studies showed increase in enzyme xanthine oxidase and mitochondrial-

derived ROS in the model of hypertension hypertension (Viel et al. 2008; Callera et al. 2006). Xanthine oxidase catalyzes the oxidation of hypoxanthine and xanthine to form free radical ($O_2^{\cdot-}$) which is present in the vascular endothelium (Lacy et al. 1998).

2: **Sodium arsenite-induced VaD** linked with oxidative stress via a activation of oxidative sensitive signaling pathway (Shi et al. 2004). Oxidative stress contributes to formation of ROS, increases activity of acetyl-cholinesterases (AChE), decreases NO protective activity (Versari et al. 2009; Tsou et al. 2005), all these factors leads to vascular endothelial dysfunction (Balakumar et al. 2008).

3: **Stroke-prone spontaneously hypertensive model** develops cognitive impairment via a several mechanism viz. WM lesions, reactive gliosis, small vessel wall hardening (Chiba et al. 2012), BBB disruption, myelin damage and mediates inflammatory response (Henning et al. 2010). All these factors collectively damages endothelium cell resulting in neurological deficit (Schreiber et al. 2013; Jalal et al. 2012). In this model there is sustained vulnerability to ischemic/hypoxic damage (Uluc et al. 2011). Moreover, enhance activity of endothelial xanthine oxidase and ROS production directly leads to increased arteriolar tone (Suzuki et al. 1998).

Procedure:

Some of the methods used to produce VaD are discussed below:

3.2.1.1. DOCA-salt induced hypertension-VaD:

Animal: Albino Wistar rats (200-240g), *Anaesthesia:* Mixture of Ketamine (60mg/kg) and xylazine (10mg/kg) and unilateral nephrectomy is performed (Sharma and Singh, 2012; Han et al. 2015). Hypertension is induced by a administered DOCA (15 mg/kg, s.c. twice weekly) for 3 month, normal water is replaced by chloride salt of Na (1%) and K (0.2%) (Sharma and Singh 2012; Bockman et al. 1992). Behavioural parameters are assessed by MWM task from 86th day onwards (Kubota et al. 2006). Arterial blood pressure is measured by BIOPAC MP100, using AcqKnowledge 3.8.2. analysis system (Sharma and Singh 2012).

3.2.1.2 Sodium arsenite-induced vascular dementia

Sodium arsenite induced VaD characterized by changes in biochemical, pathological, developmental and clinical features of cognitive impairment related dementia (Gong and O'Bryant 2010).

Animals: Adult male Wistar rats (Kesavan et al. 2014), *Chemical inducer:* Sodium arsenite (1.5 mg/kg/day i.p.) administered to animals for 2 weeks. Assessment of behavioral parameters starting from 41st day and histopathological analysis is done to confirm inflammation (Kesavan et al. 2014; Kaur et al. 2010).

3.2.1.3 Stroke-prone hypertension induced VaD

Animals: SHRSP/Izm and Wistar-Kyoto normotensive rats (WKY/Izm) were included in this study. The former strain receiving high-salt (0.8% NaCl) and low-protein (20.8%) stroke-prone diet (Abraham et al. 2002). SHRs are normotensive at birth and progressively develop stable hypertension in the first 2–4 months of life (Zhao et al. 2016). Animals develop ischemic lesions primarily in the cerebral cortex, hippocampus and the basal ganglia (Zhao et al. 2016, Yamori et al. 1976).With the aid of computerized sphygmomanometer, heart rate and systolic blood pressure is to be measured regularly (Minami et al. 1997; Abraham et al. 2002). After that, the animal is anaesthetized by using halothane and both CCA are occluded for 10 min with aneursymal clips. Further, BBB permeability is also determined after a fixed interval such as 30 min, 6 hr, 1, 5, 7 and 28 days after ischemia (Abraham et al. 2002). Furthermore, several assessments such as behavioral, histopathological, biological parameters is observed. The passive avoidance task is suitable for behavior assessment because it's utilizes the inherent behavior of rats (Kimura et al. 2000; Abraham et al. 2002).

3.2.2 Diabetic induced-VaD in rats and mice

Diabetes mellitus (DM) is a metabolic disorder of carbohydrate, fat and protein consequently fasting or postprandial blood glucose levels are increased. It is classified into two types: type 1 (insulin dependent, IDDM) and type 2 (non-insulin dependent, NIDDM), depending upon either disease is occurring due to impaired insulin release or insulin action (Abhar and Schaalan 2014). The risk factor of VaD 1.5-4 fold in diabetic people (Araki 2010). Type 2 DM being atherogenic is a chief risk factor for cerebral blood vessels as well as thromboembolic stroke-induced VaD (Luitse et al. 2012).

Mechanism

Diabetes is associated with enhance release of inflammatory cytokines augment oxidative stress-induced ROS, leading to decrease in NO production (Taye et al. 2010). The cerebral blood flow is maintained by production of NO, which is responsible for integrity/stability of vascular endothelium, inhibition of platelet aggregation and leukocyte adhesion (Caldwell et al. 2010). Moreover, ROS further lead to the decrease in NO production which is responsible for vascular endothelial dysfunction and related dementia (Liu et al. 2014; Pasquier et al. 2006).

Table 3 shows some advantages and disadvantages of hypertension-induced VaD.

3.2.2.1. Streptozotocin (STZ) induced diabetes

STZ is a broad-spectrum antibiotic possessing antitumor, diabetogenic and oncogenic properties (Like and Rossini 1976). Its diabetogenic properties are characterized by selective destruction of pancreatic islet β-cells and capable of inducing insulin-dependent diabetes mellitus (IDDM) or Type 1 DM, leading to insulin deficiency, hyperglycemia, polydipsia, and polyuria (Kolb 1987; Wu and Huan 2008).

Procedure in Mice

Animal: C57BL/6 or CD-1 male mice (Price et al. 2015), *Drug:* STZ (50mM) dissolve in citrate buffer (pH 4.5) (Furman et al. 2015). The mice injected with STZ at a dose 40 mg/kg, i.p (Murtishaw et al. 2018). Animals provided 10% sucrose water and from 6th day onwards it is replaced regular water (Furman et al. 2015; Wu and Huan 2008; Like and Rossini 1976). Fasting is essential on 9th day for about 6 hr. monitoring of blood glucose is calculated by one touch basic blood glucose monitoring system to ensure hyperglycemia in the STZ-treated subjects (Furman et al. 2015; Murtishaw et al. 2018).

Procedure in Rats

Animal: Wistar rats (250-300g) (Sharma and Singh 2011). Diabetes is produced in rats by injecting single dose of STZ, 50mg/kg i.p. dissolved in 0.1 M citrate buffer (pH 4.5). Behavioral parameters assessed by MWM task

shows learning and memory in rats (Sharma and Singh 2011). The entire test is performed 52nd day after STZ dose (Sharma and Singh 2010, 2011; Parle and Singh 2004; Morris 1984). Monitoring of blood sugar level is important after an interval of one week so as to prevent mortality (Sharma and Singh 2010, 2011; Brosky and Logothetopoulos 1969).

Note: STZ is unstable and degrades immediately after 15-20 min in citrate buffer. It must be prepared freshly before injecting into animals (Furman et al. 2015; Wu and Huan 2008). Reported data showed female are less sensitive to beta-islet cell toxin than males. Better results shown by Male diabetic rats (Kolb 1987).

Table 4 summarized advantages and disadvantages of STZ-induced VaD.

3.2.3. Hyperhomocysteinemia (HHcy) induced-VaD

Elevated homocysteine is a major determinant of cerebrovascular (brain atrophy/damage, stroke, vascular diseases, cognitive/memory decline) and cardiovascular disorders (carotid artery disease, atherosclerosis etc (Bhatia et al. 2014; Clarke et al. 2014; Lentz and Haynes 2004). Moreover, also impaired metabolism due to deficiency in various cofactors (vitamin B_6, B_{12}, folate) genetic alterations in metabolic enzymes (Methionine synthase, methyltetrahydrofolate reductase, cystathionine β-synthase and cystathionine-γ-lyase) (Bhatia and Singh 2015). This model is accepted in both rats and mice. HHcy is categorized into mild (12-30 µmol/L), moderate (30-100 µmol/L) or severe (>100µmol/L) (Streck et al. 2003; Lee et al. 2005).

Mechanism

Hyperhomocysteinemia results in excessive production of oxidation products, homocysteine thiolactone and homocysteine mixed disulfides, may lead to endothelial dysfunction due to enhance sulfation of connective tissue (den Heijer et al. 2003; Obeid and Hermann 2006). HHCy induced oxidative stress due to formation of various inflammatory mediators viz. IFN-γ, NFκ-B, TNF-α results in dysfunction of endothelial progenitor cell and RBCs hemolysis (Sudduth et al. 2013; Bhatia et al. 2014). Further, HHcy induced formation of excitatory

amino acid neurotransmitters homocysteic acid (HCA) and cysteine sulphinic acid (CSA) (Bleich et al. 2000; Obeid and Herrmann 2006) leads to activation of N-methyl-D-aspartate (NMDA) glutamate receptor (Boldyrev 2010). The activation of glutamate receptor results in generation of ROS, responsible for dementia (Bhatia and Singh 2015; Machado et al. 2011). Previous studies showed HHcy induce microvascular permeability by reducing the effect of GABA-A/B receptors and activates disintegrin and metalloproteinase, thus resulting in oxidative stress (Abahji et al. 2007; Ramin et al. 2014).

Procedure

3.2.3.1 L-Methionine induced vascular dementia

Animal: Adult male Wistar albino rats (Dessouki et al. 2017). The rats is administered L-Methionine (1.7 g/kg/day, p.o.) either for 4 weeks (Shah and Singh 2007) or 8 weeks (Sain et al. 2011; Koladiya et al. 2008) to produce hyperhomocysteinemia-induced endothelial dysfunction (Dessouki et al. 2017). Behavioral assessment is evaluated by MWM test after 4/8 weeks (Koladiya et al. 2008). Endothelial dysfunction is analysis by measuring acetylcholine-induced endothelium-dependent relaxation and sodium nitroprusside-induced endothelium-independent relaxation using isolated aortic ring preparation along with an estimation of serum nitrite concentration (Sharma and Singh 2012; Pieper 1997).

Note: In case of mice, HHcy can be produced by administering a diet deficient in vitamins B6, B12, and B9 supplemented with methionine for 14 weeks to 6 months (Sudduth et al. 2013).

Table.5. highlights some advantages and disadvantages of HHcy induced VaD.

3.2.4 Age

Aged animals are routinely used as natural models of VaD. Age-related cognitive decline and behavioral changes such as morphological alterations in the vasculature results in hypoperfusion of blood supply to the brain (Balbi et al. 2015). This model is used in both rats and mice resulting in impairment in memory, subsequently VaD (Kempsell and Fieber 2015; Erickson and Barnes 2003). Several factors that contribute to

the aging process such as genetics (Sinclair and Guarente 2006; Brown-Borg et al. 1996), metabolism (Barzilai et al. 2012), diet (Wood et al. 2004) and stress (Finkel and Holbrook 2000).

Mechanism

Aging increases stiffness of cerebral blood vessel and thinning of endothelial cells of carotid artery thereby causes reduction in CBF (Park et al. 2007; Faraci and Heistad 1998). By this, aging increases the incidence of vascular cognitive impairment (Rothwell et al. 2005; Farkas and Luiten 2001) and related dementia. It has been documented that aging-induced impairment in cerebrovascular function is mediated by generation of oxidative stress (Shaik et al. 2013; Farkas and Luiten 2001). Moreover, aging increases the activation of NADPH oxidase enzyme in cerebral blood vessels, which is a major source of ROS (Park et al. 2007). It has been shown that ROS has damaging effect on the endothelial cell of blood vessels thereby modulate in the structure and functioning of vessel wall which is related to VaD. Previous studies documented that aging impairs endothelium-dependent relaxation which is mediated by Nox2-derived ROS (Park et al. 2007) produces alteration in vascular reactivity (Papadopoulos et al. 1998). Aging induced changes in various body metabolic process that leads to a disease like diabetes, cardiovascular diseases, stroke which is the known risk factor for VaD (Barzilai et al. 2012).

Procedure

Animal: Male Wistar albino rats (Gocmez et al. 2016). The rats are divided into different age groups: Mature adult rats (6 month), middle-aged (12-14 month); old (24-26 months). Animals are placed either standard or enriched conditions (Goldman et al. 1987). In case of standard housing condition rats are kept in suspended cages (21x34×20 cm) made of stainless-steel whereas in enriched condition animals are placed in large clear plastic chambers (70×70×45 cm high) filled with a variety of stimulus objects (e.g., ladders, boxes, tunnels etc.) changed according to pool of such objects (Goldman et al. 1992). The rats remained in their respective cages for about 60 days on a 12 hr light with free access to food and water. After 60 days rats are rehoused into separate cages with food restricted diet prior to behavioural parameters and histopathological analysis (Goldman et al. 1992; Goldman et al. 1987).

Table 6 highlights advantages and disadvantages of aging-induced VaD.

3.3 M5R receptor deficient (-/-) transgenic mice

M5 cholinergic receptor is an important therapeutic target to improve working and reference memory produced by cerebrovascular dysfunction. These receptors are mainly located in cerebral cortex, hippocampus and areas important for brain function (Araya et al. 2006). M5 receptors are also present in endothelial cells of circle of willis and plays a major role in the Ach-induced relaxation of cerebral arteries (Hamel 2004; Yamada et al. 2001). M5 receptor-deficient mice (M5R−/− mice) (male or ovariectomized female) are important for VaD model. Previous studies confirmed permanent lack of M5R in transgenic mice extensively decreases CBF in several regions of brain viz. cortex, hippocampus, basal ganglia, and thalamus (Faraci and Sigmund 1999) thus, resulting in cognitive impairment. Behavioural parameter assessed by Y-maze task, object recognition task (Araya et al. 2006).

3.4 CADASIL

Cerebral autosomal dominant arteriopathy with subcortical infarcts and leukoencephalopathy (CADASIL) is an inherited monogenic small vessel disease caused by fibrous thickening of arterial wall and destruction of vascular smooth muscle, characterized by gradually diffuse bilateral subcortical white matter hyperintensities (WMH) (Mizuno 2015; Chui 2007) and multiple lacunar infarcts (Sathe et al. 2015). Cerebral arteriopathy results from a mutation in a gene called neurogenic locus notch homolog protein 3 (NOTCH3) on chromosome 19 is marked by migraine headaches, premature stroke, and vascular dementia (Venkat et al. 2015; Tournier-Lasserve et al. 1993). Further, it has been reported that all NOTCH3 mutation carriers showed WM lesions contributes to cognitive dysfunction (Jennifer and Donna 2016). The NOTCH3 receptor mainly found in adult VSMCs and is related to the maintenance of vascular structural and functional stability (Kalaria et al. 2008). The genetic mutation in the NOTCH3 gene disturbs homeostasis of blood vessels, leading to alteration in the integrity of vascular wall which ultimately decreases the supply of blood to the brain thus produce a condition of chronic hypoperfusion that produces loss of cognition and memory impairment (Iadecola 2013; Kalaria et al.

2008). Moreover, it has been shown that CADASIL results in psychiatric disorders especially mood changes associated with the neurological deficit (Tikka et al. 2014).

3.5. Cerebral amyloid angiopathy (CAA)

CAA characterized by severe disruption of the vascular architecture with a double outline barreling, microaneurysm formation, fibrinoid necrosis, vasculitis and evidence of previous microscopic haemorrhages (Farkas & Luiten 2001; Vinters et al. 1996; Vinters 1987). The pathogenesis of CAA induced VaD consists of two steps:

(1) Cerebrovascular amyloid deposition in which amyloidogenic protein is deposited in the cerebral and leptomeningeal blood vessels (Yamada 2015) resulting in neuronal damage.

(2) Vascular degenerative changes including disruption, occlusion, and permeability changes (Yamada et al. 2015; Ghiso et al. 2010) leads to Vascular endothelium dysfunction associated dementia.

3.6 High-fat diet-induced-VaD

Obesity is a important factor for hippocampus-dependent learning and memory impairment and responsible for various types of dementia. Saturated fat especially palmitic acid initiates the obesity and several path biological factors viz. microglia activation, neuroinflammation (Duffy et al. 2019; Chandalia and Abate 2007). Multiple diseased conditions such as atherosclerosis, Alzheimer's disease, thrombosis, accumulation of amyloid disturbed the membrane function and contribute to dementia of vascular origin (Davidson et al. 2013; Kalmijn 2000).

Mechanism

There is a key link between a HFD intake and cognitive impairment associated VaD. The cause of high-fat induced VaD proposed by two mechanisms:

1: Disturbed lipid homeostasis enhances the production of ROS {Superoxide anion (O^{2-}), H_2O_2} inflammatory mediators (ILs-1, ILs-6, and TNF-α), prostaglandins (PGE2) and activation of nuclear factor kappa B (NF-κB) in the brain resulting in oxidative stress (Liu et al. 2017; Rader 2000; Pickup and Crook 1998).

results in decrease delivery of essential fatty acids into the brain which compromises the integrity of cellular membranes, decreasing the function of membrane proteins such as glucose transporters (Passaro et al. 2015).

2: HFD promotes development of insulin resistance and glucose intolerance associated with memory deficits (Wincor and Greenwood 2005). Marked elevated insulin/IGF signaling potentiates neuronal injury (Henderson 2004). Previous studies reported that high-fat refined sugar diet decreases hippocampus BDNF (marker of VaD) (Passaro et al. 2015; Molteni et al. 2002).

HFD induced obesity is characterized by an excessive accumulation of adipose tissue (fat) and impaired adipose tissue function (Gu and Xu 2013; Chaput and Tremblay 2006). Adipose tissue dysfunction links to several health problems such as increased risk of insulin resistance, type 2 diabetes, fatty liver disease, hypertension, dyslipidemia, atherosclerosis, decreases protective activity of NO, which may be the chief source of vascular endothelium dysfunction and associated dementia (Bluher 2009; Roberts et al. 2013; Li et al. 2010).

Procedure:

Animal: Wistar Albino rats (Venkat et al. 2015)

Model: Induced by fed an HFD (Lard, Cholesterol, Casein, Sodium-cholate, Yee-sac powder, Sodium chloride, Vitamins, and minerals) for 3 months to produce cognitive deficit, can be evaluated by MWM test and series of biochemical and histopathological analysis to be performed (Parle and Singh 2007).

Table 7 showed some advantages and disadvantages of HFD-induced VaD.

3.7 Mutiple infarcts induced-VaD

Multi-infarct is also known as multiple lacunar and microinfarcts present in the cortex and in subcortical areas of variable sizes (Thal et al. 2012). It occurs due to atherosclerosis in the extracranial and intracranial vessels in the brain which give rise to thromboembolism or hypo- perfusion (Ferrer 2010; Hauw et al. 2008).

Mechanism

Cerebal ischemia is defined as shortage of oxygen and glucose in the cortex, striatum and hippocampal region of brain hemisphere, as a result failure in energy metabolism (Huang et al. 2007). ATP is essential for the regulation of pumping of ions ($Na^{+}K^{+}$ATPase and $Ca^{2+}Mg^{2+}$ATPase) to generate action potential by neurons (Erecinska and Silver 1989). These ionic pumps are very sensitive in response to small changes in ATP formation (Mrsic- Pelcic et al. 2002). Moreover, Failure in ATP formation also accompanied by generation of ROS free radicals {superoxide anion (O2-), hydroxyl (OH-) radical, and hydrogen peroxide (H_2O_2). Oxidative stress results in ischemia-induced abnormalities in rat brain and can be detected by histopathological changes. Increase in the number of glial cells, macrophages and formation of pro-inflammatory cytokines (ILs & TNF-α) damages brain tissues (Xu et al. 1999; Braughler and Hall 1989).

Procedure

Multiple infarction animal models can be induced by thromboembolic, cholesterol crystals and microspheres described as follow:-

3.7.1 Thromboembolic multiple infarction induced-VaD

In this model emboli of different sizes injecting (150–178 μm, 74–124 μm, and 48–74 μm) into ICA to produce thromboembolism infarct (Schneider et al. 2007). The infarct mainly seen in the in the cerebral cortex, hippocampus, thalamus and corpus striatum region of the brain (Kudo et al.1982).

Procedure

Animal: Male Wistar Rats; *Anaesthesia:* 10% chloral hydrate, 0.4 mL/100g. (Zhang et al. 2013). The suspension of 0.3 mL of 3% emboli (clot) is diluted with saline and then inserted into ICA (Zhang et al. 2013; Venkat et al. 2015). Clot is formed by taking specified amount of blood from femoral artery of wistar rats and then centrifuge at 4,000rpm for 10 min. The serum is discarded and remaining is to be stored at 37◦C for 72 hours into the incubator to form a clot. The blood is to be taken 36 hr before surgery so as to prevent the mortality in rats (Venkat et al. 2015). Morris water maze is employed to assess the memory and learning behavior in rats. After a certain time period, usually 60 days, histopathological analysis to be done of various

sizes of emboli in the hippocampal (CA1 and CA2-3) and cortex region (Zhang et al. 2013; Bederson et al. 1986; Venkat et al. 2015).

3.7.2 Micro-spheres induced multiple infarction VaD model

In this model, infarcts can be produced by injecting 700–900 micro-spheres of 48–50 μm in diameter into the right internal carotid artery (ICA). The brain region affected is cortex, striatum and hippocampus. (Takagi and Takeo 2003; Miyake et al. 1994)

Procedure:

Animal: Albino Wistar Rats (Zhang et al. 2013), *Anesthesia:* 35 mg/kg sodium pentobarbital i.p.

In this model, 900 microspheres suspended in 20% dextran solution is introducing into the right internal carotid artery using polyethylene catheter (3F size, 1.0 mm in diameter). Occlusions were made into the right external carotid and pterygopalatine artery. After completion of surgery, rats are disinfected with povidone-iodine solution (Venkat et al. 2015). The behavior of animal is noticed after 15 hr of surgery and histopathological examination revealed the infarct size (Zhang et al. 2013).The symptoms of stroke is scored as paucity of movement, truncal curvature, and forced circling during locomotion (Mcgraw 1977; Furlow and Bass 1976).

3.7.3 Cholesterol crystals induced multiple infarction VaD model

Animal: Albino Wistar rats (Venkat et al. 2015).

Multiple infarcts VaD in albino wistar rats can be produced by administering 300 cholesterol crystals sized 60 to 100 μm in 300 μL saline into the ICA either single, repeated and bilateral injections. The brain regions affected are cortex, striatum, hippocampus and sub cortical tissues leading to activation of microglial, macrophage and astrogliosis. All these changes in characteristics features related to VaD (Wang et al. 2012; Rapp et al. 2008). Working and reference memory can be assessed by MWM task, open field; novel object recognition test and Barnes maze test (Rapp et al. 2008). Neuronal injury, death and gliosis are constantly increase with time usually after 28 days of stroke (Wang et al. 2012)

4. Conclusion:

During the last decade a lot of progress has been made in understanding the neuropathology, mechanism and identifying genes of the vascular and cellular abnormalities concerned with vascular dementia. This review provides a detail knowledge on VaD, epidemiology, risk factors, pathophysiology, animal models mechanism, advantages and limitations. Models of vascular dementia provide better understanding of pathophysiology, mechanism, timing of disease onset, same as in humans. They signify which area of brain affected in all cases of dementia, and how vascular changes linked with cognitive deficit. Histopathological studies analyzed different lesion distinctiveness such as volume; location and neuroinflammation connect with the degree of cognitive dysfunction. We hope that this review will make possible the selection of the most suitable animal model for the better consequences and outcomes.

Authors' contributions NT, JU designed and wrote the manuscript which was revised and reviewed by BCJ and MR. All authors read and approved the final version of the manuscript.

Compliance with ethical standards

Conflict of interest The authors declare that they have no conflict of interest.

Ethical statement No humans or animals were used in this study and therefore ethical approval is not required.

Publisher's note Springer Nature remains neutral with regard to jurisdictional claims in published maps and institutional affiliations.

Acknowledgements We express our sincere thanks to Department of Pharmaceutical Sciences, Bhimtal Campus, Kumaun University Nainital, Uttarakhand, India for providing the facilities.

References

Abahji TN, Nill L, Ide N, Keller C, Hoffman U, Weiss N (2007) Acute hyperhomocysteinemia induces microvascular and macrovacsular endothelial dysfunction. Arch Med Res 38(4):411-6.

Abhar HS, Schaalan MF (2014) Phytotherapy in diabetes: Review on potential mechanistic perspectives. World J Diabetes 5(2):176-197.

Ábrahám CS, Harada N, Deli MA, Niwa M (2002) Transient forebrain ischemia increases the blood-brain barrier permeability for albumin in stroke-prone spontaneously hypertensive rats. Cellular and Molecular Neurobiology 22(4):455-462.

Ahn CS (2009) Effect of taurine supplementation on plasma homocysteine levels of the middle-aged Korean women. Adv Exp Med Biol 643(7):415-22.

Akdemira G, Rateladea J, Asavapanumasa N, Verkmana AS (2014) Neuroprotective effect of aquaporin-4 deficiency in a mouse model of severe global cerebral ischemia produced by transient 4-vessel occlusion. Neurosci Lett 574:70–75.

Alzheimer's Society, (2017) What is dementia? Retrieved from www.alzheimers.org.uk/download/downloads/id/3416/what_is_dementia.pdf.

Angelova P, Boyadjiev N (2013) A review on the models of obesity and metabolic syndrome in rats. Trakia Journal of Sciences 11(5):5-12.

Araki A (2010) Dementia and insulin resistance in patients with diabetes mellitus. Nihon Rinsho Japanese J Clin Med 68(3):569-74.

Araya R, Noguchi T, Yuhki M, Kitamura N, Higuchi M, Saido TC, Seki K, Itohara S, Kawano M, Tanemura K, Takashima A, Yamada K, Kondoh Y, Kanno I, Wess J, Yamada M, (2006) Loss of M5

muscarinic acetylcholine receptors leads to cerebrovascular and neuronal abnormalities and cognitive deficits in mice. Neurobiol Dis 24(2):334-44.

Back SA, Han BH, Luo NL, Chricton CA, Xanthoudakis S, Tam J, Arvin KL, Holtzman DM (2002) Selective vulnerability of late oligodendrocyte progenitors to hypoxia ischemia. J Neurosci 22(2):455–463.

Balakumar P, Kaur T, Singh M (2008) Potential target sites to modulate vascular endothelial dysfunction:Current perspectives and future directions. Toxicol 245(1-2): 49-64.

Balbi M, Ghosh M, Longden TA, Jativa VM, Gesierich B, Hellal F, Lourbopoulos A, Nelson MT, Plesnila N (2015) Dysfunction of mouse cerebral arteries during early aging. J Cereb Blood Flow Metab 35(9):1445-53.

Barzilai N, Huffman DM, Muzumdar RH, Bartke A (2012) The critical role of metabolic pathways in aging. Diabetes 61(6):1315-1322.

Bederson JB, Pitts LH, Tsuji M, Nishimura MC, Davis RL, Bart- kowski H (1986) Rat middle cerebral artery occlusion: evaluation of the model and development of a neurologic examination. Stroke 17(3):472–476.

Bhatia P, Gupta S, Sharma S (2014) Homocysteine excess and vascular endothelial dysfunction: Delineating the pathobiological mechanisms. Int J Pharmacol 10(4):200-212.

Bhatia P, Singh N (2015) Homocysteine excess: delineating the possible mechanism of neurotoxicity and depression. Fundam Clin Pharmacol 29(6):522-8.

Bink DI, Ritz K, Aronica E, vander Weerd L, Daemen MJ (2013) Mouse models to study the effect of cardiovascular risk factors on brain structure and cognition. J Cereb Blood Flow Metab 33(11):1666–1684.

Bleich S, Degner D, Bandelow B, Ahsen VN, Rüther E, Kornhuber J (2000) Plasma homocysteine is a predictor of alcohol withdrawal seizures. Neuroreport 11(12):2749-52.

Blüher M (2009) Adipose Tissue Dysfunction in Obesity. Exp Clin Endocrinol Diabetes 117(06):241-50.

Bockman CS, Jeffries WB, Pettinger WA, Abel PW (1992) Enhanced release of endothelium-derived relaxing factor in mineralocorticoid hypertension. Hypertension 20(3): 304-13

Boldyrev A (2010) Molecular mechanisms of homocysteine toxicity and possible protection against hyperhomocysteinemia. Recent Advances on Nutrition and the Prevention of Alzheimer's Disease 127-143.

Braughler JM, Hall ED (1989) Central nervous system trauma and stroke: I. Biochemical considerations for oxygen radical formation and lipid peroxidation. Free Radic Biol Med 6(3):289–301.

Briede J, Duburs G, in press (2007) Protective effect of cerebrocrast on rat brain ischaemia induced by occlusion of both common carotid arteries. Cell Biochem Funct 25(2):203-210.

Brosky G, Logothetopoulos J (1969) Streptozotocin diabetes in the mouse and guinea pig. Diabetes 18(9):606–611.

Brown-Borg HM, Borg KE, Meliska CJ, Bartke A (1996) Dwarf mice and the ageing process. Nature 384(6604):33.

Bruley-Rosset M, Ruley-Rosset M, Hercend T, Martinez J, Rappaport H, Mathé G (1981) Prevention of spontaneous tumors of aged mice by immunopharmacologic manipulation: study of immune antitumor mechanisms. J Natl Cancer Inst 66(6):1113- 1119.

Burke A, Burke WJ, Tariot PN (2017) Treatments for behavior and psychological symptoms in Alzheimer's disease and other dementias. Dementia 14(2):113-25.

Caldwell RB, Zhang W, Romero MJ, Caldwell RW (2010) Vascular dysfunction in retinopathy—an emerging role for arginase. Brain Res Bull 81(2-3):303–309.

Callera GE, Tostes RC, Yogi A, Montezano AC, Touyz RM (2006) Endothelin-1- induced oxidative stress in DOCA-salt hypertension involves NADPH-oxidase- independent mechanisms. Clin Sci 110(2):243–253.

Chamorro A, Hallenbeck J (2006) The harms and benefits of inflammatory and immune responses in vascular disease. Stroke 37(2):291–293.

Chan KY, Wang W, Wu JJ, Liu L, Theodoratou E, Car J, Middleton L, Russ TC, Deary IJ, Campbell H, Rudan I (2013) Epidemiology of Alzheimer's disease and other forms of dementia in China, 1990–2010: a systematic review and analysis. Lancet 381(9882):2016-2023.

Chandalia M, Abate N (2007) Metabolic complications of obesity: inflated or inflamed?. Journal of diabetes and its complications 21(2):128-136.

Chandra S, Romero MJ, Shatanawi A, Alkilany AM, Caldwell RB, Caldwell RW (2012) Oxidative species increase arginase activity in endothelial cells through the RhoA/Rho kinase pathway. Br J Pharmacol 165(2):506-519.

Chaput JP, Tremblay A (2006) Obesity at an early age and its impact on child development. Centre of Excellence for Early Childhood Developtment. Encyclopedia on Early Childhood Development 1-4.

Chen X, Touyz RM, Park JB, Schiffrin EL (2001) Antioxidant effects of vitamins C and E are associated with altered activation of vascular NADPH oxidase and superoxide dismutase in stroke-prone SHR. Hypertension 38(3):606–611.

Chiba T, Itoh T, Tabuchi M, Nakazawa T, Satou T (2012) Interleukin-1beta accelerates the onset of stroke in stroke-prone spontaneously hypertensive rats. Mediat Inflamm 2012:701976.

Chui HC (2007) Subcortical ischemia vascular dementia. Neurol Clin 25(3):717-740.

Chung EH, Iwasaki K, Mishima K, Egashira N, Fujiwara M (2002) Repeated cerebral ischemia induced hippocampal cell death and impairments of spatial cognition in the rat. Life Sci 72(4-5):609-19.

Clarke R, Bennett D, Parish S, Lewington S, Skeaff M, Eussen SJ, Lewerin C, Stott DJ, Armitage J, Hankey GJ, Lonn E, Spence JD, Galan P, de Groot LC, HalseyJ, Dangour AD, Collins R, Grodstein F (2014) Effects of homocysteine lowering with B vitamins on cognitive aging: meta-analysis of 11 trials with cognitive data on 22,000 individuals. Am J Clin Nutr 100(2):657-66.

Coffman TM (2011) Under pressure: the search for the essential mechanisms of hypertension. Nat Med 17(11):1402–1409.

Colbourne F, Li H, Buchan AM, Clemens JA (1999) Continuing postischemic neuronal death in CA1: influence of ischemia duration and cytoprotective doses of NBQX and SNX-111 in Rats. Stroke 30(3):662-8.

Dai DZ, Dai Y (2010) Role of endothelin receptor A and NADPH oxidase in vascular abnormalities. Vasc Health Risk Manag 6:787–794.

Davidson TL, Hargrave SL, Swithers SE, Sample CH, Fu X, Kinzig KP, Zheng W (2013) Inter-relationships among diet, obesity and hippocampal- dependent cognitive function. Neuroscience. 253:110–122.

Dayal S, Lentz SR (2008) Murine models of hyperhomocysteinemia and their vascular phenotypes. Arterioscler Thromb Vasc Biol 28(9):1596-1605.

de Bortoli VC, Júnior HZ, de Aguiar Corrêa FM, de Sousa Almeida S, de Oliveira AM (2005) Inhibitory avoidance memory retention in the elevated T-maze is impaired after perivascular manipulation of the common carotid arteries. Life Sci 76(18):2103-2114.

den Heijer T, Vermeer SE, Clarke R, Oudkerk M, Koudstaal PJ, Hofman A, Breteler MM (2003) Homocysteine and brain atrophy on MRI of non-demented elderly. Brain 126(1):170–175.

Dessouki EAM, Galal MA, Awad AS, Zaki HF (2017) Neuroprotective Effects of Simvastatin and Cilostazol in L-Methionine-Induced Vascular Dementia in Rats. Mol Neurobiol 54(7):5074-5084.

Dikalov SI, Dikalova AE (2016) Contribution of mitochondrial oxidative stress to hypertension. Curr Opin Nephrol Hypertens 25(2):73-80.

Doggrell SA, Brown L (1998) Rat models of hypertension, cardiac hypertrophy and failure. Cardiovascular Research 39(1):89-105.

Dua T, Barbui C, Clar N, Fleischmann A, Poznyak V, Ommeren VM, Yasamy MT, Mateo AJL, Birbeck GL, Drummond C, Freeman M, Giannakopoulos P, LevavI, Obot IS, Omigbodun O, Patel V, Phillips M, Prince M, Movaghar RA, RahmanA, Sander JW, Saunders JB, Servili C, Rangaswamy T, Unützer J, Ventevogel P, Vijayakumar L, Thornicroft G, Saxena S (2011) Evidence-based guidelines for mental, neurological, and substance use disorders in low-and middle-income countries: summary of WHO recommendations. PLoS Med 8(11):e1001122.

Duffy CM, Hofmeister JJ, Nixon JP, Butterick TA (2019) High fat diet increases cognitive decline and neuroinflammation in a model of orexin loss. Neurobiology of learning and memory 157:41-47.

Duthey B (2013) Background paper 6.11: Alzheimer disease and other dementias. A Public Health Approach to Innovation. 1-74.

Eizaguirre NO, Rementeria GP, González-Torres MÁ, Gaviria M (2017) Updates in vascular dementia. Heart and Mind 1(1):22-25.

Emdin CA, Rothwell PM, Salimi-Khorshidi G, Kiran A, Conrad N, Callender T, Mehta Z, Pendlebury ST, Anderson SG, Mohseni H, Woodward M (2016) Blood pressure and risk of vascular dementia:

evidence from a primary care registry and a cohort study of transient ischemic attack and stroke. Stroke 47(6):1429-1435.

Engelhart MJ, Geerlings MI, Meijer J, Kiliaan A, Ruitenberg A, van Swieten JC, Stijnen T, Hofman A, Witteman JC, Breteler MM (2004). Inflammatory proteins in plasma and the risk of dementia: the Rotterdam Study. Arch Neurol 61(5):668–672.

Erecinska M, Silver IA (1989) ATP and brain function. J Cereb Blood Flow Metab 9(1):2–19.

Erickson CA, Barnes CA (2003) The neurobiology of memory changes in normal aging. Exp Geronto 38(1-2):61-9.

Faraci FM, Heistad DD (1998) Regulation of the cerebral circulation: role of endothelium and potassium channels. Physiol Rev 78(1):53–97.

Faraci FM, Sigmund CD (1999) Vascular biology in genetically altered mice: smaller v essels, bigger insight. Circ Res 85(12):1214–1225.

Faraco G, Iadecola C (2013) Hypertension: a harbinger of stroke and dementia. Hypertension 62(5):810–817.

Farkas E, Luiten PG (2001) Cerebral microvascular pathologyin aging and Alzheimer's disease. Prog Neurobiol 64(6):575–61.

Farkas E, Luiten PG, Bari F (2007) Permanent, bilateral common carotid artery occlusion in the rat: a model for chronic cerebral hypo-perfusion-related neurodegenerative diseases. Brain Res Rev 54(1):162 180.

Ferrer I (2010) Cognitive impairment of vascular origin:neuropathology of cognitive impairment of vascular origin. J Neurol Sci 299(1-2):139–149.

Finkel T, Holbrook NJ (2000) Oxidants, oxidative stress and the biology of ageing. Nature 408(6809):239-247.

Flanagan M, Larson E, Latimer C, Cholerton B, Crane P, Montine K, White LR, Keene CD, Montine T (2015) Clinical-pathologic correlations in vascular cognitive impairment and dementia. Biochinica Biophysica Acta 1862(5):945–951.

Freeman LR, Keller JN (2012) Oxidative stress and cerebral endothelial cells: regulation of the blood–brain-barrier and antioxidant based interventions. Biochimica Biophysica Acta (BBA)-Molecular Basis of Disease 1822(5):822-829.

Furlow TW, Bass NH (1976) Arachidonate-induced cerebrovascular occlusion in the rat. Neurophysiology 26(4):297-3.

Furman BL (2015) Streptozotocin- induced diabetic models in mice and rats. Current protocols in pharmacology 70(5-47):1-20.

Gavras H, Brunner HR, Laragh JH, Vaughan ED Jr, Koss M, Cote LJ, Gavras I (1975) Malignant hypertension resulting from deoxycorticosterone acetate and salt excess: role of renin and sodium in vascular changes. Circ Res 36(2):300–309.

Ghiso J, Tomidokoro Y, Revesz T, Frangione B, Rostagno A (2010) Cerebral amyloid angiopathy and alzheimer’s disease. Hirosaki Igaku 61:S111–S124.

Gill R, Tsung A, Billiar T (2010) Linking oxidative stress to inflammation: toll-like receptors. Free Radic Biol Med. 48(9):1121–1132.

Gispen WH, Biessels GJ (2000) Cognition and synaptic plasticity in diabetes mellitus. Trends Neurosci 23(11):542–549.

Gocmez SS, Gacar N, Utkan T, Gacar G, Scarpace PJ, Tumer N (2016) Protective effects of resveratrol on aging-induced cognitive impairment in rats. Neurobiol Learn Mem 131:131-6.

Goldman H, Robert F, Berman T, Samuel G, Sharon M, Marilyn M, Harvey J, Altman HJ (1992) Cerebrovascular permeability and cognition in the aging rat. Neurobiol Aging 13(1):57–62.

Goldman H, Berman RF, Gershon S, Murphy SL, Altman HJ (1987) Correlation of behavioral and cerebrovascular functions in the aging rat. Neurobiol Aging 8(5):409-16.

Gong G, O'Bryant SE (2010) The arsenic exposure hypothesis for Alzheimer Disease. Alzheimer Dis Assoc Disord 24(4):311-6.

Gorelick PB, Scuteri A, Black SE, DeCarli C, Greenberg SM, Iadecola C, Launer LJ, Laurent S, Lopez OL, Nyenhuis D, Petersen RC, Schneider JA, Tzourio C, Arnett DK, Bennett DA, Chui HC, Higashida RT, Lindquist R, Nilsson PM, Roman GC, Sellke FW, Seshadri S (2011) Vascular contributions to cognitive impairment and dementia: a statement for healthcare professionals from the american heart association/american stroke association Stroke 42(9): 2672–2713.

Gu P, Xu A (2013) Interplay between adipose tissue and blood vessels in obesity and vascular dysfunction. Rev Endocr Metab Disord 14(1):49–58.

Hachinski V, Iadecola C, Petersen RC, Breteler MM, Nyenhuis DL, Black SE, Powers WJ, DeCarli C, Merino JG, Kalaria RN, Vinters HV (2006) National institute of neurological disorders and stroke–Canadian stroke network vascular cognitive impairment harmonization standards. Stroke 37(9):2220-2241.

Hainsworth AH, Markus HS (2008) Do in vivo experimental models reflect human cerebral small vessel disease? A systematic review. J Cereb Blood Flow Metab 28(12):1877-1891.

Hamel E (2004). Cholinergic modulation ofthe cortical microvascular bed.Prog Brain Res 145:171-8.

Han S, Uludag MO, Usanmaz SE, Ayaloglu-Butun F, Akcali KC, Demirel-Yilmaz E (2015) Resveratrol affects histone 3 lysine 27 methylation of vessels and blood biomarkers in DOCA salt-induced hypertension. Mol Biol Rep 42(1): 35-42.

Hattori Y, Enmi JI, Iguchi S, Saito S, Yamamoto Y, Tsuji M, Nagatsuka K, Kalaria RN, lida H, Ihara M (2016) Gradual Carotid Artery Stenosis in Mice Closely Replicates Hypoperfusive Vascular Dementia in Humans. J Am Heart Assoc 5(2):e002757.

Hauw JJ, de Girolami U, Zekry D (2008). The neuropathology of vascular and mixed dementia and vascular cognitive impairment. Handb Clin Neurol 89:687-703.

Henderson ST (2004) High carbohydrates diet and Alzheimer's disease. Med Hypotheses 62(5):689–700.

Henning EC, Warach S, Spatz M (2010) Hypertension-induced vascular remodeling contributes to reduced cerebral perfusion and the development of spontaneous stroke in aged SHRSP rats. J Cereb Blood Flow Metab 30(4):827–836.

Hobbenaghi R, Javanbakht J, Sadeghzadeh S, Kheradmand D, Abdi FS, Jaberi MH, Mohammadiyan MR, Khadivar F, Mollaei Y (2014) Neuroprotective effects of Nigella sativa extract on cell death in hippocampal neurons following experimental global cerebral ischemia-reperfusion injury in rats. J Neurol Sci 337(1-2):74-79.

Huang JL, Fu ST, Jiang YY, Cao YB, Guo ML, Wang Y, Xu Z (2007) Protective effects of Nicotiflorin on reducingmemory dysfunction, energy metabolism failure and oxidative stress in multi-infarct dementia model rats. Pharmacol Biochem Behav 86(4): 741–748.

Iadecola C (2013) The Pathobiology of vascular dementia. Neuron 80(4):844–866.

Ikram MA, Bersano A, Manso-Calderón R, Jia JP, Schmidt H, Middleton L, Nacmias B, Siddiqi S, Adams HH (2017) Genetics of vascular dementia–review from the ICVD working group. BMC Med 15(1): 48.

Jakubowski H (2006) Pathophysiological consequences of homocysteine excess. J Nut 136(6): 1741S-1749S.

Jalal FY,Yang Y, Thompson J, Lopez AC, Rosenberg GA (2012) Myelin loss associated with neuroinflammation in hypertensive rats. Stroke 43(4):1115–22.

Jennifer G,Donna MW (2016) Animal Models of Vascular Cognitive Impairment and Dementia (VCID). Cell Mol Neurobiol 36(2):233-9.

Jimenez R, Lopez-Sepulveda R, Kadmiri M, Romero M, Vera R, Sanchez, M, Vargas F, O'Valle F, Zarzuelo A, Dueñas M, Santos-Buelga C, Duarte J (2007) Polyphenols restore endothelial function in DOCA-salt hypertension: role of endothelin-1 and NADPH oxidase. Free Radic Biol Med 43(3):462–473.

Jiwa NS, Garrard P, Hainsworth AH (2010) Experimental models of vascular dementia and vascular cognitive impairment: a systematic review. J Neurochem 115(4):814-28.

Kalaria RN (2016) Neuropathological diagnosis of vascular cognitive impairment and vascular dementia with implications for Alzheimer's disease. Acta Neuropathol 131(5):659-85.

Kalaria RN, Maestre GE, Arizaga R, Friedland RP, Galasko D, Hall K, Luchsinger JA, Ogunniyi A, Perry EK, Potocnik F, Prince M, Stewart R, Wimo A, Zhang ZX, Antuono P (2008) Alzheimer's disease and vascular dementia in developing countries: prevalence, management, and risk factors. Lancet Neurol 7(9): 812-826.

Kalmijn S, Foley D, White L, Burchfiel CM, Curb JD, Petrovitch H, Ross GW, Havlik RJ, Launer LJ (2000) Metabolic cardiovascular syndrome and risk of dementia in Japanese-American elderly men: the Honolulu-Asia Aging Study. Arteriosclerosis, thrombosis, and vascular biology 20(10):2255-2260.

Kamat PK, Vacek JC, Kalani A, Tyagi N (2015) Homocysteine Induced Cerebrovascular Dysfunction: A Link to Alzheimer's Disease Etiology. Open Neurol 9:9-14.

Kastner RS, Aguirre-Chen C, Saul I, Yick L, Hamasaki D, Busto R, Ginsberg MD (2005) Astrocytes react to oligemia in the forebrain induced by chronic bilateral common carotid artery occlusion in rats . Brain Res 1052(1):28 – 39.

Kaundal M, Zameer S, Najmi AK, Parvez S, Akhtar M (2018) Betulinic acid, a natural PDE inhibitor restores hippocampal cAMP/cGMP and BDNF, improve cerebral blood flow and recover memory deficits in permanent BCCAO induced vascular dementia in rats. Eur J Pharmacol 832: 56-66.

Kaur T, Goel RK, Balakumar P (2010) Effect of rosiglitazone in sodium arsenite- induced experimental vascular endothelial dysfunction. Arch. Pharm Res 33(4):611-8.

Kempsell AT, Fieber LA (2015) Age-related deficits in synaptic plasticity rescued by activating PKA or PKC in sensory neurons of Aplysia californica. Front Aging Neurosci 7:173.

Kesavan M, Sarath,TS, Kannan K, Suresh S, Gupta P, Vijayakaran K, Sankar P, Kurade NP, Mishra SK, Sarkar SN (2014) Atorvastatin restores arsenic-induced vascular dysfunction in rats: modulation of nitric oxide signaling and inflammatory mediators. Toxicol Appl Pharmacol 280(1): 107-116.

Kimura S, Saito H, Minami M, Togashi H, Nakamura N, Nemoto M, Parvez HS (2000) Pathogenesis of vascular dementia in stroke-prone spontaneously hypertensive rats. Toxicology 153(1-3):167-178.

Kitamura A, Fujita Y, Oishi N, Kalaria RN, Washida K, Maki T, Okamoto Y, Hase Y, Yamada M, Takahashi J, Ito H, Tomimoto H, Fukuyama H, Takahashi R, Ihara M (2012) Selective white matter abnormalities in a novel rat modelof vascular dementia. Neurobiol Aging 33(5):1012-e25.

Kling MA, Trojanowski JQ, Wolk DA, Lee VM, Arnold SE (2013) Vascular disease and dementias: paradigm shifts to drive research in new directions. Alzheimers Dement 9(1):76-92.

Koladiya RU, Jaggi AS, Singh N, Sharma BK (2008) Ameliorative role of Atorvastatin and Pitavastatin in L-Methionine induced vascular dementia in rats. BMC Pharmacol 8(1):14.

Kolb H (1987) Mouse models of insulin dependent diabetes: Low-dose streptozocin-induced diabetes and nonobese diabetic (NOD) mice. Diabetes Metab Rev 3(3):751-778.

Korczyn AD (2016) What is new in vascular dementia?. BMC Med 14:175.

Kubota Y, Umegaki K, Kagota S, Tanaka N, Nakamura K, Kunitomo M, Shinozuka K (2006) Evaluation of blood pressure measured by tail-cuff methods (without heating) in spontaneously hypertensive rats. Biol Pharm Bull 29(8):1756-8.

Kudo M, Aoyama A, Ichimori S, Fukunaga N (1982) An animal model of cerebral infarction. Homologous blood clot emboli in rats. Stroke 13(4):505–508.

Kudo T, Takeda M, Tanimukai S, Nishimura T (1993) Neuropathologic changes in the gerbil brain after chronic hypoperfusion. Stroke 24(2):259-264.

Lacy F, Gough DA, Schmid-Schonbein GW (1998) Role of xanthine oxidase in hydrogen peroxide production. Free Radic Biol Med 25(6):720–727.

Lee H, Kim JM, Kim HJ, Lee I, Chang, N (2005) Folic acid supplementation can reduce the endothelial damage in rat brain microvasculature due to hyperhomocysteinemia. J Nutr 135(3):544-8.

Lentz SR, Haynes WG (2004) Homocysteine: is it a clinically important cardiovascular risk factor?. Cleve Clin J Med 71(9):729-34.

Li FY, Cheng KK, Lam KS, Vanhoutte PM, Xu A (2010) Cross-talk between adipose tissue and vasculature: role of adiponectin. Acta Physiol 203(1):167–80.

Li Z, Pang L, Fang F, Zhang G, Zhang J, Xie M, Wang L (2012) Resveratrol attenuates brain damage in a rat model of focal cerebral ischemia via up- regulation of hippocampal Bcl-2. Brain Res 1450:116–124.

Li WZ.,Wu WY, Huang H, Wu YY, Yin YY (2013) Protective effect of bilobalide on learning andmemory impairment in rats with vascular dementia. Mol Med Rep 8(3):935–941.

Like AA, Rossini AA (1976) Streptozotocin induced pancreatic insulitis: New model of diabetes mellitus. Science 193(4215):415-417.

Lindner MD, Gribkoff VK, Donlan NA, Jones TA (2003) Long-lasting functional disabilities in middle-aged rats with small cerebral infarcts. J Neurosci 23(34):10913–10922.

Liu H, Zhang J (2012) Cerebral hypoperfusion and cognitive impairment: the pathogenic role of vascular oxidative stress. Int J Neurosci 122(9):494–499.

Liu J, Liu WB, Ji XT, Fei Z, Cheng G (2012) One-stage apertura thoracis superior approach for four-vessel occlusion in rats. Chin J Traumatol 15(1):13-16.

Liu LL, Yan L, Chen YH, Zeng GH, Zhou Y, Chen HP, Peng WJ, He M, Huang QR (2014) A role for diallyl trisulfide in mitochondrial antioxidative stress contributes to its protective effects against vascular endothelial impairment. Eur J Pharmacol 725:23– 31.

Liu T, Zhang L, Joo D, Sun SC (2017) NF-κB signaling in inflammation Signal Transduct Target Ther 2:17023.

Luca M, Luca A, Calandra C (2015) The role of oxidative damage in the pathogenesis and progression of Alzheimer's disease and vascular dementia. Oxidative medicine and cellular longevity 2015:504678.

Luitse MJ, Biessels GJ, Rutten GE, Kappelle LJ (2012) Diabetes, hyperglycaemia, and acute ischemia stroke. Lancet Neurol 11(3):261–271.

Machado FR, Ferreira AG, da Cunha AA, Tagliari B, Mussulini BH, Wofchuk S, Wyse AT (2011) Homocysteine alters glutamate uptake and Na+, K+-ATPase activity and oxidative status in rats hippocampus: protection by vitamin C. Metab Brain Dis 26(1):61-67.

Mallia BAM, Barbara C, Rao R (2018) Vascular cognitive impairment and vascular dementia. InnovAiT 11(5):249-255.

Manwani B, Friedler B, Verma R, Venna VR, Mc Cullough LD, Liu F (2014) Perfusion of ischemic brain in young and aged animals: a laser speckle flowmetry study. Stroke 45(2):571–578.

Marshall RS (2012) Effects of altered cerebral hemodynamics on cognitive function. J Alzheimers Dis 32(3):633–642.

Matin N, Fisher C, Jackson WF, Dorrance AM (2016) Bilateral common carotid artery stenosis in normotensive rats impairs endothelium-dependent dilation of parenchymal arterioles. Am J Physiol Heart Circ Physiol 310(10):H1321-9.

McGraw CP (1977) Experimental cerebral infarction: Effect of pentobar-bital in mongolian gerbils. Arch Neurol 34(6):334-336.

Minami M, Kimura S, Endo T, Hamaue N, Hirafuji M, Togashi H, Matsumoto M, Yoshioka M, Saito H, Watanabe S, Kobayashi T (1997) Dietary docosahexaenoic acid increases cerebral acetylcholine levels and improves passive avoidance performance in stroke-prone spontaneously hypertensive rats. Pharmacology Biochemistry and Behavior 58(4):1123-1129.

Miyake K, Takagi N, Takeo S (1994) Effects of naftidrofuryl oxalate on microsphere embolism induced decrease in regiona lblood flow of rat brain. Br J Pharmacol 112(1):226-30.

Mizuno T (2015) Sub cortical ischemic vascular dementia: lesson from hereditary cerebral small vessel disease. Neurology Brain and Nerve 67(4):403-412.

Molteni R, Barnard RJ, Ying Z, Roberts CK, Gomez-Pinilla F (2002) A high-fat, refined sugar diet reduces hippocampal brain-derived neurotrophic factor, neuronal plasticity, and learning. Neuroscience 112(4):803–814.

Morris R (1984) Developments of a water-maze procedure for studying spatial learning in the rat.J Neurosci 11(1):47–60.

Mrsic-Pelcic J, Zupan G, Maysinger D, Pelcic G, Vitezic D, Simonic A (2002) The influence of MK-801 on the hippocampal free arachidonic acid level and Na+K+ATPase activity in global cerebral ischemia-exposed rats. Prog Neuro-psychopharmacol Biol Psychiatry 26(7-8):1319–26.

Murtishaw AS, Heaney CF, Bolton MM, Belmonte KCD, Langhardt MA, Kinney JW, (2018) Intermittent streptozotocin administration induces behavioral and pathological features relevant to Alzheimer's disease and vascular dementia. Neuropharmaco 137:164-177.

Nakaji K, Ihara M, Takahashi C, Itohara S, Noda M, Takahashi R, Tomomoto H (2006) Matrix metalloproteinase-2 plays a critical role in the pathogenesis of white matter lesions after chronic cerebral hypoperfusion in rodents. Stroke 37(11): 2816−2823.

S Neha, Jaggi RK, Singh N (2014) Animal models of dementia and cognitive dysfunction. Life Sci 109(2):73-86.

Nishio K, Ihara M, Yamasaki N, Kalaria RN, Maki T, Fujita Y, Ito H, Oishi N, Fukuyama H, Miyakawa T, Takahashi R, Tomimoto H (2010) A mouse model characterizing features of vascular dementia with hippocampal atrophy. Stroke 41(6):1278- 84.

Nita DA, Nita V, Spulber S, Moldovan M, Popa DP, Zagrean AM, Zagrean L (2001) Oxidative damage following cerebral ischemia depends on reperfusion-a biochemical study in rat. J Cell Mol Med 5(2):163–170.

Obeid R, Herrmann W (2006) Mechanisms of homocysteine neurotoxicity in neurodegenerative diseases with special reference to dementia. FEBS Lett 580(13):2994- 3005.

Osborne NN, Chidlow G, Nash MS, Wood JP (1999) The potential of neuroprotection in glaucoma treatment. Curr Opin Ophthalmol 10(2):82–92.

Ostrowski RP, Colohan AR, Zhang JH (2005) Mechanisms of hyperbaric oxygen-induced neuroprotection in a rat model of subarachnoid hemorrhage. J Cereb Blood Flow Metab 25(5):554-571.

Papadopoulos MC, Koumenis IL, Yuan TY, Giffard RG (1998) Increasing vulnerability of astrocytes to oxidative injury with age despite constant antioxidant defenses. Neuroscience 82(3): 915–25.

Park L, Josef A, Helene G, Ping Z, Costantino I (2007) Nox2-derived reactive oxygen species mediate neurovascular dysregulation in the aging mouse Brain. J Cerebr Blood Flow Metab 27(12):1908–1918

Parle M, Singh N (2004) Animal models for testing memory. Asia Pac J Pharmacol 16:101 102.

Parle M, Singh N (2007) Reversal of memory deficits by Atorvastatin and Simvastatin in rats.Yakugaku Zasshi. 127(7):1125–37.

Pasquier F, Boulogne A, Leys D, Fontaine P (2006) Diabetes mellitus and dementia. Diabetes Metab 32(5):403-414.

Passaro A, Dalla Nora E, Morieri ML, Soavi C, Sanz JM, Zurlo A, Fellin R, Zuliani G (2015) Brain-derived neurotrophic factor plasma levels: relationship with dementia and diabetes in the elderly population. J Gerontol A Biol Sci Med Sci 70(3):294-302.

Patel A, Moalem A, Cheng H, Babadjouni RM, Patel K, Hodis DM, Chandegara D, Cen S, He S, Liu Q, Mack WJ (2017) Chronic cerebral hypoperfusion induced by bilateral carotid artery stenosis causes selective recognition impairment in adult mice. Neurol Res 39(10):910-917.

Petito CK,Olarte JP, Roberts B, Nowak TS, Pulsinelli WA(1998) Selective glial vulnerability following transient global ischemia in rat brain. J Neuropathol Exp Neuro 57(3):231-238.

Pickup JC, Crook MA (1998) Is type II diabetes mellitus a disease of the innate immune system? Diabetologia 41(10):1241–1248.

Pieper GM (1997) Diabetic induced endothelial dysfunction in rat aorta: role of hydroxyl radicals. Cardiovasc Res 34(1):145-56.

Plaschke K (2005) Aspects of ageing in chronic cerebral oligaemia. Mechanisms of degeneration and compensation in rat models. J Neural Transm 112(3):393–413.

Prince M, Ali GC, Guerchet M, Prina AM, Albanese E, Yu-Tzu Wu YT (2016) Recent global trends in the prevalence and incidence of dementia, and survival with dementia. Alzheimers Res Ther. 8(1):23.

Rader DJ (2000) Inflammatory markers of coronary risk. N Engl J Med 343(16):1179–1182.

Ramin A, Mahta A, Mallack E, Luo JJ (2014) Homocysteinemia and neurological disorders: a Review. J Clin Neurol 10(4):281-288.

Ranjithkumar R, Premnath P, Ramanathan M (2015) Measurement of Inflammatory Mediators at Different Time Intervals after Neuronal Injury Induced by Bilateral Common Carotid Artery Occlusion Model. J Pharm Sci. & Res 7(9):662-667.

Rapp JH, Pan XM, Neumann M, Hong M, Hollenbeck K, Liu J (2008) Microemboli composed of cholesterol crystals disrupt the blood-brain barrier and reduce cognition. Stroke 39(8):2354-2361.

Ratnatunge SS, Silva DVA (2015) Management of dementia: review of evidence. J Ceylon College Physicians 46:24-28.

Regier NG, Gitlin LN (2017) Towards defining restlessness in individuals with dementia. Aging Men Health 21(5):543-552.

Rizzi L, Rosset I, Roriz-Cruz M (2014) Global epidemiology of dementia: Alzheimer’s and vascular types. Biomed Res Int 8:908915.

Roberts CK, Hevener AL, Barnard R.J (2013) Metabolic Syndrome and Insulin Resistance: Underlying Causes and Modification by Exercise Training. Compr Physiol 3(1): 1–58.

Roberts JM, Maniskas ME, Bix GJ (2018) Bilateral carotid artery stenosis causes unexpected early changes in brain extracellular matrix and blood-brain barrier integrity in mice. PloS one 13(4):e0195765.

Romero MJ, Platt DH, Tawfik HE, Labazi M, El-Remessy AB, Bartoli M, Caldwell RB, Caldwell RW (2008) Diabetes-induced coronary vascular dysfunction involves increased arginase activity. Circ Res 102(1):95-102.

Rothwell PM, Coull AJ, Silver LE, Fairhead JF, Giles MF, Lovelock CE, Redgrave JN, Bull LM, Welch SJ, Cuthbertson FC, Binney LE, Gutnikov SA, Anslow P, Banning AP, Mant D, Mehta Z (2005) Population-based study of event-rate, incidence, case fatality, and mortality for all acute vascular events in all arterial territories (Oxford Vascular Study). Lancet 366(9499):1773–1783.

Rufa A, Malandrini A, Dotti MT, Berti G, Salvadori C, Federico A (2005) Typical pathological changes of CADASIL in the optic nerve. Neurol Sci 26(4):271–4.

Sain H, Sharma B, Jaggi AS, Singh N (2011) Pharmacological investigations on potential of peroxisome proliferator-activated receptor-gamma agonists in hyperhomocysteinemia-induced vascular dementia in rats. Neuroscience 192:322–33.

Sathe S, Beninati E, Raz E, Inglese M (2015) Development of lacune:what do we find in CADASIL?. Neurology 84(14):P2-255.

Schneider JA, Arvanitakis Z, Bang W, Bennett DA (2007) Mixed brain pathologies account for most dementia cases in community-dwelling older persons. Neurol 69(24):2197–2204.

Schreiber S, Bueche CZ, Garz C, Braun H (2013) Blood brain barrier breakdown as the starting point of cerebral small vessel disease? - new insights from a rat model. Exp Transl Stroke Med. 5(1):4.

Scuteri A, Tesauro M, Guglini L, Lauro D, Fini M, Di Daniele N (2013) Aortic stiffness and hypotension episodes are associated with impaired cognitive function in older subjects with subjective complaints of memory loss. Int J Cardiol 169(5):371–7.

Shah DI, Singh M (2007) Possible role of Akt to improve vascular endothelial dysfunction in diabetic and hyperhomocysteinemic rats. Mol Cell Biochem 295(1-2):65-74.

Shaik S, Zhiwei W, Hiroyuki I, Pengda L, Wenyi W (2013) Endothelium Aging and Vascular Diseases. In Tech 1-20.

Sharma B, Sharma PM (2013) Arsenic toxicity induced endothelial dysfunction and dementia: pharmacological interdiction by histone deacetylases and inducible nitric oxide synthase inhibitors. Toxicol Appl Pharmacol 273(1):180-188.

Sharma B, Singh N (2010) Pitavastatin and 4′-hydroxy-3′-methoxyacetophenone (HMAP) reduce cognitive dysfunction in vascular dementia during experimental diabetes. Curr Neurovasc Res 7(3):180–191.

Sharma B, Singh N (2011) Attenuation of vascular dementia by sodium butyrate in streptozotocin diabetic rats. Psychopharmacol 215(4):677–687.

Sharma B, Singh N (2012) Defensive effect of natrium diethyldithiocarbamate trihydrate (NDDCT) and lisinopril in DOCA-salt hypertension-induced vascular dementia in rats. Psychopharmacology 223(3):307-317.

Shi H, Shi X, Liu KJ (2004) Oxidative mechanism of arsenic toxicity and carcinogenesis. Mol Cell Biochem 255(1-2):67-78.

Shibata M, Ohtani R, Ihara M, Tomimoto H (2004) White matter lesions and glial activation in a novel mouse model chronic cerebral hypoperfusion. Stroke 35(11):2598–2603.

Shibata M, Yamasaki N, Miyakawa T, Kalaria RN, Fujita Y, Ohtani R, Ihara M, Takahashi R, Tomimoto H (2007) Selective impairment of working memory in a mouse model of chronic cerebral hypoperfusion. Stroke 38(10):2826–2832.

Sierra C, Domenech M, Camafort M, Coca A (2012) Hypertension and mild cognitive impairment. Curr Hypertens Rep 14(6):548–55.

Sinclair DA, Guarente L (2006) Unlocking the secrets of longevity genes. Sci Am 294(3): 54- 47.

Stasiak A, Mussur M, Unzeta M, Samadi AL, Marco-Contelles J, Agnieszka Fogel W (2014) Effects of novel monoamine oxidases and cholinesterases targeting compounds on brain neurotransmitters and behavior in rat model of vascular dementia. Curr Pharm Des 20(2):161-171.

Stevens WD, Fortin T, Pappas BA (2002) Retinal and optic nerve degeneration after chronic carotid ligation: time course and role of light exposure. Stroke 33(4):1107–1112.

Streck EL, Delwing D, Tagliari B, Matté C, Wannmacher CM, Wajner M, Wyse AT (2003). Brain energy metabolism is compromised by the metabolites accumulating in homocystinuria. Neurochem Int 43(6):597-602.

Sudduth TL, Powell DK, Smith CD, Greenstein A, Wilcock DM (2013) Induction of hyperhomocysteinemia models vascular dementia by induction of cerebral microhemorrhages and neuroinflammation. J Cereb Blood Flow Metab 33(5):708-15.

Suzuki H, DeLano FA, Parks DA, Jamshidi N, Granger DN, Ishii H, Suematsu M, Zweifach BW, Schmid-Schönbein GW (1998) Xanthine oxidase activity associated with arterial blood pressure in spontaneously hypertensive rats. Proc Natl Acad Sci 95(8):4754-4759.

Takagi K, Takeo S (2003) The model of stroke induced by microsphere embolism in rats. Nihon Yakurigaku Zasshi. Folia pharmacologica Japonica.121(6): 440-6.

Taye A, Saad AH, Kumar AH, Morawietz H (2010) Effect of apocynin on NADPH oxidase-mediated oxidative stress-LOX-1-eNOS pathway in human endothelial cells exposed to high glucose. Eur J Pharmacol 627(1-3):42–48.

Thal DR, Grinberg LT, Attems J (2012) Vascular dementia: different forms of vessel disorders contribute to the development of dementia in the elderly brain. Exp Gerontol. 47(11):816-824.

Tikka S, Baumann M, Siitonen M, Pasanen P, Pöyhönen M, Myllykangas L, Viitanen M, Fukutake T, Cognat E, Joutel A, Kalimo H (2014) Cadasil and Carasil. Brain Pathology 24(5):525-544.

Tiwari N, Bhatia P, Kumar A, Jaggi AS, Singh N (2018) Potential of carnosine, a histamine precursor in rat model of bilateral common carotid artery occlusion induced vascular dementia. Fundam Clin Pharmacol 32(5):516-531.

Toledo JB, Arnold SE, Raible K, Brettschneider J, Xie SX, Grossman M, Monsell SE, Kukull WA, Trojanowski JQ (2013) Contribution of cerebrovascular disease in autopsy confirmed neurodegenerative disease cases in the National Alzheimer's Coordinating Centre. Brain 136(9): 2697-2706.

Toshima Y, Satoh S, Ikegaki I, Asano T (2000) A new model of cerebral microthrombosis in rats and the neuroprotective effect of a Rho-kinase inhibitor. Stroke 31(9): 2245-49.

Tournier LE, Joutel A, Melki J, Weissenbach J, Lathrop GM, Chabriat H,Mas JL, Cabanis EA, Baudrimont M, Maciazek J, Bach MA (1993) Cerebral autosomal dominant arteriopathy with subcortical infarcts and leukoencephalopathy maps to chromosome 19q12. NatGenet 3(3):256-9.

Tousoulis D, Antoniades C, Tentolouris C, Goumas G, Stefanadis C, Toutouzas P (2002) L-arginine in cardiovascular disease: dream or reality?. Vasc Med 7(3):203–211.

Triantafyllidi H, Arvaniti C, Lekakis J, Ikonomidis I, Siafakas N, Tzortzis S, Trivilou P, ZervaL, Stamboulis E, Kremastinos DT (2009) Cognitive impairment is related to increased arterial stiffness and microvascular damage in patients with never-treated essential hypertension. Am J Hypertens 22(5):525–30.

Tsou TC, Tsai FY, Hsieh YW, Li LA, Yeh SC, Chang LW (2005) Arsenite induces endothelial cytotoxicity by downregulation of vascular endothelial nitric oxide synthase. Toxicol Appl Pharmacol 208(8):277-284.

Ueno M, Tomimoto H, Akiguchi I, Wakita H, Sakamoto H (2002) Blood– brain barrier disruption in white matter lesions in a rat model of chronic cerebral hypoperfusion. J Cereb Blood Flow Metab 22(1):97–104.

Uluç K, Miranpuri A, Kujoth GC, Aktüre E, Başkaya MK (2011) Focal cerebral ischemia model by Endovascular Suture Occlusion of the Middle Cerebral Artery in the Rat. J Vis Exp 5(48):1978.

vander Flier WM., Scheltens P (2005) Epidemiology and risk factors of dementia. J Neurol Neurosurg Psychiatry 76(5):v2-v7.

Venkat P, Chopp M, Chen J (2015) Models and mechanism of vascular dementia. Exp Neurol. 272:97-108.

Versari D, Daghini E, Virdis A, Ghiadoni L, Taddei S (2009) Endothelial dysfunction as a target for prevention of cardiovascular Disease. Diabetes Care 32(2):314-321.

Viel EC, Benkirane K, Javeshghani D, Touyz RM, Schiffrin EL (2008) Xanthine oxidase and mitochondria contribute to vascular superoxide anion generation in DOCA-salt hypertensive rats. Am J Physiol Heart Circ Physiol 295(1):H281–288.

Vinters HV (1987) Cerebral amyloid angiopathy. A critical review. Stroke 18(2):311-324.

Vinters HV, Wang ZZ, Secor DL (1996) Brain parenchymal and microvascular amyloid in Alzheimer's disease. Brain Pathol 6(2):179–195.

Wada-Isoe K, Wakutani Y, Urakami K, Nakashima K (2004) Elevated interleukin-6 levels in cerebrospinal fluid of vascular dementia patients. Acta Neurol Scand 110(2):124–127.

Wakita H, Tomimoto H, Akiguchi I, Kimura J (1994) Glial activation and white matter changes in the rat brain induced by chronic cerebral hypoperfusion: an immunohistochemical study. Acta Neuropathologica 87(5):484-492.

Walker EJ, Rosenberg GA (2010) Divergent role for MMP-2 in myelin breakdown and oligodendrocyte death following transient global ischemia. J Neurosci Res 88(4):764-773.

Wallin A, Román GC, Esiri M, Kettunen P, Svensson J, Paraskevas GP, Kapaki E (2018) Update on Vascular Cognitive Impairment Associated with Subcortical Small-Vessel Disease. J Alzheimers Dis 62(3):1417-1441.

Wan H (2014) Establishment of an animal model of vascular dementia. Exp Ther Med 8(5): 1599-1603.

Wang M, Iliff JJ, Liao Y, Chen MJ, Shinseki MS, Venkataraman A, Cheung J, Wang W, Nedergaard M (2012) Cognitive deficits and delayed neuronal loss in a mouse model of multiple microinfarcts. J Neurosci 32(50):7948-60.

Wilson CJ, Finch CE,Cohen HJ (2002) Cytokines and cognition the case for a head-to- toe inflammatory paradigm. J Am Geriatr Soc 50(12):2041–2056.

Wimo A, Jonsson L, Bond J, Prince M, Winblad B (2013) The worldwide economic impact of dementia 2010. Alzheimers Dement 9(1):1-11.

Winocur G, Greenwood CE (2005) Studies of the effects of high fat diets on cognitive function in a rat model. Neurobiol Aging 1:46–49.

Wood JG, Rogina B, Lavu S, Howitz K,Helfand SL,Tatar M, Sinclair D (2004) Sirtuin activators mimic caloric restriction and delay ageing in metazoans. Nature 430(7000):686-689.

Wu KK, Huan Y (2008) Streptozotocin-induced diabetic models in mice and rats. Curr Protoc Pharmacol 5(5):47.

Wu R, Millette E, Wu L, de Champlain J (2001) Enhanced superoxide anion formation in vascular tissues from spontaneously hypertensive and desoxycorticoster one acetate-salt hypertensive rats. J Hypertens 19(4):741–48.

Xi Y, Wang M, Zhang W, Bai M, Du Y, Zhang Z, Li Z, Miao J (2014) Neuronal damage, central cholinergic dysfunction and oxidative damage correlate with cognitive deficits in rats with chronic cerebral hypoperfusion. Neurobiol Learn Mem 109:7-19.

Xu H, Shao N, Zhang M (1999) Experimental study on multi-infarct dementia treated with reinforcing essence to refresh mental activity method. Chin J Integr Med 19(6):359–62.

Yadav RS, Chandravanshi LP, Shukla RK, Sankhwar WL, Ansari RW, Shukla PK, Pant AB, Khanna VK (2011) Neuroprotective efficacy of curcumin in arsenic induced cholinergic dysfunction in rats. Neurotoxicol 32(6):760-768.

Yamada M, Lamping KG, Duttaroy A, Zhang W, Cui Y, Bymaster FP, McKinzie DL, Felder CC, Deng CX, Faraci FM, Wess J (2001) Cholinergic dilation of cerebral blood vessels is abolished in M(5) muscarinic acetylcholine receptor knockout mice. Proc Natl Acad Sci 98(24):14096-101.

Yamada M (2015) Cerebral Amyloid Angiopathy: Emerging Concepts. J Stroke 17(1):17-30.

Yamaguchi M, Calvert JW, Kusaka G, Zhang JH (2005) One-stage anterior approach for four vessel occlusion in rat. Stroke 36(10):2212-2214.

Yamori Y, Horie R, Handa H, Sato M, Fukase M (1976) Pathogenetic similarity of strokes in stroke-prone spontaneously hypertensive rats and humans. Stroke 7(1):46–53.

Yoshizaki K, Adachi K, Kataoka S, Watanabe A, Tabira T, Takahashi K, Wakita H (2008) Chronic cerebral hypoperfusion induced by right unilateral common carotid artery occlusion causes delayed white matter lesions and cognitive impairment in adult mice. Exp Neurol 210(2):585-591.

Zhang C, Hein TW, Wang W, Miller MW, Fossum TW, McDonald MM, Humphrey JD, Kuo L (2004) Upregulation of vascular arginase in hypertension decreases nitric oxide−mediated dilation of coronary arterioles. Hypertension 44(6):935-943.

Zhang HA, Gao M, Chen B, Shi L, Wang Q, Yu X, Xuan Z, Gao L, Du G (2013). Evaluation of hippocampal injury and cognitive function induced by embolization in the rat brain. Anat Rec (Hoboken) 296(8):1207-14.

Zhang Y, Shi Z, Liu M, Liu S, Yue W, Liu S, Xiang L, Lu H, Liu P, Wisniewski T, Wang J, (2014) Prevalence of cognitive impairment no dementia in a rural area of Northern China. Neuroepidemiology 42(4):197-203.

Zhao F, Yuan J, Lu G, Zhang LH, Chen ZY, Wáng YXJ (2016) T1ρ relaxation time in brain regions increases with ageing: an experimental MRI observation in rats. Br J Radiol 89(1057):20140704.

Figure caption:

Figure 1: Risk factors and characteristics features of VaD

Figure 2: Mechanistic pathway for the pathogenesis of VaD

Figure 3: Mechanism of cerebral dysfunction (Vessel occlusion model)

Table legends:

Table 1: Experimental models of vascular dementia

Table 2: Some of the advantages and limitations of vessel occlusion models

Table 3: Advantages and disadvantages if hypertension induced VaD

Table 4: Advantages and limitations of STZ induced VaD model

Table 5: Advantages and Disadvantages of hyper-homocystenemia induced VaD

Table 6: Advantages and Limitations of Age-induced VaD

Table 7: Advantages and Disadvantages of High-fat diet-induced VaD

Table 8: Advantages and Limitations of Multiple infarct model of VaD

Click here to access/download;Figure;Figure 1.jpg

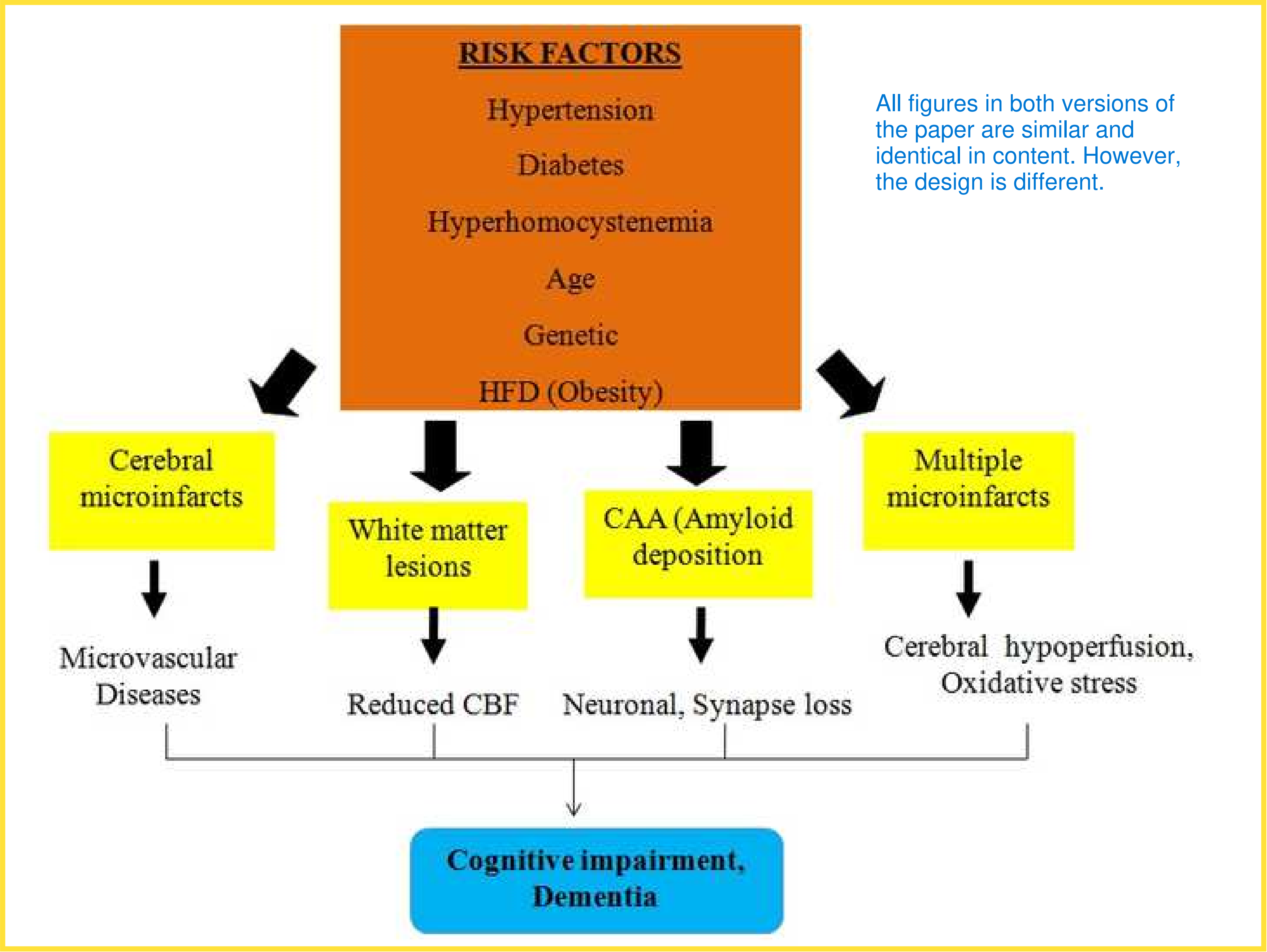

All figures in both versions of the paper are similar and identical in content. However, the design is different.

Click here to access/download;Figure;Figure 2.jpg

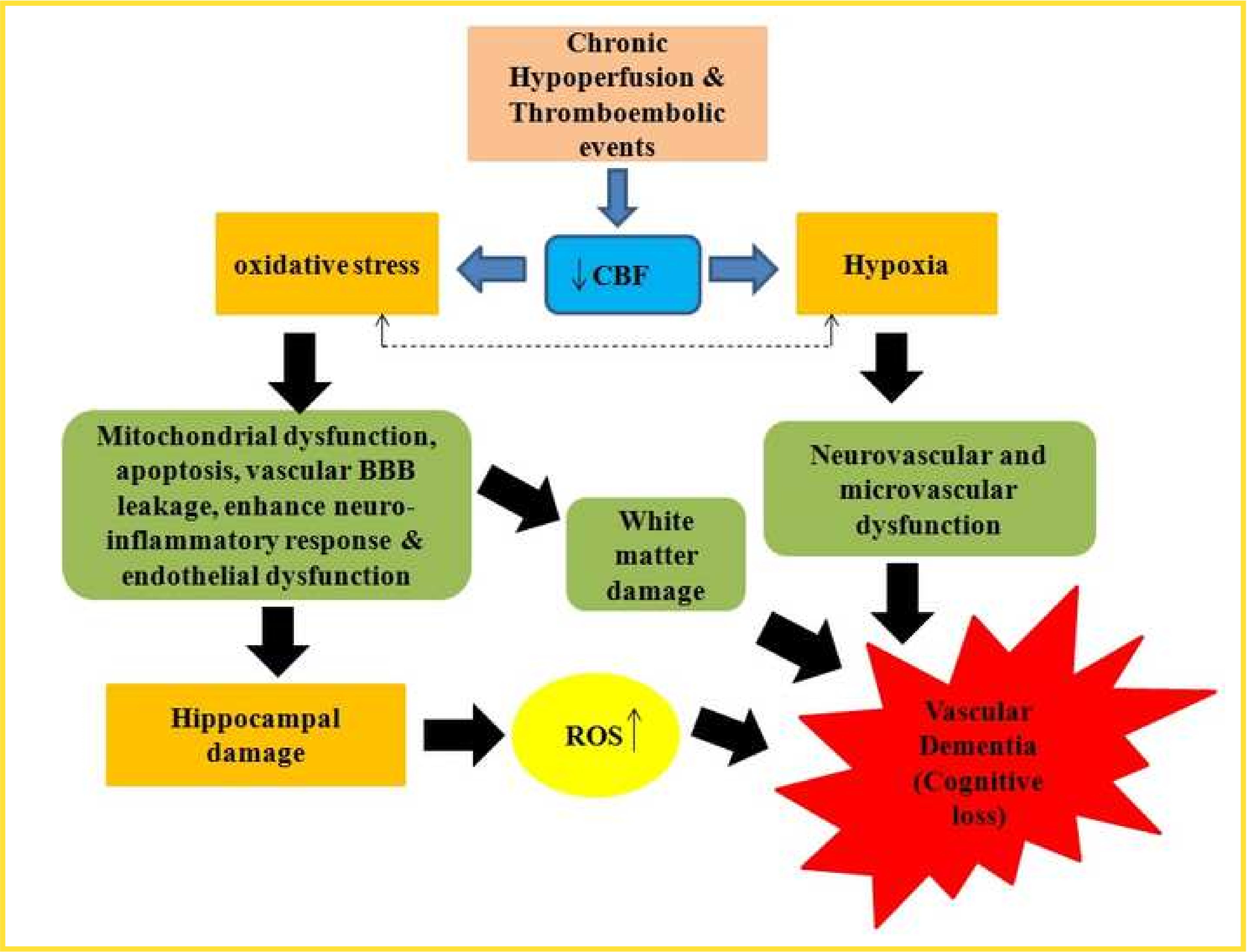

 Click here to access/download;Figure;Figure 3.jpg

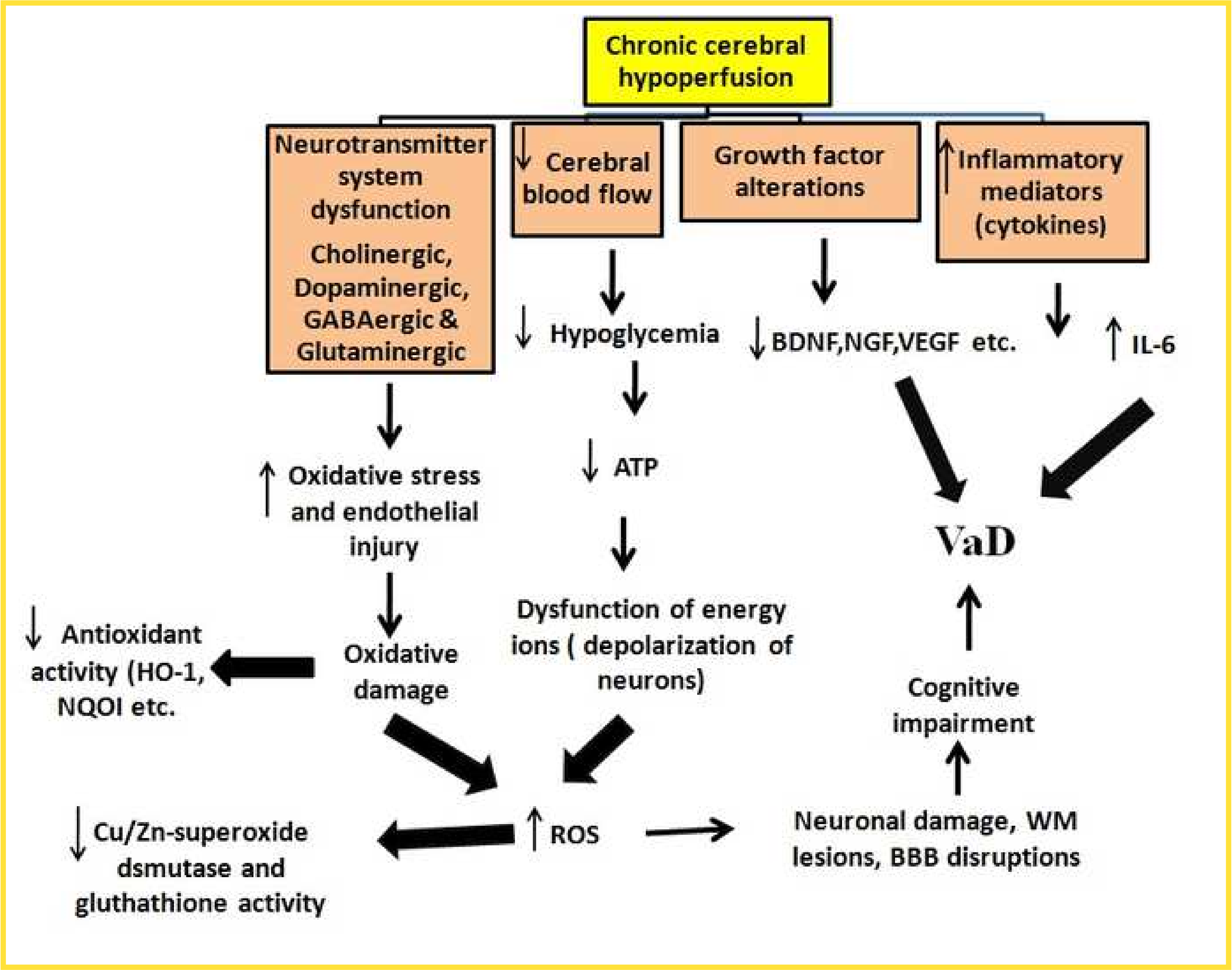

Table

All tables in both versions of the publication are similar, but not identical.

Table 1: Experimental models of vascular dementia

S. No.	Types of Model	Name of Model
1	Vessel occlusion models	2 Vessel occlusion (2VO) model<br>4 Vessel Occlusion model (Clamping of both common carotid arteries and both vertebral arteries)<br>Unilateral CCA (common carotid artery) occlusion (UCCAo) model<br>Bilateral common carotid artery (CCA) stenosis (BCCAS) model
2	Models using risk factors to induce VaD	Hypertension<br>(DOCA-salt induced hypertension induced VaD, Sodium-arsenite induced hypertension induced VaD Stroke prone spontaneously hypertensive model)<br>Diabetic rats and mouse (Streptozotocin induced VaD)<br>Hyperhomocysteine (L-Methionine induced VaD)<br>Age
3	M5R(-/-) transgenic mice Model	
4	CADASIL	
5	CAA	Cerebral amyloid angiopathy
6	High fat diet induced Models	
7	Multiple infarcts Models	Thromboembolic multiple infarction model<br>Micro-spheres induced multiple infarction models<br>Cholesterol crystals induced multiple infarction models

Table 2: Advantages and limitations of vessel occlusion models

Advantages	Limitations	References
i) The rat is a frequently used species in consequence of the good survival rate, satisfactory recovery from surgery, easy and reproducible behavioral testing, relatively low costs, and ethical acceptance.<br>ii) One-step surgical procedure that produces high-grade forebrain cognitive impairment.<br>iii) A high rate of predictable ischemic neuronal damage.<br>iv) The reproducibility of the VaD related histopathological damage of this model is more than 90%.<br>v) This model is suitable for molecular, biochemical, and physiological studies, as well as for evaluation of vasculoprotective procedures and agents.<br>vi) A model may be used in behavioral observations, studies for the evaluation of drug efficacy, and screening	i) This model is restricted to rats since they have a complete circle of willis and is not suitable for use in mouse since they lack fully developed posterior communicating arteries of the circle of Willis and can suffer from severe ischemia.<br>ii) Technically complex, higher rate of surgical trauma and high animal mortality<br>iii) Possibility of postischemic seizures especially in case of 4 VO<br>iv) Variability in surgical procedure and in occlusion durations may affect the results<br>v) In BCCAo, learning and memory dysfunction is reversible.<br>vi) In bilateral, there is mild short-term memory loss only.<br>vii) In BCCAO, there is damage to RGCs (Retinal ganglionic cell) and/or photoreceptors thus produce dysfunction in visual pathway/retinal perfusion. It has been shown that during	Farkas et al., 2007; Venkat et al., 2015; Wang, 2014; Kastner et al., 2005; Ueno et al., 2002; Stevens et al., 2002; Hachinski et al., 2006; Jiwa et al., 2010; Osborne et al., 1999; Kitamura et al., 2012

experiments and provides a valuable basis for the investigation of VaD.<br>vii) Ameroid constrictor device used for occlusion can predictably achieve gradual narrowing of the CCAo and produce cerebral hypoperfusion more accurately.<br>viii) Bilateral common carotid artery occlusion (BCCAO) is tolerated by adult rats due to an effective collateral flow through the basilar artery and the arterial circle of Willis.	BCCAO, blood flow in the retina may be more severely reduced than in the brain because the intraocular pressure increases the level of hypoperfusion thus causes visual impairment.<br>viii) In unilateral occlusion model, there may be chances that infarcts cannot be histologically detectable.<br>ix) Hypoperfusion to the brain produced in 2-VO model is relatively less severe than a 4-VO model.<br>x) There is a need for expert surgery.	

Table 3: Advantages and limitations of hypertension induced VaD

Advantages	Limitations	References
i) Non-invasive, relatively quick, consistent, economical and has a short time course.<br>ii) In case of SHR rats, there is lack of inter-individual variations.<br>iii) Ease and rapidity of producing the model.	i) Impairment in visual pathway<br>ii) Variable expression of symptoms<br>iii) It is a slowly developing model	Kaur et al., 2010; Yadav et al., 2011; Sharma and Sharma, 2013; Doggrell and Brown, 1998; Venkat et al., 2015

Table 4. Advantages and limitations of STZ induced VaD model

Advantages	Limitations	References
i) It is well-established model and the diabetes - induced VaD can be induced at any given age.<br>ii) VaD produced in animals may be thought to be corresponding to the symptoms shown in humans, as diabetes produced in this case i seems to be close resemblance to human type-1 diabetes mellitus.<br>iii) This model has been proven very useful for producing chronic hyperglycemia induced VaD.	i) Careful evaluation of whether VaD or AD has onset is required.<br>ii) STZ-diabetic rats develop end-organ damage that affects the eyes, kidneys, heart, blood vessels and nervous system. So care of the animal is required time to time.<br>iii) Higher mortality rate more than 20 % in the single dose (noted that STZ-treated diabetic rats, provide 10% sucrose water for 2 days after the STZ injection).	Gispen and Biessels, 2000; Venkat et al., 2015; Wu and Huan, 2008.

Table.5. Advantages and limitationsof hyper-homocystenemia induced VaD

Advantages	Limitations	References
i) Easy and reliable model to induce VaD.<br>ii) The level of homocysteine can be managed by vitamin B6, vitamin B9, vitamin B12 including taurine supplementation	i) There are alterations in several metabolites due to the elevation of homocysteine that directly influences vascular pathophysiology	Jakubowski, 2006; Ahn, 2009; Dayal and Lentz, 2008.

Table 6: Advantages and Limitations of Age-induced VaD

Advantages	Limitations	References
i) Non-invasive and natural model to produce VaD.<br>ii) No alteration in the central neurochemical manipulations as there is no drug or chemical compound is required to produce VaD<br>iii) Aged animals (roughly defined as >half normal lifespan) can exhibit cognitive and neuropathological changes resembling human disease	i) The Requirement for using an increased number of animals because of age-related high mortality rate.	Neha et al., 2014; Jiwa et al., 2010; Manwani et al., 2014; Lindner et al., 2003; Bruley-Rosset et al., 1981.

Table 7. Advantages and limitations of High-fat diet-induced VaD

Advantages	Limitations	References
i) One advantage of the animal models is the opportunity of precise control of diet and motor activity.<br>ii) HFD enables the monitory of the connection between body mass, the number of consumed fats and the effect from the use of different types of fats.<br>iii) Easy and simple to introduce VaD.<br>iv) This model mimics some features of cognitive impairment related dementia and the importance of increased level of cholesterol in the pathophysiology of vascular endothelial dysfunction and related dementia.	i) Variability in the duration of HFD and in a percentage of high fat and source of fat may influence the results.<br>ii) Time-consuming.	Venkat et al., 2015; Angelova and Boyadjiev, 2013; Neha et al., 2014.

Table 8: Advantages and Limitations of Multiple infarct model of VaD

Advantages	Limitations	References
i) Endothelial damage and subsequent thrombotic occlusion are selectively induced in carotid perforating arteries<br>ii)The microthrombotic occlusion associated with small cerebral infarcts of almost equal in size and neurological deficits can be observed in most rats	i) Short-term or transient deficits<br>ii) Variability in emboli material, size, and numbers make comparisons difficult	Venkat et al., 2015; Toshima et al., 2000
