# Supplementary material for: Fake paper identification in the pool of withdrawn and rejected manuscripts submitted to Naunyn–Schmiedeberg’s Archives of Pharmacology
Source: Naunyn Schmiedebergs Arch Pharmacol. 2023 Oct 5;397(4):2171–81. doi: 10.1007/s00210-023-02741-w (PMC10933159; doi:10.1007/s00210-023-02741-w)
Supplement: Supplementary file 11 — Supplementary file11 (PDF 103 KB) [file 210_2023_2741_MOESM11_ESM.pdf]

Figure S11

Dear. Prof. Roland Seifert

Thank you for your prompt reply!

Firstly: please allow me to introduce myself briefly. My name is Robin. My major is computer algorithm. 1. I do not work in a university. I am responsible for the research and development of new energy in a new energy enterprise in China. My research field is "the application of artificial intelligence in the future new energy". 2. There are many friends around me who need to publish various scientific research papers (SCI （Science Citation Index， SCI） or SSCI （Social Sciences Citation Index）). You can understand that I am an intermediary, and this is my sideline.

3. I read your published paper and felt that your major was what my friends needed, so I took the liberty to send an email to the email address on your paper.

Secondly: In order not to waste too much of your time, please allow me to give you a detailed description of my expected cooperation needs:

1. Cooperation mode 1: Please help my friends write the paper. The paper will be published in the name of the authors I provide. Your name will not appear on the paper. If you have a written paper to transfer, I also welcome it.

A) Who selects the journal: The journal is selected by you, but it needs to be retrieved by SCI or SSCI. You can inform me of the journals you are sure to publish, and I will try my best to choose the appropriate journals from the ones you tell me.

B) How to decide the paper title: You can tell me your familiar research field, and I will give you the general paper title according to the research field you give, then you can determine the specific paper title and start writing.

C) What are your responsibilities? You are responsible for the writing and publishing of papers.

D) How to give the thank-you fee: I will give you a thank-you fee of 2000 dollars for each paper.

2. Cooperation mode 2: You are allowed to add new authors to the articles you are ready to publish or will be accepted

A) What kind of author position is required: independent first author or independent correspondence author.

B) Please send me the following information: the article title, abstract, the name of the journal to be published, the estimated time when the article will be accepted by the journal (I know it cannot be accurate, but the estimated time is enough), the current preparation status of the article (for example, the article is ready to submit, the article has been submitted and is being reviewed, etc.), the author position you will transfer (for example, the corresponding author, the first author, etc.), and the final number of authors of the article.

C) Thank you fee: If the first author or corresponding author is transferred, the thank you fee is US $1000 and half of the APC fee will be borne.

3. Payment method of the thank you fee: 50% of the thank you fee will be paid when the article receives the acceptance notice of the journal, and the remaining 50% of the thank you fee will be paid by transfer when the article is officially published.

I look forward to working with you in both of the above modes! If I offend you, please accept my most sincere apology!

Thank you again!
We look forward to your reply!
Warmly Regards
Robin
